# 2-Aryl-6-Polyfluoroalkyl-4-Pyrones as Promising R^F^-Building-Blocks: Synthesis and Application for Construction of Fluorinated Azaheterocycles

**DOI:** 10.3390/molecules26154415

**Published:** 2021-07-21

**Authors:** Sergey A. Usachev, Diana I. Nigamatova, Daria K. Mysik, Nikita A. Naumov, Dmitrii L. Obydennov, Vyacheslav Y. Sosnovskikh

**Affiliations:** Institute of Natural Sciences and Mathematics, Ural Federal University, 51 Lenina Ave., 620000 Ekaterinburg, Russia; s.a.usachev@urfu.ru (S.A.U.); nigamatova.di@yandex.ru (D.I.N.); mysik.darya@gmail.com (D.K.M.); niquezor@yandex.ru (N.A.N.); dobydennov@mail.ru (D.L.O.)

**Keywords:** β-diketones, 4-pyrones, fluorinated heterocycles, regioselective bromination

## Abstract

A convenient and general method for the direct synthesis of 2-aryl-6-(trifluoromethyl)-4-pyrones and 2-aryl-5-bromo-6-(trifluoromethyl)-4-pyrones has been developed on the basis of *one-pot* oxidative cyclization of (*E*)-6-aryl-1,1,1-trifluorohex-5-ene-2,4-diones via a bromination/dehydrobromination approach. This strategy was also applied for the preparation of 2-phenyl-6-polyfluoroalkyl-4-pyrones and their 5-bromo derivatives. Conditions of chemoselective enediones bromination were found and the key intermediates of the cyclization of bromo-derivatives to 4-pyrones were characterized. Synthetic application of the prepared 4-pyrones has been demonstrated for the construction of biologically important CF_3_-bearing azaheterocycles, such as pyrazoles, pyridones, and triazoles.

## 1. Introduction

**Figure 1:**
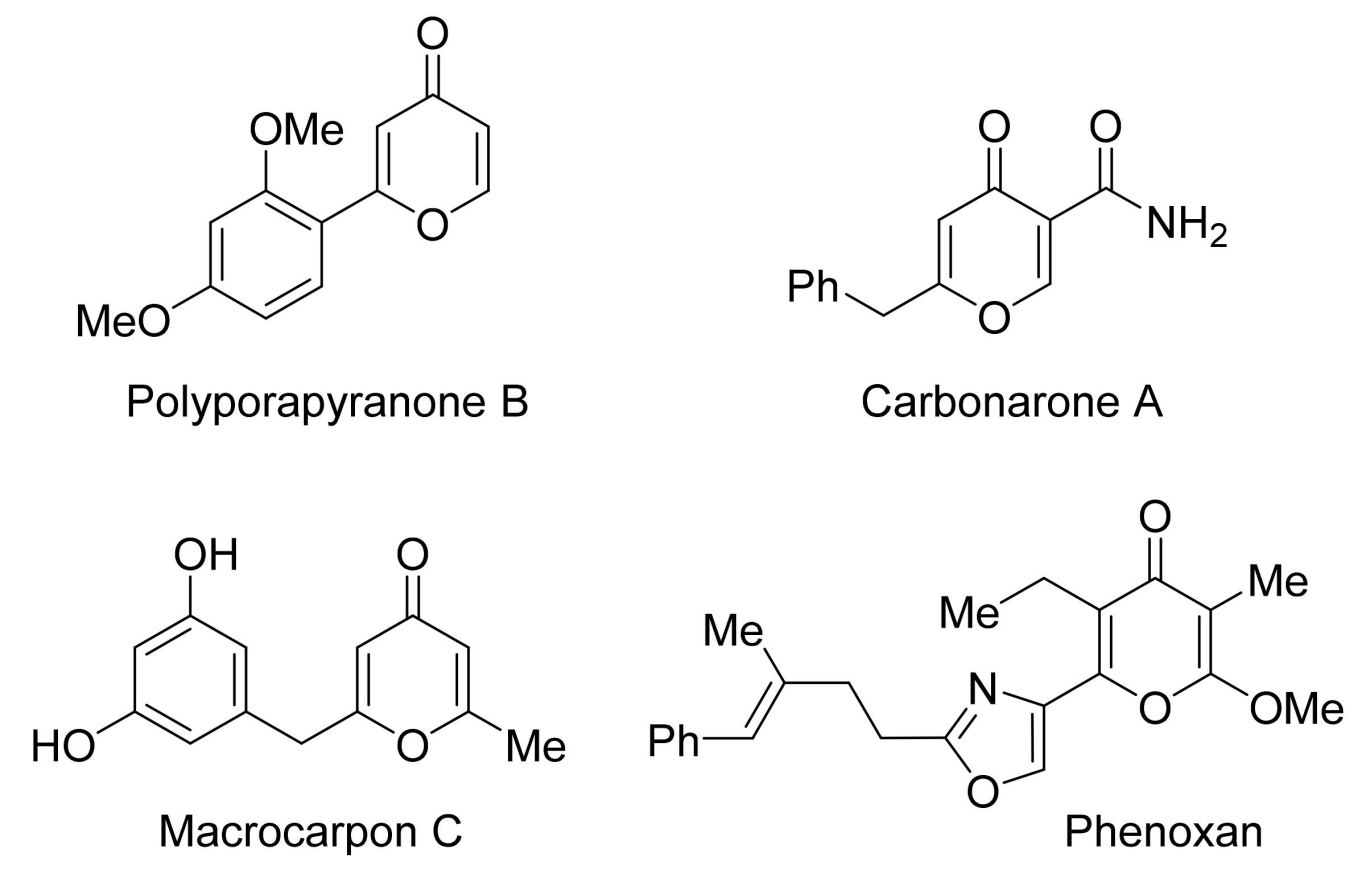
Some representative examples of biologically important arylated 4-pyrones.

4-Pyrone unit is an important heterocyclic motif, which is frequently present in numerous natural products that exhibit a wide range of important biological activities [1,2,3,4,5] and medicines, such as phenoxan [5] (Figure 1). Mainly, pyrones are of interest as useful and versatile building-blocks for the synthesis of valuable molecules [6,7,8,9], including pharmaceuticals [10] and pyranic fluorophores [11]. Pyrones as polyelectrophilic substrates and cyclic enone are able to react regioselectively with nucleophiles to undergo ring-opening transformations affording azaheterocycles [12,13,14,15,16]. The formation of the product is usually very sensitive to the reaction conditions (acidity, solvent polarity, temperature) that allows divergent syntheses on the basis of 4-pyrones [15,16].

It is known that fluorine-bearing compounds are of considerable interest for medicinal chemistry and agrochemistry [17]. The search for readily available and highly functionalized fluorinated building blocks for the construction of complex molecules is an important task for the synthesis of new bioactive substances.

To date considerable attention has been focused on 4-pyrones bearing the electron-withdrawing R^F^ group, which demonstrate higher reactivity with high chemoselectivity of processes compared to non-fluorinated analogs [17,18]. The ring-opening reactions of 2-R^F^-4-pyrones can be applied for the preparation of a wide range of trifluoromethylated azaheterocycles, such as indoles, regioisomeric pyrazoles, pyridines, benzodiazepines etc., [14,16,17,18].

**Scheme 1:**
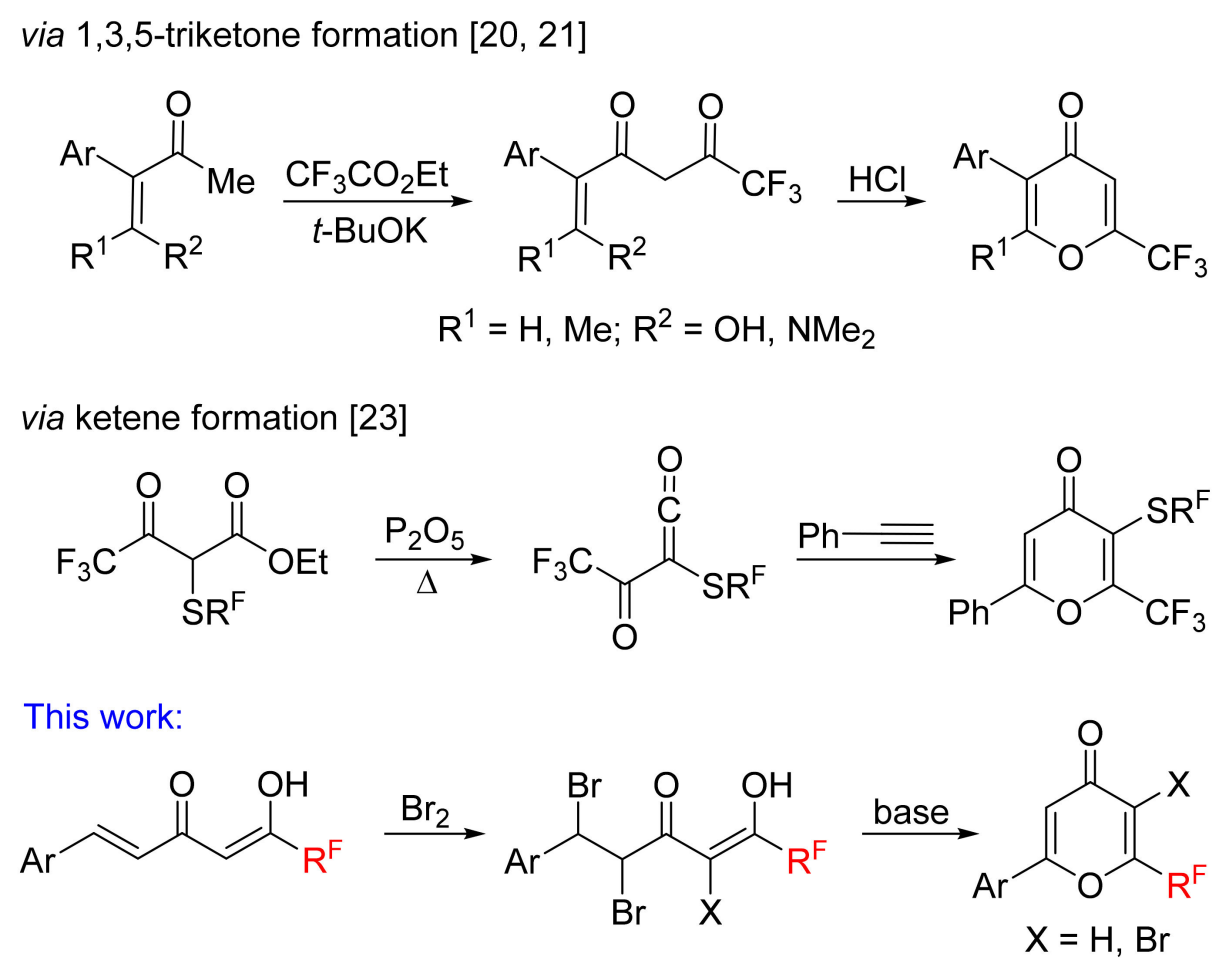
The main strategies in the synthesis of arylated 2-R^F^-4-pyrones [20,21,23].

Despite the promising synthetic utility of CF_3_-bearing 4-pyrones, they remain to be hard-to-reach compounds [17,18]. The methods for the construction of 4-pyrones are actively developed, and in the literature there are three general approaches for the synthesis of 2-R^F^-4-pyrones based on (i) cyclization of tricarbonyl compounds or their derivatives prepared via Claisen condensation [19,20,21,22], (ii) cycloadditions of ketenes [23,24,25], and (iii) modification of substituted 2-R^F^-4-pyrones (usually for carboxylic acid derivatives) via side-chain transformations [26] (Scheme 1). However, there are usually some limitations connected with the narrow scope of obtained pyrones, variation of the nature of the substituents in several positions of the pyrone ring, and preparation of 4-pyrones bearing long-chain polyfluoroakyl substituents.

Inspired by the chemistry of 2-CF_3_-chromones (benzopyrones) [27] and 2-CF_3_-4-pyrones [18], we decided to find an approach for the construction of a range of novel arylated 2-R^F^-4-pyrones as useful building-blocks for the preparation of fluorinated heterocycles. The number of 2-aryl-6-(trifluoromethyl)-4-pyrones described in the literature is limited by several examples [23,24,25], and there is also the publication regarding the reaction of 2-phenyl-6-(trifluoromethyl)-4-pyrone and salicylic aldehyde with no mention of its synthesis [28]. Thus, there is no general method for the synthesis of the fluorinated 2-aryl-4-pyrones at the moment.

Recently, methods for the preparation of pyrones based on the cyclization of acetylenyl-1,3-diketones have been actively developed [29,30,31,32,33], which allows the switchable preparation of 4-pyrones or furanones. Similar approach is the use of dibromo derivatives, which can react as synthetic equivalents of acetylenyl-1,3-diketones and undergo dehydrobromination reactions under basic conditions [34,35,36,37]. This method was applied for the preparation of 2-aryl-4-pyrones bearing the methyl and carboethoxy substituents. However, low selectivity of the bromination reaction of the starting enediones, difficult isolation of intermediate products, low yields, and very high sensitivity to the nature of the substituents strongly restrict its utilization.

The chemical properties of 6-aryl-1,1,1-trifluorohex-5-ene-2,4-diones remain unexplored, but they can be considered as a platform for the preparation of new fluorinated compounds [38,39,40,41]. The presence of several functional groups (conjugated double bonds and the diketone moiety) in their molecules makes them attractive and useful tools in organic synthesis. The introduction of the strong electron-withdrawing trifluoromethyl group can lead to a change in the chemoselectivity of the bromination reaction of 6-aryl-1,1,1-trifluorohex-5-ene-2,4-diones in comparison with non-fluorinated analogs and to changes in the cyclization step due to the ability of this group to stabilize cyclic and hemiacetal forms [17].

In the present work, we have developed a general method for the preparation of 2-aryl-6-polyfluoroalkyl-4-pyrones and their 3-bromo derivatives based on *one-pot* cyclization through the stage of bromination/dehydrobromination of enediones. The synthetic potential of the pyrones was demonstrated on the example of regioselective reactions with N-nucleophiles.

## 2. Results and Discussion

**Scheme 2:**
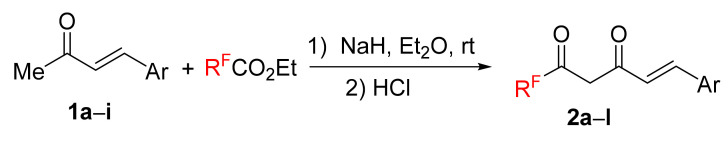
Synthesis of enediones **2** via Claisen condensation.

We started our research with the preparation of fluorinated enediones **2** based on the condensation reaction of 4-aryl-3-buten-2-ones **1** with ethyl polyfluoroalkanoates in the presence of sodium hydride in diethyl ether at room temperature or lithium hydride in benzene under reflux (Scheme 2, Table 1). Both methods were comparably effective, and CF_3_-bearing products **2a**–**i** were obtained in 51–92% yields. Compounds **2a**,**f**–**h** have been described in the literature, but they were prepared by other methods [38,39,40,41]. Besides, this approach was applied for the preparation of polyfluorinated enediones **2j–l** in 43–51%.

**Scheme 3:**
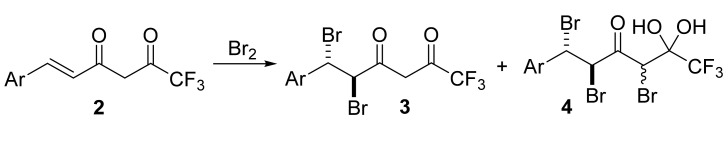
Reaction of enediones **2** with Br_2_.

We first focused on the bromination of CF_3_-containing compounds **2a–i** in order to obtain active intermediates and to develop a general method for the construction of pyrones based on enediones **2**. Both the double bond and the diketone moiety in 6-aryl-1,1,1-trifluorohex-5-ene-2,4-diones **2** can undergo an electrophilic attack of bromine. To estimate the influence of conditions on conversion, the chemo- and stereoselectivity (see detailed data in Supplementary Materials), the bromination reaction was examined for compound **2b** bearing the *para*-fluorophenyl substituent (Scheme 3, Table 2). It was shown that the most active fragment is the double bond that leads to the initial formation of dibromo derivative **3b,** and no product of initial attack only at the methylene group of the diketone moiety was found. At the same time, compounds **3** due to the absence of conjugation were able to react with Br_2_ by the diketone moiety at a comparable rate with the starting enediones **2** giving tribromo derivatives **4**.

When 1 equiv. of bromine was used, incomplete conversion was usually observed, accompanied by the formation of tribromo derivative **4b**. Bromination in acetic acid gave low conversion (26%) and predominant formation of the tribromo derivative (**3b**:**4b** = 9:17) (entry 1). Carrying out the reaction in aprotic polar solvents CH_2_Cl_2_ and *t*-BuOMe made it possible to increase the conversion to 58 and 83%, respectively, but the selectivity turned out to be low (entries 2,4). The use of non-polar aprotic solvents (dioxane, CS_2_) led to the improved **3b**:**4b** ratio and 71–75% conversion (entries 5,6). When the reaction was carried out in benzene, the highest conversion (88%), the highest NMR yield of **3b**, and the good selectivity of the reaction were found (**3b**:**4b** = 78:10) (entry 7). A slight increase in the amount of bromine (up to 1.2 equiv.) led to a decrease in selectivity (**3b**:**4b** = 79:21), although the starting compound **2b** was not detected at all (entry 8).

Further investigation showed that the bromination reaction is very sensitive to the nature of the substituents in the aromatic ring (Scheme 3, Table 2, and Table S1 in Supplementary Materials). Thus, the reaction proceeded with the highest selectivity with one equivalent of bromine in benzene for compounds bearing moderate and good *π*-donors (4-MeO, 4-F or Ar = 2-Th) (entries 7,10,11). The introduction of electron-withdrawing substituents (4-Cl, 3-NO_2_) into the aromatic ring decreased the selectivity of the transformation for compounds **2c,e** due to incomplete conversion and the formation of tribromo derivatives **4** as major products (entries 12,13). Even in the case of enedione **2a** (Ar = Ph), selectivity and conversion of bromination in benzene turned out to be poor (entry 14), however, the use of CH_2_Cl_2_ as a solvent improved the selectivity and the conversion (entry 15). The introduction of the strong electron-donating substituent (4-Me_2_N) led to preferential bromination at the aromatic ring rather than at the double bond followed by cyclization because of basic properties of the substituent (Scheme S1 in Supplementary Materials). It should be noted that, in all cases, compounds **3** were formed as a sole *anti*-dibromo diastereoisomer according to the classical concept of electrophilic addition of bromine to alkenes via the cyclic bromonium cation [42].

Further, we decided to develop a procedure for the bromination of both fragments of enediones **2** simultaneously for the selective preparation of tribromo derivatives **4**. The reaction of enedione **2b** with an excess of bromine (2.2 equiv.) in benzene gave products with low selectivity (**3b**:**4b** = 56:44) (Table 2, entry 9). Moreover, significant amounts of corresponding dihydropyrones were formed as a result of spontaneous acid-catalyzed cyclization. One of the highest yields of compound **4b** in reaction with 1 equiv. of bromine was observed in dichloromethane (entry 2). The use of bromine (2.5 equiv.) in this solvent led to the almost selective formation of tribromo derivative **4b** as a mixture of two diastereoisomers in a ratio of 80:20 (entry 3).

**Scheme 4:**
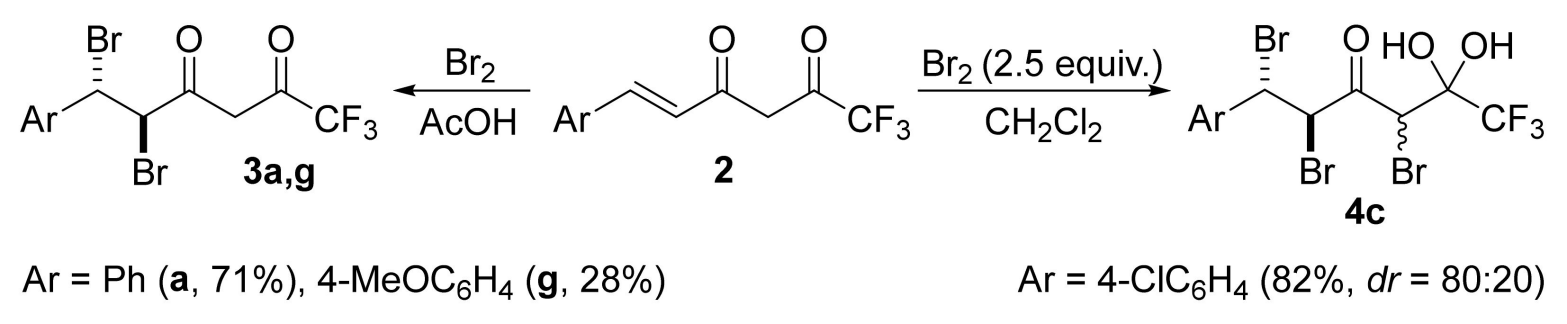
Preparative bromination of enediones **2**.

Although the reaction of enediones **2** with Br_2_ gave dibromo derivatives **3** or tribromo derivatives **4** as major products, we did not usually isolate them in pure form to avoid additional losses because of their ability to cyclize and high reactivity. But for several cases, these compounds were obtained as a single isomer by recrystallization. The reaction of enediones **2a,g** with one equivalent of bromine in acetic acid made it possible to obtain dibromo derivatives **3a** and **3g** in 71 and 28% yields, respectively (Scheme 4).

The reaction of chloro-substituted enedione **2c** with an excess of bromine (2.5 equiv.) in CH_2_Cl_2_ led to tribromo derivative **4c** (*dr* = 80:20) smoothly in 82% yield. Compound **4c** in pure crystalline form prepared by recrystallization from hexane is stable and can be stored for a long time without changes.

The structure of tribromo derivatives **4** was established on the basis of elemental analysis data and ^1^H and ^19^F NMR spectra (Table S3 in Supplementary Materials). In the ^1^H NMR spectra of these compounds in CDCl_3_, two singlets of the diastereotopic OH groups were observed at *δ* 4.06–4.46 and 4.62–4.79 ppm. The ^19^F NMR spectra exhibit the CF_3_ group at *δ* 79.7–79.9 ppm indicating its location at the saturated carbon atom. For compounds **2** and **3**, the characteristic chemical shifts of the trifluoromethyl group appeared at *δ* 84.5–85.0 and 86.0 ppm, respectively (Table S2 in Supplementary Materials).

**Scheme 5:**
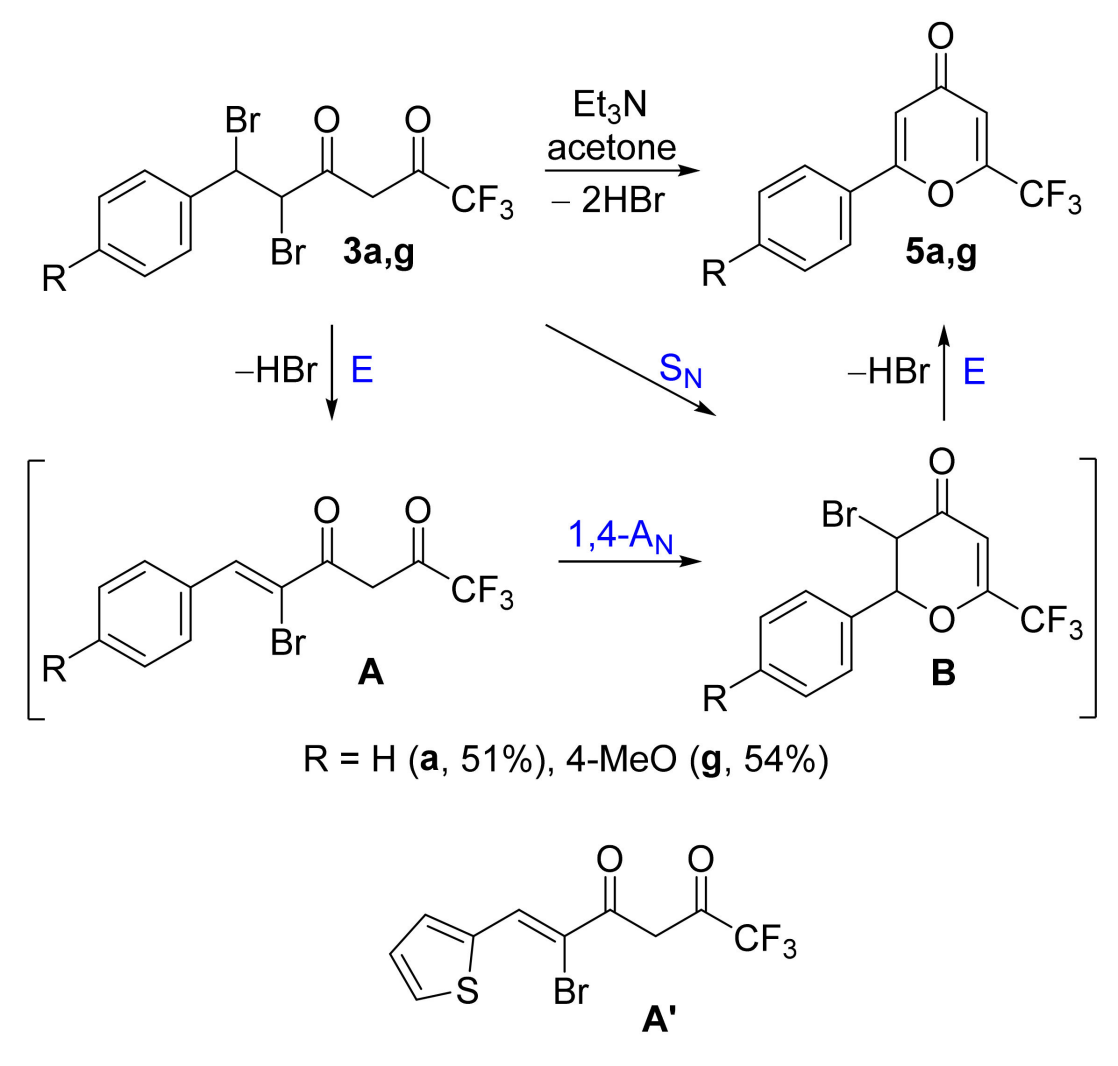
Cyclization of dibromo derivatives **3** to pyrones **5**.

At the next stage of our work, we studied the cyclization of dibromo derivatives **3a,g** in the presence of bases. The use of *t*-BuOK in THF or pyridine, DIPEA, DBU, NaHCO**_3_** in various solvents (acetone, ethanol, DMSO) at room temperature did not allow us to obtain 4-pyrone **5a** in an acceptable yield. The most favorable conditions turned out to be stirring with a 2.5-fold excess of triethylamine in acetone for three days (Scheme 5). In addition, the isolation of the product, in this case, does not require chromatography, and pyrones **5a,g** precipitated in pure form upon dilution with water in 51–54% yields. This approach can be scaled up to 5 g of enedione **2a**, and the yield of pyrone **5a** was 36% in two stages.

The cyclization mechanism of compounds **3** includes the formation of dihydropyran intermediate **B** via two possible pathways. The first path comprises the elimination of hydrogen bromide (E) and the production of bromoenone **A**, which then undergoes an intramolecular Michael reaction (1,4-A_N_). The second possibility is the direct intramolecular nucleophilic substitution of bromine (S_N_). As mentioned above, the cyclic pyranic form **B** was observed in the bromination reaction mixtures (Table 2), whereas bromoenones **A** were detected by NMR spectroscopy on the treatment of the dibromo derivatives **3** with pyridine at room temperature. The easy formation of 2-bromoenones from 2,3-dibromoketones is also well presented in the literature [42]. Thus, it can be assumed that the first path is more preferable in basic conditions. Intermediate **A′** was isolated in pure form with the use of column chromatography (56% yield) from enedione **2i** via bromination (1 equiv.) and subsequent monodehydrobromination by DIPEA at room temperature.

**Scheme 6:**
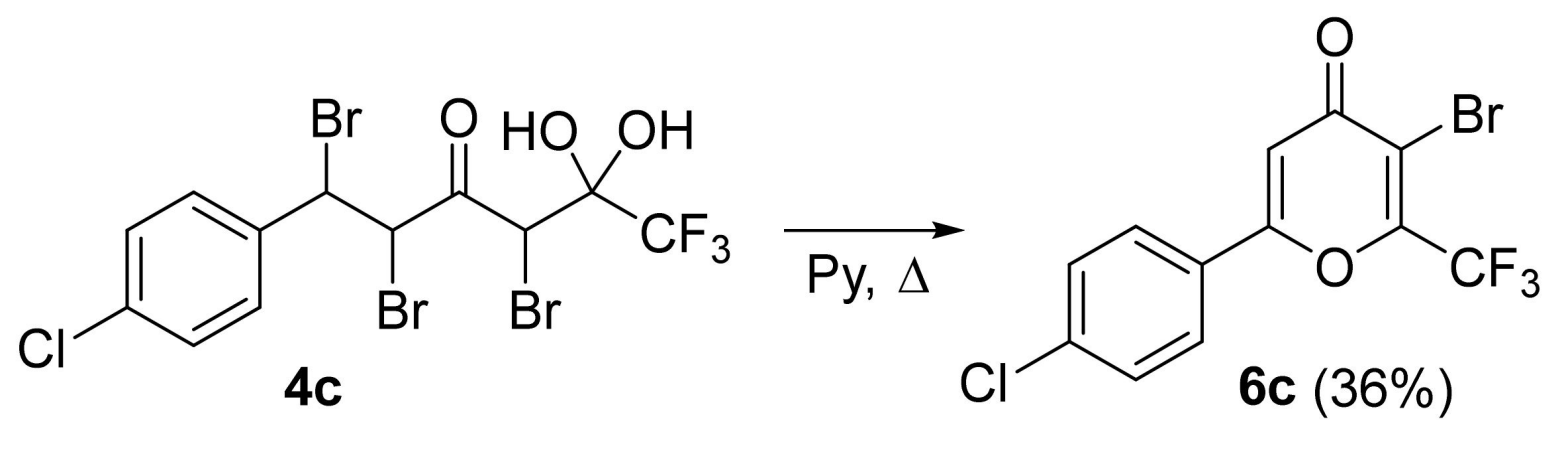
Cyclization of tribromo derivative **4c** to pyrone **6c**.

Tribromo derivatives **4** undergo cyclization under the action of triethylamine in acetone at room temperature much slower, furthermore, the high basicity of the medium could provide a possibility of side reactions associated with detrifluoroacetylation [41]. Therefore, dehydrohalogenation of **4** was carried out by refluxing for 10 h in pyridine as a solvent and a weaker base (Scheme 6). The cyclization of compound **4c** gave pyrone **6c** in 36% yield. These results demonstrate that the cyclization step determines mainly total output of the pyrones. Moreover, the difference in reaction rates of cyclization of tribromo derivatives **4** and dibromo derivatives **3** allowed the isolation of pyrones **5** in the presence of Et_3_N in acetone selectively, even when the reaction mixtures of the brominated intermediates were used.

**Scheme 7:**
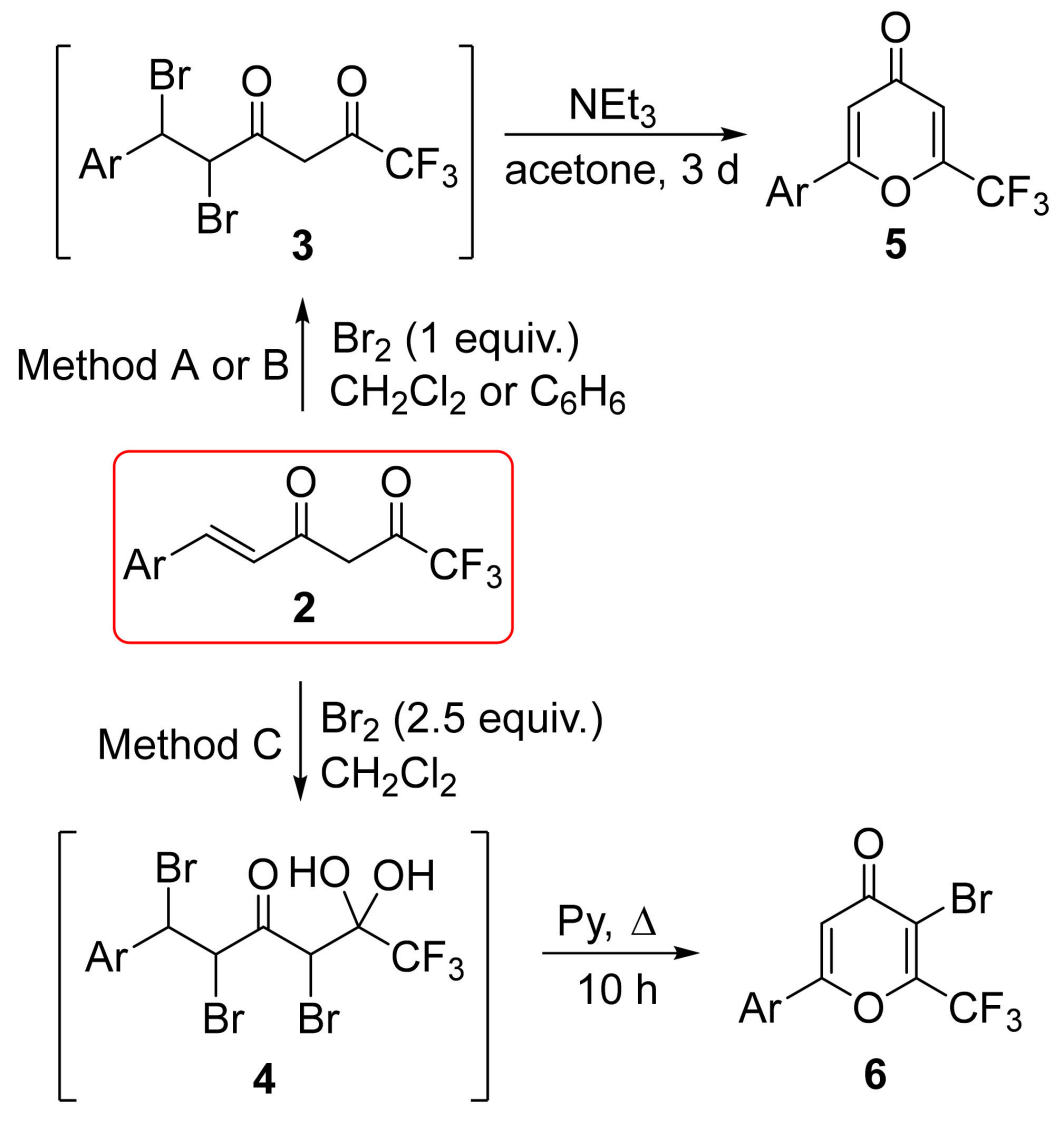
*One-pot* preparation of pyrones **5** and **6** from enediones **2**.

With the optimized conditions of the bromination and cyclization of intermediate bromo derivatives in hand, we decided to develop a simple *one-pot* two-step preparative method for the synthesis of 4-pyrones **5** and **6** based on oxidative cyclization of enediones **2**. Considering that the reaction mixtures of bromination contain various bromine-bearing forms (cyclic and acyclic), the overall efficiency and convenience of this two-step process without intermediate purification can improve (Scheme 7, Table 3).

For the preparation of 4-pyrones **5** in the *one-pot* approach, enediones **2** were treated with 1 equiv. of bromine in benzene (Method A) for compounds bearing electron-donating substituents (F, Me, MeO) and in CH_2_Cl_2_ (Method B) for compounds bearing weak electron-withdrawing substituents (Cl, Br) or H at the C-4 position. After bromination, the solvent was evaporated, and the cyclization was carried out using Et_3_N (2.5 equiv.) in acetone, as a result, pyrones **5** were obtained in 28–69% yields.

The preparation of 3-bromo-4-pyrones **6** with a *one-pot* method was possible only for substrates bearing weak electron-donating (H, 4-Me, 4-F) and electron-withdrawing groups (4-Cl, 3-NO_2_) in the benzene ring since aromatic bromination was unavoidable with an excess of bromine in case of highly activated aryl substituents. At the first stage, enedione **2** was treated with 2.5 equiv. of bromine in CH_2_Cl_2_ and subsequent heating at 100 °C in pyridine for 10 h led to the formation of pyrones **6** in 4–42% yields (Method C).

**Scheme 8:**
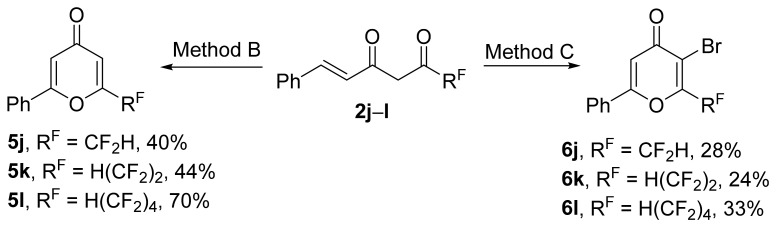
Synthesis of 2-aryl-6-polyfluoroalkyl-4-pyrones **6j–l**.

This *one-pot* approach was also applied for the preparation of phenyl-4-pyrones bearing various polyfluoroalkyl substituent. No significant difference in reactivity was observed depending on fluorine content or chain length of R^F^-moiety, and the cyclization of enediones **2j**–**l** in the presence of 1 equiv. of bromine (Method B) gave 4-pyrones **5j–l** in 40–70% yields (Scheme 8). The use of Method C led to 3-bromopyrones **6j–l** in 24–33% yields.

The structure of 4-pyrones **5** and **6** was established on the basis of elemental analysis data, HRMS and NMR spectra. In the ^1^H NMR spectra of compounds **5** in CDCl_3_, characteristic doublets of H-3 and H-5 protons of the pyrone ring were observed at *δ* 6.63–6.88 ppm with a coupling constant of 1.8–2.2 Hz. In the case of pyrones **6**, the signal of the H-5 proton of the pyrone ring appeared as a singlet at *δ* 6.88–7.04 ppm (CDCl_3_).

**Scheme 9:**
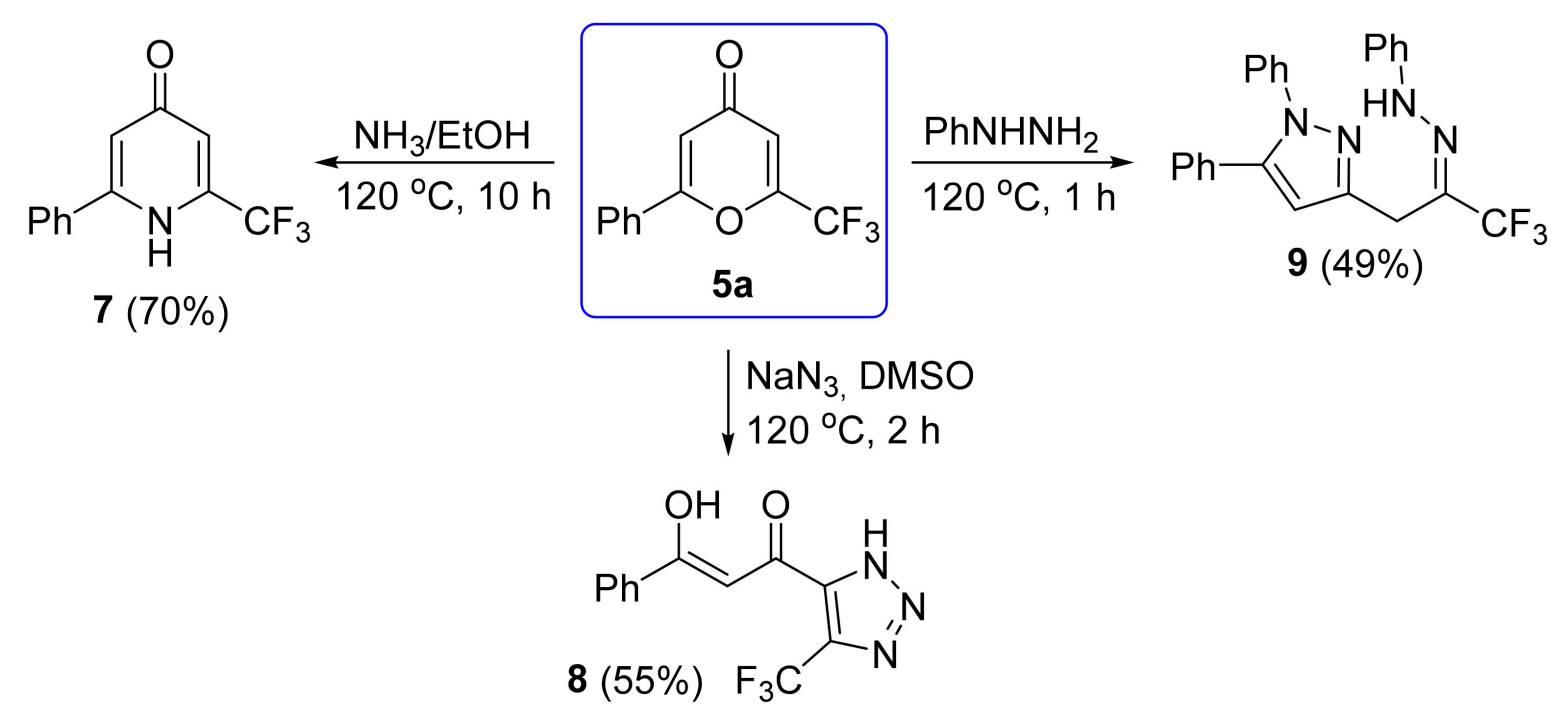
Reaction of pyrone **5a** with N-nucleophiles for the synthesis of fluorinated azaheterocycles.

The next step was to study the chemical properties of 2-aryl-6-(trifluoromethyl)-4-pyrones with N-nucleophiles to obtain azaheterocycles (Scheme 9). The reaction of pyrone **5a** with ammonia in ethanol in an autoclave at 120 °C for 10 h led to the formation of pyridone **7** in 70% yield. The ring-opening transformation of pyrone **5a** with sodium azide [43] in DMSO afforded triazole **8** in 55% yield. Phenylhydrazine reacted with pyrone **5a** regioselectively attacking with the amino group at the C-2 and C-4 positions to give pyrazole **9**. The structure of pyrazole **9** was assigned based on NMR spectra according to the literature data [16,34].

Thus, a convenient and general method for the synthesis of 2-aryl-6-polyfluoroalkyl-4-pyrones and their bromo derivatives has been developed based on *one-pot* oxidative cyclization of fluorinated enediones. The selectivity of monobromination of 6-aryl-1,1,1-trifluorohex-5-ene-2,4-diones is connected with the predominant electrophilic attack on the double bond and strongly depends on the solvent used and the nature of the substituent in the aromatic ring, which determines the scope of obtained fluorinated 2-aryl-4-pyrones. 6-Aryl-1,1,1-trifluorohex-5-ene-2,4-diones bearing electron-withdrawing and weak electron-donating substituents in the aromatic ring react with an excess of bromine leading to tribromo derivatives as a result of bromination of the double bond and the diketone moiety. Di- and tribromo derivatives in a basic medium selectively undergo cyclization to form 4-pyrones. The resulting 4-pyrones have proven to be useful building blocks and have been utilized to synthesize CF_3_-bearing heterocycles, which are of further interest in terms of potential biological activity.

## 3. Materials and Methods

NMR spectra were recorded on Bruker DRX-400 (^1^H: 400 MHz, ^13^C: 100 MHz, ^19^F: 376.5 MHz) and Bruker Avance III-500 (^1^H: 500 MHz, ^13^C: 126 MHz, ^19^F: 471 MHz) spectrometers in DMSO-*d*_6_ and CDCl_3_. The chemical shifts (*δ*) are reported in ppm relative to the internal standard TMS (^1^H NMR), C_6_F_6_ (^19^F NMR), and residual signals of the solvents (^13^C NMR). IR spectra were recorded on a Shimadzu IRSpirit-T spectrometer using an attenuated total reflectance (ATR) unit (FTIR mode, ZnSe crystal), the absorbance maxima (*ν*) are reported in cm^–1^. Elemental analyses were performed on an automatic analyzer PerkinElmer PE 2400. Melting points were determined using a Stuart SMP40 melting point apparatus. Column chromatography was performed on silica gel (Merck 60, 70–230 mesh). All solvents that were used were dried and distilled by standard procedures. Arylideneacetones **1** have been synthesized according to the procedure, previously described in the literature [44].

### 3.1. Synthesis of Enediones ***2a**–**l***

General Procedure. A suspension of NaH (60% by weight in mineral oil) (240 mg, 6.0 mmol) in dry Et_2_O (5 mL) was cooled on an ice bath, then CF_3_CO_2_Et (0.86 g, 6.0 mmol) and a solution of arylideneacetone 1 (4.0 mmol) in dry Et_2_O (3 mL) were successively added under stirring. The reaction mixture was warmed to room temperature and stirred for 20 h. Then 1M aqueous HCl (10 mL) was added, and the organic phase was separated (if the precipitate of diketonate formed, it was filtered and treated with 1M aqueous HCl (10 mL)). The aqueous phase was extracted with AcOEt (5 mL). The combined organics were washed with H_2_O and dried over anhydrous Na_2_SO_4_, the solvents evaporated, and the residue recrystallized from hexane with decantation of hot solution from insoluble tarry by-products.

General procedure (with LiH) [38]. A solution of arylideneacetone 1 (4.0 mmol) in dry C_6_H_6_ (1 mL) and CF_3_CO_2_Et (0.86 g, 6.0 mmol) were successively added to a suspension of LiH (48 mg, 6.0 mmol) in dry C_6_H_6_ (4 mL) under stirring. The reaction mixture was warmed until the reaction started and then was stirred at room temperature for 20 h. After that, the reaction mixture was poured into a mixture of ice (4 g) and H_2_SO_4_ (1 mL), extracted with chloroform (5 mL), and dried over MgSO_4_. The solvents were evaporated, and the residue recrystallized from hexane with decantation of hot solution from insoluble tarry by-products.

*(E)-1,1,1-Trifluoro-6-phenylhex-5-ene-2,4-dione* (**2a**). Yield 0.73 g (75%), colorless needles, mp 56–58 °C (lit. mp 58 °C [40], 61–61.5 °C [38]).^1^H NMR (400 MHz, CDCl_3_) enol form: *δ* 6.04 (1H, s, 3-CH); 6.58 (1H, d, *J* = 15.9 Hz, 5-CH); 7.37–7.57 (3H, m, H Ph); 7.53–7.61 (2H, m, H-2, H-6 Ph); 7.77 (1H, d, *J* = 15.9 Hz, 6-CH); 14.22 (1H, s, OH). The analytical data are in consistence with the literature [38,40].

*(E)-1,1,1-Trifluoro-6-(4-fluorophenyl)hex-5-ene-2,4-dione* (**2b**). Yield 0.67 g (64%), yellow prisms, mp 83–84 °C. IR (ATR) ν 1637, 1578, 1509, 1444, 1414. ^1^H NMR (500 MHz, CDCl_3_) enol form: *δ* 6.02 (1H, s, 3-CH); 6.50 (1H, d, *J* = 15.8 Hz, 5-CH); 7.12 (2H, t, *J* = 8.6 Hz, *J*_HF_ = 8.6 Hz, H-3, H-5 Ar); 7.57 (2H, dd, *J* = 8.7 Hz, *J* = 5.3 Hz, H-2, H-6 Ar); 7.73 (1H, d, *J* = 15.8 Hz, 6-CH); 14.24 (1H, s, OH). ^19^F NMR (376 MHz, CDCl_3_) *δ* 53.9 (tt, *J*_HF_ = 8.4 Hz, *J*_HF_ = 5.4 Hz, F); 84.7 (s, CF_3_). ^13^C NMR (126 MHz, CDCl_3_) *δ* 95.6 (q, ^3^*J*_CF_ = 1.4 Hz, C-3); 116.4 (d, ^2^*J*_CF_ = 22.0 Hz, C-3, C-5 Ar); 116.7 (q, ^1^*J*_CF_ = 286.6 Hz, CF_3_); 120.8 (d, ^6^*J*_CF_ = 2.4 Hz, C-5); 130.4 (s, C-1 Ar); 130.5 (d, ^4^*J*_CF_ = 8.8 Hz, C-2, C-6 Ar); 142.3 (C-6); 164.3 (d, ^1^*J*_CF_ = 253.2 Hz, C-4 Ar); 180.5 (q, ^2^*J*_CF_ = 36.1 Hz, C-2); 180.8 (C-4). HRMS (ESI) *m/z* [M + H]^+^. Calcd for C_12_H_9_F_4_O_2_: 261.0539. Found: 261.0533.

*(E)-6-(4-Chlorophenyl)-1,1,1-trifluorohex-5-ene-2,4-dione* (**2c**). Yield 0.66 g (60%), yellow needles, mp 92–93 °C. IR (ATR) ν 3072, 3035, 1632, 1610, 1573, 1561, 1491, 1454, 1410. ^1^H NMR (500 MHz, CDCl_3_) enol form: *δ* 6.03 (1H, s, 3-CH); 6.54 (1H, d, *J* = 15.8 Hz, 5-CH); 7.40 (2H, d, *J* = 8.5 Hz, H Ar); 7.50 (2H, d, *J* = 8.5 Hz, H Ar); 7.71 (1H, d, *J* = 15.8 Hz, 6-CH); 14.17 (1H, s, OH). ^19^F NMR (471 MHz, CDCl_3_) *δ* 84.6 (s, CF_3_). ^13^C NMR (126 MHz, CDCl_3_) δ 95.80 (q, ^3^*J*_CF_ = 1.4 Hz, C-3); 116.63 (q, ^1^*J*_CF_ = 285.6 Hz, CF_3_); 121.5 (s, C-5); 129.4 (s, 2H); 129.6 (s, 2H); 132.7 (s, C Ar); 137.1 (s, C Ar); 142.1 (s, C-6); 180.4 (s, C-4); 180.8 (q, ^2^*J*_CF_ = 36.1 Hz, C-2). Anal. Calcd for C_12_H_8_ClF_3_O_2_: C 52.10; H 2.91. Found: C 51.93; H 2.94%.

*(E)-6-(4-Bromophenyl)-1,1,1-trifluorohex-5-ene-2,4-dione* (**2d**). Yield 1.18 g (92%), white crystals, mp 86–87 °C. IR (ATR) ν 1632, 1609, 1582, 1487, 1454, 1406. ^1^H NMR (400 MHz, CDCl_3_) enol form: *δ* 6.03 (1H, s, 3-CH); 6.56 (1H, d, ^3^*J* = 15.9 Hz, 5-CH); 7.43 (2H, d, ^3^*J* = 8.4 Hz, H Ar); 7.56 (2H, d, ^3^*J* = 8.4 Hz, H Ar); 7.69 (1H, d, ^3^*J* = 15.9 Hz, 6-CH); 14.15 (1H, s, OH). ^19^F NMR (376 MHz, CDCl_3_) *δ* 84.7 (s, CF_3_). ^13^C NMR (101 MHz, CDCl_3_) *δ* 95.8 (unresolved q, C-3); 116.7 (q, ^1^*J*_CF_ = 285.5 Hz, CF_3_); 121.6 (C-5); 125.5 (C Ar); 129.8 (2C Ar); 132.4 (2C Ar); 133.1 (C Ar); 142.1 (C-6); 180.4 (C-4); 180.8 (q, ^2^*J*_CF_ = 36.2 Hz, C-2). Anal. Calcd for C_12_H_8_BrF_3_O_2_: C 44.89; H 2.51. Found: C 45.03; H 2.57%.

*(E)-1,1,1-Trifluoro-6-(3-nitrophenyl)hex-5-ene-2,4-dione* (**2e**). Yield 0.59 g (51%), white scales, mp 118–119 °C. IR (ATR) ν 1644, 1610, 1577, 1523, 1477, 1429. ^1^H NMR (400 MHz, CDCl_3_) enol form: *δ* 6.10 (1H, s, 3-CH); 6.70 (1H, d, ^3^*J* = 15.9 Hz, 5-CH); 7.63 (1H, t, ^3^*J* = 8.0 Hz, H-5 Ar); 7.79 (1H, d, ^3^*J* = 15.9 Hz, 6-CH); 7.86 (1H, dt, ^3^*J* = 7.7 Hz, ^4^*J* = 1.0 Hz, H-6 Ar); 8.27 (1H, ddd, ^3^*J* = 8.5 Hz, ^4^*J* = 2.1 Hz, ^4^*J* = 0.8 Hz, H-4 Ar); 8.44 (1H, t, ^4^*J* = 1.8 Hz, H-2 Ar); 13.96 (1H, s, OH). ^19^F NMR (376 MHz, CDCl_3_) *δ* 84.5 (s, CF_3_). HRMS (ESI) *m/z* [M – H]^–^. Calcd for C_12_H_7_F_3_NO_4_: 286.0327. Found: 286.0331.

*(E)-1,1,1-Trifluoro-6-(p-tolyl)hex-5-ene-2,4-dione* (**2f**). Yield 0.67 g (65%), yellow needles, mp 61–62 °C. IR (ATR) ν 3031, 2956, 2926, 2360, 1652, 1594, 1575, 1516, 1447, 1416. ^1^H NMR (400 MHz, CDCl_3_) enol form: *δ* 2.40 (3H, s, Me); 6.01 (1H, s, 3-CH); 6.53 (1H, d, *J* = 15.8 Hz, 5-CH); 7.23 (2H, d, *J* = 8.0 Hz, H-3, H-5 Ar); 7.47 (2H, d, *J* = 8.1 Hz, H-2, H-6 Ar); 7.75 (1H, d, *J* = 15.8 Hz, 6-CH); 14.30 (1H, s, OH). ^19^F NMR (376 MHz, CDCl_3_) *δ* 84.7 (s, CF_3_). ^13^C NMR (101 MHz, CDCl_3_) *δ* 21.6 (CH_3_); 95.4 (q, ^3^*J*_CF_ = 1.5 Hz, C-3); 116.8 (q, ^1^*J*_CF_ = 285.3 Hz, CF_3_); 120.0 (C-5); 128.6 (2C Ar); 129.9 (2C Ar); 131.5 (C Ar); 141.9 (C Ar); 143.90 (C-6); 180.1 (q, ^2^*J*_CF_ = 36.0 Hz, C-2); 181.5 (C-4). Anal. Calcd for C_13_H_11_F_3_O_2_: C 60.94; H 4.33. Found: C 60.96; H 4.47%. The analytical data are in consistence with the literature [41].

*(E)-1,1,1-Trifluoro-6-(4-methoxyphenyl)hex-5-ene-2,4-dione* (**2g**). Yield 0.88 g (81%), yellow needles, mp 100–101 °C (lit. mp 98 °C [40]). IR (ATR) ν 1633, 1568, 1511, 1453, 1441, 1420. ^1^H NMR (500 MHz, CDCl_3_) enol form: *δ* 3.86 (3H, s, OMe); 6.00 (1H, s, 3-CH); 6.45 (1H, d, ^3^*J* = 15.8 Hz, 5-CH); 6.94 (2H, d, ^3^*J* = 8.8 Hz, H-3, H-5 Ar); 7.53 (2H, d, ^3^*J* = 8.4 Hz, H-2, H-6 Ar); 7.74 (1H, d, ^3^*J* = 15.8 Hz, 6-CH); 14.45 (1H, s, OH). ^19^F NMR (471 MHz, CDCl_3_) *δ* 84.8 (s, CF_3_). ^13^C NMR (101 MHz, CDCl_3_) *δ* 55.5 (OMe); 95.2 (q, ^2^*J*_CF_ = 1.2 Hz, C-3); 114.7 (2C Ar); 116.9 (q, ^1^*J*_CF_ = 285.1 Hz, CF_3_); 118.6 (C-5); 126.9 (C Ar); 130.5 (2C Ar); 143.7 (C-6); 162.2 (C Ar); 179.7 (q, ^2^*J*_CF_ = 35.9 Hz, C-2); 181.9 (C-4). Anal. Calcd for C_13_H_11_F_3_O_3_: C 57.36; H 4.07. Found: C 57.38; H 4.20%. The analytical data are in consistence with the literature [40].

*(E)-6-(4-(Dimethylamino)phenyl)-1,1,1-trifluorohex-5-ene-2,4-dione* (**2h**). Yield 0.76 g (67%), magenta needles, mp 79–80 °C (lit. mp 78–79 °C [39]). IR (ATR) ν 2900, 1556, 1519, 1453, 1430, 1360, 1328. ^1^H NMR (500 MHz, CDCl_3_) enol form: *δ* 3.07 (6H, s, NMe_2_); 5.95 (1H, s, 3-CH); 6.35 (1H, d, ^3^*J* = 15.6 Hz, 5-CH); 6.68 (2H, d, ^3^*J* = 8.9 Hz, H-3, H-5 Ar); 7.47 (2H, d, ^3^*J* = 8.9 Hz, H-2, H-6 Ar); 7.74 (1H, d, ^3^*J* = 15.6 Hz, 6-CH); 14.69 (1H, s, OH). ^19^F NMR (471 MHz, CDCl_3_) *δ* 85.0 (s, CF_3_). Anal. Calcd for C_14_H_14_F_3_NO_2_: C 58.95; H 4.95; N 4.91. Found: C 58.92; H 4.94; N 4.79%. The analytical data are in consistence with the literature [39].

*(E)-1,1,1-Trifluoro-6-(thiophen-2-yl)hex-5-ene-2,4-dione* (**2i**). Yield 0.83 g (84%), yellow rectangular prisms, mp 94–95 °C. IR (ATR) ν 1577, 1503, 1437, 1416. ^1^H NMR (400 MHz, CDCl_3_) enol form: *δ* 5.99 (1H, s, 3-CH); 6.36 (1H, d, ^3^*J* = 15.5 Hz, 5-CH); 7.11 (1H, dd, ^3^*J* = 4.9 Hz, ^3^*J* = 3.8 Hz, H-4 Th); 7.35 (1H, d, ^3^*J* = 3.5 Hz, H-3 Th); 7.48 (1H, d, ^3^*J* = 5.0 Hz, H-5 Th); 7.88 (1H, d, ^3^*J* = 15.5 Hz, 6-CH); 14.28 (1H, s, OH). ^19^F NMR (376 MHz, CDCl_3_) *δ* 84.8 (s, CF_3_). ^13^C NMR (101 MHz, CDCl_3_) *δ* 95.5 (unresolved q, C-3); 116.8 (q, ^1^*J*_CF_ = 285.3 Hz, CF_3_); 119.8 (C-5); 128.6; 130.2; 132.4; 136.2; 139.8; 180.1 (q, ^2^*J*_CF_ = 36.0 Hz, C-2); 180.8 (C-4). Anal. Calcd for C_10_H_7_F_3_O_2_S: C 48.39; H 2.84. Found: C 48.36; H 2.75%.

*(E)-1,1-Difluoro-6-phenylhex-5-ene-2,4-dione* (**2j**). Yield 0.39 g (43%), yellow crystals, mp 85–87 °C. IR (ATR) ν 3030, 1647, 1495, 1341, 1180, 1105, 867, 757. ^1^H NMR (500 MHz, CDCl_3_) enol form: *δ* 5.91 (1H, t, ^2^*J*_HF_ = 54.3 Hz, CF_2_H); 6.04 (1H, s, 3-CH); 6.58 (1H, d, *J* = 15.9 Hz, 5-CH); 7.39–7.44 (3H, m, H Ph); 7.51–7.62 (2H, m, H-2, H-6 Ph); 7.72 (1H, d, *J* = 15.9 Hz, 6-CH); 14.58 (1H, s, OH). ^19^F NMR (471 MHz, CDCl_3_) *δ* 35.0 (dd, ^2^*J*_HF_ = 54.4 Hz, ^4^*J*_HF_ = 0.5 Hz, CF_2_H). ^13^C NMR (126 MHz, CDCl_3_) *δ* 96.0 (t, ^3^*J*_CF_ = 1.9 Hz, C-4); 109.6 (t, ^1^*J*_CF_ = 249.0 Hz, CF_2_H); 121.8 (C-6); 128.4 (2C Ph); 129.1 (2C Ph); 130.8 (C Ph); 134.4 (C Ph); 142.7 (C-7); 181.0 (C-5); 186.5 (t, ^2^*J*_CF_ = 25.7 Hz, C-3). Anal. Calcd for C_12_H_10_F_2_O_2_: C 64.29; H 4.50. Found: C 64.69; H 4.35.

*(E)-6,6,7,7-Tetrafluoro-1-phenylhept-1-ene-3,5-dione* (**2k**). Yield 0.47 g (43%), yellow liquid. IR (ATR) ν 3028, 1633, 1580, 1449, 1244, 1110, 700. ^1^H NMR (400 MHz, CDCl_3_) enol form: *δ* 6.10 (tt, ^2^*J*_HF_ = 52.9 Hz, ^3^*J*_HF_ = 5.1 Hz, CF_2_H); 6.13 (1H, s, 4-CH); 6.58 (1H, d, *J* = 15.9 Hz, 6-CH); 7.40–7.45 (3H, m, H Ph); 7.53–7.61 (2H, m, H-2, H-6 Ph); 7.77 (1H, d, *J* = 15.9 Hz, 7-CH); 14.45 (1H, s, OH). ^19^F NMR (376 MHz, CDCl_3_) *δ* 23.4 (dt, ^2^*J*_HF_ = 52.9 Hz, ^3^*J*_FF_ = 6.9 Hz, CF_2_H); 35.8 (tdd, ^3^*J*_FF_ = 6.9 Hz, ^3^*J*_HF_ = 5.1 Hz, ^4^*J*_HF_ = 0.8 Hz, CF_2_). ^13^C NMR (126 MHz, CDCl_3_) *δ* 96.8 (unresolved t, C-4); 109.1 (tt, ^1^*J*_CF_ = 251.2 Hz, ^2^*J*_CF_ = 34.1 Hz, CF_2_H); 109.5 (tt, ^1^*J*_CF_ = 258.5 Hz, ^2^*J*_CF_ = 27.8 Hz, CF_2_); 121.3 (C-6); 128.4 (2C Ph); 129.1 (2C Ph); 131.0 (C Ph); 134.3 (C Ph); 143.6 (C-7); 180.1 (q, ^2^*J*_CF_ = 26.7 Hz, C-3); 180.9 (C-5). HRMS (ESI) *m/z* [M + H]^+^. Calcd for C_13_H_11_F_4_O_2_: 275.0695. Found: 275.0694.

*(E)-6,6,7,7,8,8,9,9-Octafluoro-1-phenylnon-1-ene-3,5-dione* (**2l**). Yield 0.76 g (51%), yellow liquid. IR (ATR) ν 2929, 1633, 1580, 1450, 1172, 1034, 972, 787. ^1^H NMR (500 MHz, CDCl_3_) *δ* enol form (88%): 6.08 (1H, s, 4-CH); 6.12 (1H, tt, ^2^*J*_HF_ = 52.0 Hz, ^3^*J*_HF_ = 5.5 Hz, CF_2_H); 6.58 (1H, d, *J* = 15.8 Hz, 2-CH); 7.39–7.47 (3H, m, H Ph); 7.54–7.61 (2H, m, H-2, H-6 Ph); 7.79 (1H, d, *J* = 15.8 Hz, 1-CH); 14.33 (1H, s, OH); keto form (12%): 2.77 (2H, s, CH_2_); 6.08 (1H, tt, ^2^*J*_HF_ = 52.0 Hz, ^3^*J*_HF_ = 5.5 Hz, CF_2_H); 6.46 (1H, d, *J* = 15.9 Hz, 2-CH); 7.39–7.47 (3H, m, H Ph); 7.54–7.61 (2H, m, H-2, H-6 Ph); 7.81 (1H, d, *J* = 15.9 Hz, 1-CH). ^19^F NMR (471 MHz, CDCl_3_) *δ* enol form (88%): 24.4 (dm, ^2^*J*_HF_ = 51.9 Hz, CF_2_H); 32.0 –32.1 (m, 8-CF_2_); 37.0 (t, ^3^*J*_FF_ = 8.2 Hz, 7-CF_2_); 40.5 (t, ^3^*J*_FF_ = 9.9 Hz, 6-CF_2_); keto form (12%): 24.4 (dm, ^2^*J*_HF_ = 51.9 Hz, CF_2_H); 31.9–32.0 (m, 8-CF_2_); 36.9 (t, ^3^*J*_FF_ = 8.2 Hz, 7-CF_2_); 42.3 (t, ^3^*J*_FF_ = 9.6 Hz, 6-CF_2_). ^13^C NMR (126 MHz, CDCl_3_) *δ* 97.2 (unresolved m, C-6); 107.6 (tt, ^1^*J*_CF_ = 254.7 Hz, ^2^*J*_CF_ = 30.9 Hz, CF_2_H); 107.7–113.5 (m, 3CF_2_); 121.0 (C-8); 128.5 (2C Ph); 129.1 (2C Ph); 131.2 (C Ph); 134.2 (C Ph); 144.0 (C-9); 180.5 (C-7); 182.5 (t, ^2^*J*_CF_ = 26.7 Hz, C-5). HRMS (ESI) *m/z* [M + H]^+^. Calcd for C_15_H_11_F_8_O_2_: 375.0631. Found: 375.0627.

### 3.2. Synthesis of Compounds ***3a**,**g***

General procedure. A solution of Br_2_ (3.20 g, 0.020 mol) in glacial AcOH (5.7 mL) was added dropwise to enedione 2 (0.020 mol) in glacial AcOH (18 mL) cooled by a water bath. After that, the reaction mixture was stirred for 1 h at room temperature. The resulting solution was diluted by water (30 mL). The precipitate that formed was filtered. If it is needed, the pure product can be obtained by recrystallization from heptane.

*(5S,6R)-5,6-Dibromo-1,1,1-trifluoro-6-phenylhexane-2,4-dione* (**3a**). Yield 5.71 g (71%), beige powder, mp 119–120 °C. IR (ATR) ν 1665, 1629, 1604, 1497, 1458, 1434. ^1^H NMR (400 MHz, DMSO-*d*_6_) enol form: *δ* 5.56 (1H, d, ^3^*J* = 11.7 Hz, CHBr); 6.08 (1H, d, ^3^*J* = 11.7 Hz, CHBr); 6.30 (1H, s, 3-CH); 7.29–7.46 (3H, m, H Ph); 7.49–7.54 (2H, m, H-2, H-6 Ph). ^1^H NMR (400 MHz, CDCl_3_) *δ* 4.96 (1H, d, ^3^*J* = 11.6 Hz, CHBr); 5.39 (1H, d, ^3^*J* = 11.6 Hz, CHBr); 6.24 (1H, s, 3-CH); 7.38–7.50 (5H, m, H Ph). ^19^F NMR (471 MHz, CDCl_3_) *δ* 86.0 (s, CF_3_). ^13^C NMR (126 MHz, CDCl_3_) *δ* 48.8; 51.2; 96.5; 117.1 (q, *J* = 280.4 Hz, CF_3_); 128.0; 129.0; 129.6; 137.4; 171.9 (q, *J* = 37.2 Hz, C-2); 190.6. HRMS (ESI) *m/z* [M – H]^–^. Calcd for C_12_H_8_Br_2_F_3_O_2_: 398.8843. Found: 398.8835.

*(5S,6R)-5,6-Dibromo-1,1,1-trifluoro-6-(4-methoxyphenyl)hexane-2,4-dione* (**3g**). Yield 2.42 g (28%), yellow powder, mp 124–125 °C. IR (ATR) ν 1665, 1607, 1514, 1462, 1443. ^1^H NMR (500 MHz, CDCl_3_) enol form: *δ* 3.84 (3H, s, OMe); 4.93 (1H, d, ^3^*J* = 11.6 Hz, CHBr); 5.37 (1H, d, ^3^*J* = 11.6 Hz, CHBr); 6.21 (1H, s, 3-CH); 6.92 (2H, d, ^3^*J* = 8.6 Hz, H-3, H-5 Ar); 7.35 (2H, d, ^3^*J* = 8.6 Hz, H-2, H-6 Ar); 13.50 (1H, s, OH). ^19^F NMR (376 MHz, CDCl_3_) *δ* 86.0 (s, CF_3_). Anal. Calcd for C_13_H_11_Br_2_F_3_O_3_: C 36.14; H 2.57. Found: C 36.48; H 2.73%.

### 3.3. Synthesis of Compound ***4c***

A solution of Br_2_ (400 mg, 2.5 mmol) in CH_2_Cl_2_ (2 mL) was added to enedione **2c** (277 mg, 1 mmol) in CH_2_Cl_2_ (8 mL) under cooling on an ice-bath. The reaction mixture was left at room temperature for 15 h, and then the solvent was evaporated without heating. The residue is a mixture of diastereoisomeres (*dr* = 80:20), which was recrystallized from hexane to obtain single isomer in pure form for analysis.

*(1Rʹ,2Sʹ)-1,2,4-Tribromo-1-(4-chlorophenyl)-6,6,6-trifluoro-5,5-dihydroxyhexan-3-one* (**4c**). Yield 0.44 g (82%), white powder, mp 125–128 °C (toluene-hexane). After recrystallization from hexane, mp 154–155 °C (for the major diastereoisomer in pure form). IR (ATR) ν 3015, 1714, 1597, 1493, 1295, 1207, 990, 830, 715. ^1^H NMR (500 MHz, CDCl_3_) *δ* major diastereomer (80%): 4.38 (1H, s, OH); 4.74 (1H, s, OH); 4.95 (1H, s, 4-CHBr); 5.24 (1H, d, ^3^*J* = 11.3 Hz, 2-CHBr); 5.35 (1H, d, ^3^*J* = 11.3 Hz, 1-CHBr); 7.32–7.43 (4H, m, H Ar); minor diastereomer (20%): 3.94 (1H, s, OH); 4.58 (1H, s, OH); 4.98 (1H, s, 4-CHBr); 5.19 (1H, d, ^3^*J* = 11.3 Hz, 2-CHBr); 5.27 (1H, d, ^3^*J* = 11.3 Hz, 1-CHBr); 7.34–7.41 (4H, m, H Ar). ^19^F NMR (471 MHz, CDCl_3_) *δ* major diastereomer: 79.7 (s, CF_3_). Anal. Calcd for C_12_H_9_Br_3_ClF_3_O_3_: C 27.02; H 1.70. Found: C 27.32; H 1.79%.

### 3.4. Synthesis of Compound ***A’***

A solution of dibromide **3i**, which was prepared by reaction of enedione **2i** (248 mg, 1 mmol) with Br_2_ (160 mg, 1 mmol) in CH_2_Cl_2_ (3.4 mL) without additional purification, and DIPEA (323 mg, 2.5 mmol) in CH_2_Cl_2_ (5 mL) was stirred at room temperature for 15 h. The reaction mixture was then quenched with aqueous HCl (10 mL, 0.5 M), the organic phase was separated, washed with water and brine, dried under Na_2_SO_4_, and evaporated. The crude product was purified by column chromatography (CH_2_Cl_2_).

*5-Bromo-1,1,1-trifluoro-6-(thiophen-2-yl)hex-5-ene-2,4-dione* (**A’**). Yield 183 mg (56%), dark yellow solid, mp 84–85 °C. IR (ATR) ν 1623, 1573, 1413. ^1^H NMR (500 MHz, CDCl_3_) enol form: *δ* 6.69 (1H, s, 3-CH); 7.22 (1H, dd, ^3^*J* = 4.9 Hz, ^3^*J* = 3.5 Hz, H-4 Th); 7.65 (1H, d, ^3^*J* = 3.5 Hz, H-3 Th); 7.72 (1H, d, ^3^*J* = 4.9 Hz, H-5 Th); 8.53 (1H, s, 6-CH); 14.77 (1H, s, OH). ^19^F NMR (471 MHz, CDCl_3_) *δ* 85.5 (s, CF_3_). ^13^C NMR (126 MHz, CDCl_3_) *δ* 95.0 (q, ^3^*J*_CF_ = 2.1 Hz, C-4); 112.4; 117.2 (q, ^1^*J*_CF_ = 283.1 Hz, CF_3_); 127.6; 133.2; 134.1; 136.9; 137.7; 176.2 (q, ^2^*J*_CF_ = 37.1 Hz, C-2); 182.3 (C-4). HRMS (ESI) *m/z* [M + H]^+^. Calcd for C_10_H_7_BrF_3_O_2_S: 326.9302. Found: 326.9315.

### 3.5. Synthesis of Pyrones ***5***

General procedure. A solution of Br_2_ (160 mg, 1.0 mmol) in the solvent (1.7 mL) was added to enedione 2 (1 mmol) in the solvent (2.7 mL) under cooling on an ice-bath (C_6_H_6_ was used for Method A, CH_2_Cl_2_ – Method B). After that, the reaction mixture was stirred at room temperature for 15 h, and then the solvent was evaporated without heating. Then acetone (4.3 mL) was added to the residue, and the solution was treated with Et_3_N (0.253 g, 2.5 mmol) under cooling on an ice-bath. After that, the reaction mixture was stirred at room temperature for 3 days and was diluted with H_2_O (15 mL). The precipitate was filtered, washed with water, and recrystallized from hexane.

*2-Phenyl-6-(trifluoromethyl)-4H-pyran-4-one* (**5a**). Method B. Yield 115 mg (48%), pale-yellow fine crystals, mp 85–86 °C. *Procedure* from dibromide **3a**: Solutions of dibromide **3a** (2.00 g, 5.0 mmol) in acetone (20 mL) was treated with Et_3_N (1.26 g, 12.5 mmol) under cooling on an ice bath. After that, the reaction mixture was stirred at room temperature for 3 days and diluted with H_2_O (75 mL). The precipitate was filtered, washed with water, and recrystallized from hexane. Yield 51% (0.60 g), pale-yellow fine crystals, mp 85–86 °C. IR (ATR) ν 3068, 1668, 1632, 1605, 1579, 1498, 1451, 1415. ^1^H NMR (400 MHz, CDCl_3_) *δ* 6.76 (1H, d, ^4^*J* = 2.1 Hz, CH); 6.84 (1H, d, ^4^*J* = 2.1 Hz, CH); 7.50–7.61 (3H, m, H Ph); 7.76–7.80 (m, 2H, H-2, H-6 Ph). ^19^F NMR (471 MHz, CDCl_3_) *δ* 90.3 (s, CF_3_). ^13^C NMR (126 MHz, CDCl_3_) *δ* 112.3; 114.7 (q, ^3^*J*_CF_ = 2.3 Hz, C-5); 118.5 (q, ^1^*J*_CF_ = 274.8 Hz, CF_3_); 126.0 (2C Ar); 129.3 (2C Ar); 129.9; 132.2; 152.4 (q, ^2^*J*_CF_ = 39.6 Hz, C-6); 163.9; 178.0 (CO). Anal. Calcd for C_12_H_7_F_3_O_2_: C 60.01; H 2.94. Found: C 59.63; H 2.56%.

*2-(4-Fluorophenyl)-6-(trifluoromethyl)-4H-pyran-4-one* (**5b**). Method A. Yield 0.18 g (69%), beige powder, mp 109–110 °C. IR (ATR) ν 3064, 1674, 1637, 1601, 1509, 1291, 1085, 945, 838. ^1^H NMR (400 MHz, CDCl_3_) *δ* 6.78 (1H, d, ^4^*J* = 2.1 Hz, CH); 6.82 (1H, d, ^4^*J* = 2.1 Hz, CH); 7.19–7.25 (2H, m, H-3, H-5 Ar); 7.76–7.85 (2H, m, H-2, H-6 Ar). ^19^F NMR (471 MHz, CDCl_3_) *δ* 55.8 (tt, ^3^*J*_FH_ = 8.2 Hz, ^4^*J*_FH_ = 5.1 Hz, F-Ar); 90.3 (s, CF_3_). ^13^C NMR (126 MHz, CDCl_3_) *δ* 112.1 (d, ^6^*J*_CF_ = 0.8 Hz, C-4); 114.8 (q, ^3^*J*_CF_ = 2.6 Hz, C-5); 116.7 (d, ^2^*J*_CF_ = 22.3 Hz, C-3, C-5 Ar); 118.3 (q, ^1^*J*_CF_ = 273.5 Hz, CF_3_); 126.1 (d, ^4^*J*_CF_ = 3.3 Hz, C-1 Ar); 128.3 (d, ^3^*J*_CF_ = 9.0 Hz, C-2, C-6 Ar); 152.4 (q, ^2^*J*_CF_ = 39.4 Hz, C-6); 163.0 (C-2); 164.9 (d, ^1^*J*_CF_ = 253.2 Hz, C-4 Ar); 177.9 (CO). Anal. Calcd for C_12_H_6_F_4_O_2_·0.33H_2_O: C 54.57; H 2.54. Found: C 54.31; H 2.57%.

*2-(4-Chlorophenyl)-6-(trifluoromethyl)-4H-pyran-4-one* (**5c**). Method B. Yield 170 mg (62%), beige crystals, mp 135–137 °C. IR (ATR) ν 3061, 1671, 1632, 1605, 1492, 1416, 1407. ^1^H NMR (400 MHz, CDCl_3_) *δ* 6.76 (1H, d, ^4^*J* = 2.1 Hz, CH); 6.81 (1H, d, ^4^*J* = 2.1 Hz, CH); 7.51 (2H, d, ^3^*J* = 8.7 Hz, H Ar); 7.72 (2H, d, ^3^*J* = 8.7 Hz, H Ar). ^19^F NMR (376 MHz, CDCl_3_) *δ* 90.3 (s, CF_3_). ^13^C NMR (126 MHz, CDCl_3_) *δ* 112.4 (C-3); 114.8 (q, ^3^*J*_CF_ = 2.6 Hz, C-5); 118.4 (q, ^1^*J*_CF_ = 273.7 Hz, CF_3_); 127.3 (2C Ar); 128.3 (C Ar); 129.7 (2C Ar); 138.7; 152.4 (q, ^2^*J*_CF_ = 39.6 Hz, C-6); 162.8; 177.7 (CO). Anal. Calcd for C_12_H_6_ClF_3_O_2_: C 52.48; H 2.20. Found: C 52.47; H 2.40%.

*2-(4-Bromophenyl)-6-(trifluoromethyl)-4H-pyran-4-one* (**5d**). Method B. Yield 0.207 mg (65%), colorless powder, mp 161–163 °C. IR (ATR) ν 3059, 1668, 1654, 1645, 1627, 1600, 1590, 1488, 1414, 1402. ^1^H NMR (500 MHz, CDCl_3_) *δ* 6.76 (1H, d, ^4^*J* = 2.1 Hz, H-3); 6.82 (1H, d, ^4^*J* = 2.1 Hz, H-5); 7.64 (2H, d, ^3^*J* = 8.8 Hz, H Ar); 7.67 (2H, d, ^3^*J* = 8.8 Hz, H Ar). ^19^F NMR (471 MHz, CDCl_3_) *δ* 90.3 (s, CF_3_). ^13^C NMR (126 MHz, CDCl_3_) *δ* 112.4 (C-3); 114.9 (q, ^3^*J*_CF_ = 2.6 Hz, C-5); 118.4 (q, ^1^*J*_CF_ = 273.9 Hz, CF_3_); 127.1 (C Ar); 127.4 (2C Ar); 128.8 (C Ar); 132.6 (2C Ar); 152.4 (q, ^2^*J*_CF_ = 39.7 Hz, C-6); 162.9 (C-2); 177.7 (CO). Anal. Calcd for C_12_H_6_BrF_3_O_2_: C 45.17; H 1.90. Found: C 45.00; H 1.94%.

*2-(p-Tolyl)-6-(trifluoromethyl)-4H-pyran-4-one* (**5f**). Method B. Yield 71 mg (28%), colorless needles, 105–107 °C. IR (ATR) ν 1645, 1632, 1608, 1594, 1569, 1511, 1447. ^1^H NMR (500 MHz, CDCl_3_) *δ* 2.44 (3H, s, Me); 6.74 (1H, d, *J* = 2.1 Hz, H-5); 6.80 (1H, d, *J* = 2.1 Hz, H-3); 7.32 (2H, d, *J* = 8.1 Hz, H-3, H-5 Ar); 7.67 (2H, d, *J* = 8.1 Hz, H-2, H-6 Ar). ^19^F NMR (471 MHz, CDCl_3_) *δ* 90.3 (s, CF_3_). ^13^C NMR (126 MHz, CDCl_3_) *δ* 21.5 (CH_3_); 111.6 (C-3); 114.6 (q, ^3^*J*_CF_ = 2.5 Hz, C-5); 118.5 (q, ^1^*J*_CF_ = 273.8 Hz, CF_3_); 125.9 (2C Ar); 127.0 (C Ar); 130.0 (2C Ar); 143.1(C Ar); 152.3 (q, ^2^*J*_CF_ = 39.5 Hz, C-6); 164.1 (C-2); 178.0 (CO). HRMS (ESI) *m/z* [M + H]^+^. Calcd for C_13_H_10_F_3_O_2_: 255.0633. Found: 255.0625.

*2-(4-Methoxyphenyl)-6-(trifluoromethyl)-4H-pyran-4-one* (**5g**). Method A. Yield 176 mg (65%), yellow crystals, mp 106–107 °C. IR (ATR) ν 3019, 1685, 1598, 1522, 1489, 1432. ^1^H NMR (500 MHz, CDCl_3_) *δ* 3.89 (3H, s, Me); 6.73 (1H, d, ^4^*J* = 2.1 Hz, CH); 6.75 (1H, d, ^4^*J* = 2.1 Hz, CH); 7.02 (2H, d, ^3^*J* = 8.9 Hz, H-3, H-5 Ar); 7.73 (2H, d, ^3^*J* = 8.9 Hz, H-2, H-6 Ar). ^19^F NMR (376 MHz, CDCl_3_) *δ* 90.3 (s, CF_3_). ^13^C NMR (126 MHz, CDCl_3_) *δ* 55.5 (CH_3_); 110.7 (C-3); 114.6 (q, ^3^*J*_CF_ = 2.5 Hz, C-5); 114.7 (2C Ar); 118.6 (q, ^1^*J*_CF_ = 273.6 Hz, CF_3_); 122.1 (C Ar); 127.8 (2C Ar); 152.1 (q, ^2^*J*_CF_ = 39.4 Hz, C-6); 162.9; 164.0; 178.0 (CO). Anal. Calcd for C_13_H_9_F_3_O_3_: C 57.79; H 3.36. Found: C 57.39; H 3.39%.

*2-(Thiophen-2-yl)-6-(trifluoromethyl)-4H-pyran-4-one* (**5i**). Method A. Yield 153 mg (62%), yellow crystals, mp 87–88 °C. IR (ATR) ν 3042, 1668, 1652, 1630, 1594, 1427, 1408. ^1^H NMR (400 MHz, CDCl_3_) *δ* 6.68 (1H, d, *J* = 2.1 Hz, H-5); 6.71 (1H, d, *J* = 2.1 Hz, H-3); 7.19 (1H, dd, ^3^*J* = 4.9 Hz, ^3^*J* = 3.9 Hz, H-4 Th); 7.60 (1H, d, ^3^*J* = 4.9 Hz, H-5 Th); 7.65 (1H, d, ^3^*J* = 3.9 Hz, H-3 Th). ^19^F NMR (376 MHz, CDCl_3_) *δ* 90.3 (s, CF_3_). ^13^C NMR (126 MHz, CDCl_3_) *δ* 110.7 (C-3); 114.8 (q, ^3^*J*_CF_ = 2.4 Hz, C-5); 118.4 (q, ^1^*J*_CF_ = 274.2 Hz, CF_3_); 128.7 (C Ar); 129.1 (C Ar); 130.9 (C Ar); 132.9 (C Ar); 151.9 (q, ^2^*J*_CF_ = 39.7 Hz, C-6); 159.5; 177.4 (CO). HRMS (ESI) *m/z* [M + H]^+^. Calcd for C_10_H_6_F_3_O_2_S: 247.0041. Found: 247.0031.

*2-(Difluoromethyl)-6-phenyl-4H-pyran-4-one* (**5j**). Yield 40% (89 mg), yellow powder, mp 135–136 °C. IR (ATR) ν 1665, 1616, 1596, 1497, 1358, 1177, 1108, 1055, 925. ^1^H NMR (500 MHz, CDCl_3_) *δ* 6.47 (1H, t, ^2^*J* = 53.7 Hz, CHF_2_); 6.63 (1H, d, ^4^*J* = 2.2 Hz, H-3); 6.84 (1H, d, ^4^*J* = 2.2 Hz, H-5); 7.49–7.58 (3H, m, H Ph); 7.78 (2H, dd, ^3^*J* = 8.1 Hz, ^4^*J* = 1.1 Hz, H-2, H-6 Ph). ^19^F NMR (471 MHz, CDCl_3_) *δ* 38.3 (d, ^2^*J* = 53.7 Hz, CF_2_H). ^13^C NMR (126 MHz, CDCl_3_) *δ* 109.0 (t, ^1^*J*_CF_ = 242.9 Hz, CF_2_H); 112.2; 114.4 (t, ^3^*J*_CF_ = 4.0 Hz, C-3); 126.0 (2C Ar); 129.2 (2C Ar); 130.3; 132.0; 157.0 (t, ^2^*J*_CF_ = 27.2 Hz, C-2); 163.9; 178.6 (CO). HRMS (ESI) *m/z* [M + H]^+^. Calcd for C_12_H_9_F_2_O_2_: 223.0571. Found: 223.0574.

*2-Phenyl-6-(1,1,2,2-tetrafluoroethyl)-4H-pyran-4-one* (**5k**). Yield 44% (120 mg), yellow powder, mp 125–126 °C. IR (ATR) ν 3074, 2924, 1663, 1622, 1452, 1239, 1104, 940. ^1^H NMR (500 MHz, CDCl_3_) *δ* 6.10 (1H, tt, ^2^*J* = 53.1 Hz, ^3^*J* = 2.7 Hz, CHF_2_); 6.77 (1H, d, ^4^*J* = 1.8 Hz, CH); 6.84 (1H, d, ^4^*J* = 1.8 Hz, CH); 7.50–7.60 (3H, m, H Ph); 7.76 (2H, dd, ^3^*J* = 7.8 Hz, ^4^*J* = 1.4 Hz, H-2, H-6 Ph). ^19^F NMR (471 MHz, CDCl_3_) *δ* 26.9 (dt, ^2^*J*_HF_ = 53.0 Hz, ^3^*J*_FF_ = 4.0 Hz, CF_2_); 41.1 (td, ^3^*J*_FF_ = 4.0 Hz, ^3^*J*_HF_ = 2.7 Hz, CF_2_H). HRMS (ESI) *m/z* [M + H]^+^. Calcd for C_13_H_9_F_4_O_2_: 273.0539. Found: 273.0542.

*2-(1,1,2,2,3,3,4,4-Octafluorobutyl)-6-phenyl-4H-pyran-4-one* (**5l**). Yield 70% (261 mg), pale-yellow semi-solid liquid. IR (ATR) ν 3047, 1660, 1629, 1602, 1497, 1404, 1118, 944, 767. ^1^H NMR (500 MHz, CDCl_3_) *δ* 6.08 (1H, tt, ^2^*J* = 51.8 Hz, ^3^*J* = 5.1 Hz, CHF_2_); 6.83 (1H, d, ^4^*J* = 2.0 Hz, CH); 6.88 (1H, d, ^4^*J* = 2.0 Hz, CH); 7.51 (2H, dd, ^3^*J* = 7.9 Hz, ^3^*J* = 7.3 Hz, H-3, H-5 Ph); 7.57 (1H, tt, ^3^*J* = 7.3 Hz, ^4^*J* = 1.3 Hz, H-4 Ph); 7.75 (2H, dd, ^3^*J* = 7.9 Hz, ^4^*J* = 1.3 Hz, H-2, H-6 Ph). ^19^F NMR (376 MHz, CDCl_3_) *δ* 24.8 (dm, ^2^*J*_HF_ = 51.8 Hz, CF_2_H); 32.5–32.7 (m, 3-CF_2_); 37.75–37.85 (m, 2-CF_2_); 43.8 (tt, ^3^*J*_FF_ = 11.4 Hz, ^3^*J*_FF_ = 1.7 Hz, 1-CF_2_). HRMS (ESI) *m/z* [M + H]^+^. Calcd for C_15_H_9_F_8_O_2_: 373.0475. Found: 373.0479.

### 3.6. Synthesis of Pyrones ***6***

General procedure (Method C). A solution of Br_2_ (400 mg, 2.5 mmol) in CH_2_Cl_2_ (4.25 mL) was added to enedione 2 (1 mmol) in CH_2_Cl_2_ (2.7 mL) under cooling on an ice-bath. After that, the reaction mixture was stirred at room temperature for 15 h, and then the solvent was evaporated without heating. The residue was refluxed in pyridine (3 mL) at 100 °C for 10 h. The excess of pyridine was evaporated under reduced pressure and the product was purified by flash-chromatography (EtOAc). The product was hot-extracted with hexane (3 × 5 mL). The solvent was evaporated until 2 mL of hexane, and the product crystallized from the extract after cooling.

*3-Bromo-6-phenyl-2-(trifluoromethyl)-4H-pyran-4-one* (**6a**). Yield 73 mg (23%), beige solid, mp 132–133 °C. IR (ATR) ν 3069, 1646, 1600, 1451, 1146, 973, 769. ^1^H NMR (500 MHz, CDCl_3_) *δ* 6.94 (1H, s, H-5); 7.50–7.57 (2H, m, H-3, H-5 Ph); 7.57–7.62 (1H, m, H-4 Ph); 7.79 (2H, d, ^3^*J* = 7.4 Hz, H-2, H-6 Ph). ^19^F NMR (471 MHz, CDCl_3_) *δ* 95.6 (s, CF_3_). ^13^C NMR (126 MHz, CDCl_3_) *δ* 109.3; 116.3; 118.8 (q, ^1^*J*_CF_ = 276.4 Hz, CF_3_); 126.0 (2C Ph); 129.3; 129.4 (2C Ph); 132.6; 149.7 (q, ^2^*J*_CF_ = 38.0 Hz, C-6); 163.2; 172.8 (CO). Anal. Calcd for C_12_H_6_BrF_3_O_2_: C 45.17; H 1.90. Found: C 45.36; H 1.86%.

*3-Bromo-6-(4-fluorophenyl)-2-(trifluoromethyl)-4H-pyran-4-one* (**6b**). Yield 94 mg (28%), beige solid, mp 156–167 °C. IR (ATR) ν 1667, 1627, 1556, 1412. ^1^H NMR (500 MHz, CDCl_3_) *δ* 6.88 (1H, s, H-5); 7.20–7.28 (2H, m, H-3, H-5 Ar); 7.77–7.85 (2H, m, H-2, H-6 Ar). ^19^F NMR (471 MHz, CDCl_3_) *δ* 56.4 (tt, ^3^*J*_FH_ = 8.2 Hz, ^4^*J*_FH_ = 5.2 Hz, F-Ar); 95.6 (s, CF_3_). ^13^C NMR (126 MHz, CDCl_3_) *δ* 109.1 (d, ^6^*J*_CF_ = 1.1 Hz, H-5); 116.4 (q, ^3^*J*_CF_ = 1.1 Hz, C-3); 116.8 (d, ^2^*J*_CF_ = 22.3 Hz, C-3, C-5 Ar); 118.7 (q, ^1^*J*_CF_ = 276.6 Hz, CF_3_); 125.5 (d, ^4^*J*_CF_ = 3.4 Hz, C-1 Ar); 128.4 (d, ^3^*J*_CF_ = 9.1 Hz, C-2, C-6 Ar); 149.6 (q, ^2^*J*_CF_ = 39.6 Hz, C-2); 162.2; 162.3 (d, ^1^*J*_CF_ = 255.4 Hz, C-4 Ar); 172.7 (CO). Anal. Calcd for C_12_H_5_BrF_4_O_2_: C 42.76; H 1.50. Found: C 42.39; H 1.33%.

*3-Bromo-6-(4-chlorophenyl)-2-(trifluoromethyl)-4H-pyran-4-one* (**6c**). Yield 120 mg (34%), beige solid, mp 149–150 °C. *Procedure* from tribromide **3c**: Tribromide **3c** (533 mg, 1.0 mmol) was refluxed in pyridine (3 mL) at 100 °C for 10 h. The excess of pyridine was evaporated under reduced pressure, and the product purified by flash-chromatography (EtOAc). The product was hot-extracted with hexane (3×5 mL). The solvent was evaporated until 2 mL of hexane, and the product crystallized from extract after cooling. Yield 36% (127 mg), beige solid, mp 149–150 °C. IR (ATR) ν 3069, 1645, 1600, 1577, 1561, 1498, 1451. ^1^H NMR (400 MHz, CDCl_3_) *δ* 6.91 (1H, s, H-5); 7.52 (2H, d, ^3^*J* = 8.7 Hz, H Ar); 7.72 (2H, d, ^3^*J* = 8.7 Hz, H Ar). ^19^F NMR (376 MHz, CDCl_3_) *δ* 95.6 (s, CF_3_). ^13^C NMR (126 MHz, CDCl_3_) *δ* 109.4 (C-5); 116.5 (C-3); 118.7 (q, ^1^*J*_CF_ = 276.5 Hz, CF_3_); 127.3 (2C Ar); 127.7 (C Ar); 129.8 (2C Ar); 139.1; 149.7 (q, ^2^*J*_CF_ = 38.1 Hz, C-2); 162.1; 172.7 (CO). HRMS (ESI) *m/z* [M + H]^+^. Calcd for C_12_H_6_BrClF_3_O_2_: 352.9186. Found: 352.9183.

*3-Bromo-6-(3-nitrophenyl)-2-(trifluoromethyl)-4H-pyran-4-one* (**6e**). Yield 15 mg (4%), yellowish solid, mp 122–125 °C. IR (ATR) ν 3074, 3055, 1672, 1647, 1615, 1596, 1532, 1442. ^1^H NMR (500 MHz, DMSO-*d*_6_) *δ* 7.58 (1H, s, H-5); 7.90 (1H, t, ^3^*J* = 8.1 Hz, H-5 Ar); 8.39 (1H, ddd, ^3^*J* = 7.9 Hz, ^4^*J* = 1.7 Hz, ^4^*J* = 0.9 Hz, H-6 Ar); 8.46 (1H, ddd, ^3^*J* = 8.2 Hz, ^4^*J* = 2.2 Hz, ^4^*J* = 0.8 Hz, H-4 Ar); 8.68 (1H, dd, ^4^*J* = 2.2 Hz, ^4^*J* = 1.7 Hz, H-2 Ar). ^1^H NMR (400 MHz, CDCl_3_) *δ* 7.04 (1H, s, H-5); 7.78 (1H, t, ^3^*J* = 8.8 Hz, H-5 Ar); 8.12 (1H, ddd, ^3^*J* = 7.9 Hz, ^4^*J* = 1.7 Hz, ^4^*J* = 1.0 Hz, H-6 Ar); 8.45 (1H, ddd, ^3^*J* = 8.3 Hz, ^4^*J* = 2.2 Hz, ^4^*J* = 1.0 Hz, H-4 Ar); 8.64 (1H, dd, ^4^*J* = 2.2 Hz, ^4^*J* = 1.7 Hz, H-2 Ar). ^19^F NMR (376 MHz, CDCl_3_) *δ* 95.6 (s, CF_3_). ^13^C NMR (126 MHz, DMSO-*d*_6_) *δ* 110.9; 115.8; 118.6 (q, ^1^*J*_CF_ = 277.0 Hz, CF_3_); 120.8; 126.5; 131.0; 131.2; 132.4; 148.3; 148.9 (q, ^2^*J*_CF_ = 36.8 Hz, C-2); 160.2; 172.5 (CO). HRMS (ESI) *m/z* [M + H]^+^. Calcd for C_12_H_6_BrF_3_NO_4_: 363.9427. Found: 363.9429.

*3-Bromo-6-(p-tolyl)-2-(trifluoromethyl)-4H-pyran-4-one* (**6f**). Yield 107 mg (32%), beige solid, mp 170–171 °C. IR (ATR) ν 1644, 1608, 1595, 1569, 1511, 1449. ^1^H NMR (500 MHz, CDCl_3_) *δ* 2.44 (3H, s, Me); 6.90 (1H, s, H-5); 7.33 (2H, d, *J* = 8.1 Hz, H-3, H-5 Ar); 7.67 (2H, d, *J* = 8.1 Hz, H-2, H-6 Ar). ^19^F NMR (471 MHz, CDCl_3_) *δ* 95.6 (s, CF_3_). ^13^C NMR (126 MHz, CDCl_3_) *δ* 21.5 (CH_3_); 111.6 (C-3); 114.6 (q, ^3^*J*_CF_ = 2.5 Hz, C-5); 118.5 (q, ^1^*J*_CF_ = 273.8 Hz, CF_3_); 125.9 (2C Ar); 127.0 (C Ar); 130.0 (2C Ar); 143.1(C Ar); 152.3 (q, ^2^*J*_CF_ = 39.5 Hz, C-6); 164.1 (C-2); 178.0 (CO). HRMS (ESI) *m/z* [M + H]^+^. Calcd for C_13_H_9_BrF_3_O_2_: 332.9733. Found: 332.9737.

*3-Bromo-2-(difluoromethyl)-6-phenyl-4H-pyran-4-one* (**6j**). Yield 84 mg (28%), beige solid, mp 122–123 °C. ^1^H NMR (500 MHz, CDCl_3_) *δ* 6.93 (1H, s, H-5); 7.00 (1H, t, ^2^*J*_HF_ = 52.1 Hz, CHF_2_); 7.50–7.61 (3H, m, H Ph); 7.80–7.84 (2H, m, H-2, H-6 Ph). ^19^F NMR (376 MHz, CDCl_3_) *δ* 39.3 (d, ^2^*J*_HF_ = 52.1 Hz, CF_2_H). ^13^C NMR (126 MHz, CDCl_3_) *δ* 109.3 (t, ^1^*J*_CF_ = 242.3 Hz, CF_2_H); 109.5; 116.5 (t, ^3^*J*_CF_ = 5.3 Hz, C-3); 126.1 (2C Ph); 129.3 (2C Ph); 129.6; 132.4; 152.9 (t, ^2^*J*_CF_ = 23.9 Hz, C-2); 163.6; 172.9 (CO). HRMS (ESI) m/z [M + H]^+^. Calcd for C_13_H_10_F_3_O_2_: 255.0633. Found: 255.0629.

*3-Bromo-6-phenyl-2-(1,1,2,2-tetrafluoroethyl)-4H-pyran-4-one* (**6k**). Yield 77 mg (24%), beige solid, mp 122–125 °C. IR (ATR) ν 3074, 3002, 1642, 1452, 1383, 1231, 1213, 1083, 975, 842. ^1^H NMR (500 MHz, CDCl_3_) *δ* 6.32 (1H, tt, ^2^*J*_HF_ = 52.8 Hz, *J* = 4.0 Hz, CHF_2_); 6.95 (1H, s, H-5); 7.51–7.56 (2H, m, H-3, H-5 Ph); 7.57–7.61 (1H, m, H-4 Ph); 7.75–7.79 (2H, m, H-2, H-6 Ph). ^19^F NMR (471 MHz, CDCl_3_) *δ* 26.3 (dt, ^3^*J*_FF_ = 6.6 Hz, ^3^*J*_HF_ = 4.1 Hz, CF_2_); 44.6 (td, ^2^*J*_FF_ = 52.8 Hz, ^3^*J*_FF_ = 6.6 Hz, CF_2_H). ^13^C NMR (126 MHz, CDCl_3_) *δ* 108.9 (tt, ^1^*J*_CF_ = 254.1 Hz, ^2^*J*_CF_ = 35.8 Hz, CF_2_H); 109.2; 111.4 (tt, ^1^*J*_CF_ = 256.9 Hz, ^2^*J*_CF_ = 28.9 Hz, CF_2_); 117.6; 126.0 (2C Ph); 129.4 (2C Ph); 130.5; 132.5; 150.9 (t, ^2^*J*_CF_ = 27.4 Hz, C-2); 163.6; 172.8 (CO). HRMS (ESI) m/z [M + H]^+^. Calcd for C_13_H_7_BrF_4_O_2_: 350.9644. Found: 350.9650.

*3-Bromo-2-(1,1,2,2,3,3,4,4-octafluorobutyl)-6-phenyl-4H-pyran-4-one* (**6l**). Yield 149 mg (33%), beige solid, mp 86–88 °C. IR (ATR) ν 3075, 1640, 1499, 1453, 1377, 1145, 943. ^1^H NMR (400 MHz, CDCl_3_) *δ* 6.12 (3H, tt, ^2^*J*_HF_ = 51.9 Hz, ^3^*J*_HF_ = 5.2 Hz, CHF_2_); 6.95 (1H, s, H-5); 7.51–7.62 (3H, m, H Ph); 7.74–7.78 (2H, m, H-2, H-6 Ph). ^19^F NMR (376 MHz, CDCl_3_) *δ* 24.9 (dm, ^2^*J*_HF_ = 51.9 Hz, CF_2_H); 32.4 –32.6 (m, 3-CF_2_); 38.7–38.8 (m, 2-CF_2_); 49.3 (t, ^3^*J*_FF_ = 11.6 Hz, 1-CF_2_). HRMS (ESI) m/z [M + H]^+^. Calcd for C_15_H_7_BrF_8_O_2_: 450.9580. Found: 450.9590.

### 3.7. Synthesis of Fluorinated Azaheterocycles ***7**–**9***

*2-Phenyl-6-(trifluoromethyl)pyridin-4(1H)-one* (**7**). A solution of pyrone **5a** (120 mg, 0.5 mmol) in EtOH (2 mL), saturated with NH_3_ at room temperature, was heated in an autoclave at 100 °C for 8 h. The reaction mixture was diluted with H_2_O (10 mL), and the formed precipitate was filtered and recrystallized from EtOH. Yield 84 mg (70%), colorless crystals, mp 133–134 °C. IR (ATR) ν 1614, 1579, 1481, 1427, 1460, 1429. ^1^H NMR (400 MHz, DMSO-*d*_6_) *δ* pyridinol form (96%): 7.05 (1H, s, H-3); 7.25 (1H, s, H-5); 7.39–7.48 (3H, m, H Ph); 7.91 (2H, d, *J* = 6.8 Hz, H-2, H-6 Ph); pyridone form (4%): 6.87 (1H, d, ^4^*J* = 2.0 Hz, H-3); 6.84 (1H, d, ^4^*J* = 2.0 Hz, H-5); 7.39–7.48 (3H, m, H Ph); 7.87–7.94 (2H, m, H-2, H-6 Ph); the signals from NH and OH were not observed due to the fast exchange with water. ^19^F NMR (376 MHz, CDCl_3_) *δ* pyridone form (4%): 93.4 (s, CF_3_); pyridinol form (96%): 93.6 (s, CF_3_). ^13^C NMR (126 MHz, CDCl_3_) *δ* 108.3 Hz, 111.2 Hz, 121.2 (q, ^1^*J*_CF_ = 274.4 Hz, CF_3_); 127.1 (2C Ph); 128.9 (2C Ph); 130.0 Hz, 137.2 Hz, 148.1 Hz, 158.9 Hz, 166.5. Anal. Calcd for C_12_H_8_F_3_NO: C 60.26; H 3.37; N 5.86. Found: C 60.35; H 3.26; N 5.84%.

*1-Phenyl-3-(4-(trifluoromethyl)-1H-1,2,3-triazol-5-yl)propane-1,3-dione* (**8**). To a solution of pyrone **5a** (96 mg, 0.4 mmol) in DMSO (1 mL), NaN_3_ (29 mg, 0.44 mmol) was added, and the mixture was stirred at 115–120 °C for 30 min. The reaction was then diluted with aqueous HCl (10 mL, 0.1 M), and the formed precipitate was filtered and recrystallized from toluene. Yield 65 mg (55%), pale-brown plates, mp 189–190 °C. IR (ATR) ν 3268 (NH), 1600, 1575, 1535, 1492, 1460, 1429. ^1^H NMR (400 MHz, DMSO-*d*_6_) *δ* enol form (82%): 7.19 (1H, s, 2-CH); 7.59 (2H, t, *J* = 7.6 Hz, H-3, H-5 Ph); 7.68 (1H, t, *J* = 7.4 Hz, H-4 Ph); 8.05 (2H, d, *J* = 7.4 Hz, H-2, H-6 Ph); 14.70–17.9 (2H, broad s, NH, OH); keto form (18%): 4.85 (2H, s, CH_2_); 7.58 (2H, t, *J* = 7.4 Hz, H-3, H-5 Ph); 7.70 (1H, t, *J* = 7.4 Hz, H-4 Ph); 8.01 (2H, d, *J* = 7.4 Hz, H-2, H-6 Ph); 14.70–17.9 (1H, broad s, NH). ^19^F NMR (376 MHz, DMSO-*d*_6_) *δ* keto form (18%): 102.2 (s, CF_3_); enol form (82%): 103.2 (s, CF_3_). ^13^C NMR (126 MHz, DMSO-*d*_6_) *δ* enol form: 94.9 (CH=); 120.7 (q, ^1^*J*_CF_ = 268.5 Hz, CF_3_); 127.2 (2C Ar); 129.1 (2C Ar); 133.2 (C Ar); 133.2 (C Ar); 136.2 (q, ^2^*J*_CF_ = 40.1 Hz, C-CF_3_); 140.9 (C Ar); 179.6; 182.2; keto form: 50.7 (CH_2_); 120.2 (q, ^1^*J*_CF_ = 267.1 Hz, CF_3_); 128.5 (2C Ar); 128.9 (2C Ar); 133.9 (C Ar); 136.0 (C Ar); 136.2 (q, ^2^*J*_CF_ = 40.5 Hz, C-CF_3_); 142.4 (C Ar); 187.9 (CO); 194.8 (CO). Anal. Calcd for C_12_H_8_F_3_N_3_O_2_: C 50.89; H 2.85; N 14.84. Found: C 50.90; H 2.78; N 14.85%.

*(E)-1,5-Diphenyl-3-(3,3,3-trifluoro-2-(2-phenylhydrazono)propyl)-1H-pyrazole* (**9**). A mixture of pyrone **5a** (120 mg, 0.5 mmol) and phenylhydrazine (270 mg, 2.5 mmol) was heated at 120 °C for 3 h. The reaction mixture was cooled to room temperature, treated with 1M HCl solution (10 mL), the formed precipitate was filtered and recrystallized from EtOH. Yield 65 mg (55%), colorless crystals, mp 171–173 °C. IR (ATR) ν 3267 (NH), 1605 (C=N), 1543, 1500, 1457. ^1^H NMR (500 MHz, CDCl_3_) *δ* 3.84 (2H, s, H-3); 6.40 (1H, s, H-5); 6.91 (1H, t, *J* = 7.3 Hz, H-4 Ph); 7.11 (2H, d, *J* = 7.8 Hz, H-2, H-6 Ph); 7.19 (2H, dd, ^3^*J* = 7.7 Hz, ^4^*J* = 1.5 Hz, H-2, H-6 Ph); 7.22–7.38 (3H, m, H Ph); 9.99 (1H, s, NH). ^19^F NMR (376 MHz, CDCl_3_) *δ* 92.8 (s, CF_3_). ^13^C NMR (126 MHz, CDCl_3_) *δ* 24.6; 107.2; 113.5 (2C); 121.4; 121.94 (q, ^1^*J*_CF_ = 272.0 Hz, CF_3_); 125.1 (2C); 127.8; 128.5 (2C); 128.6; 128.7 (2C); 128.78 (q, ^2^*J*_CF_ = 33.9 Hz, C=N); 129.0 (2C); 129.2 (2C); 129.8; 139.6; 144.1; 144.8; 147.1. Anal. Calcd for C_24_H_19_F_3_N_4_: C 68.56; H 4.56; N 13.33. Found: C 68.42; H 4.38; N 13.34%.

## Figures and Tables

**Table 1:** The scope of prepared enediones **2**.

Enedione	Ar	R^F^	Yield, %	Mp, °C
**2a**	Ph	CF_3_	75	56–58
**2b**	4-FC_6_H_4_	CF_3_	64	83–84
**2c**	4-ClC_6_H_4_	CF_3_	60	92–93
**2d**	4-BrC_6_H_4_	CF_3_	92	86–87
**2e**	3-O_2_NC_6_H_4_	CF_3_	51 ^a^	119–120
**2f**	4-MeC_6_H_4_	CF_3_	65	61–62
**2g**	4-MeOC_6_H_4_	CF_3_	81	104–105
**2h**	4-Me_2_NC_6_H_4_	CF_3_	67	79–80
**2i**	2-C_4_H_3_S	CF_3_	84	94–95
**2j**	Ph	CF_2_H	43	85–87
**2k**	Ph	H(CF_2_)_2_	43	Liq.
**2l**	Ph	H(CF_2_)_4_	51	Liq.

^a^ Lithium hydride in benzene under reflux.

**Table 2:** Selectivity of bromination of enediones **2** ^a^.

№	Ar	Enedione	Br_2_ Equiv.	Solvent	2:3:4 Ratio ^b^
1	4-FC_6_H_4_	****2b****	1.0	AcOH ^e^	74:(7 + 2 ^c^):(16 + 1 ^c^)
2	4-FC_6_H_4_	**2b**	1.0	CH_2_Cl_2_	42:(37 + 2 ^c^):19
3	4-FC_6_H_4_	**2b**	2.5	CH_2_Cl_2_	0:7 ^c^:93
4	4-FC_6_H_4_	**2b**	1.0	*t*-BuOMe	17:(31 + 4 ^c^):(39 + 9 ^c^)
5	4-FC_6_H_4_	**2b**	1.0	dioxane	29:65:6
6	4-FC_6_H_4_	**2b**	1.0	CS_2_	25:(65 + 2 ^c^):(7 + 1 ^c^)
7	4-FC_6_H_4_	**2b**	1.0	C_6_H_6_	12:78:10
8	4-FC_6_H_4_	**2b**	1.2	C_6_H_6_	0:(75 + 4 ^c^):(19 + 2 ^c^)
9	4-FC_6_H_4_	**2b**	2.2	C_6_H_6_	0:(46 + 10 ^c^):(33 + 11 ^c^)
10	4-MeOC_6_H_4_	****2g****	1.0	C_6_H_6_	3:75:22
11	2-C_4_H_3_S	**2i**	1.0	C_6_H_6_	9:(80 + 8 ^d^):3
12	4-ClC_6_H_4_	**2c**	1.0	C_6_H_6_	62:(10 + 3 ^c^):25
13	3-O_2_NC_6_H_4_	**2e**	1.0	C_6_H_6_	55:(14 + 2 ^c^):(26 + 3 ^c^)
14	Ph	**2a**	1.0	C_6_H_6_	62:24:14
15	Ph	**2a**	1.0	CH_2_Cl_2_	0:(86 + 2 ^c^):12

^a^ A solution of Br_2_ was added to enedione **2** under cooling on an ice-bath. The reaction mixture was stirred at room temperature for 15 h. ^b^ Based on the ^19^F and ^1^H NMR spectra. ^c^ Cyclized to dihydropyrone. ^d^ A product of dehydrobromination was spontaneously formed. ^e^ Br_2_ was added at room temperature.

**Table 3:** The scope of 4-pyrone synthesis via the *one-pot* oxidative cyclization of enediones **2**.

Enedione	Ar	Pyrone	Method	Yield, %
**2a**	Ph	**5a**	B	48
**2b**	4-FC_6_H_4_	**5b**	A	69
**2c**	4-ClC_6_H_4_	**5c**	B	62
**2d**	4-BrC_6_H_4_	**5d**	B	65
**2f**	4-MeC_6_H_4_	**5f**	B	28
**2g**	4-MeOC_6_H_4_	**5g**	A	65
**2i**	2-C_4_H_3_S	**5i**	A	62
**2a**	Ph	**6a**	C	23
**2b**	4-FC_6_H_4_	**6b**	C	28
**2c**	4-ClC_6_H_4_	**6c**	C	34
**2e**	3-O_2_NC_6_H_4_	**6e**	C	4
**2f**	4-MeC_6_H_4_	**6f**	C	42

Method A: The mixture of enedione **2** (1.0 mmol) with bromine (1.0 mmol) in C_6_H_6_ was stirred at room temperature. Cyclization was carried out in the presence of Et_3_N (2.5 equiv.) in acetone. Method B: The mixture of enedione **2** (1.0 mmol) with bromine (1.0 mmol) was stirred in CH_2_Cl_2_ at room temperature. Cyclization was carried out in the presence of Et_3_N in acetone. Method C: The mixture of enedione 2 (1.0 mmol) with bromine (2.5 mmol) in CH_2_Cl_2_ was stirred at room temperature. Cyclization was carried in pyridine at 100 °C for 10 h.

## Data Availability

Data is contained within the article and Supplementary Materials.

## Supplementary Materials

The following are available online, Scheme S1. Reaction of (3Z,5E)-6-(4-(dimethylamino)phenyl)-1,1,1-trifluoro-4-hydroxyhexa-3,5-dien-2-one with Br2 in benzene; Table S1. Composition of reaction mixtures of bromination of enediones 2; Table S2. Characteristic chemical shifts in 1H and 19F NMR spectra of compounds 3; Table S3. Characteristic chemical shifts in 1H and 19F NMR spectra of compounds 4; Table S4. Characteristic chemical shifts in 1H and 19F NMR spectra of dihydropyrone forms of 3; Table S5. Characteristic chemical shifts in 1H and 19F NMR spectra of dihydropyrone forms of 4; full 1H, 19F, and 13C NMR spectra of all synthesized compounds.

## Sample Availability

Samples of the compounds are not available from the authors.
