# Supplementary material for: 2-Aryl-6-Polyfluoroalkyl-4-Pyrones as Promising RF-Building-Blocks: Synthesis and Application for Construction of Fluorinated Azaheterocycles
Source: Molecules. 2021 Jul 21;26(15):4415. doi: 10.3390/molecules26154415 (PMC8347980; doi:10.3390/molecules26154415)
Supplement: Supplementary file 1 [file molecules-26-04415-s001.zip › molecules-1297286-supplementary.pdf]

Supplementary Materials

2-Aryl-6-polyfluoroalkyl-4-pyrones as promising R^F-building-blocks: synthesis and application for construction of fluorinated azaheterocycles

Sergey A. Usachev [1], Diana I. Nigamatova [1], Daria K. Mysik [1], Nikita A. Naumov [1], Dmitrii L. Obydennov [1], and Vyacheslav Y. Sosnovskikh [1,*]

[1] Institute of Natural Sciences and Mathematics, Ural Federal University, 51 Lenina Ave., 620000 Ekaterinburg, Russian Federation

* Correspondence: vy.sosnovskikh@urfu.ru; Tel.: +7 (343) 3899597

Table of contents

Scheme S1. Reaction of (3*Z*,5*E*)-6-(4-(dimethylamino)phenyl)-1,1,1-trifluoro-4-hydroxyhexa-3,5-dien-2-one with Br_2 in benzene S2

Table S1. Composition of reaction mixtures of bromination of enediones **2** S3

Table S2. Characteristic chemical shifts in ^1H and ^{19}F NMR spectra of compounds **3** S4

Table S3. Characteristic chemical shifts in ^1H and ^{19}F NMR spectra of compounds **4** S5

Table S4. Characteristic chemical shifts in ^1H and ^{19}F NMR spectra of dihydropyrone forms of **3** S6

Table S5. Characteristic chemical shifts in ^1H and ^{19}F NMR spectra of dihydropyrone forms of **4** S7

^1H, ^{19}F, and ^{13}C NMR spectra of compounds **2–9** and **A′** S8-S93

Scheme S1. Reaction of (3*Z*,5*E*)-6-(4-(dimethylamino)phenyl)-1,1,1-trifluoro-4-hydroxyhexa-3,5-dien-2-one with Br_2 in benzene.

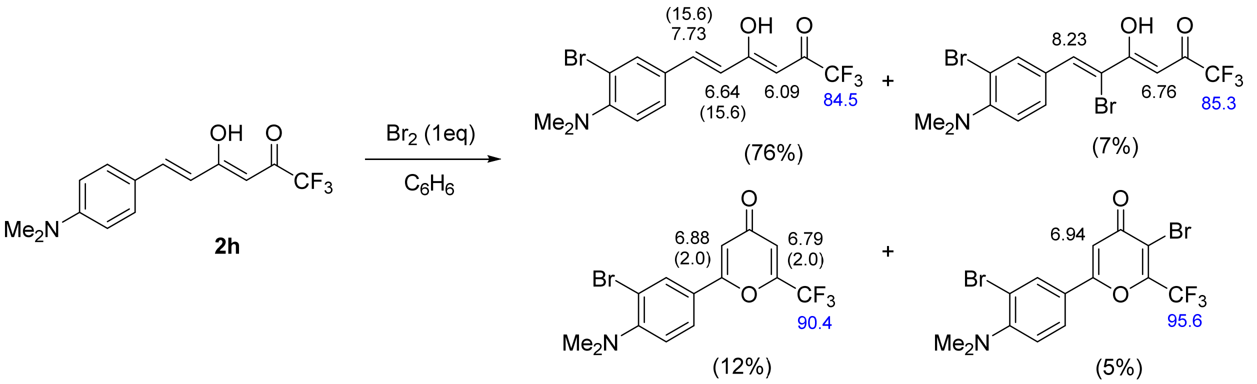

The presence of the strong donor and moderate basic substituent completely changed the composition of the reaction mixture in the case of dimethylaminophenyl derivative **2h**. Thus, when one equivalent of bromine was used in benzene, selective bromination of the aromatic fragment occurred with subsequent partial bromination at the double bond and methylene group followed by spontaneous dehydrobromination and ring closure to corresponding pyrones. The characteristic signals and coupling constants (in parentheses) in the ^{1}H (in black) and ^{19}F (in blue) NMR spectra of the reaction mixture after benzene evaporation are shown in the scheme. Complete bromination of the dimethylaminophenyl group follows from the absence of signals for aromatic protons with δ <7.5 ppm. The treatment of enedione **2h** with a threefold excess of bromine led to a complex mixture of products. The absence of signals in the ^{19}F NMR spectrum of the reaction mixture suggests that the reaction was accompanied by detrifluoroacetylation.

Table S1. Composition of reaction mixtures of bromination of enediones **2**.

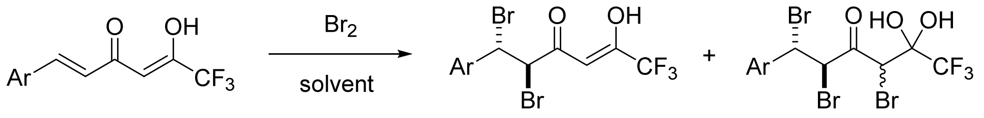

2 **3** **4**

Ar	**2,3,4**	Br_2, equiv.	Solvent	**2**:**3**:**4** ratio[a]
4-FC_6H_4	**b**	1.0	dioxane	29:65:6
4-FC_6H_4	**b**	1.3	dioxane	4:75:21
4-FC_6H_4	**b**	1.0	*t*-BuOMe	17:(31+4[b]):(39+9[b])
4-FC_6H_4	**b**	1.0	CS_2	25:(65+2[b]):(7+1[b])
4-FC_6H_4	**b**	1.0	AcOH	74:(7+2[b]):(16+1[b])
4-FC_6H_4	**b**	1.0	CH_2Cl_2	42:(37+2[b]):19
4-FC_6H_4	**b**	1.5	CH_2Cl_2	11:45:43
4-FC_6H_4	**b**	2.5	CH_2Cl_2	0:7[b]:93
4-FC_6H_4	**b**	1.0	benzene	12:77:11
4-FC_6H_4	**b**	1.2	benzene	0:(75+4[b]):(19+2[b])
4-FC_6H_4	**b**	2.2	benzene	0:(48+9[b]):(32+10[b])
Ph	**a**	1.0	CH_2Cl_2	0:(86+2[b]):12
Ph	**a**	1.0	benzene	55:28:17
Ph	**a**	2.2	benzene	0:(27+15[b]):(42+16[b])
4-ClC_6H_4	**c**	1.0	benzene	60:(9+2[b]):(27+2[b])
4-ClC_6H_4	**c**	2.2	benzene	0:(18+21[b]):(47+13[b])
4-ClC_6H_4	**c**	2.1	$CHCl_3$	0:5[b]:95
3-$NO_2C_6H_4$	**e**	1.0	benzene	51:(14+3[b]):(28+4[b])
3-$NO_2C_6H_4$	**e**	2.0	$CHCl_3$	0:(38+3[b]):(55+4[b])
4-MeC_6H_4	**f**	2.0	$CHCl_3$	0:(25+9[b]):(43+23[b])
4-$MeOC_6H_4$	**g**	1.0	benzene	3:75:22
4-$MeOC_6H_4$	**g**	2.0	$CHCl_3$	0:(21+15[b]):(43+22[b])
2-C_4H_3S	**i**	1.0	benzene	9:(80+8[c]):3
2-C_4H_3S	**i**	1.0	$CHCl_3$	16:(72+1[b]+6[c]):5
2-C_4H_3S	**i**	2.5	CH_2Cl_2	0:0:(52+48[d])

[a] Based on ^1H and ^{19}F NMR spectra
[b] Cyclized to dihydropyrone **B** or **C**
[c] A product of dehydrobromination **A** was spontaneously formed
[d] A product of aromatic bromination was also formed

Table S2. Characteristic chemical shifts in the ^{1}H and ^{19}F NMR spectra of compounds **3** used for the analysis of reaction mixtures, *δ*, ppm (*J*, Hz)

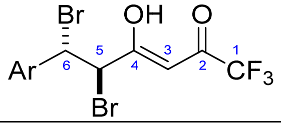

Ar	**3**	CF_3	6-CH	5-CH	3-CH
Ph	**a**	86.0	5.37 (11.6)	4.94 (11.6)	6.22
4-FC_6H_4	**b**	86.0	5.36 (11.6)	4.88 (11.6)	6.21
4-ClC_6H_4	**c**	86.0	5.34 (11.6)	4.88 (11.6)	6.20
4-BrC_6H_4	**d**	86.0	5.26 (11.6)	4.81 (11.6)	6.13
3-$O_2NC_6H_4$	**e**	86.0	5.45 (11.6)	4.94 (11.6)	6.23
4-MeC_6H_4	**f**	86.0	5.35 (11.6)	4.93 (11.6)	6.21
4-$MeOC_6H_4$	**g**	86.0	5.37 (11.6)	4.93 (11.6)	6.21
2-C_4H_3S	**i**	86.0	5.72 (11.4)	4.92 (11.4)	6.20

Table S3. Characteristic chemical shifts in the ^{1}H and ^{19}F NMR spectra of compounds **4** used for the analysis of reaction mixtures, *δ*, ppm (*J*, Hz)

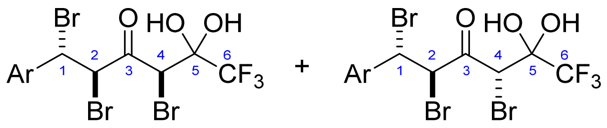

Ar	**4**	CF_3	1-CH	2-CH	4-CH	$C(OH)_2$
Ph *(major)*	**a**	–	5.39 (11.3)	5.30 (11.3)	4.96	4.46; 4.79
Ph *(minor)*	**a**	–	5.30 (11.3)	5.24 (11.3)	5.00	4.04; 4.63
4-FC_6H_4 *(major)*	**b**	79.7	5.38 (11.3)	5.24 (11.3)	4.95	4.48; 4.80
4-FC_6H_4 *(minor)*	**b**	79.9	5.29 (11.3)	5.19 (11.3)	4.99	4.03; 4.61
4-ClC_6H_4 *(major)*	**c**	79.7	5.35 (11.3)	5.24 (11.3)	4.95	4.45; 4.79
4-ClC_6H_4 (*minor*)	**c**	–	5.27 (11.3)	5.19 (11.3)	4.98	3.94; 4.58
3-$O_2NC_6H_4$ (*major*)	**e**	79.7	5.46 (11.3)	5.28 (11.3)	4.96	4.37; 4.75
3-$O_2NC_6H_4$ (*minor*)	**e**	79.8	5.38 (11.2)	5.24 (11.2)	4.99	3.96; 4.58
4-MeC_6H_4 (*major*)	**f**	79.7	5.37 (11.3)	5.29 (11.3)	4.96	4.43; 4.77
4-MeC_6H_4 (*minor*)	**f**	79.8	5.28 (11.3)	5.23 (11.3)	5.00	4.01; 4.61
4-$MeOC_6H_4$ (*major*)	**g**	79.7	5.40 (11.3)	5.29 (11.3)	4.96	4.48; 4.79
4-$MeOC_6H_4$ (*minor*)	**g**	79.9	5.30 (11.3)	5.22 (11.3)	5.00	4.07; 4.50
2-C_4H_3S (*major*)	**i**	79.7	5.73 (11.1)	5.26 (11.1)	4.94	4.44; 4.78
2-(5-BrC_4H_2S) (*major*)	**i-Br**	–	5.61 (11.1)	5.16 (11.1)	4.96	4.44; 4.78

Table S4. Characteristic chemical shifts in the ^{1}H and ^{19}F NMR spectra of dihydropyrone form of **3** used for the analysis of reaction mixtures, δ, ppm (J, Hz)

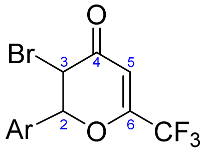

dihydropyrone form of **3**

Ar	dihydropyrone form of **3**	CF_3	2-CH	3-CH	5-CH
Ph	**a**	–	5.56 (11.3)	5.37 (11.3)	5.85
4-FC_6H_4	**b**	86.7	5.51 (11.3)	5.36 (11.3)	5.84
4-ClC_6H_4	**c**	–	5.51 (11.3)	5.34 (11.3)	5.84
3-$O_2NC_6H_4$	**e**	86.7	5.56 (11.6)	overlaped	5.85
4-MeC_6H_4	**f**	86.6	5.56 (11.3)	5.36 (11.3)	5.84
4-$MeOC_6H_4$	**g**	86.7	5.55 (11.3)	5.37 (11.3)	5.84

Table S5. Characteristic chemical shifts in the ^{1}H and ^{19}F NMR spectra of dihydropyrone form of **4** used for the analysis of reaction mixtures, δ, ppm (J, Hz)

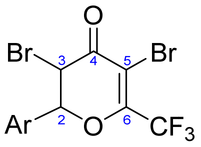

dihydropyrone form of **4**

Ar	dihydropyrone form of **4**	CF_3	2-CH	3-CH
Ph	**a**	–	5.80 (11.5)	5.44 (11.5)
4-FC_6H_4	**b**	90.2	5.74 (11.5)	5.43 (11.5)
4-ClC_6H_4	**c**	–	5.73 (11.5)	5.41 (11.5)
3-$O_2NC_6H_4$	**e**	90.2	5.75 (11.3)	5.51 (11.3)
4-MeC_6H_4	**f**	90.2	5.79 (11.5)	5.43 (11.6)
4-$MeOC_6H_4$	**g**	90.2	5.79 (11.4)	5.45 (11.4)

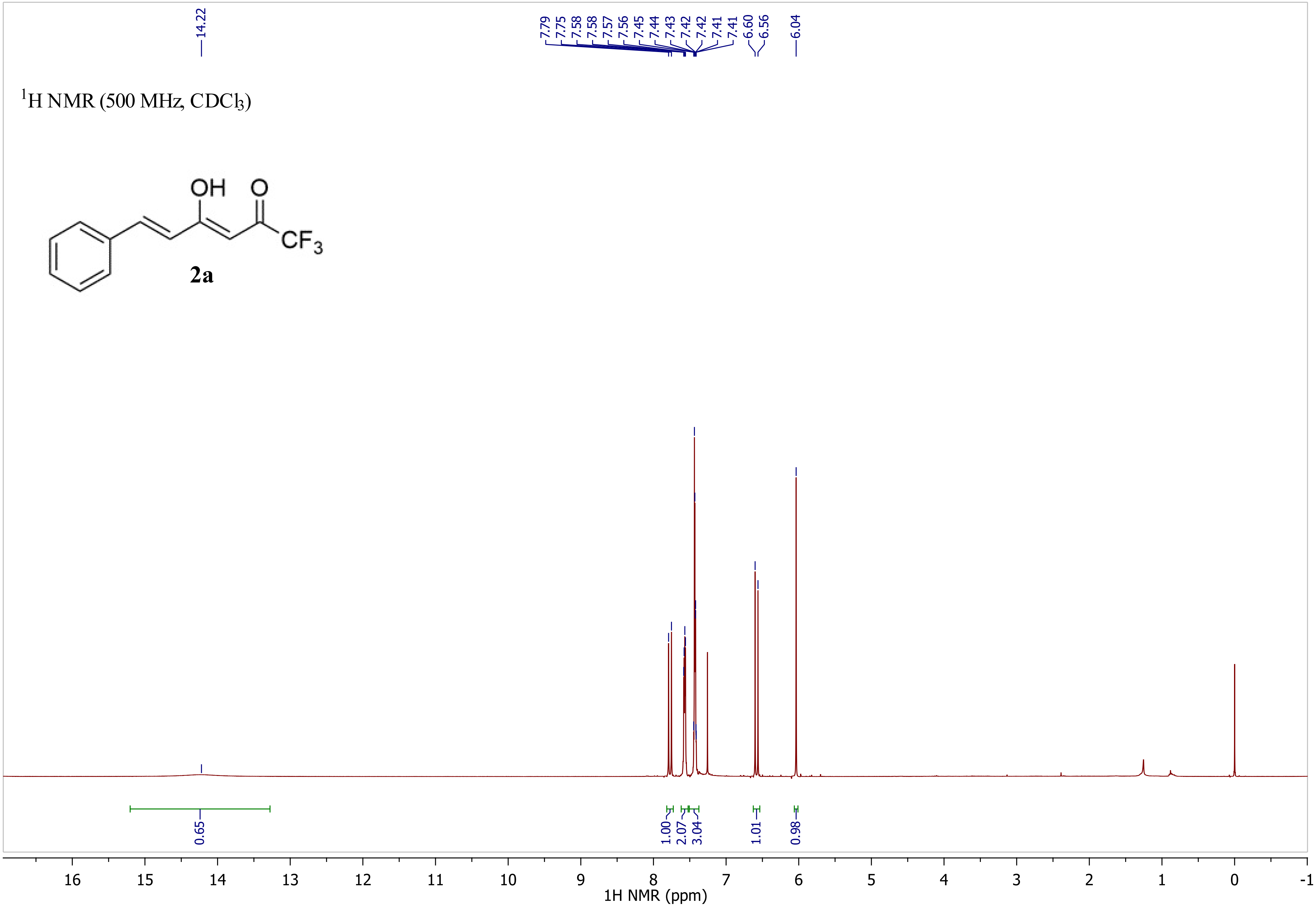

14.22
7.79
7.75
7.58
7.58
7.57
7.56
7.45
7.44
7.43
7.42
7.42
7.41
7.41
6.60
6.56
6.04
^{1}H NMR (500 MHz, $CDCl_3$)
OH
O
CF_3
2a
0.65
1.00
2.07
3.04
1.01
0.98
16
15
14
13
12
11
10
9
8
7
6
5
4
3
2
1
0
-1
1H NMR (ppm)

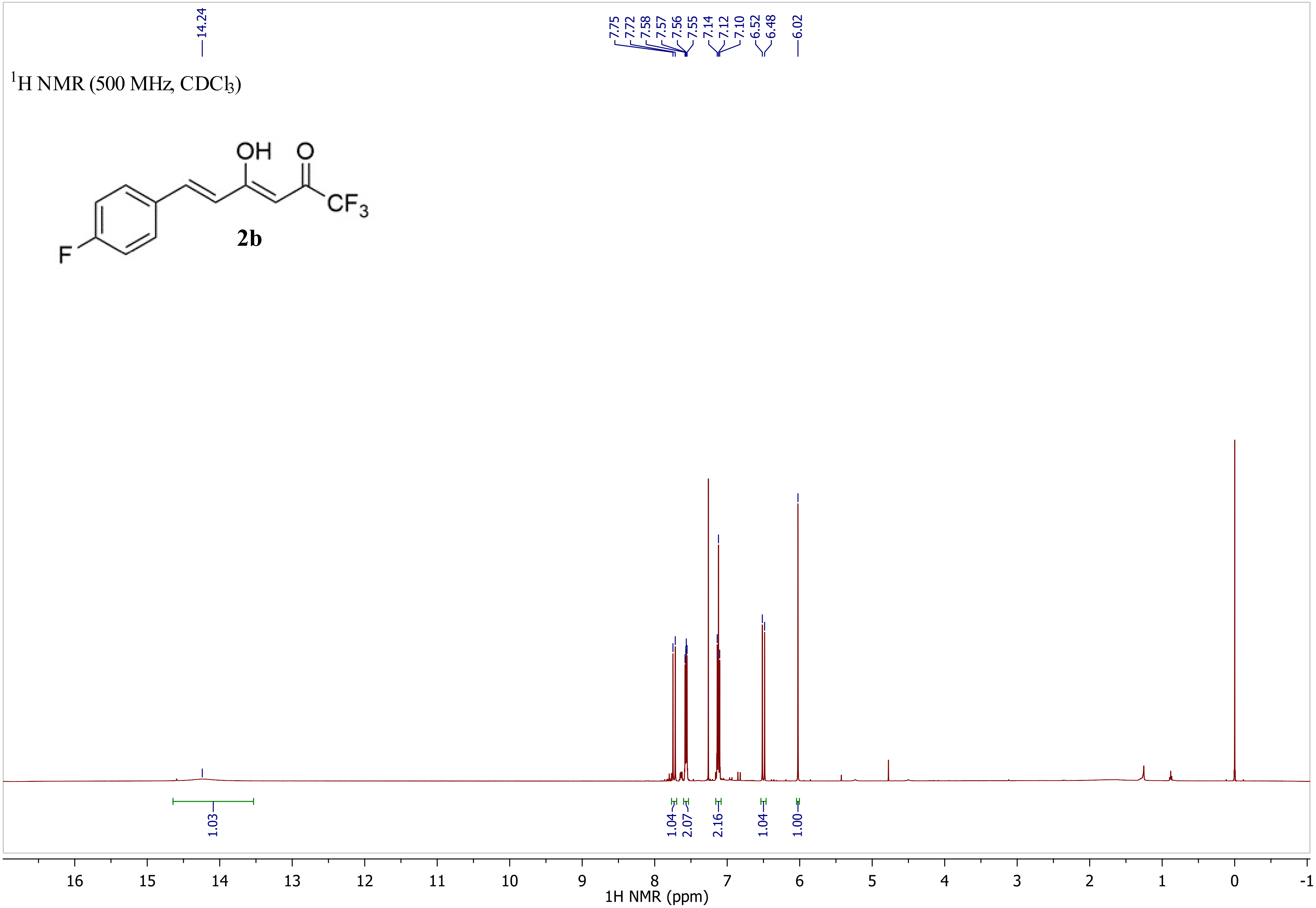

14.24
7.75
7.72
7.58
7.57
7.56
7.55
7.14
7.12
7.10
6.52
6.48
6.02
¹H NMR (500 MHz, CDCl3)
OH
O
CF3
F
2b
1.03
1.04
2.07
2.16
1.04
1.00
16
15
14
13
12
11
10
9
8
7
6
5
4
3
2
1
0
-1
1H NMR (ppm)

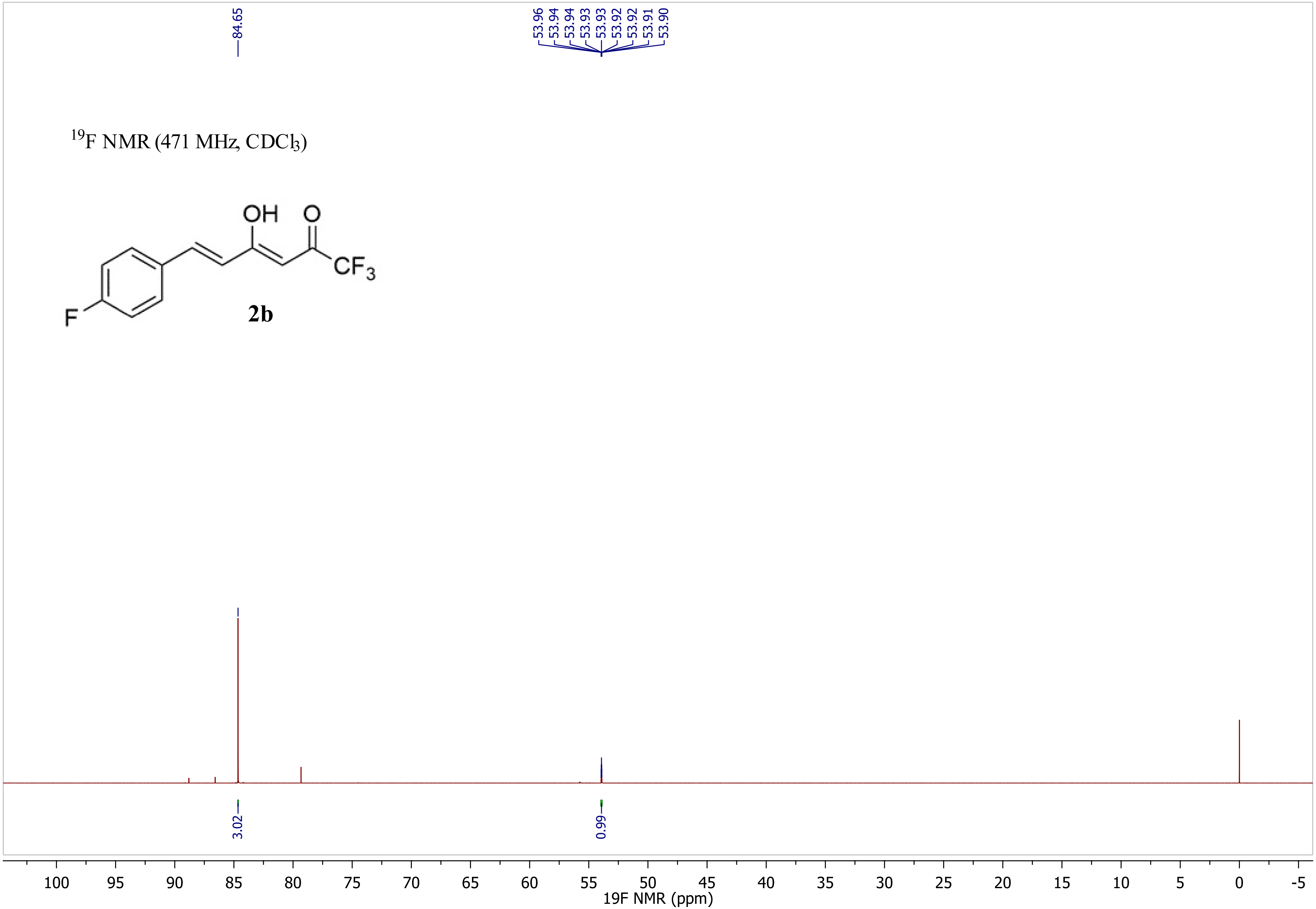

84.65
53.96
53.94
53.94
53.93
53.93
53.92
53.92
53.91
53.90
^{19}F NMR (471 MHz, $CDCl_3$)
OH
O
CF3
F
2b
3.02
0.99
100 95 90 85 80 75 70 65 60 55 50 45 40 35 30 25 20 15 10 5 0 -5
19F NMR (ppm)

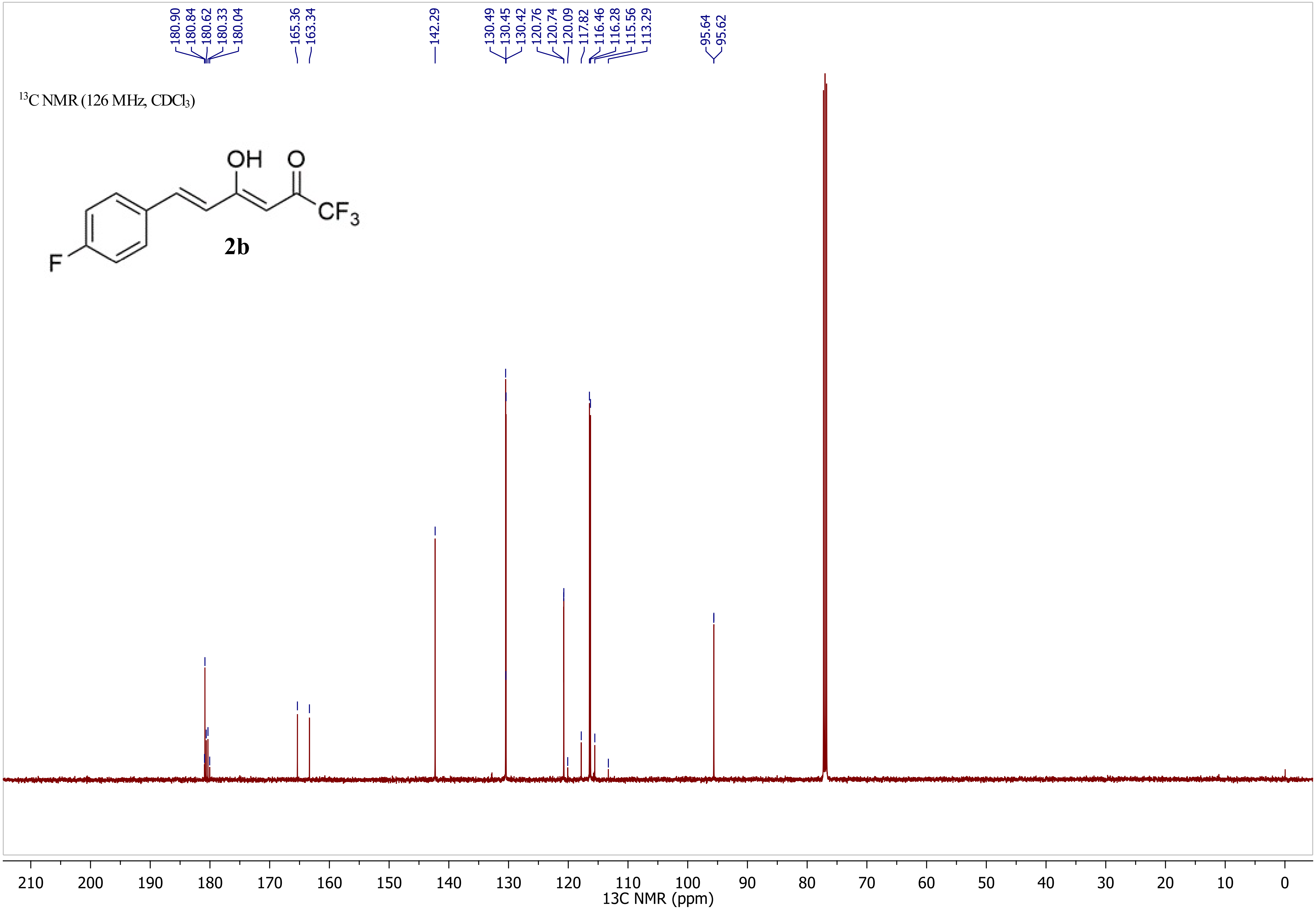
180.90
180.84
180.62
180.33
180.04
165.36
163.34
142.29
130.49
130.45
130.42
120.76
120.74
120.09
117.82
116.46
116.28
115.56
113.29
95.64
95.62
^{13}C NMR (126 MHz, $CDCl_3$)
OH
O
F
CF_3
2b
210 200 190 180 170 160 150 140 130 120 110 100 90 80 70 60 50 40 30 20 10 0
13C NMR (ppm)

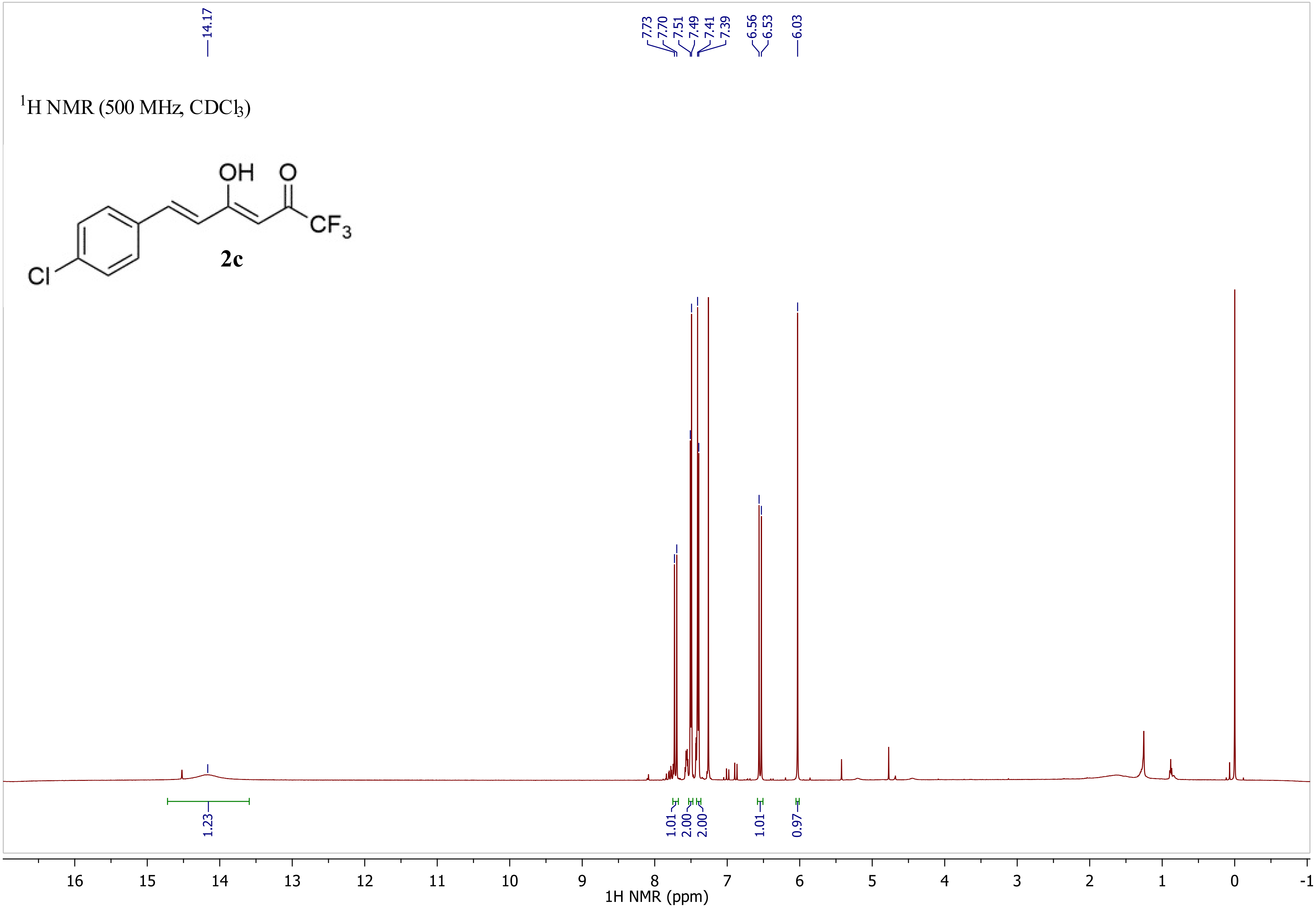
1H NMR (500 MHz, CDCl3)
OH
O
CF3
Cl
2c
14.17
7.73
7.70
7.51
7.49
7.41
7.39
6.56
6.53
6.03
1.23
1.01
2.00
2.00
1.01
0.97
16 15 14 13 12 11 10 9 8 7 6 5 4 3 2 1 0 -1
1H NMR (ppm)

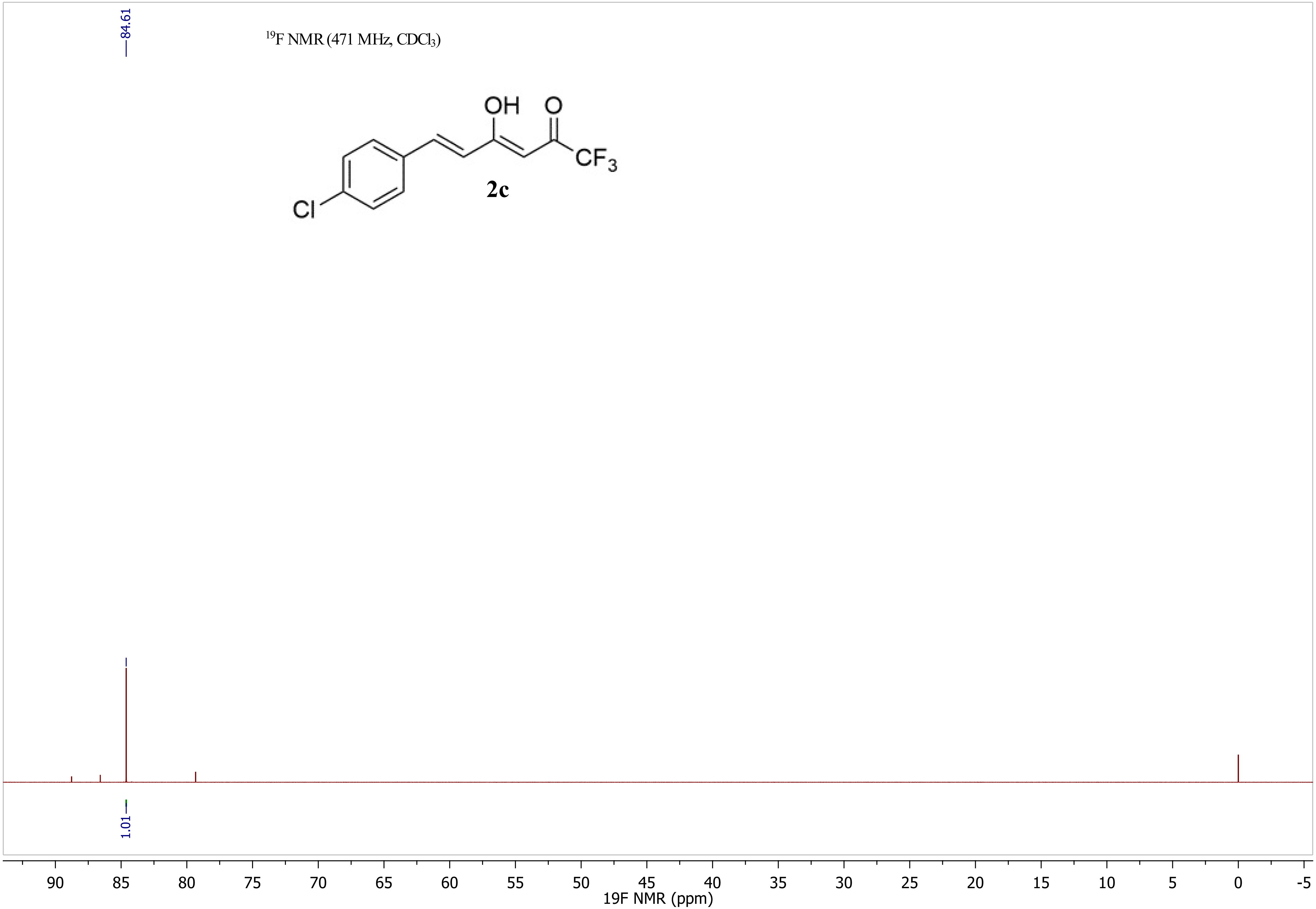
84.61
19F NMR (471 MHz, CDCl3)
OH
O
CF3
Cl
2c
1.01
90
85
80
75
70
65
60
55
50
45
40
35
30
25
20
15
10
5
0
-5
19F NMR (ppm)

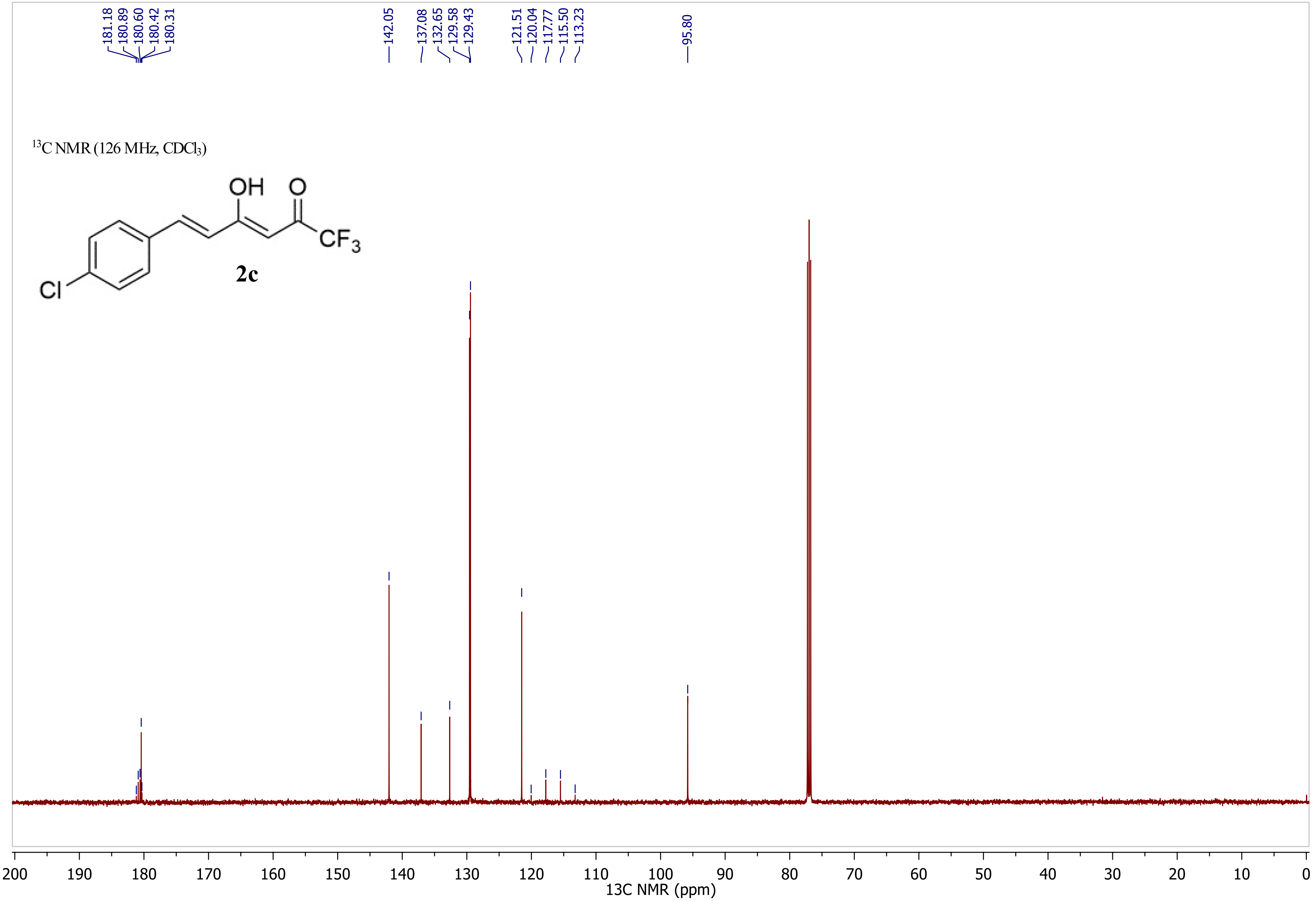
181.18
180.89
180.60
180.42
180.31
142.05
137.08
132.65
129.58
129.43
121.51
120.04
117.77
115.50
113.23
95.80
^{13}C NMR (126 MHz, $CDCl_3$)
OH
O
CF_3
Cl
2c
200 190 180 170 160 150 140 130 120 110 100 90 80 70 60 50 40 30 20 10 0
13C NMR (ppm)

^{1}H NMR (400 MHz, $CDCl_3$)

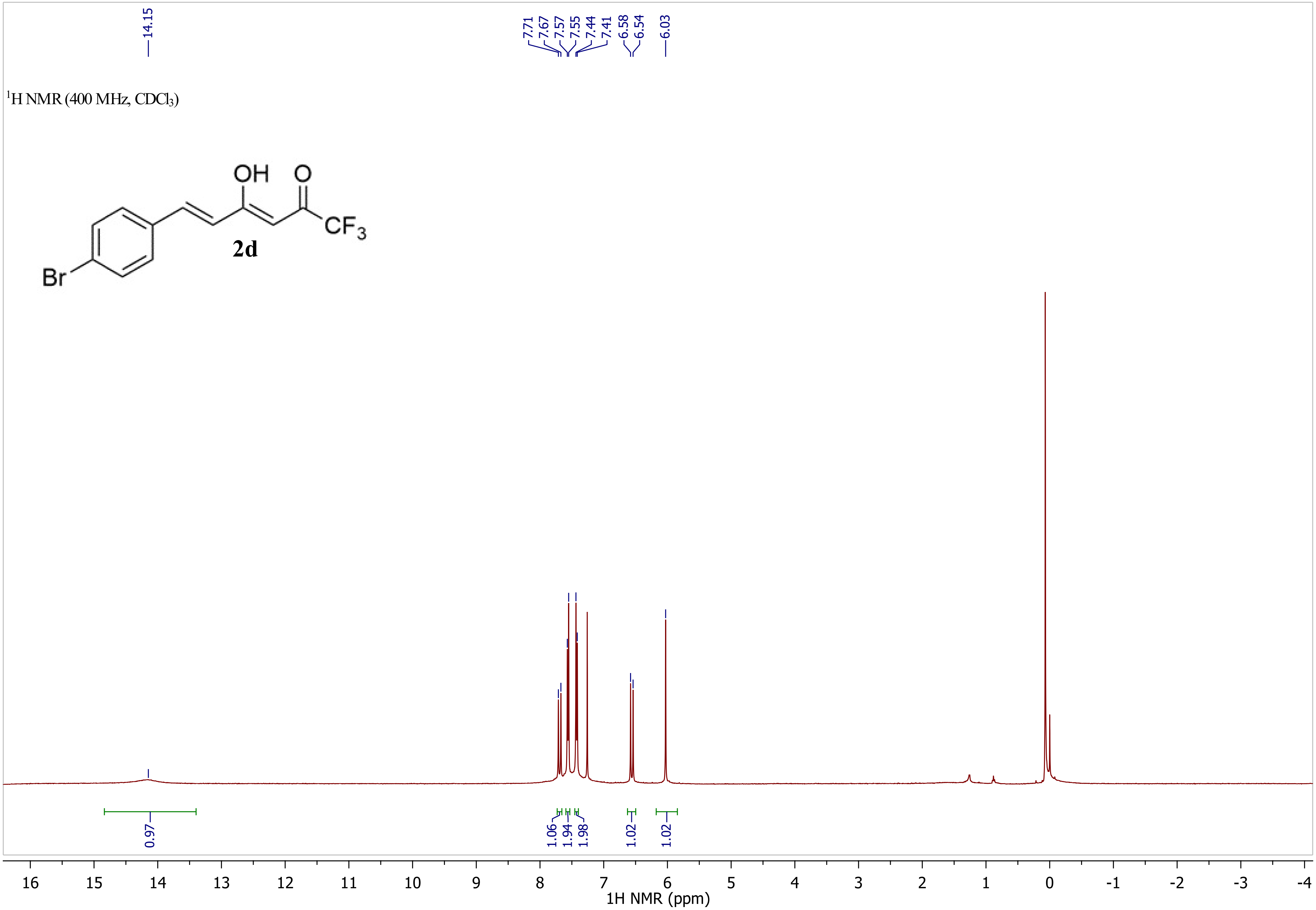

2d

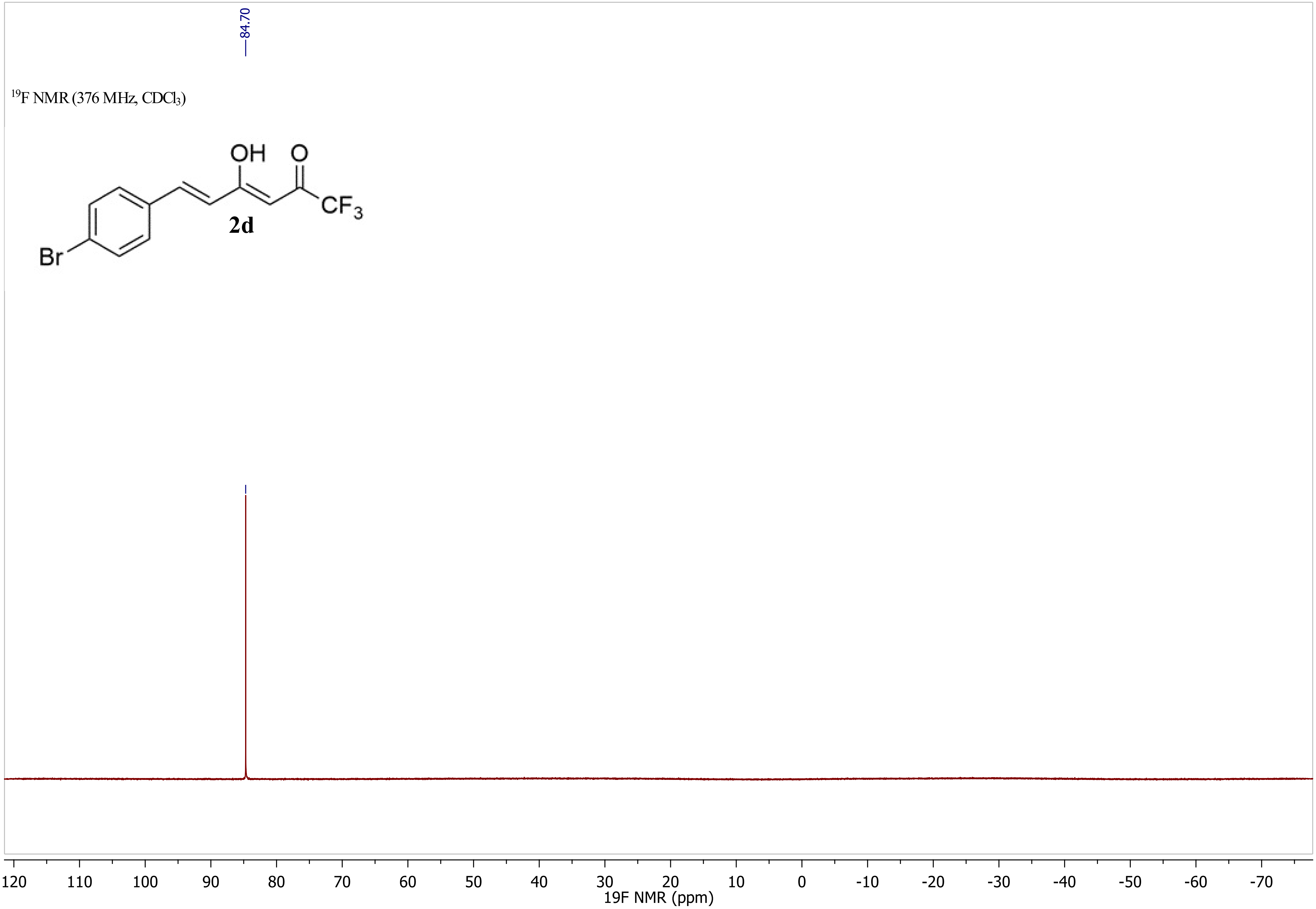
84.70
19F NMR (376 MHz, CDCl3)
OH
O
CF3
Br
2d
120 110 100 90 80 70 60 50 40 30 20 10 0 -10 -20 -30 -40 -50 -60 -70
19F NMR (ppm)

^{13}C NMR (101 MHz, $CDCl_3$)

2d

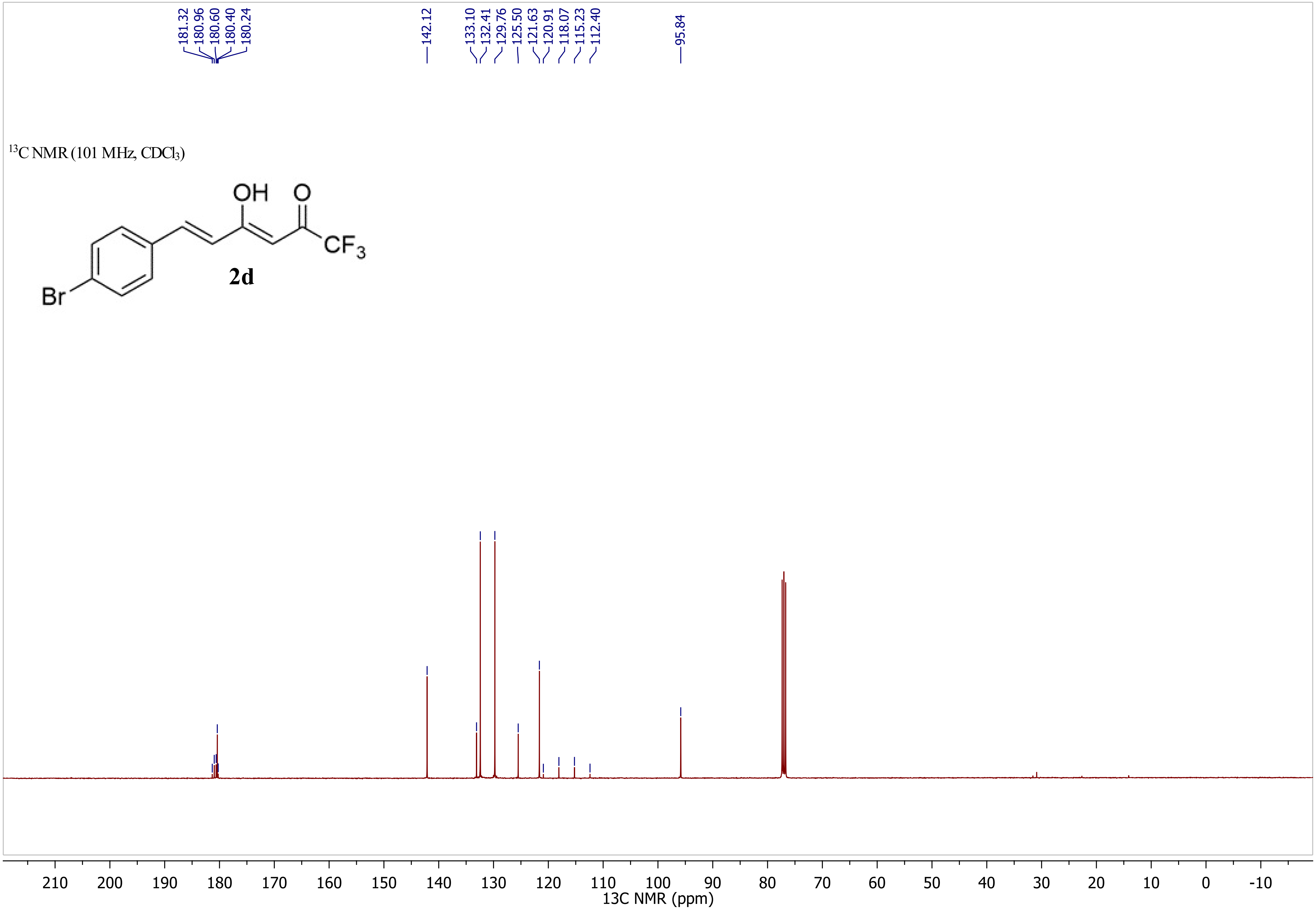

^{1}H NMR (400 MHz, $CDCl_3$)

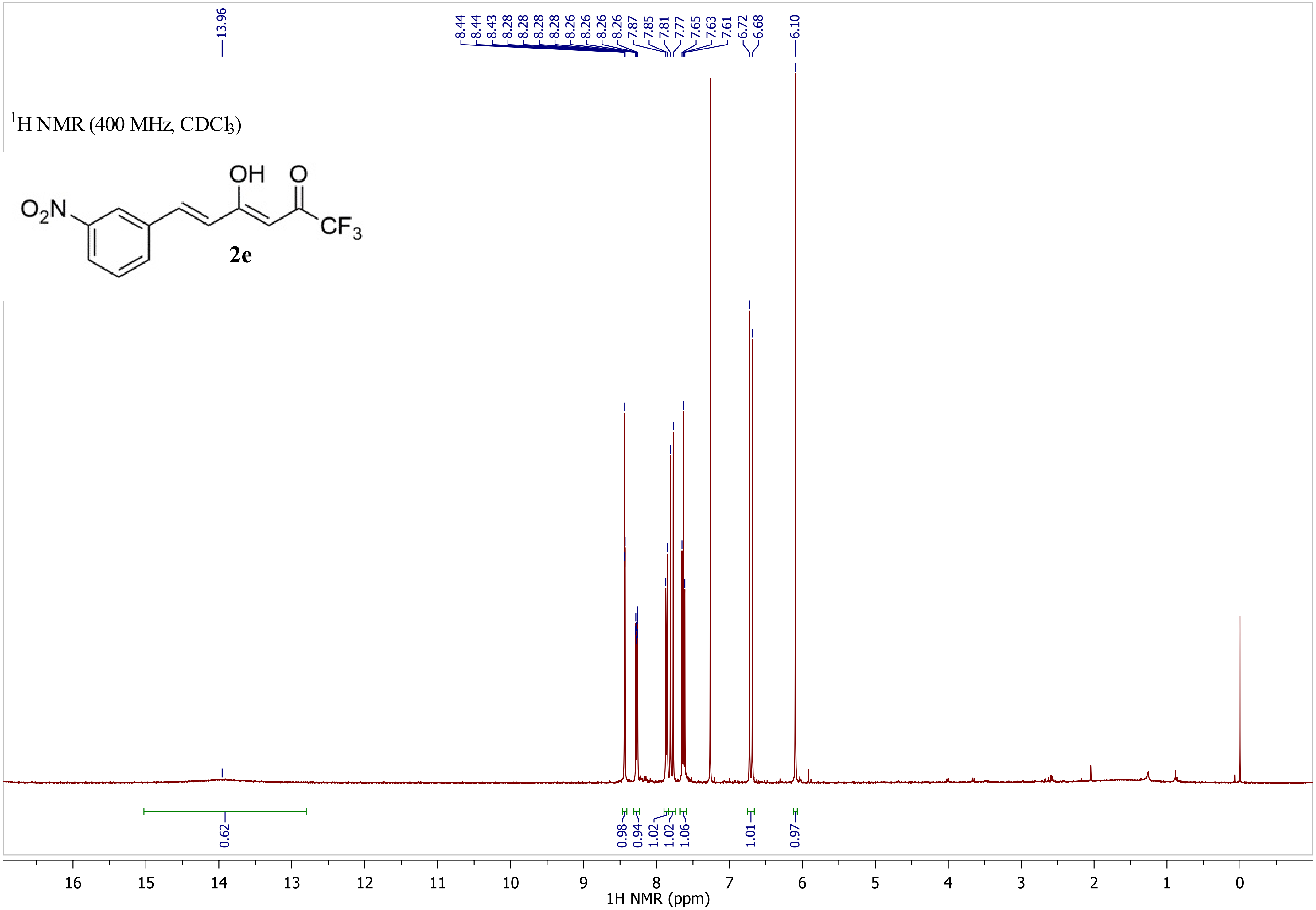

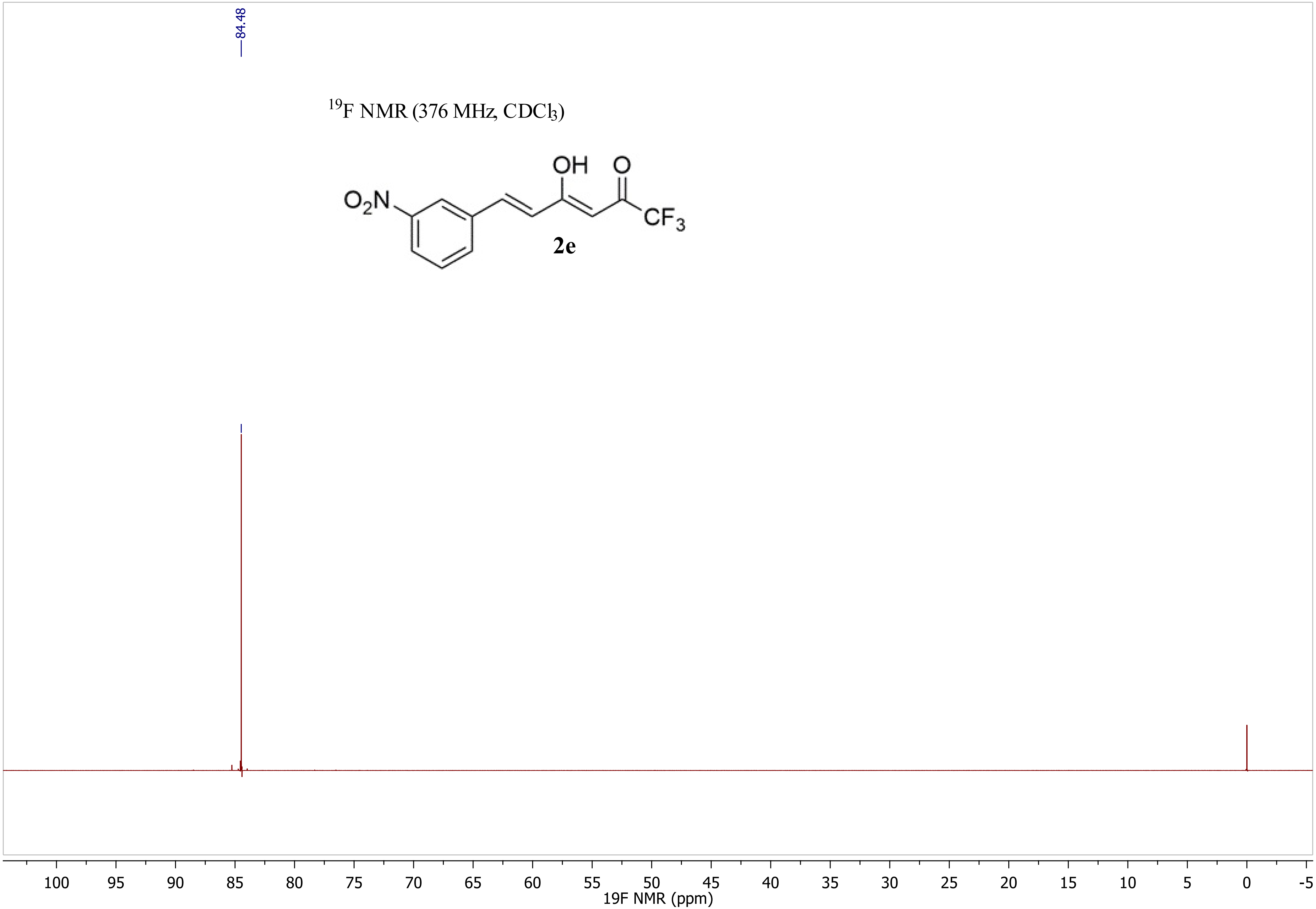

84.48
^{19}F NMR (376 MHz, CDCl$_3$)
OH
O
O_2N
CF_3
2e
100 95 90 85 80 75 70 65 60 55 50 45 40 35 30 25 20 15 10 5 0 -5
19F NMR (ppm)

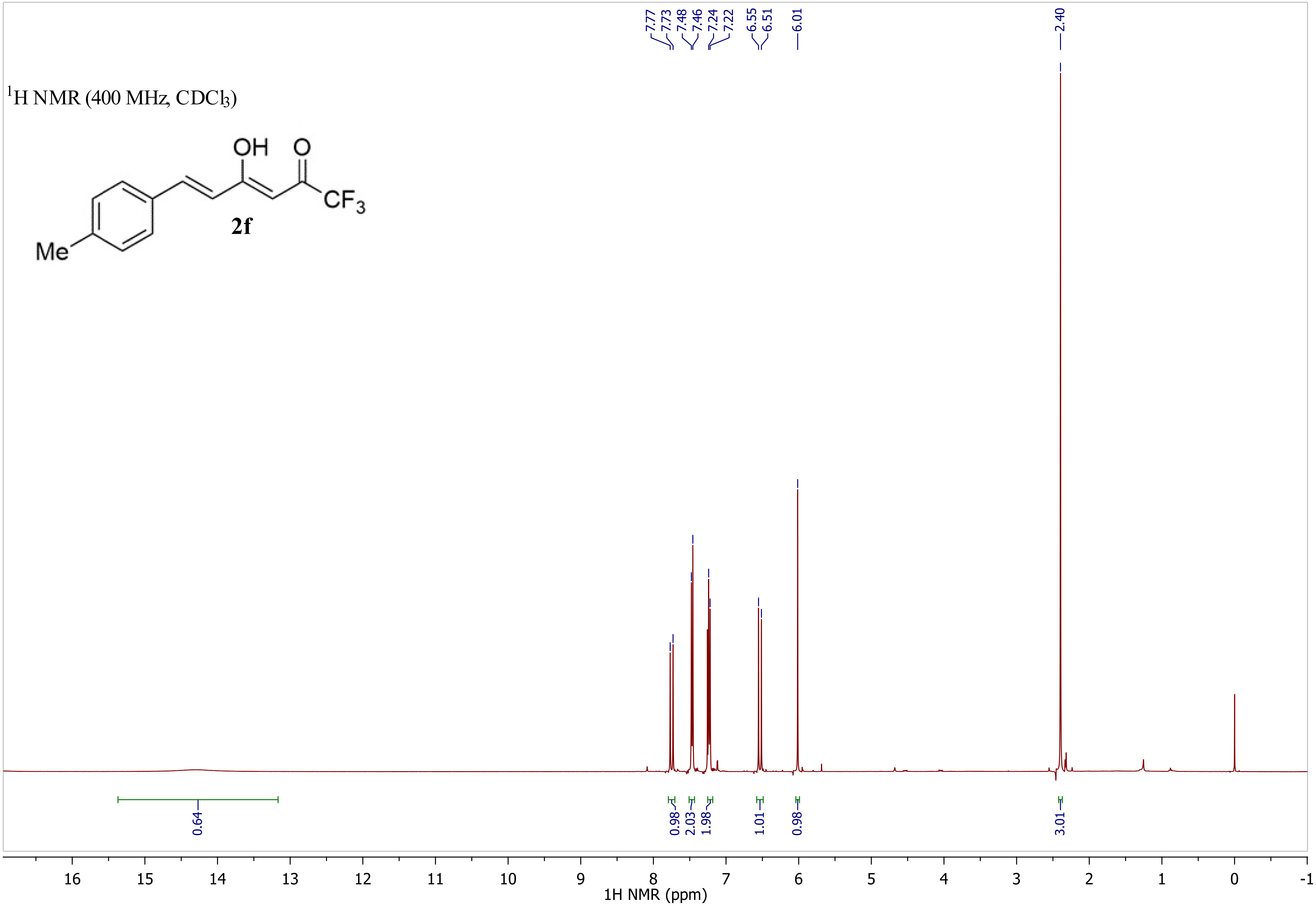

7.77
7.73
7.48
7.46
7.24
7.22
6.55
6.51
6.01
2.40
^{1}H NMR (400 MHz, $CDCl_3$)
OH
O
CF_3
Me
2f
0.64
0.98
2.03
1.98
1.01
0.98
3.01
16 15 14 13 12 11 10 9 8 7 6 5 4 3 2 1 0 -1
1H NMR (ppm)

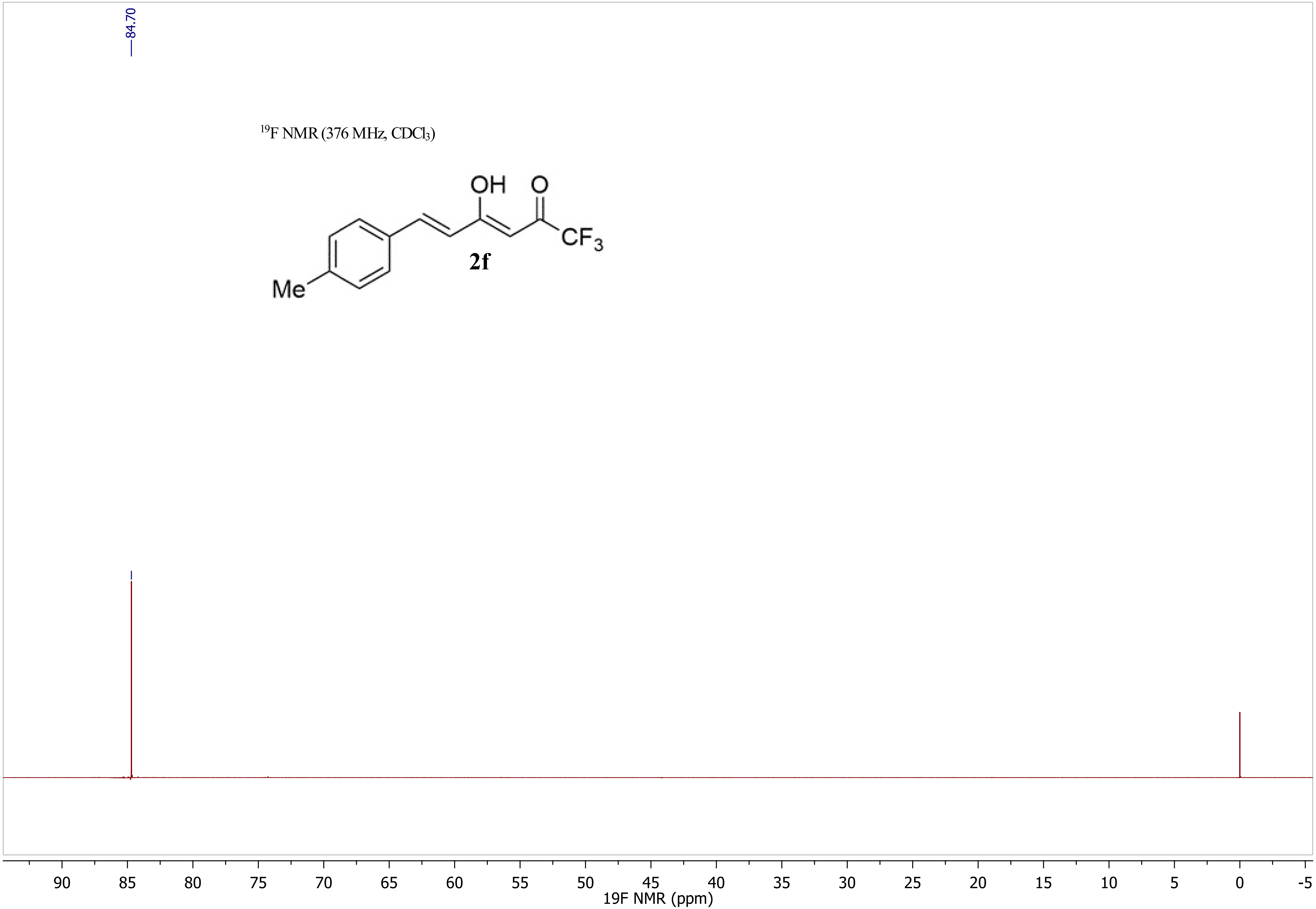
84.70
19F NMR (376 MHz, CDCl3)
OH
O
CF3
Me
2f
90 85 80 75 70 65 60 55 50 45 40 35 30 25 20 15 10 5 0 -5
19F NMR (ppm)

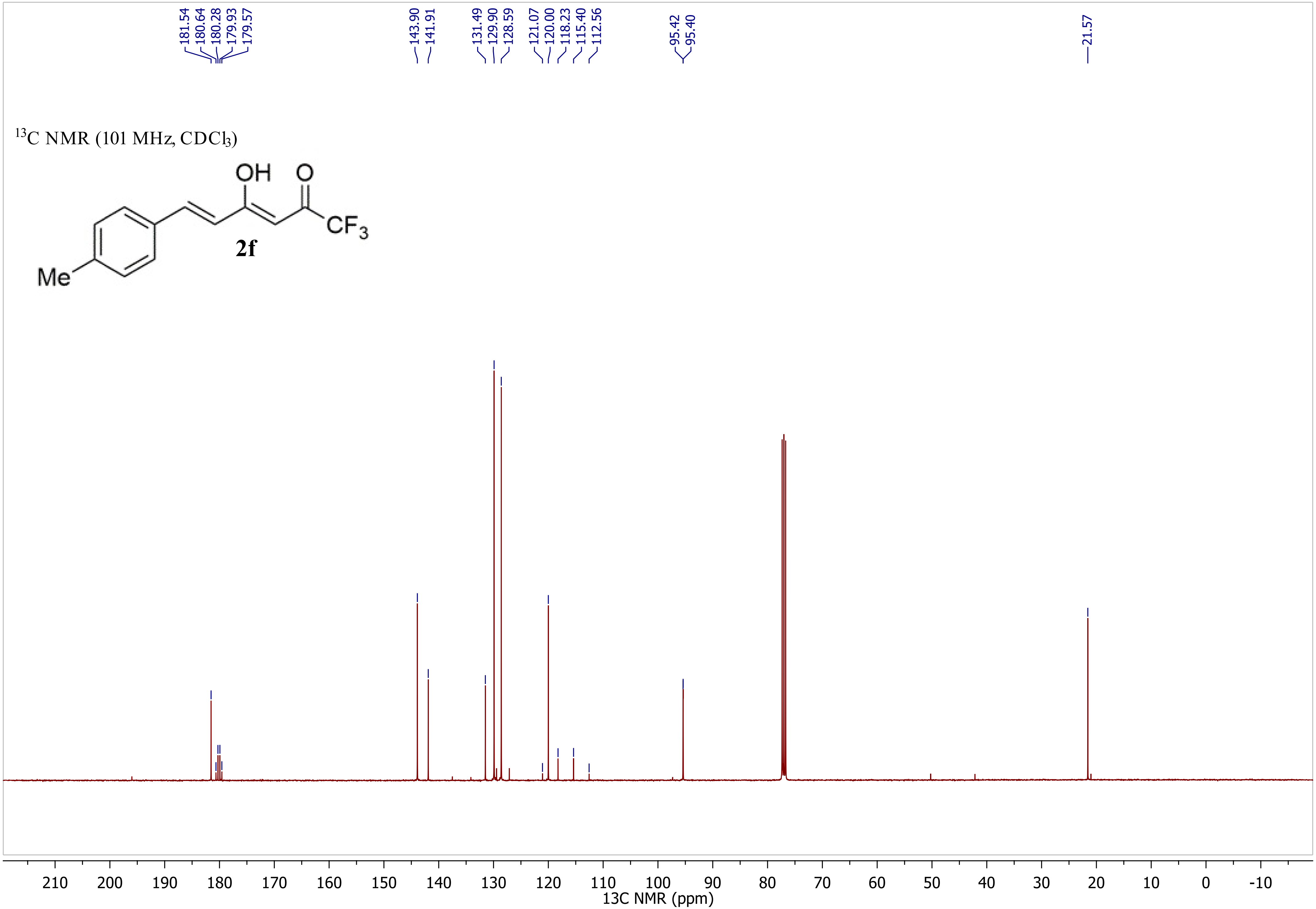

181.54
180.64
180.28
179.93
179.57
143.90
141.91
131.49
129.90
128.59
121.07
120.00
118.23
115.40
112.56
95.42
95.40
21.57
^{13}C NMR (101 MHz, CDCl$_3$)
OH
O
CF$_3$
2f
Me
210 200 190 180 170 160 150 140 130 120 110 100 90 80 70 60 50 40 30 20 10 0 -10
13C NMR (ppm)

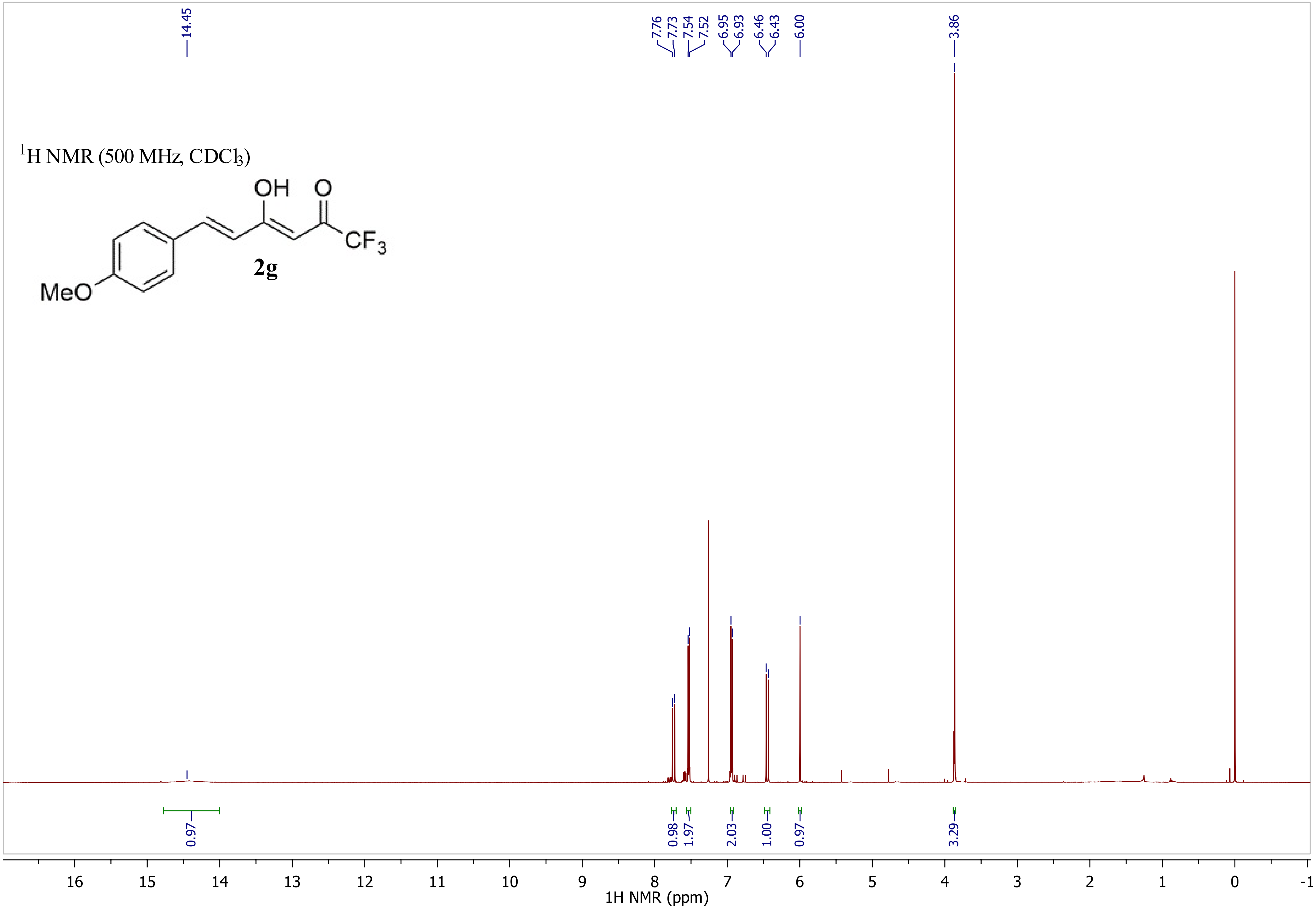

14.45
7.76
7.73
7.54
7.52
6.95
6.93
6.46
6.43
6.00
3.86
^{1}H NMR (500 MHz, CDCl$_3$)
OH
O
CF$_3$
MeO
2g
0.97
0.98
1.97
2.03
1.00
0.97
3.29
16 15 14 13 12 11 10 9 8 7 6 5 4 3 2 1 0 -1
1H NMR (ppm)

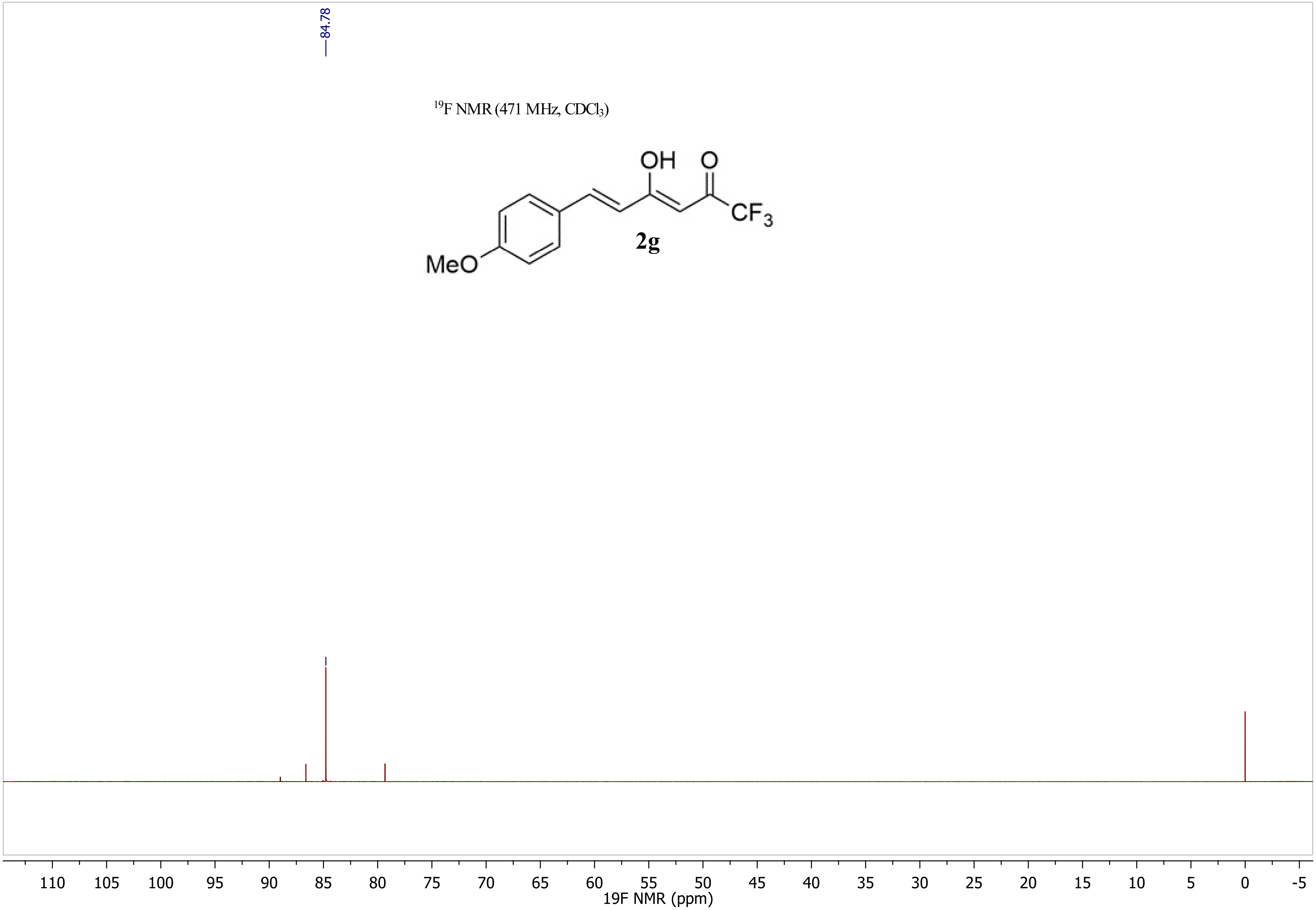

84.78
19F NMR (471 MHz, CDCl3)
OH O
CF3
MeO
2g
110 105 100 95 90 85 80 75 70 65 60 55 50 45 40 35 30 25 20 15 10 5 0 -5
19F NMR (ppm)

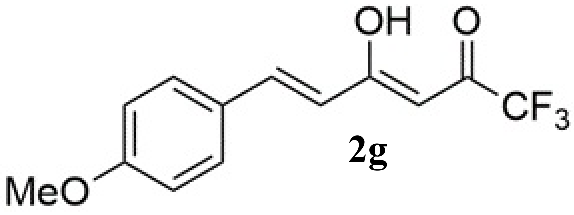
OH
O
CF3
MeO
2g

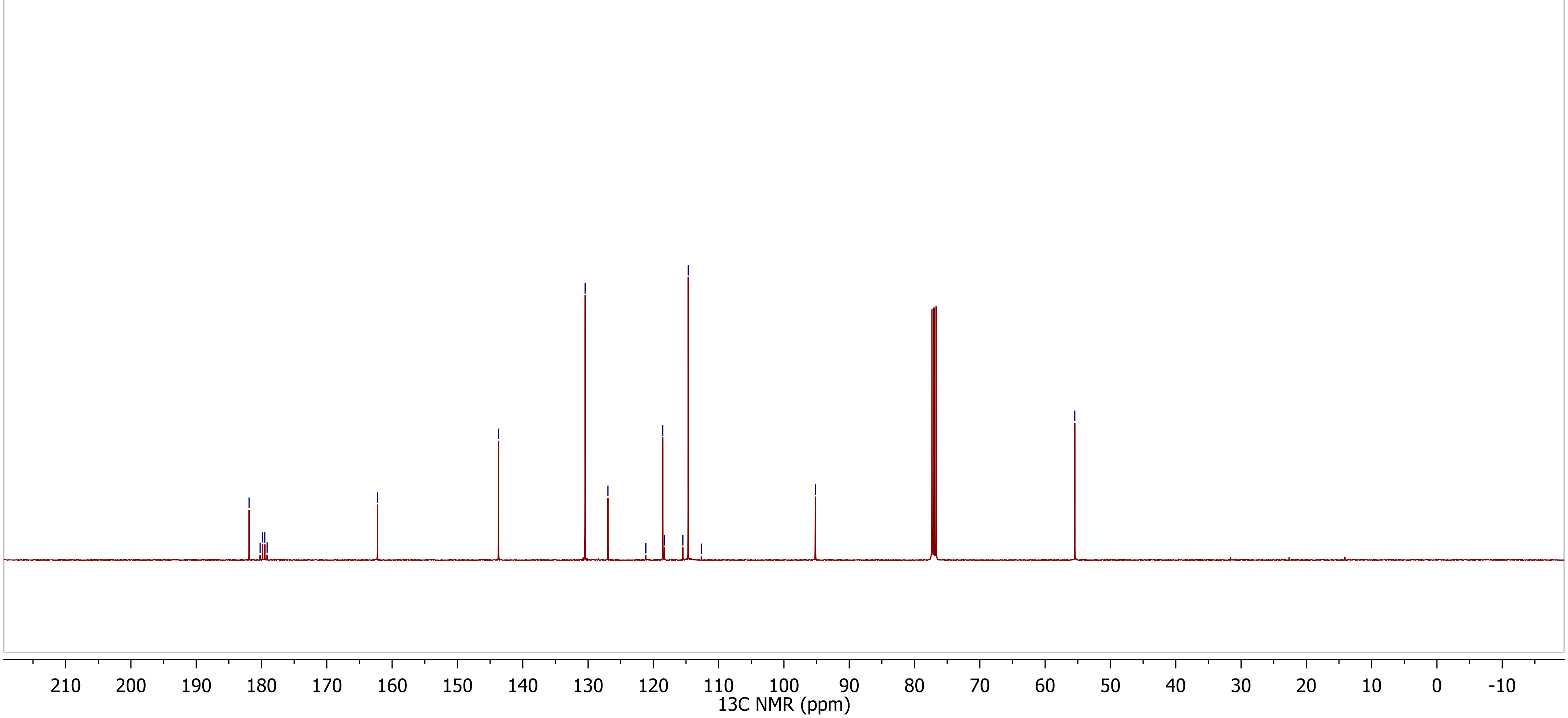
181.90
180.21
179.86
179.50
179.14
162.25
143.71
130.45
126.95
121.14
118.55
118.30
115.47
114.65
112.64
95.19
95.18
55.46
13C NMR (101 MHz, CDCl3)
210 200 190 180 170 160 150 140 130 120 110 100 90 80 70 60 50 40 30 20 10 0 -10
13C NMR (ppm)

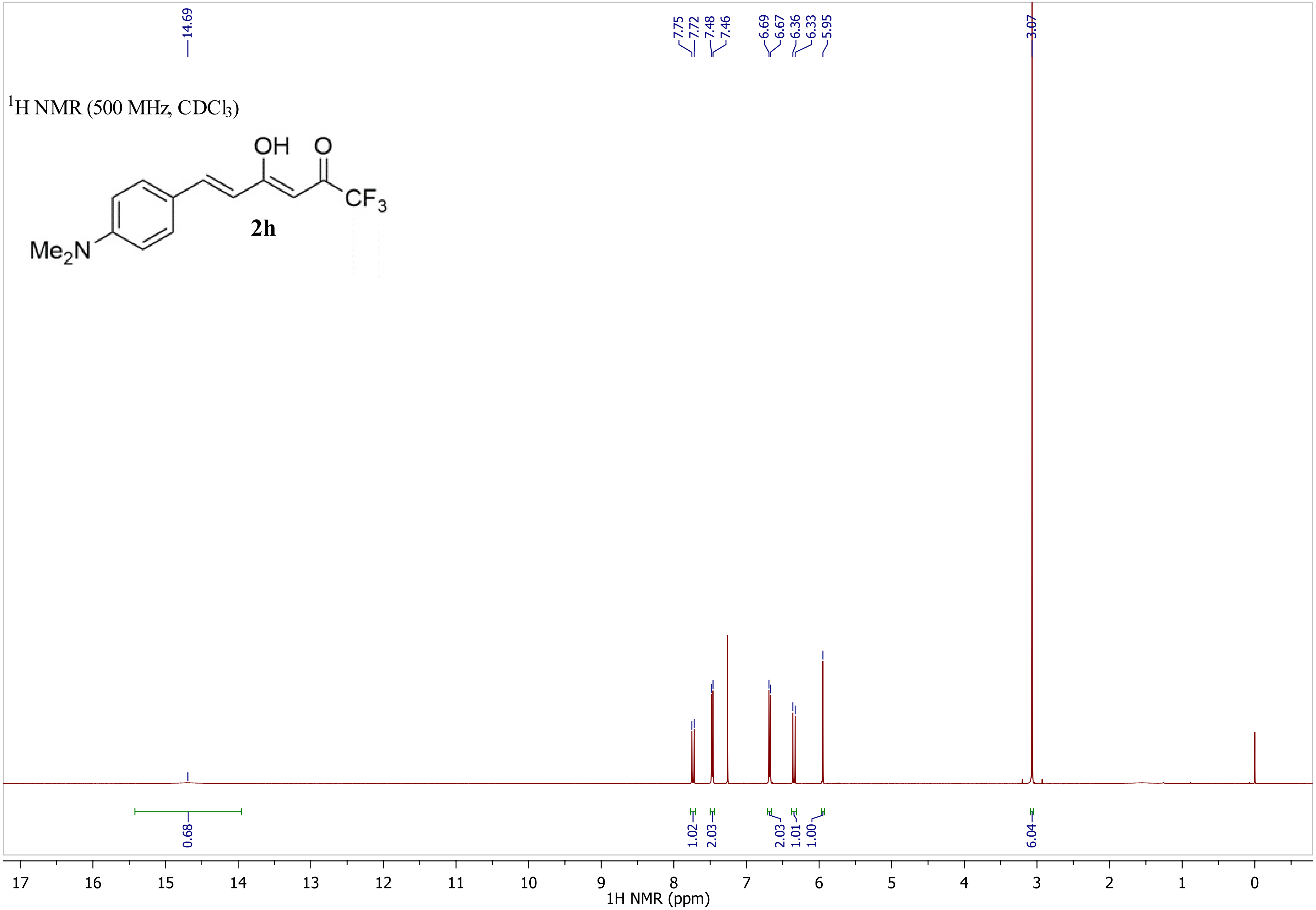
14.69
7.75
7.72
7.48
7.46
6.69
6.67
6.36
6.33
5.95
3.07
1H NMR (500 MHz, CDCl3)
OH
O
CF3
Me2N
2h
0.68
1.02
2.03
2.03
1.01
1.00
6.04
17
16
15
14
13
12
11
10
9
8
7
6
5
4
3
2
1
0
1H NMR (ppm)

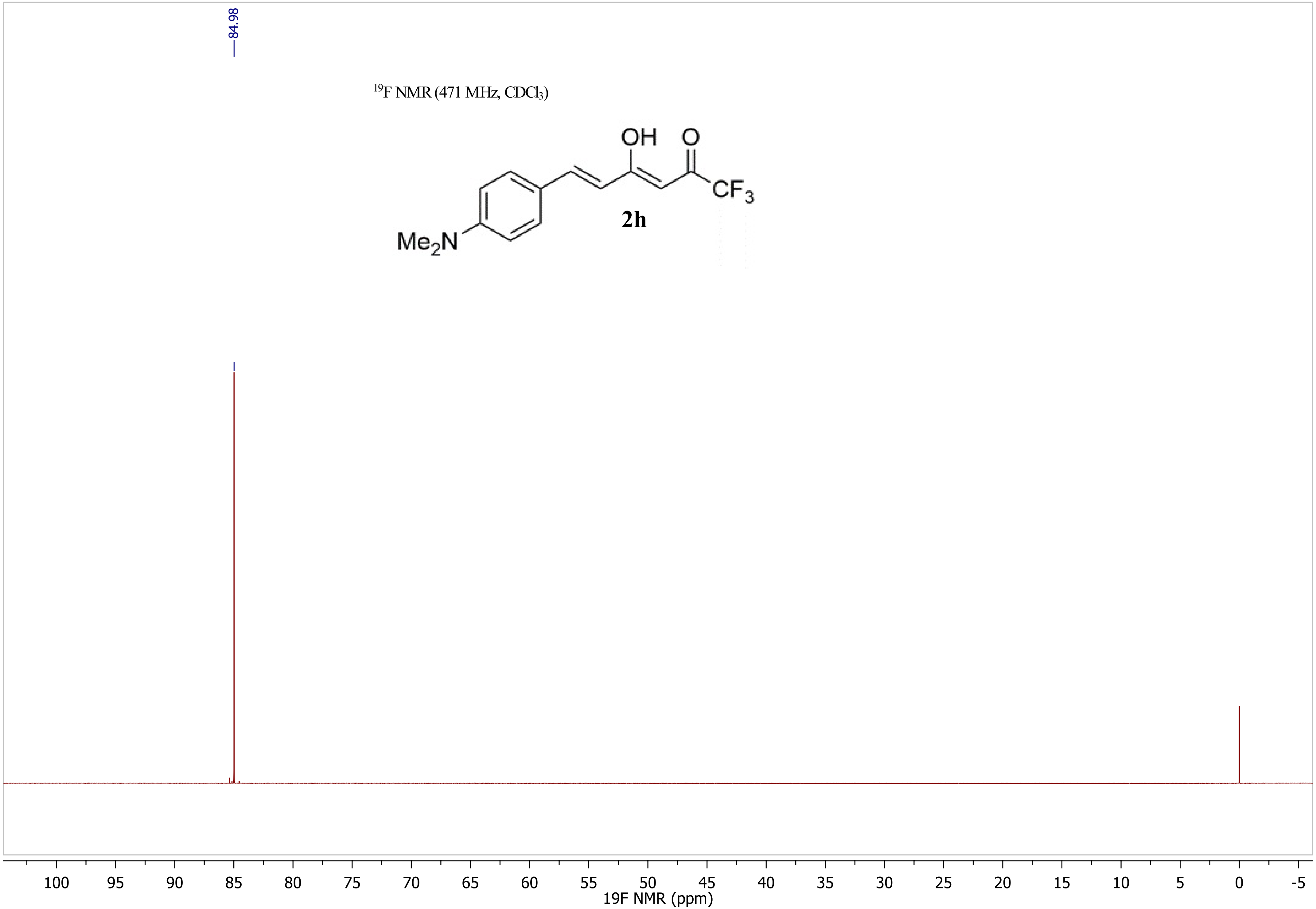

84.98
19F NMR (471 MHz, CDCl3)
OH
O
CF3
Me2N
2h
100
95
90
85
80
75
70
65
60
55
50
45
40
35
30
25
20
15
10
5
0
-5
19F NMR (ppm)

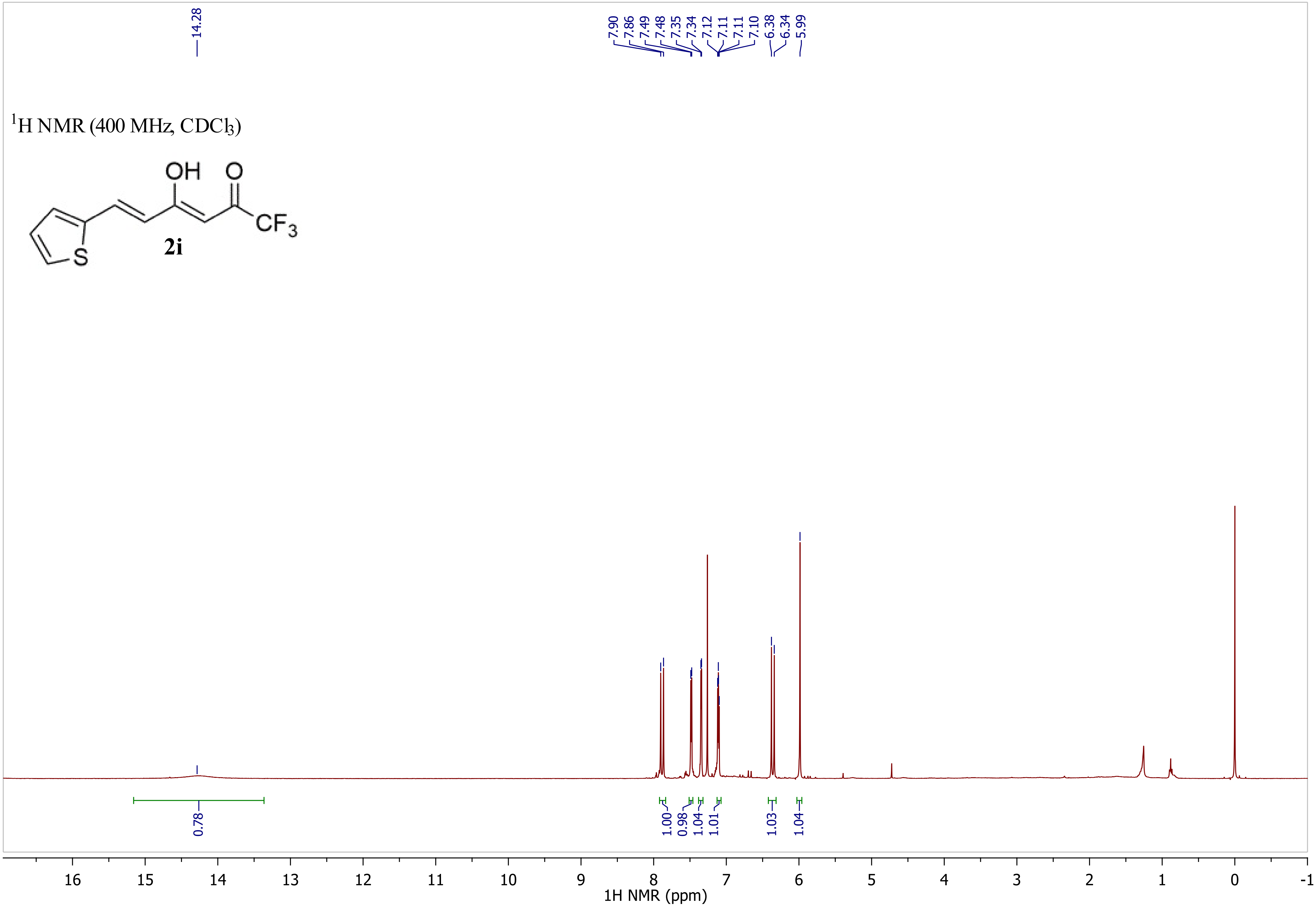
1H NMR (400 MHz, CDCl3)
OH
O
CF3
S
2i
14.28
7.90
7.86
7.49
7.48
7.35
7.34
7.12
7.11
7.11
7.10
6.38
6.34
5.99
0.78
1.00
0.98
1.04
1.01
1.03
1.04
16
15
14
13
12
11
10
9
8
7
6
5
4
3
2
1
0
-1
1H NMR (ppm)

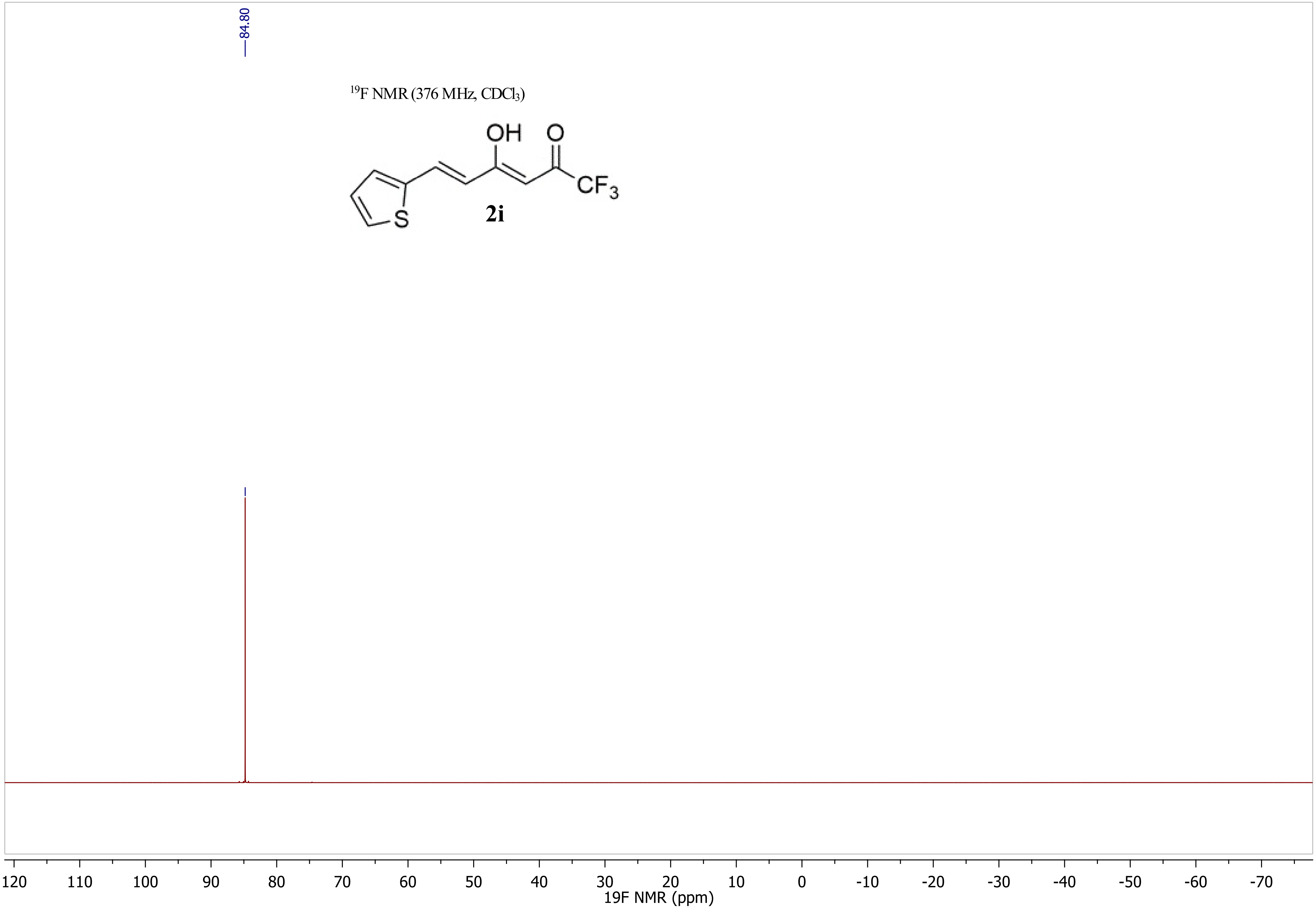

84.80
^{19}F NMR (376 MHz, $CDCl_3$)
OH
O
CF_3
S
2i
120 110 100 90 80 70 60 50 40 30 20 10 0 -10 -20 -30 -40 -50 -60 -70
19F NMR (ppm)

^{13}C NMR (101 MHz, $CDCl_3$)

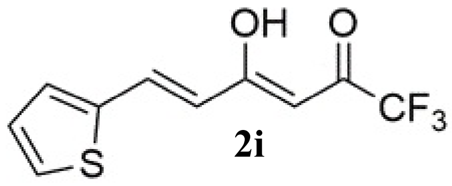

2i

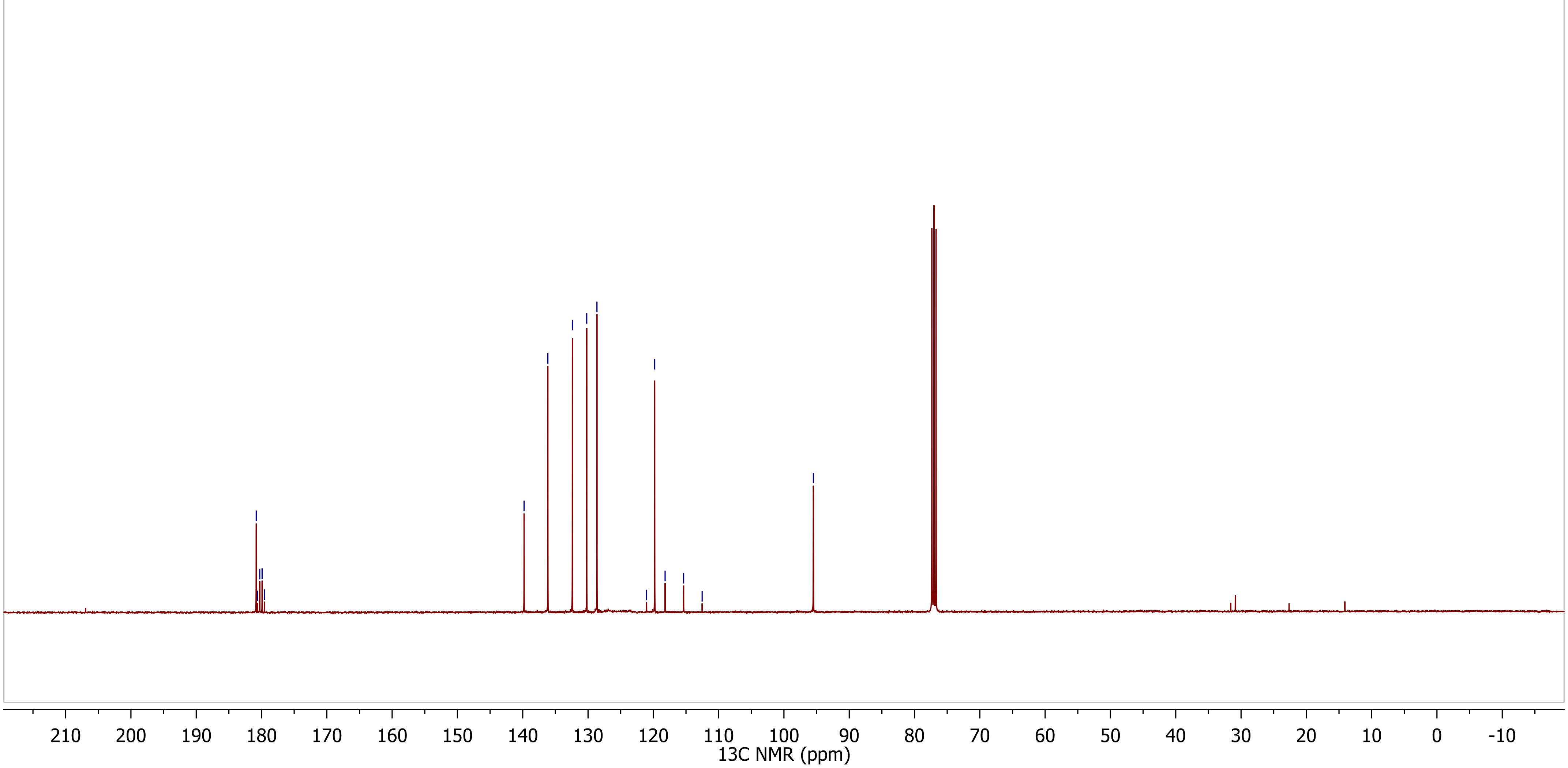

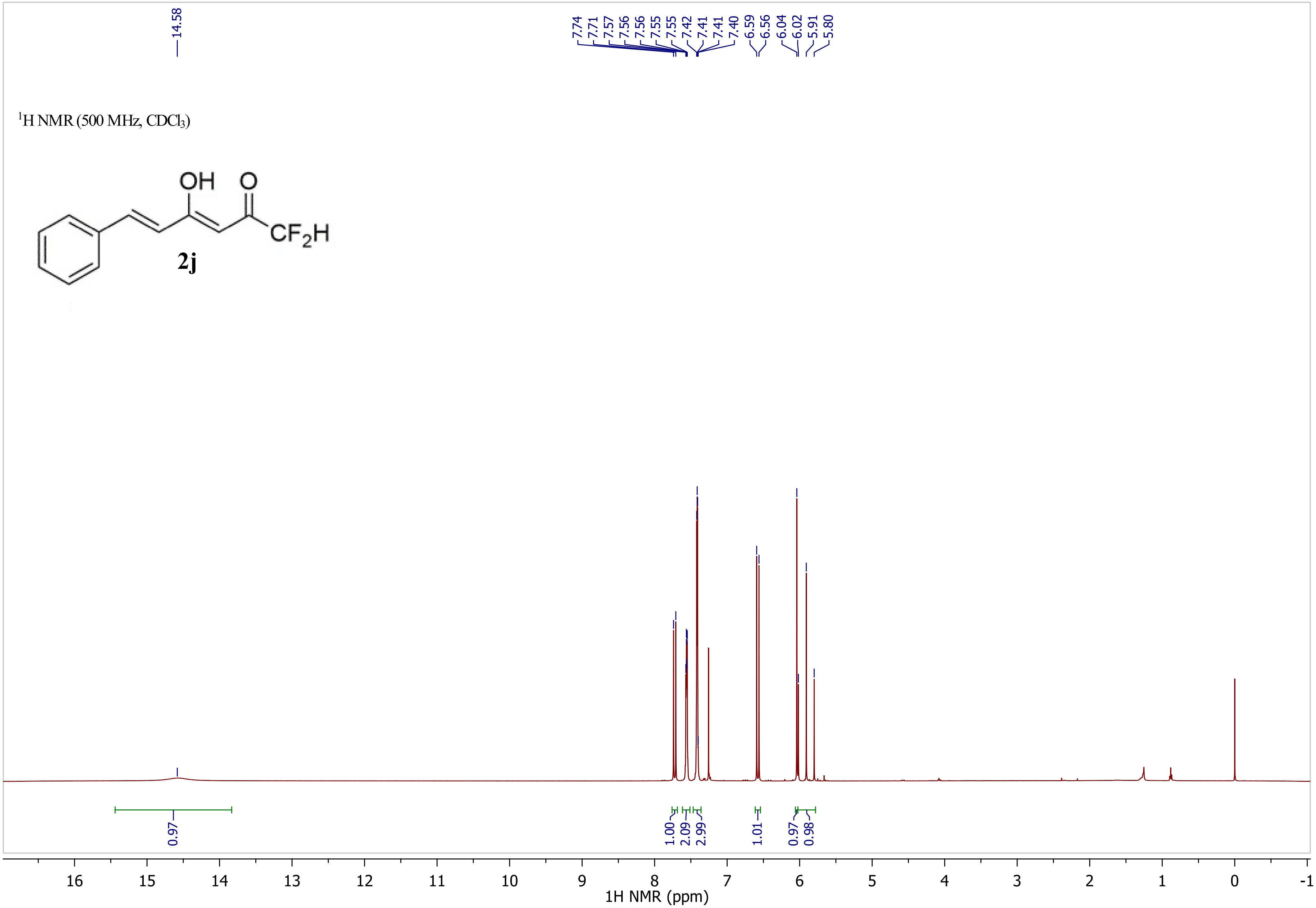

14.58
7.74 7.71 7.57 7.56 7.56 7.55 7.55 7.42 7.41 7.41 7.40 6.59 6.56 6.04 6.02 5.91 5.80
1H NMR (500 MHz, CDCl3)
OH O
CF2H
2j
0.97
1.00 2.09 2.99
1.01
0.97 0.98
16 15 14 13 12 11 10 9 8 7 6 5 4 3 2 1 0 -1
1H NMR (ppm)

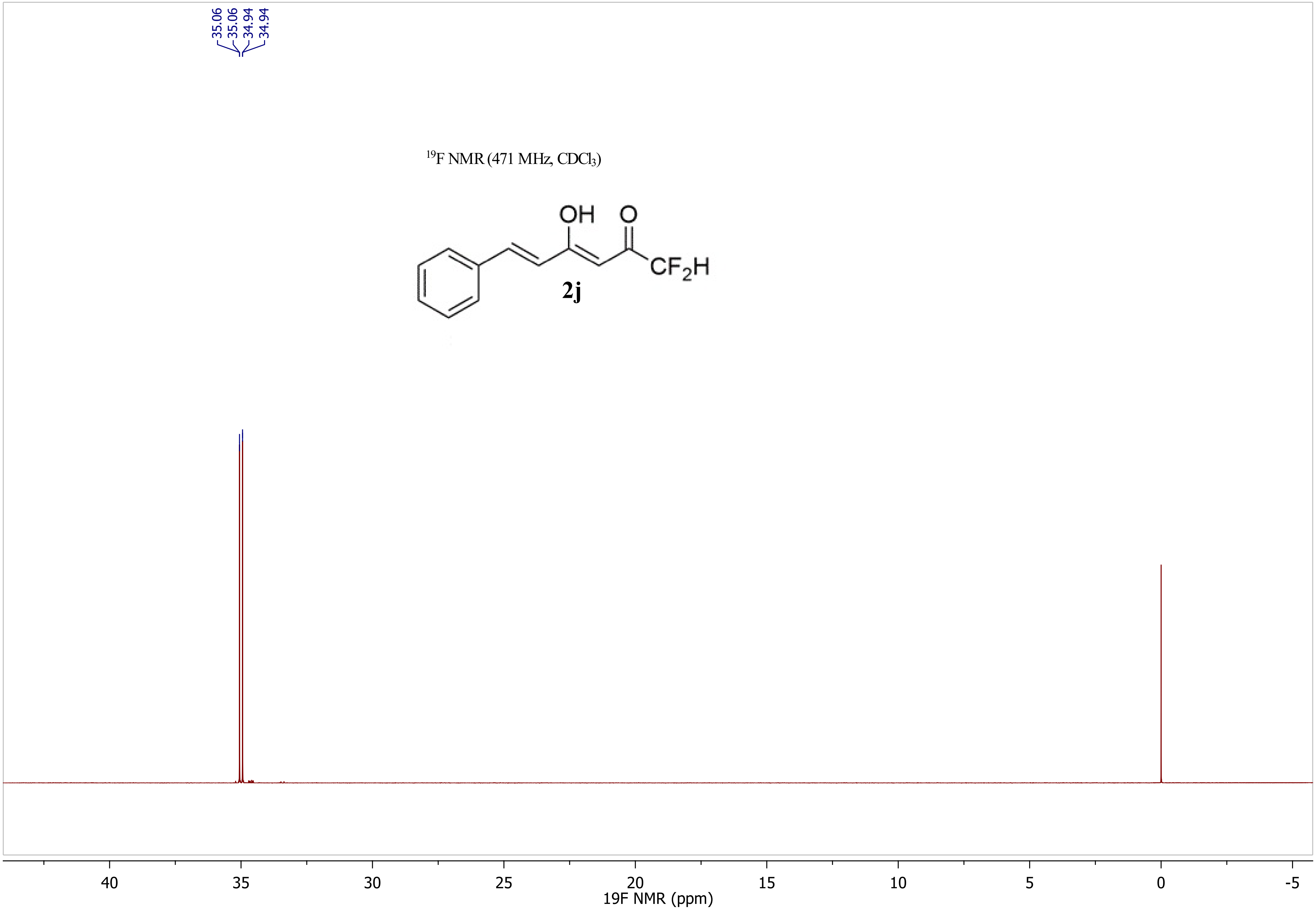
35.06
35.06
34.94
34.94
19F NMR (471 MHz, CDCl3)
OH O
CF2H
2j
40 35 30 25 20 15 10 5 0 -5
19F NMR (ppm)

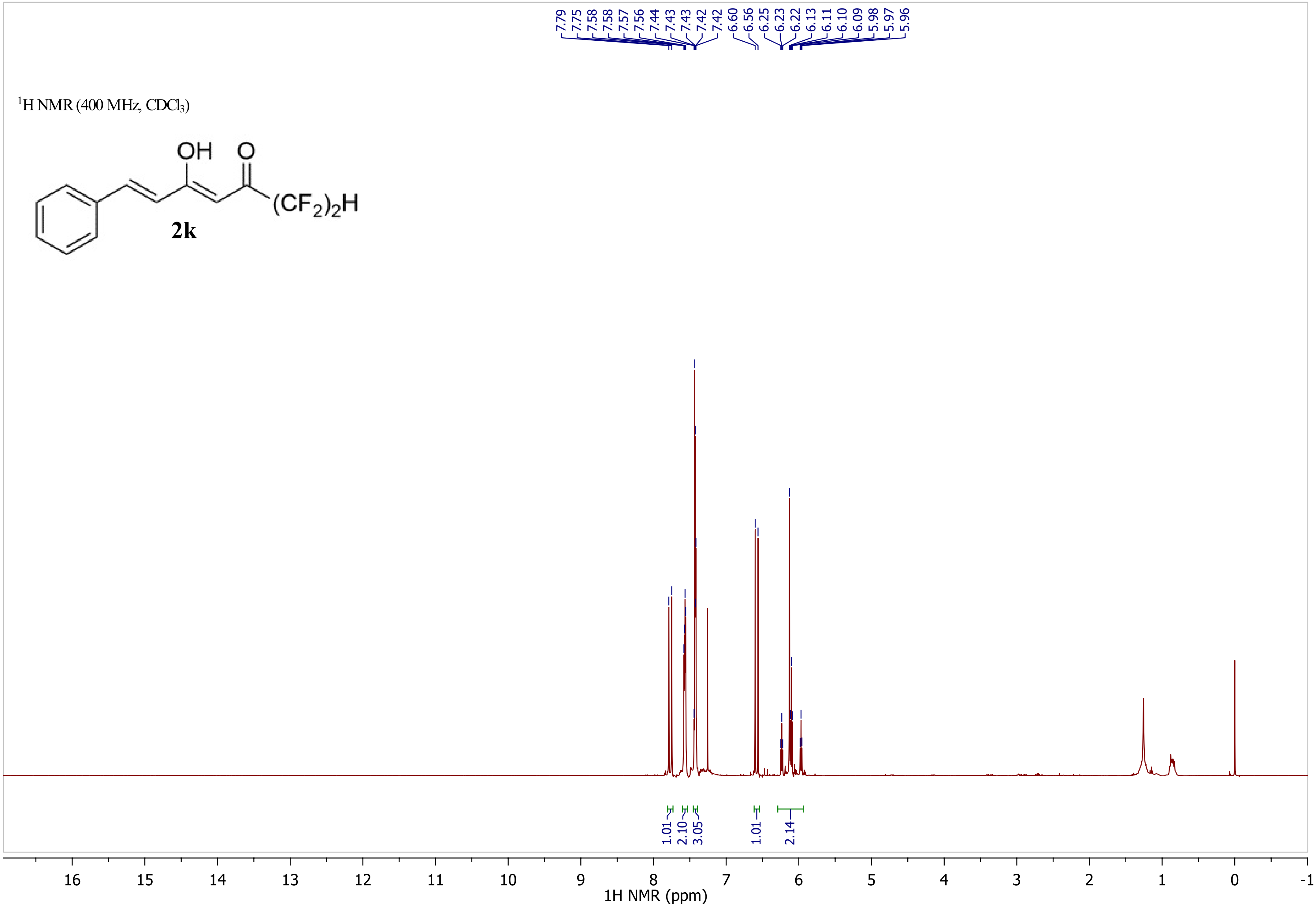
7.79 7.75 7.58 7.58 7.57 7.56 7.44 7.43 7.43 7.42 7.42 6.60 6.56 6.25 6.23 6.22 6.13 6.11 6.10 6.09 5.98 5.97 5.96
1H NMR (400 MHz, CDCl3)
OH
O
(CF2)2H
2k
1.01
2.10
3.05
1.01
2.14
16 15 14 13 12 11 10 9 8 7 6 5 4 3 2 1 0 -1
1H NMR (ppm)

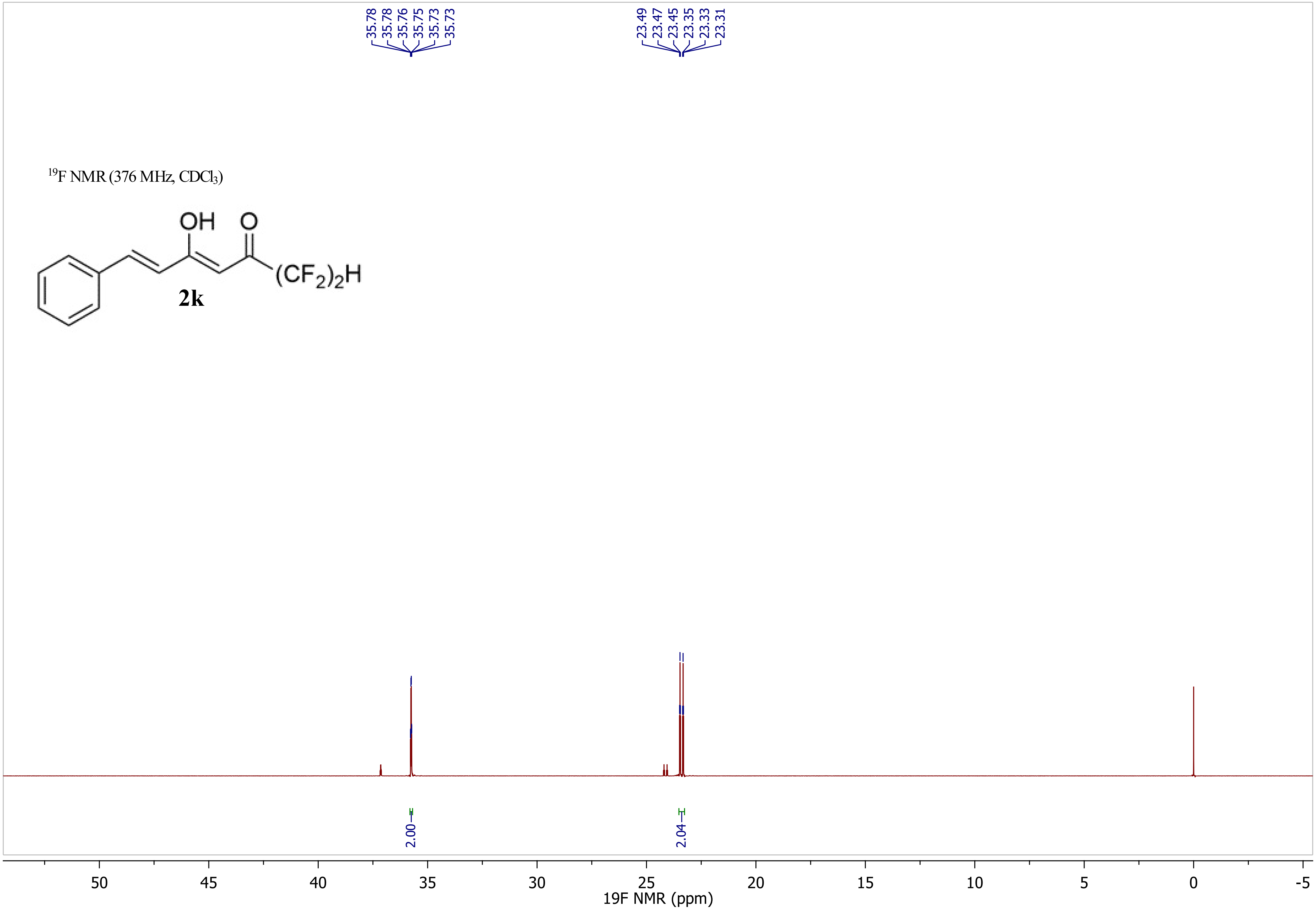

^{19}F NMR (376 MHz, CDCl$_3$)
OH
O
$(CF_2)_2H$
2k
35.78
35.78
35.76
35.75
35.73
35.73
23.49
23.47
23.45
23.35
23.33
23.31
2.00
2.04
50
45
40
35
30
25
20
15
10
5
0
-5
19F NMR (ppm)

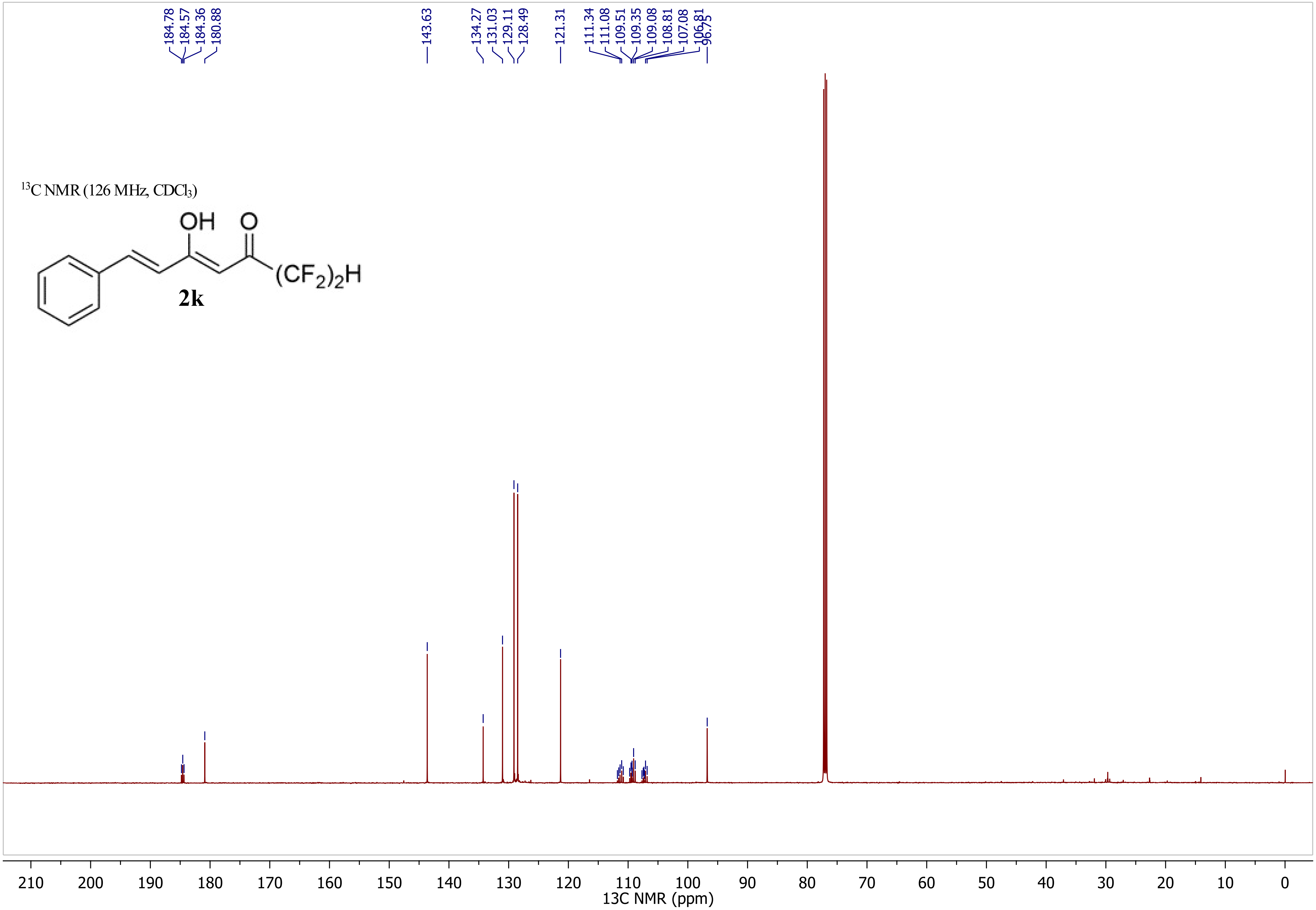
184.78
184.57
184.36
180.88
143.63
134.27
131.03
129.11
128.49
121.31
111.34
111.08
109.51
109.35
109.08
108.81
107.08
106.81
96.75
13C NMR (126 MHz, CDCl3)
OH
O
(CF2)2H
2k
210
200
190
180
170
160
150
140
130
120
110
100
90
80
70
60
50
40
30
20
10
0
13C NMR (ppm)

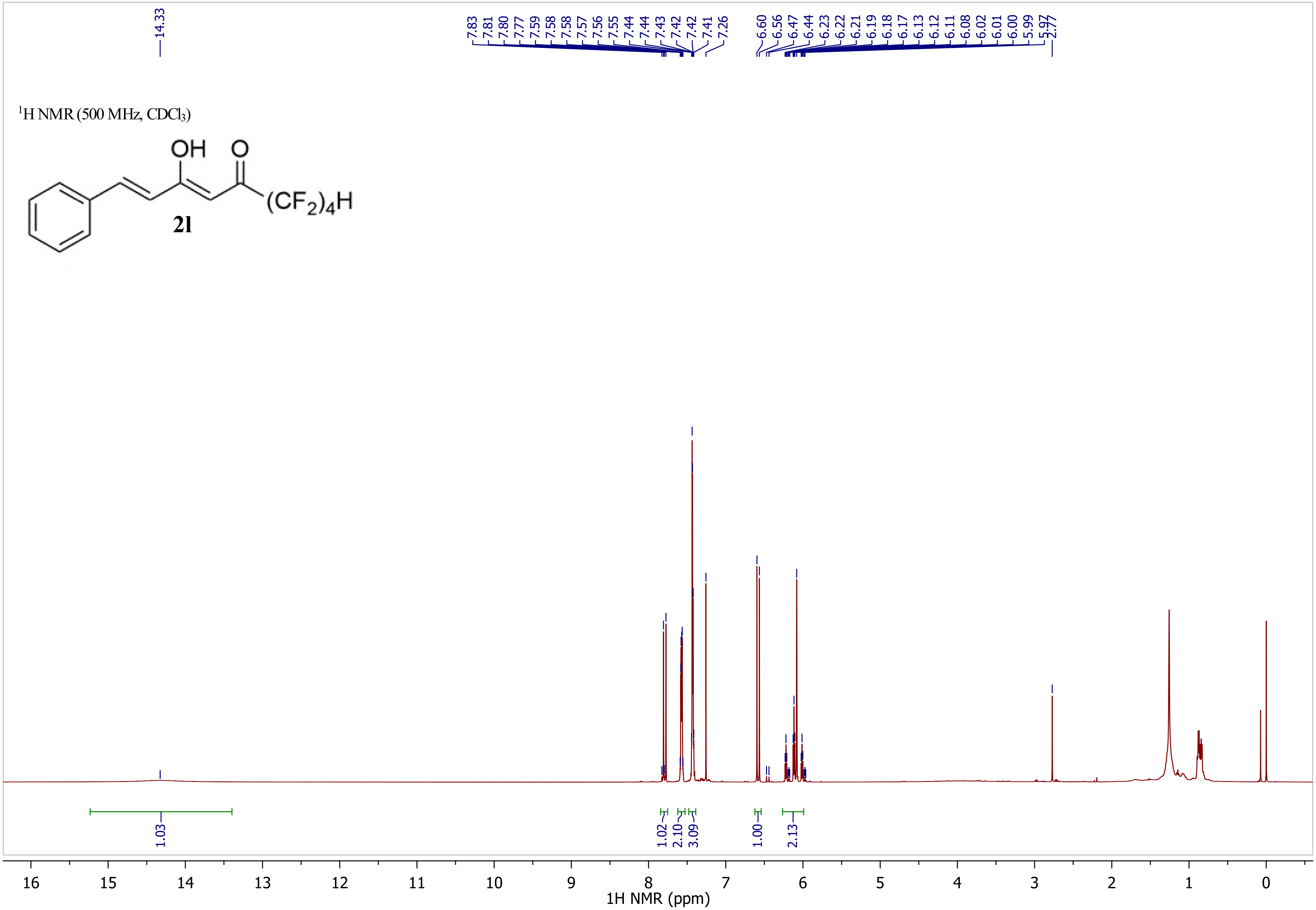
14.33
7.83 7.81 7.80 7.77 7.59 7.58 7.58 7.57 7.56 7.55 7.44 7.44 7.43 7.42 7.42 7.41 7.26
6.60 6.56 6.47 6.44 6.23 6.22 6.21 6.19 6.18 6.17 6.13 6.12 6.11 6.08 6.02 6.01 6.00 5.99 5.97 2.77
1H NMR (500 MHz, CDCl3)
OH O
(CF2)4H
2l
1.03
1.02 2.10 3.09
1.00
2.13
16 15 14 13 12 11 10 9 8 7 6 5 4 3 2 1 0
1H NMR (ppm)

^{13}C NMR (126 MHz, $CDCl_3$)

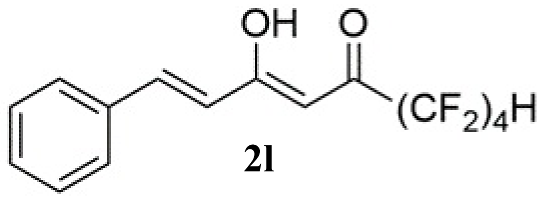

2l

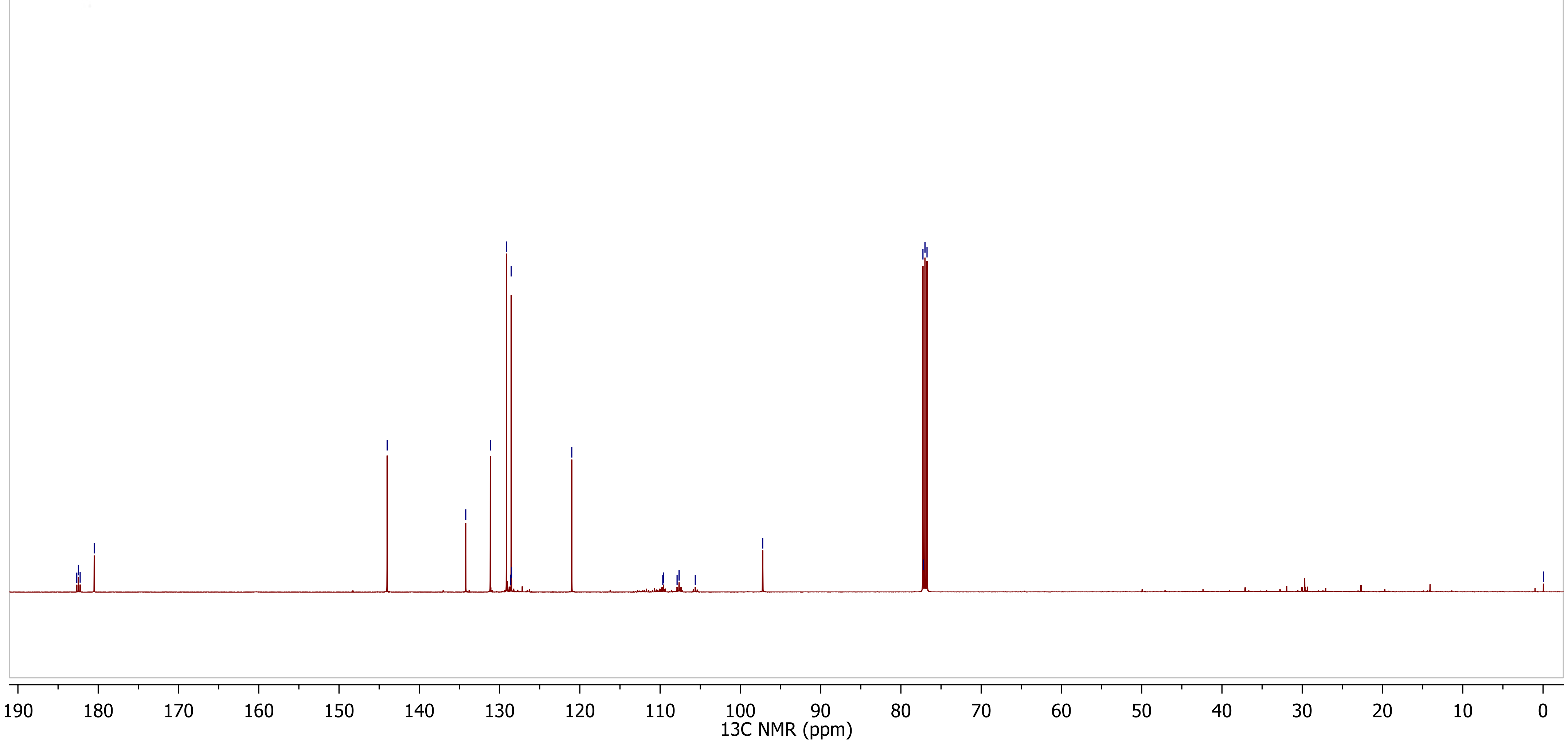

^{19}F NMR (471 MHz, $CDCl_3$)

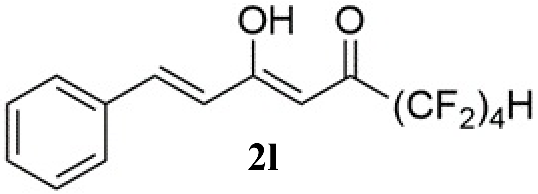

2l

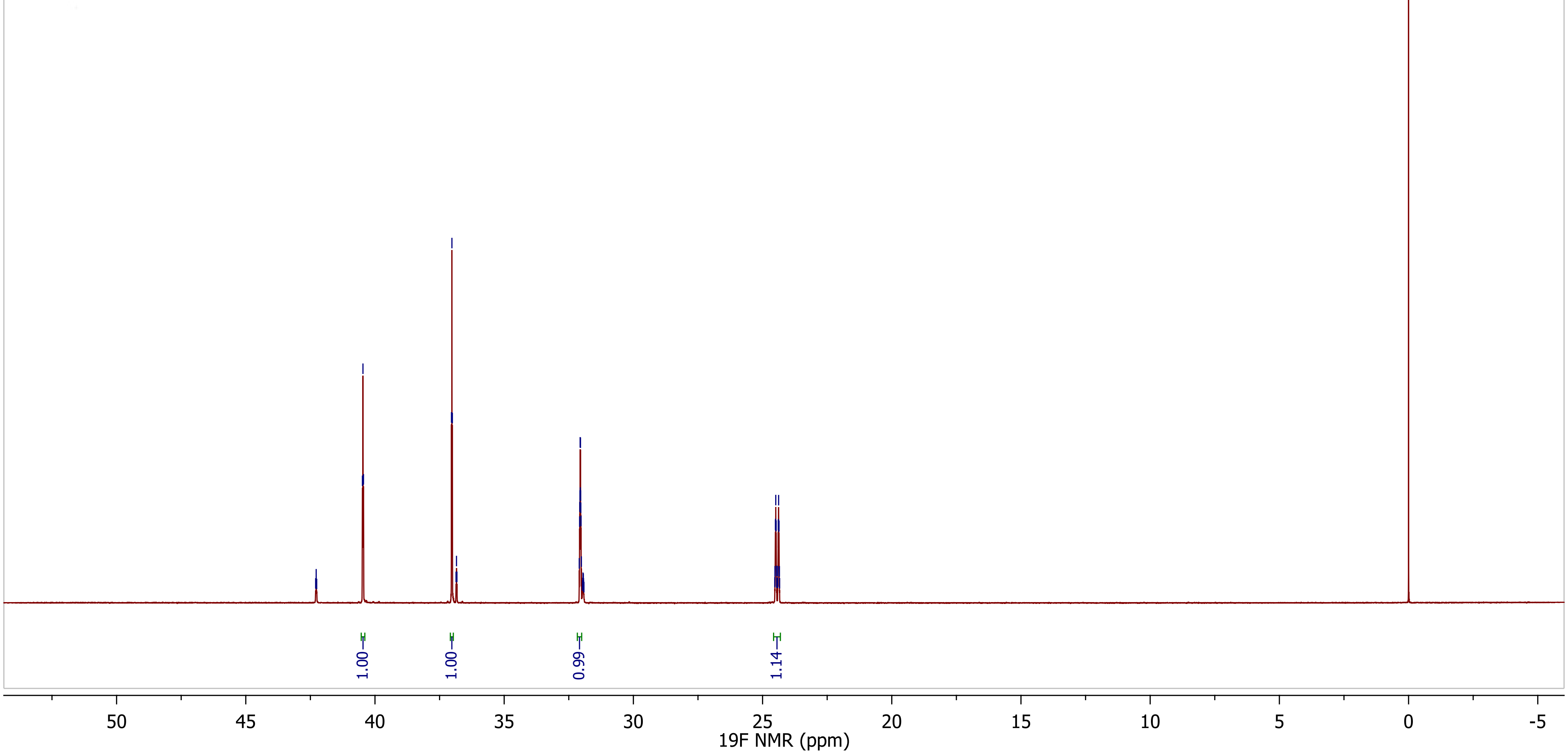

^{1}H NMR (500 MHz, $CDCl_3$)

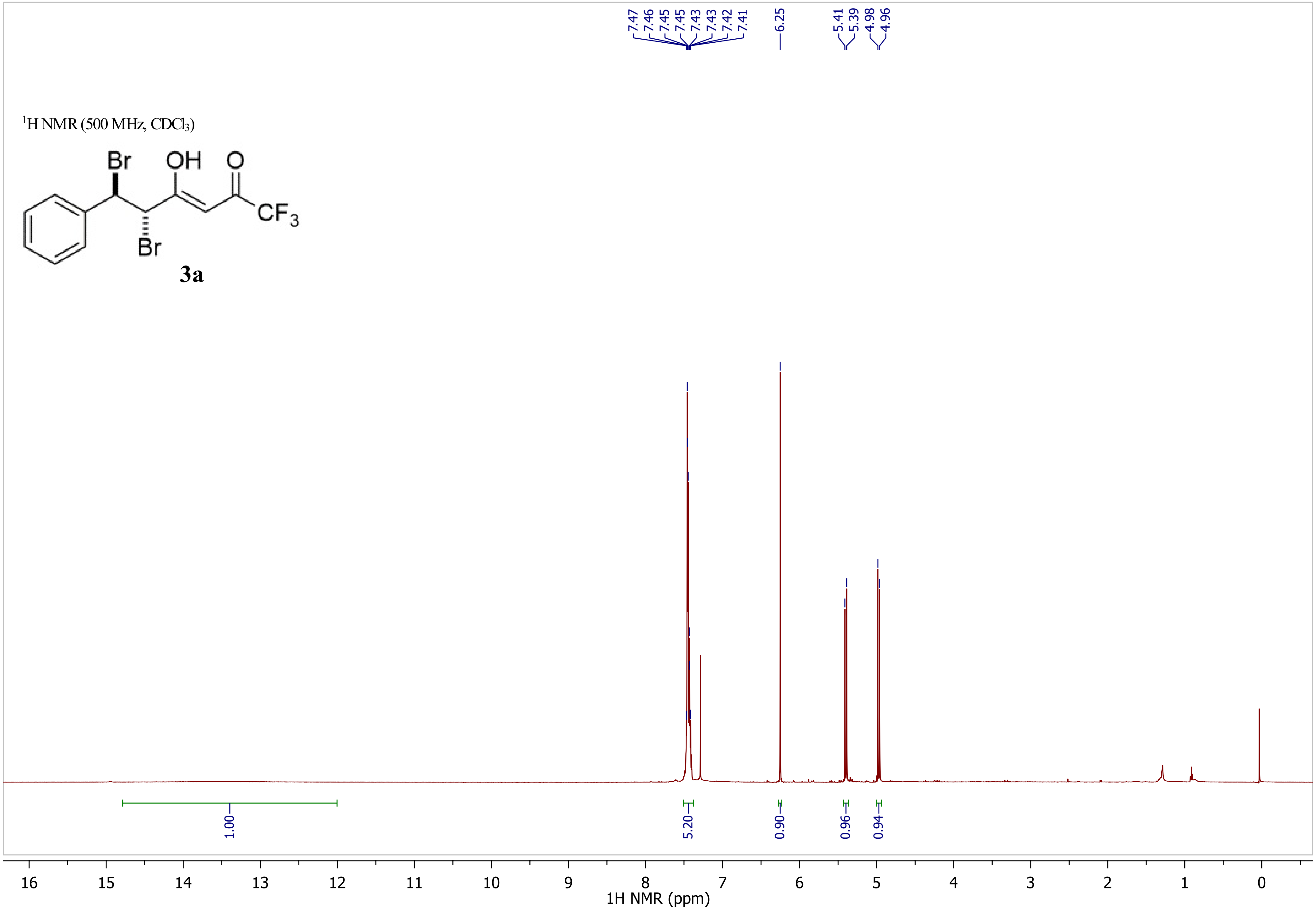

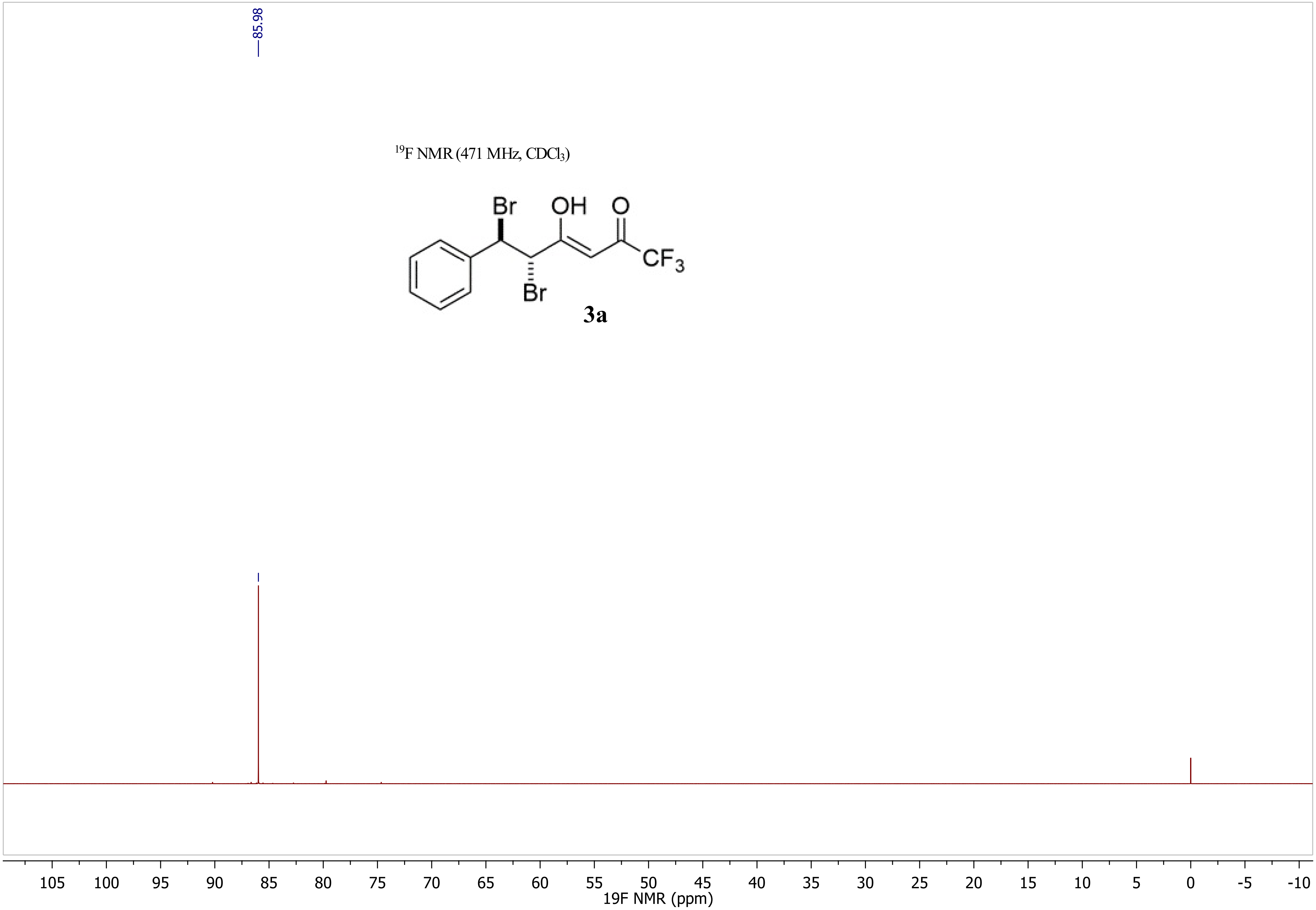

85.98
^{19}F NMR (471 MHz, $CDCl_3$)
Br
OH
O
CF3
Br
105
100
95
90
85
80
75
70
65
60
55
50
45
40
35
30
25
20
15
10
5
0
-5
-10
19F NMR (ppm)

3a

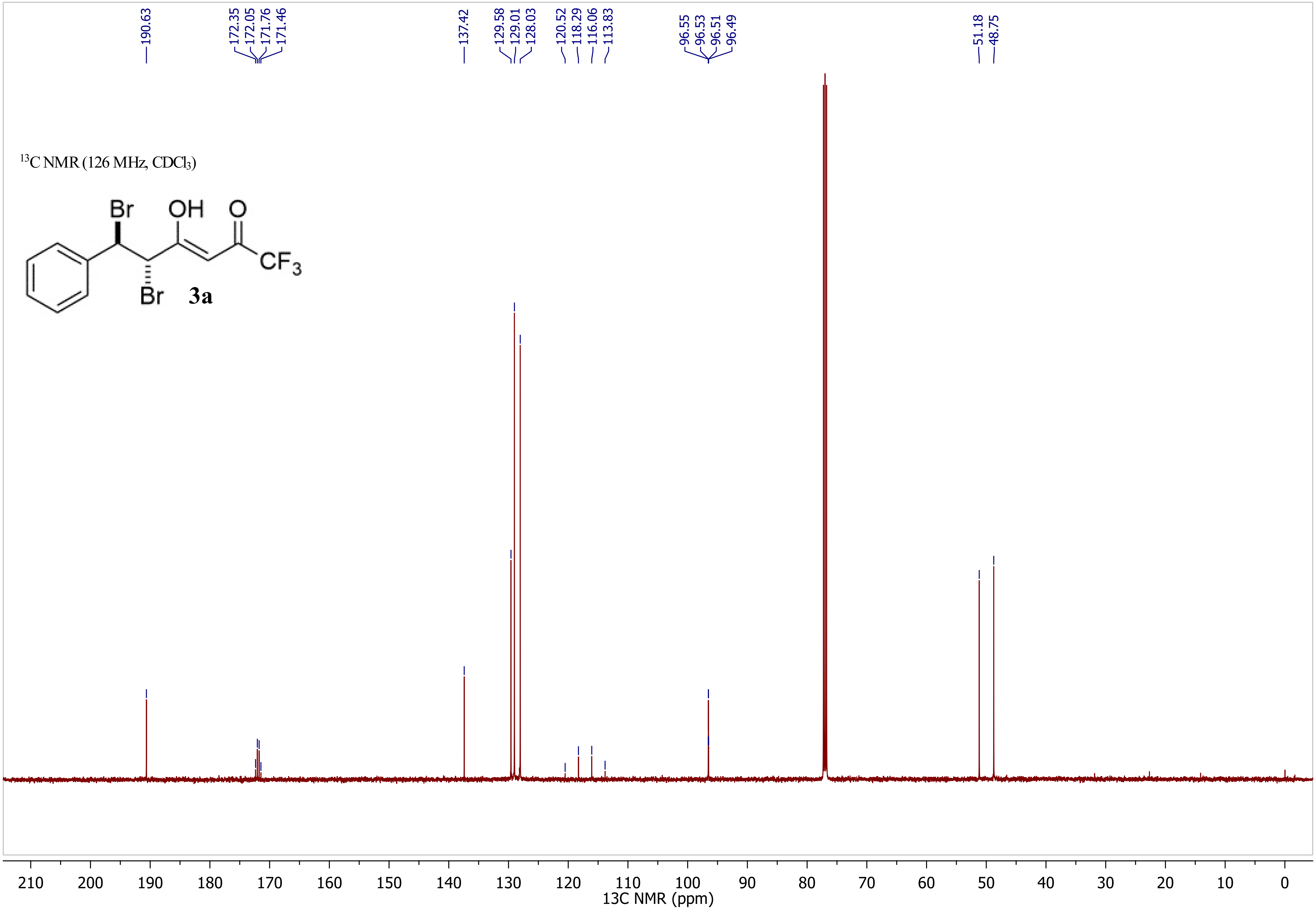
190.63
172.35
172.05
171.76
171.46
137.42
129.58
129.01
128.03
120.52
118.29
116.06
113.83
96.55
96.53
96.51
96.49
51.18
48.75
^{13}C NMR (126 MHz, CDCl$_3$)
Br
OH
O
CF3
Br
3a
210
200
190
180
170
160
150
140
130
120
110
100
90
80
70
60
50
40
30
20
10
0
13C NMR (ppm)

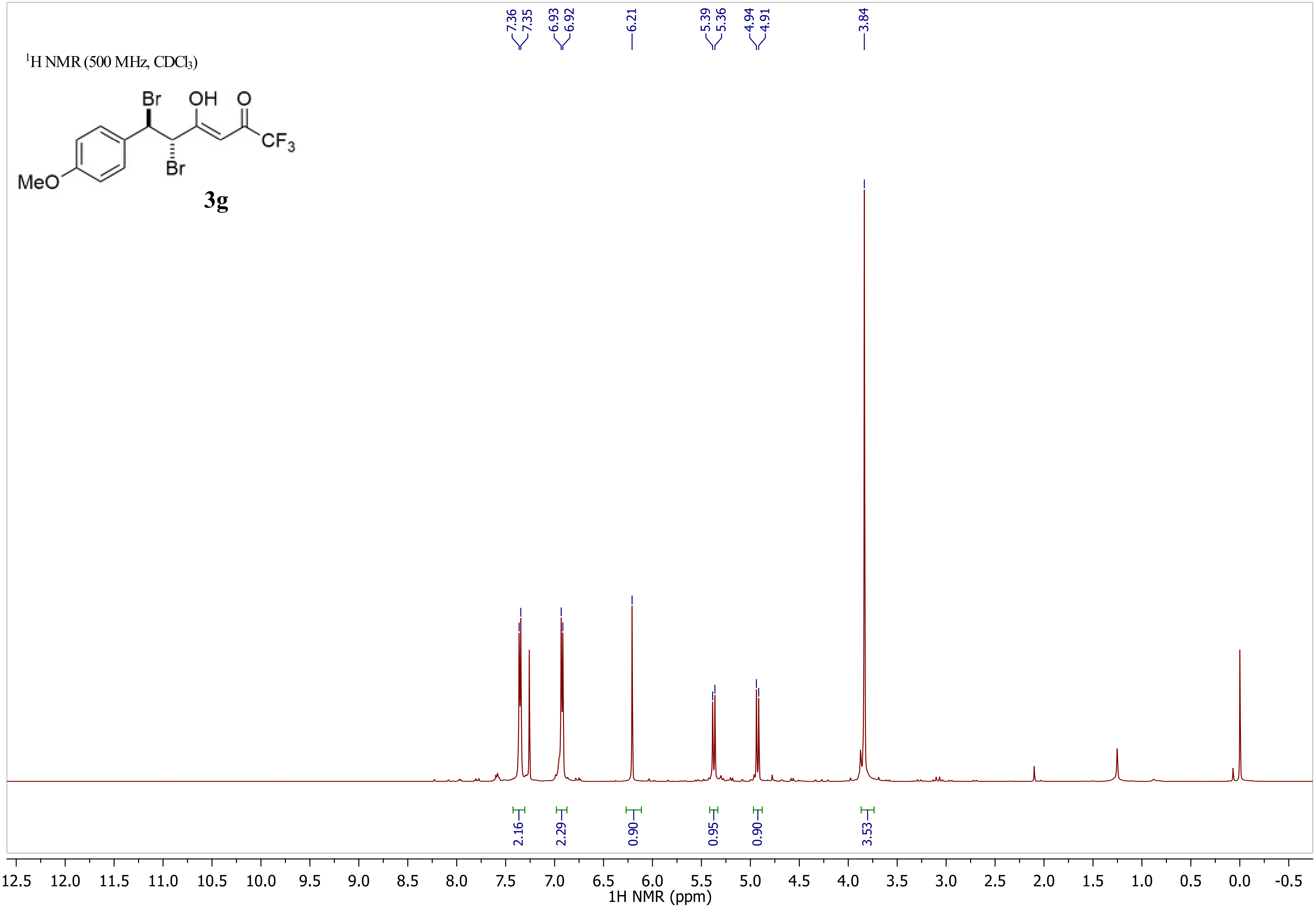

^{1}H NMR (500 MHz, $CDCl_3$)
Br
OH
O
CF_3
MeO
Br
3g
7.36
7.35
6.93
6.92
6.21
5.39
5.36
4.94
4.91
3.84
2.16
2.29
0.90
0.95
0.90
3.53
12.5 12.0 11.5 11.0 10.5 10.0 9.5 9.0 8.5 8.0 7.5 7.0 6.5 6.0 5.5 5.0 4.5 4.0 3.5 3.0 2.5 2.0 1.5 1.0 0.5 0.0 -0.5
1H NMR (ppm)

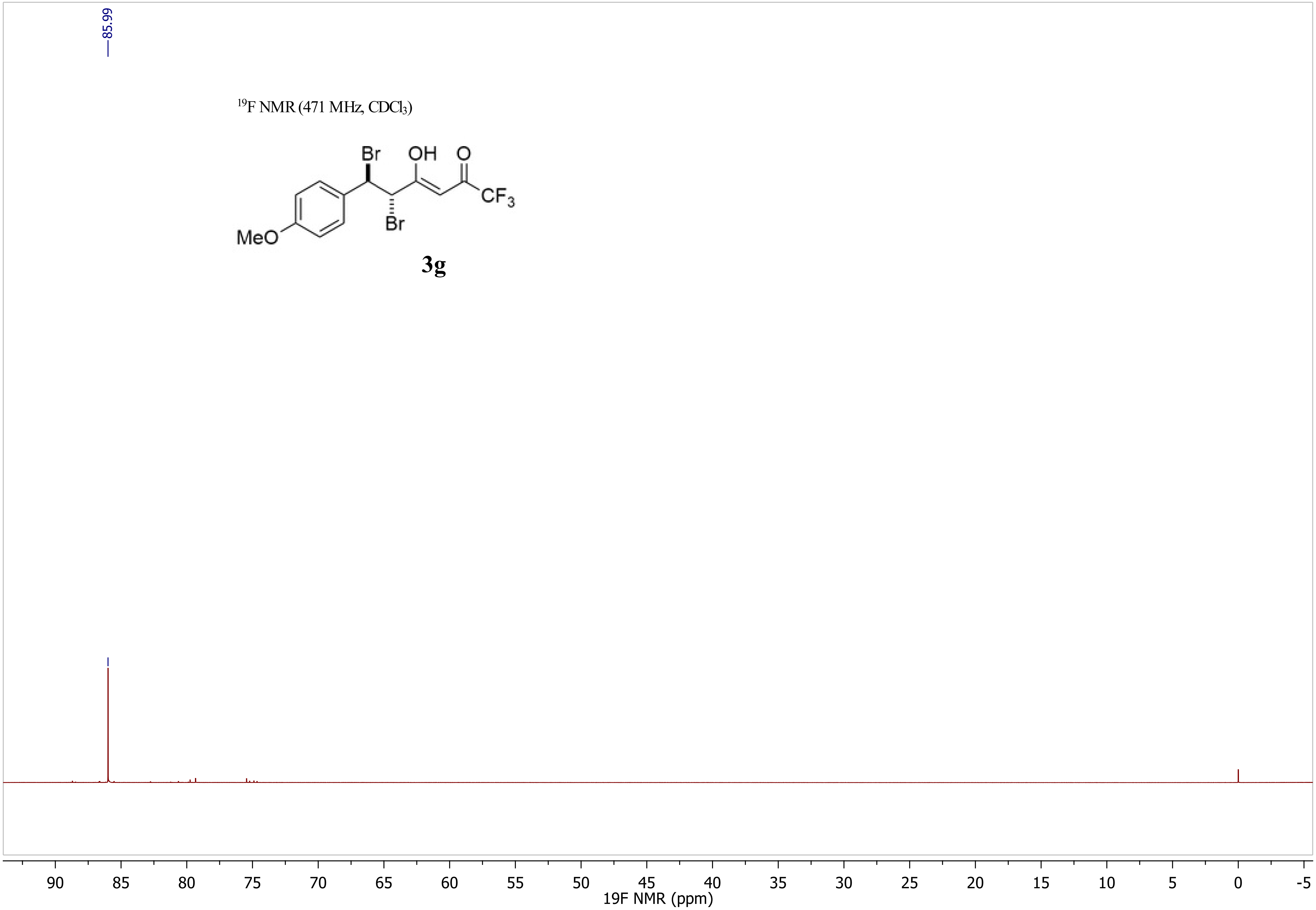

85.99
19F NMR (471 MHz, CDCl3)
Br
OH
O
CF3
MeO
Br
3g
90 85 80 75 70 65 60 55 50 45 40 35 30 25 20 15 10 5 0 -5
19F NMR (ppm)

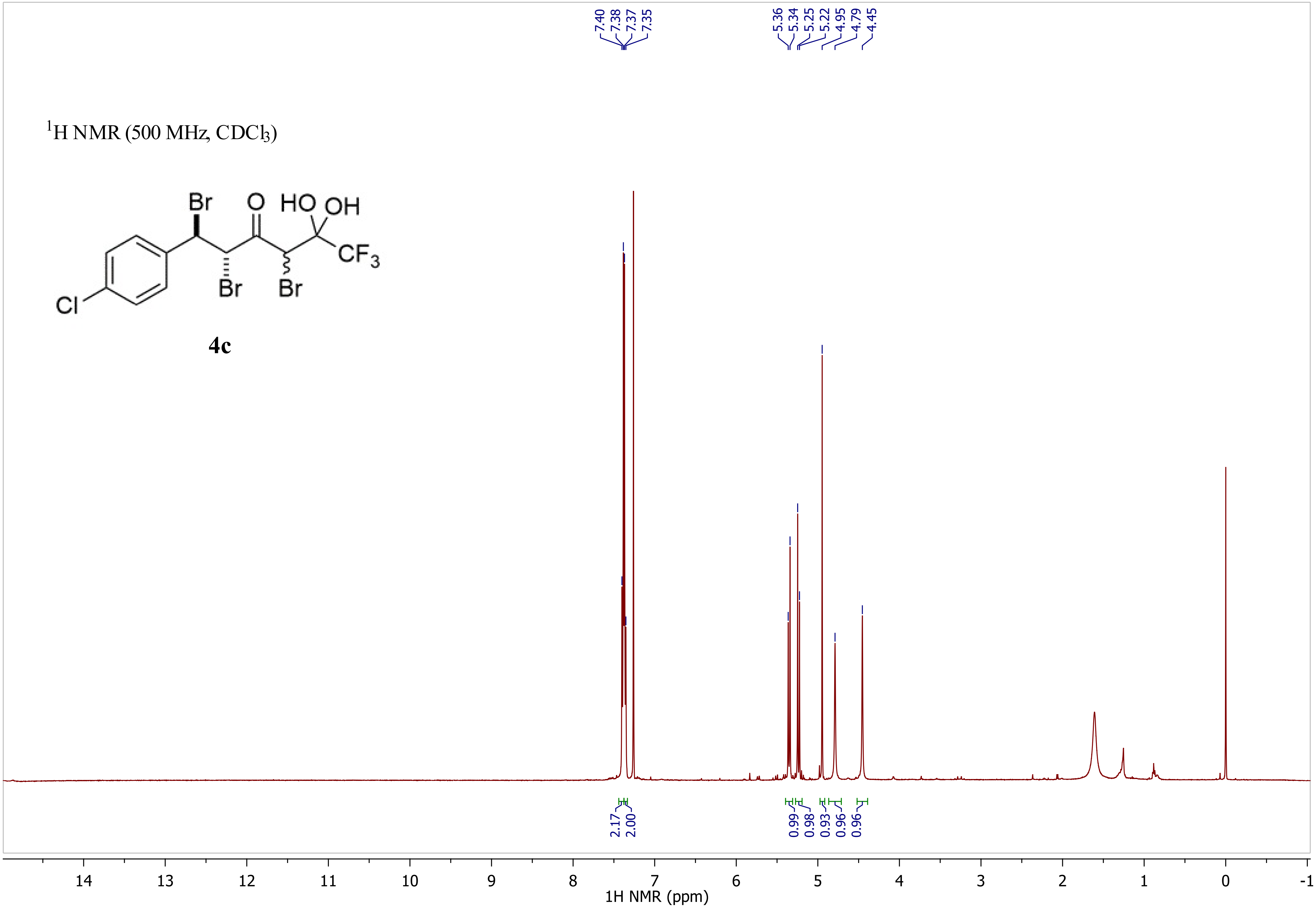

^{1}H NMR (500 MHz, CDCl$_3$)
4c
Br
O
HO
OH
CF$_3$
Cl
Br
Br
7.40
7.38
7.37
7.35
5.36
5.34
5.25
5.22
4.95
4.79
4.45
2.17
2.00
0.99
0.98
0.93
0.96
0.96
14 13 12 11 10 9 8 7 6 5 4 3 2 1 0 -1
1H NMR (ppm)

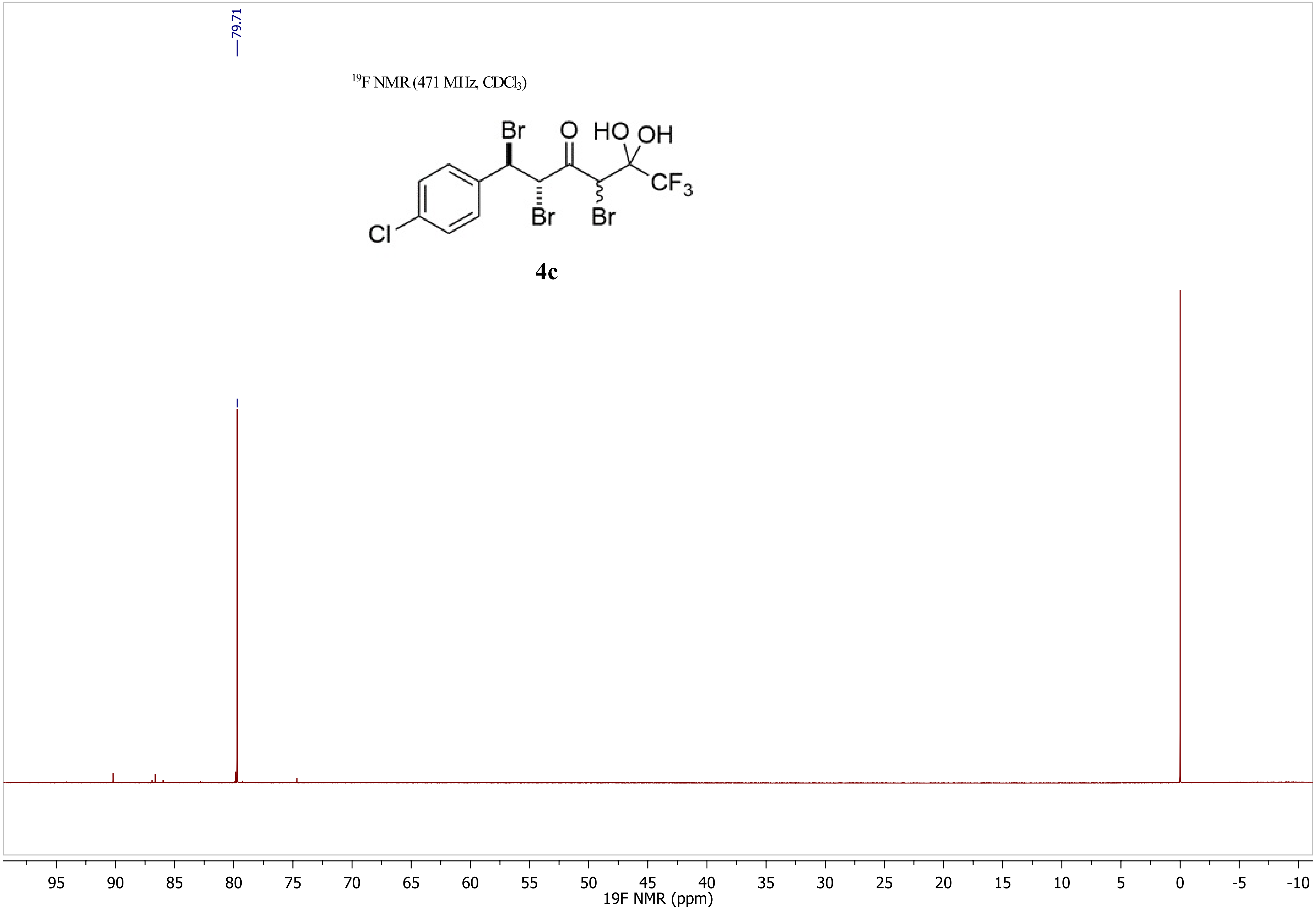
79.71
19F NMR (471 MHz, CDCl3)
Br
O
HO
OH
CF3
Br
Br
Cl
4c
95
90
85
80
75
70
65
60
55
50
45
40
35
30
25
20
15
10
5
0
-5
-10
19F NMR (ppm)

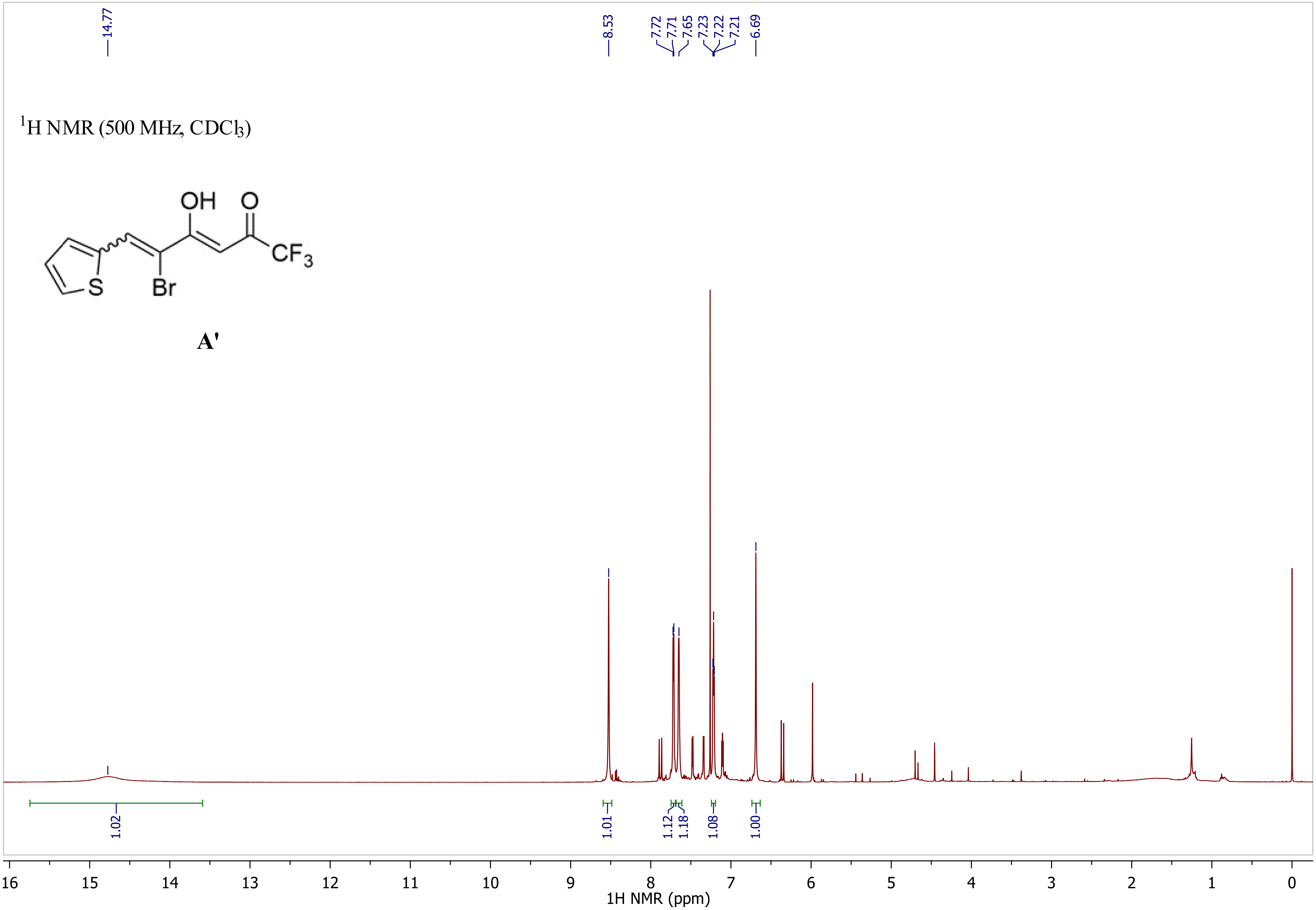

14.77
8.53
7.72
7.71
7.65
7.23
7.22
7.21
6.69
1H NMR (500 MHz, CDCl3)
OH
O
CF3
S
Br
A'
1.02
1.01
1.12
1.18
1.08
1.00
16 15 14 13 12 11 10 9 8 7 6 5 4 3 2 1 0
1H NMR (ppm)

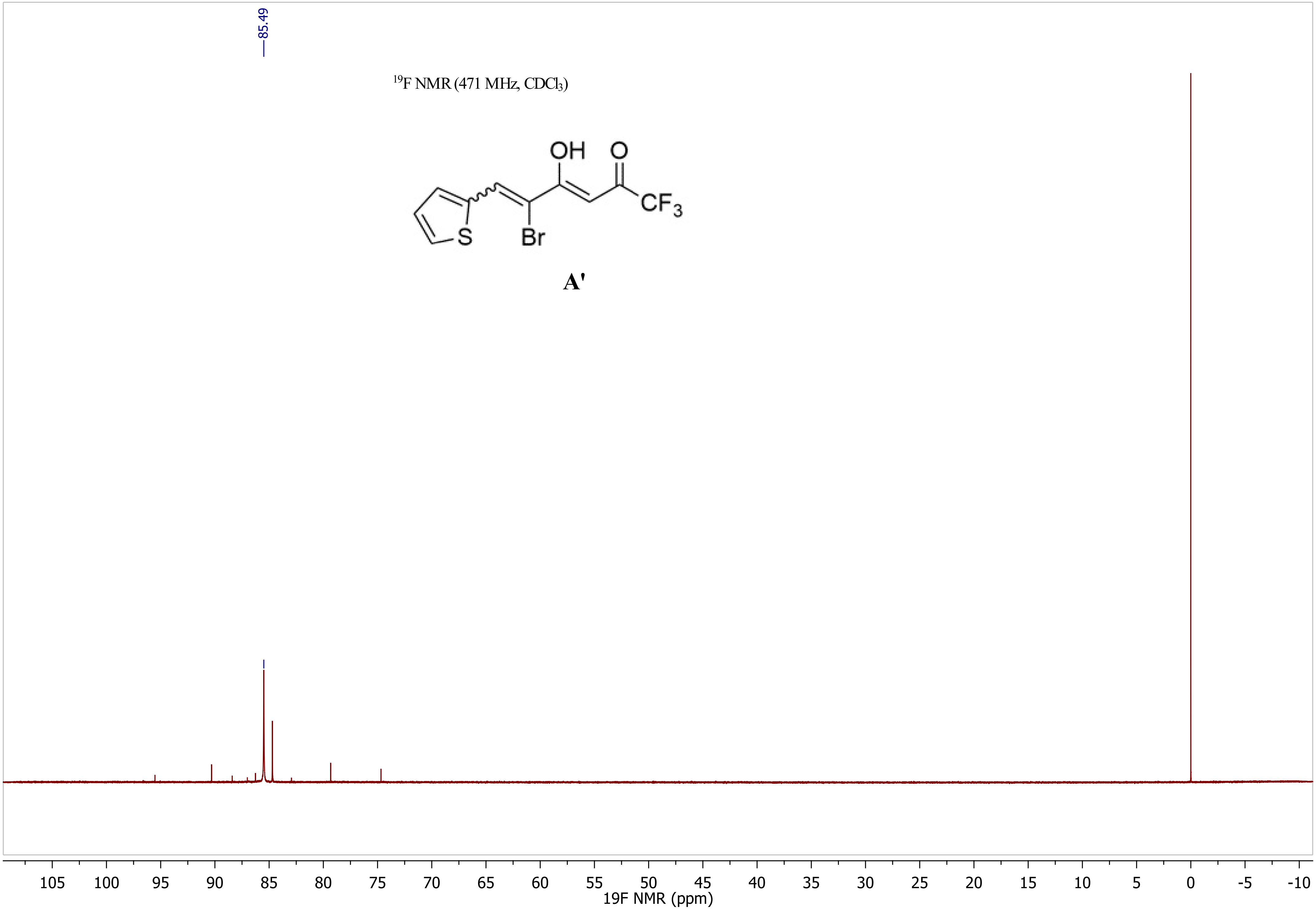
85.49
19F NMR (471 MHz, CDCl3)
OH
O
CF3
S
Br
A'
105 100 95 90 85 80 75 70 65 60 55 50 45 40 35 30 25 20 15 10 5 0 -5 -10
19F NMR (ppm)

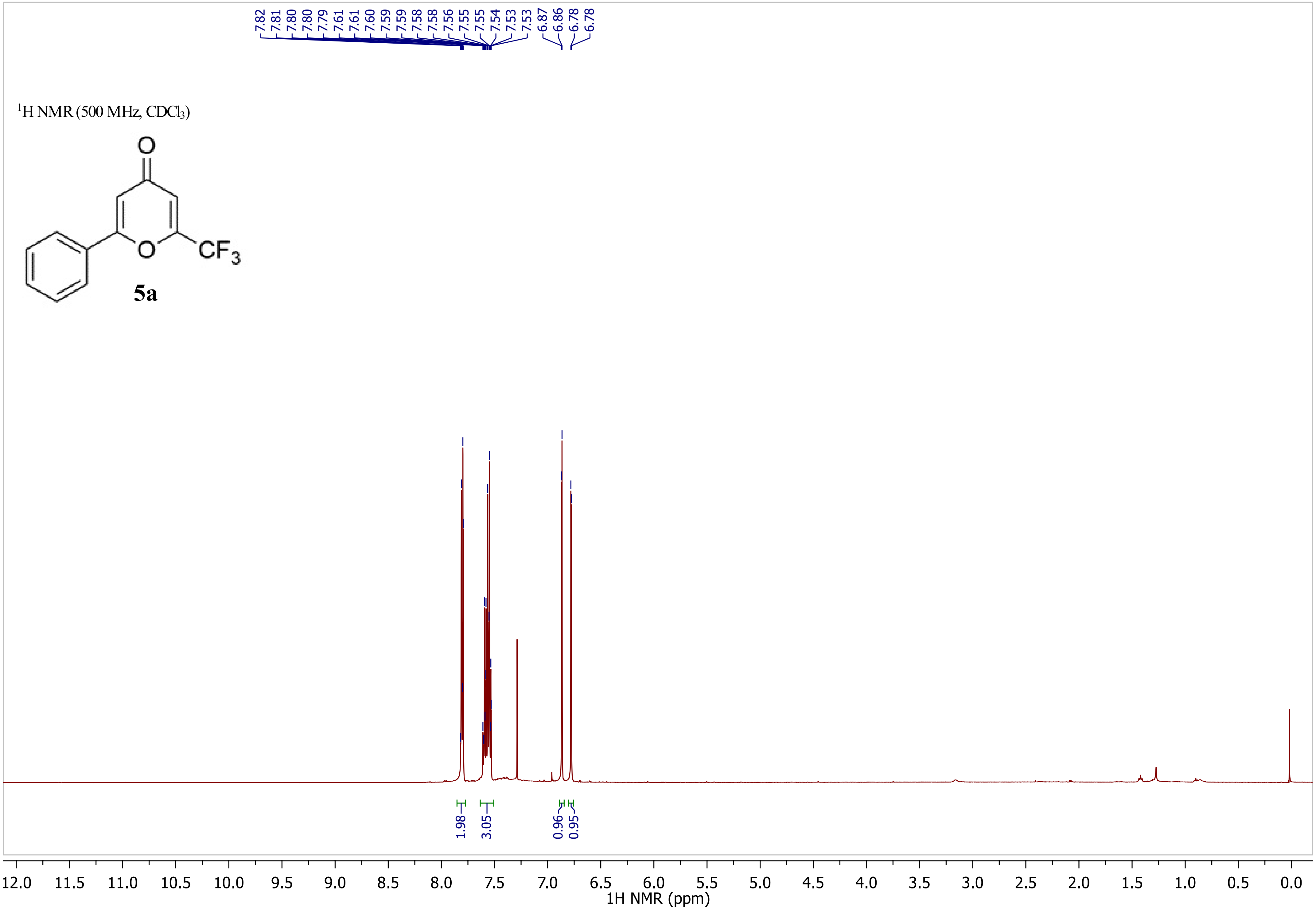

7.82 7.81 7.80 7.80 7.79 7.61 7.61 7.60 7.59 7.59 7.58 7.58 7.56 7.55 7.55 7.54 7.53 7.53 6.87 6.86 6.78 6.78
^{1}H NMR (500 MHz, $CDCl_3$)
O
O
CF_3
5a
1.98
3.05
0.96
0.95
12.0 11.5 11.0 10.5 10.0 9.5 9.0 8.5 8.0 7.5 7.0 6.5 6.0 5.5 5.0 4.5 4.0 3.5 3.0 2.5 2.0 1.5 1.0 0.5 0.0
1H NMR (ppm)

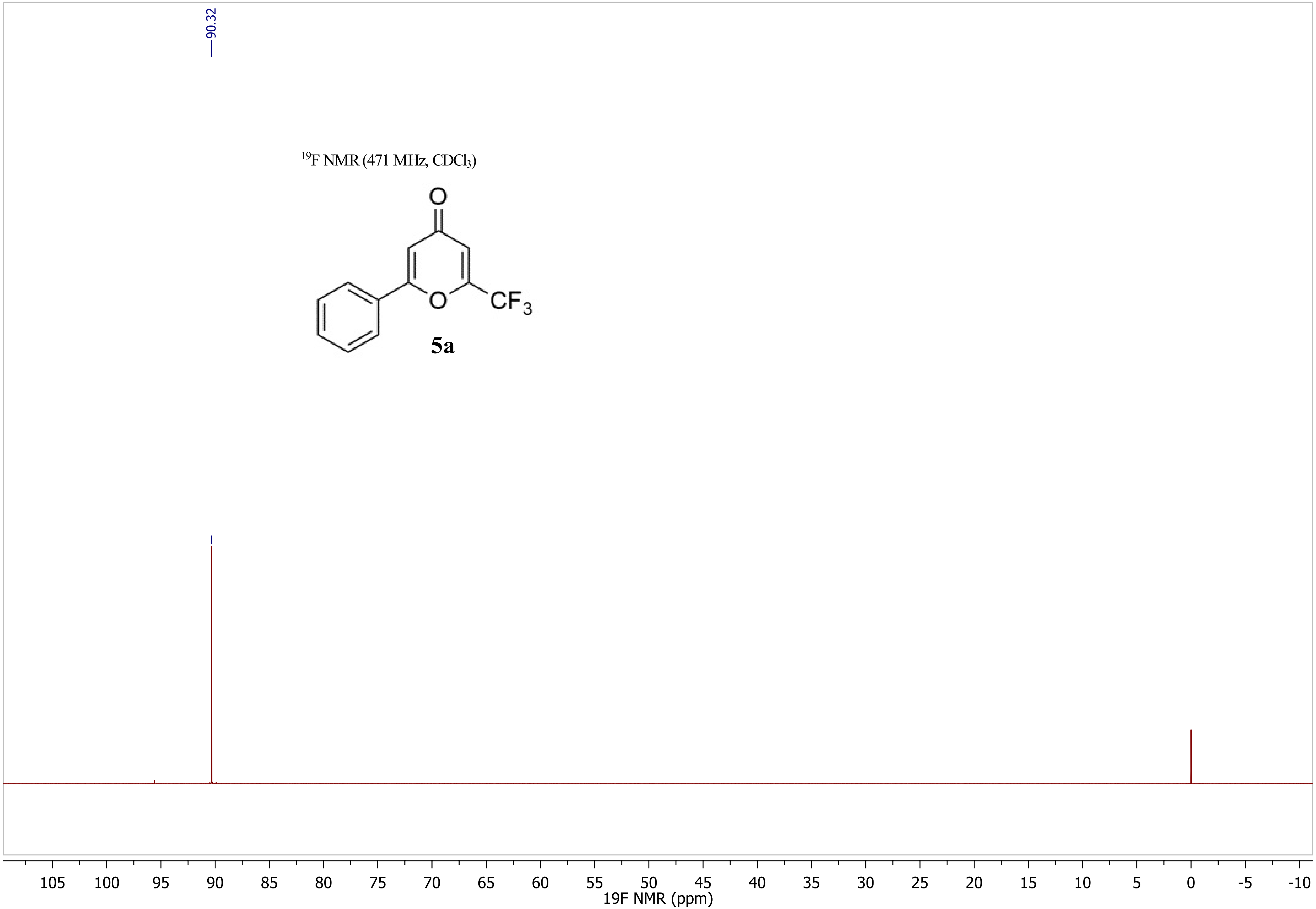
-90.32
19F NMR (471 MHz, CDCl3)
O
O
CF3
5a
105 100 95 90 85 80 75 70 65 60 55 50 45 40 35 30 25 20 15 10 5 0 -5 -10
19F NMR (ppm)

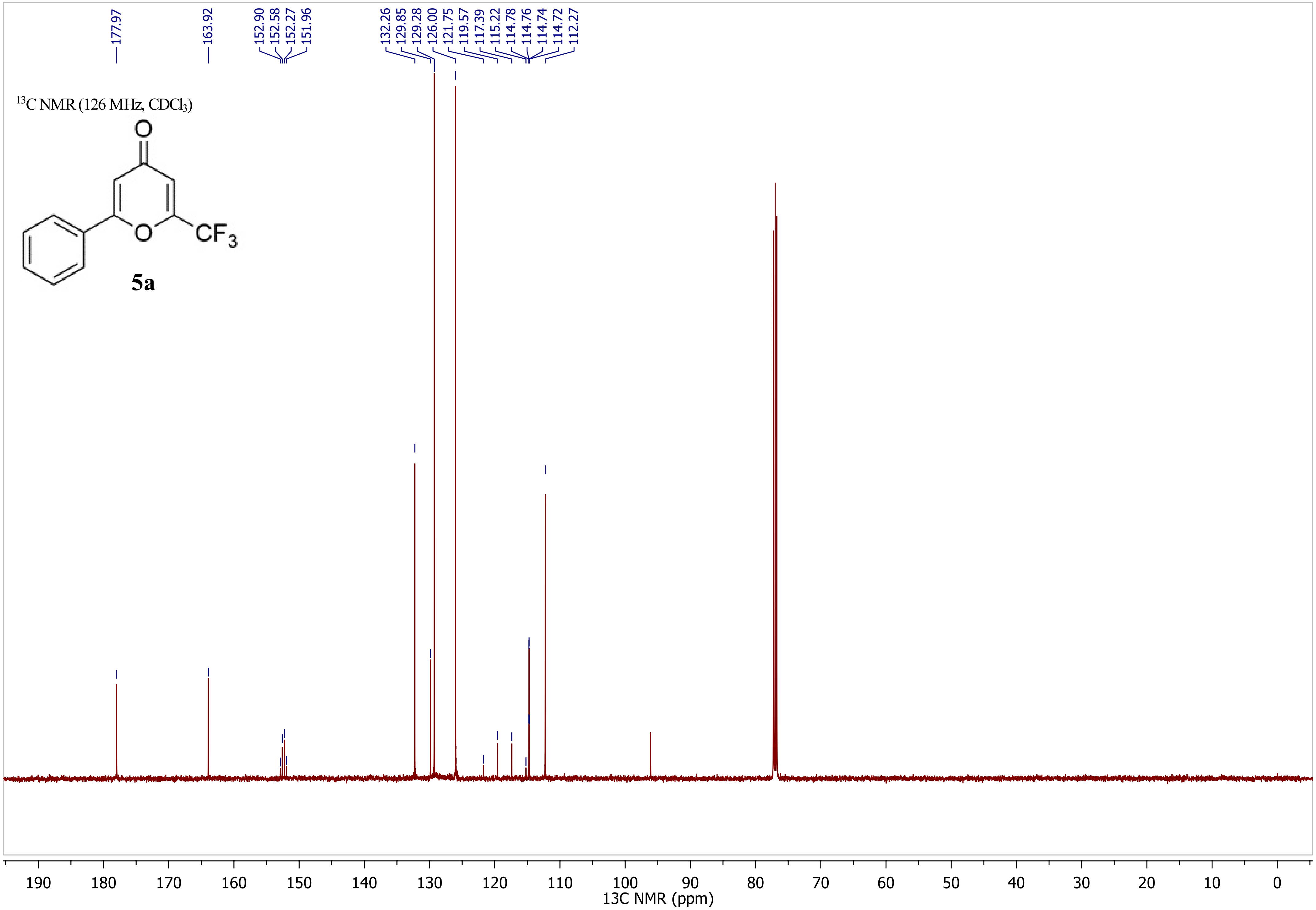
177.97
163.92
152.90
152.58
152.27
151.96
132.26
129.85
129.28
126.00
121.75
119.57
117.39
115.22
114.78
114.76
114.74
114.72
112.27
^{13}C NMR (126 MHz, CDCl$_3$)
O
O
CF$_3$
5a
190 180 170 160 150 140 130 120 110 100 90 80 70 60 50 40 30 20 10 0
13C NMR (ppm)

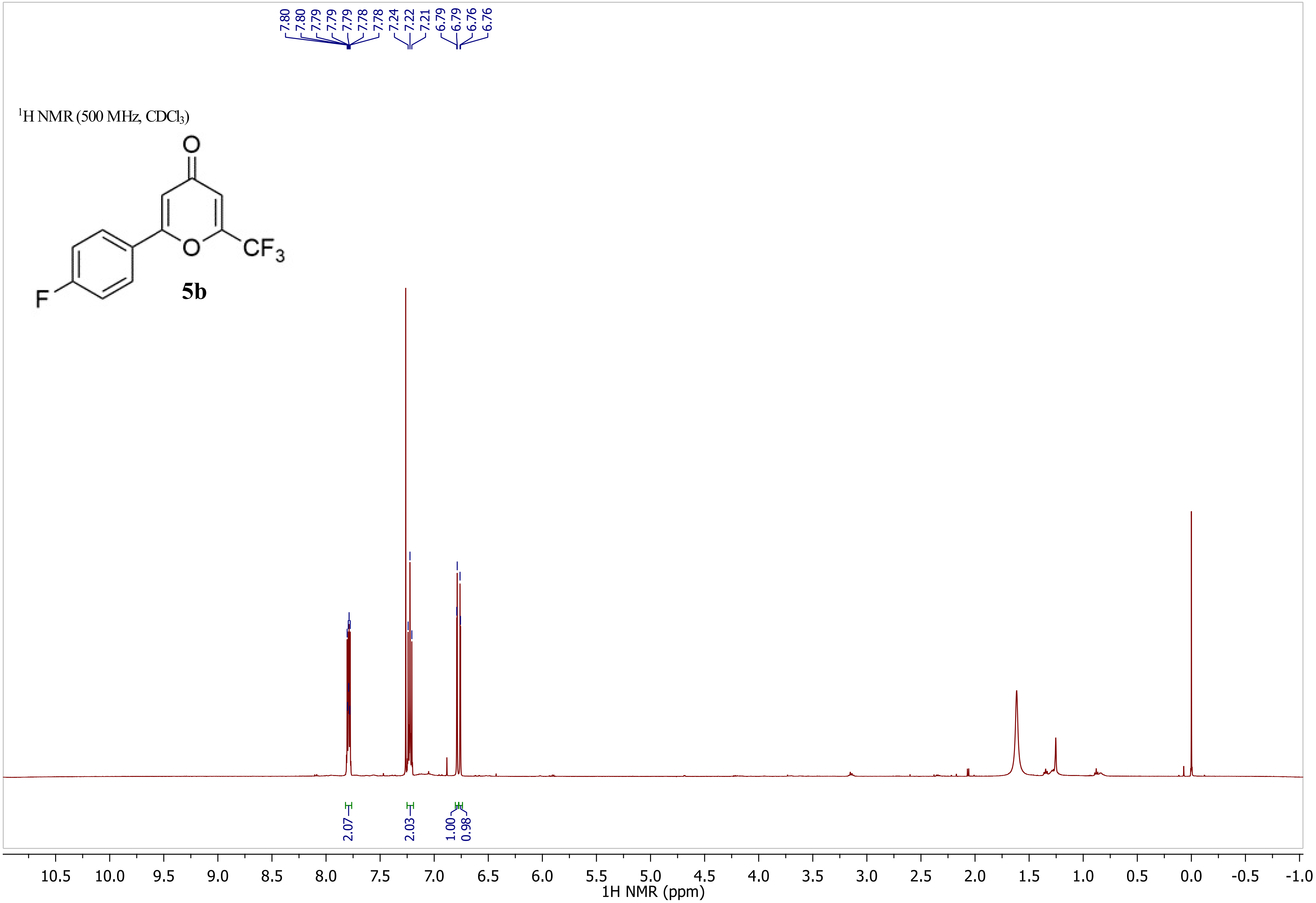

1H NMR (500 MHz, CDCl3)
5b
O
O
CF3
F
7.80
7.80
7.79
7.79
7.79
7.78
7.78
7.24
7.22
7.21
6.79
6.79
6.76
6.76
2.07
2.03
1.00
0.98
10.5 10.0 9.5 9.0 8.5 8.0 7.5 7.0 6.5 6.0 5.5 5.0 4.5 4.0 3.5 3.0 2.5 2.0 1.5 1.0 0.5 0.0 -0.5 -1.0
1H NMR (ppm)

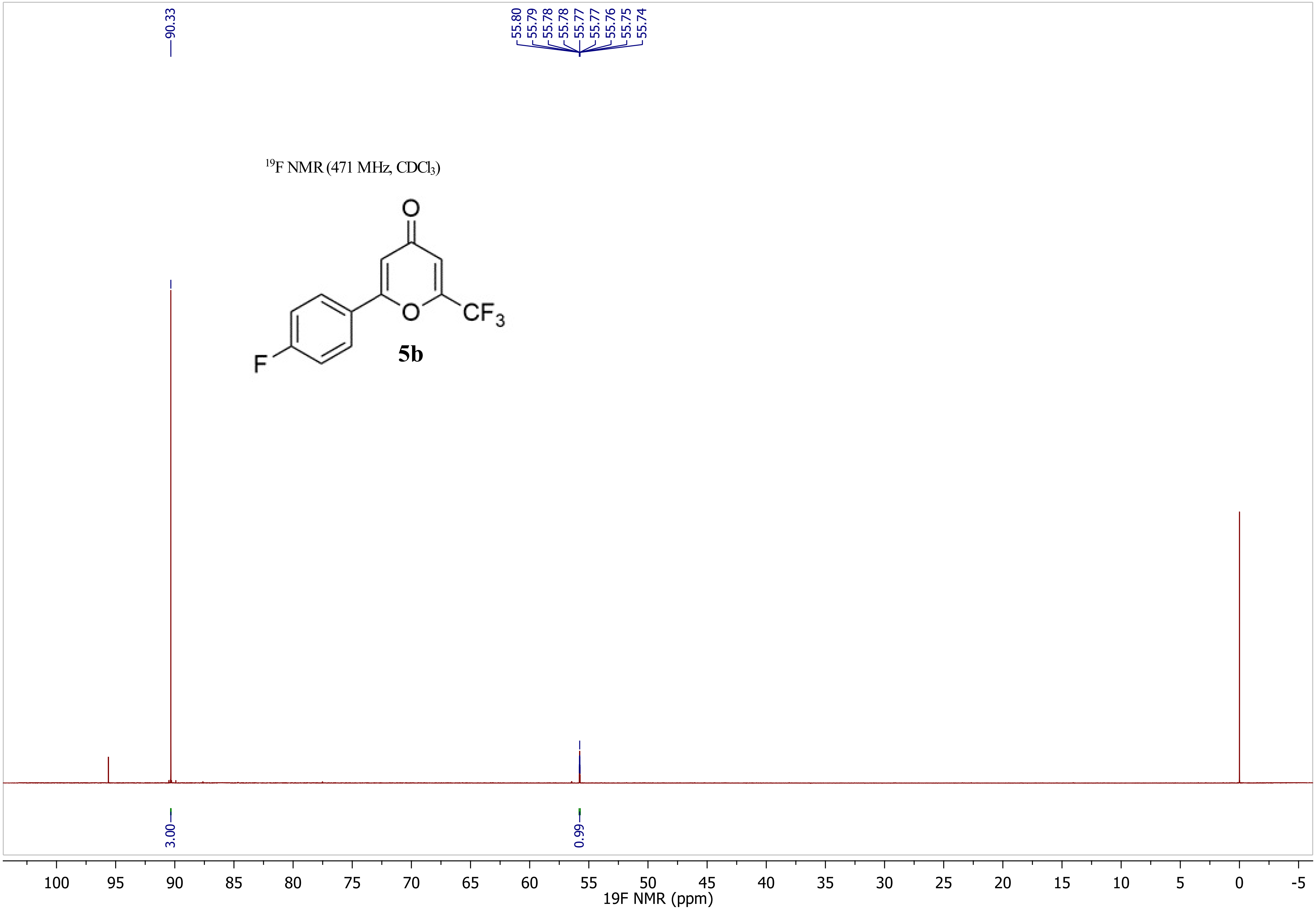

90.33
55.80
55.79
55.78
55.78
55.77
55.77
55.76
55.75
55.74
19F NMR (471 MHz, CDCl3)
O
O
CF3
F
5b
3.00
0.99
100
95
90
85
80
75
70
65
60
55
50
45
40
35
30
25
20
15
10
5
0
-5
19F NMR (ppm)

^{1}H NMR (400 MHz, $CDCl_3$)

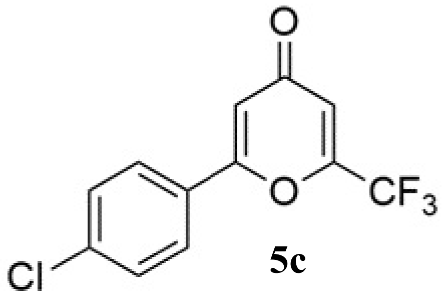

5c

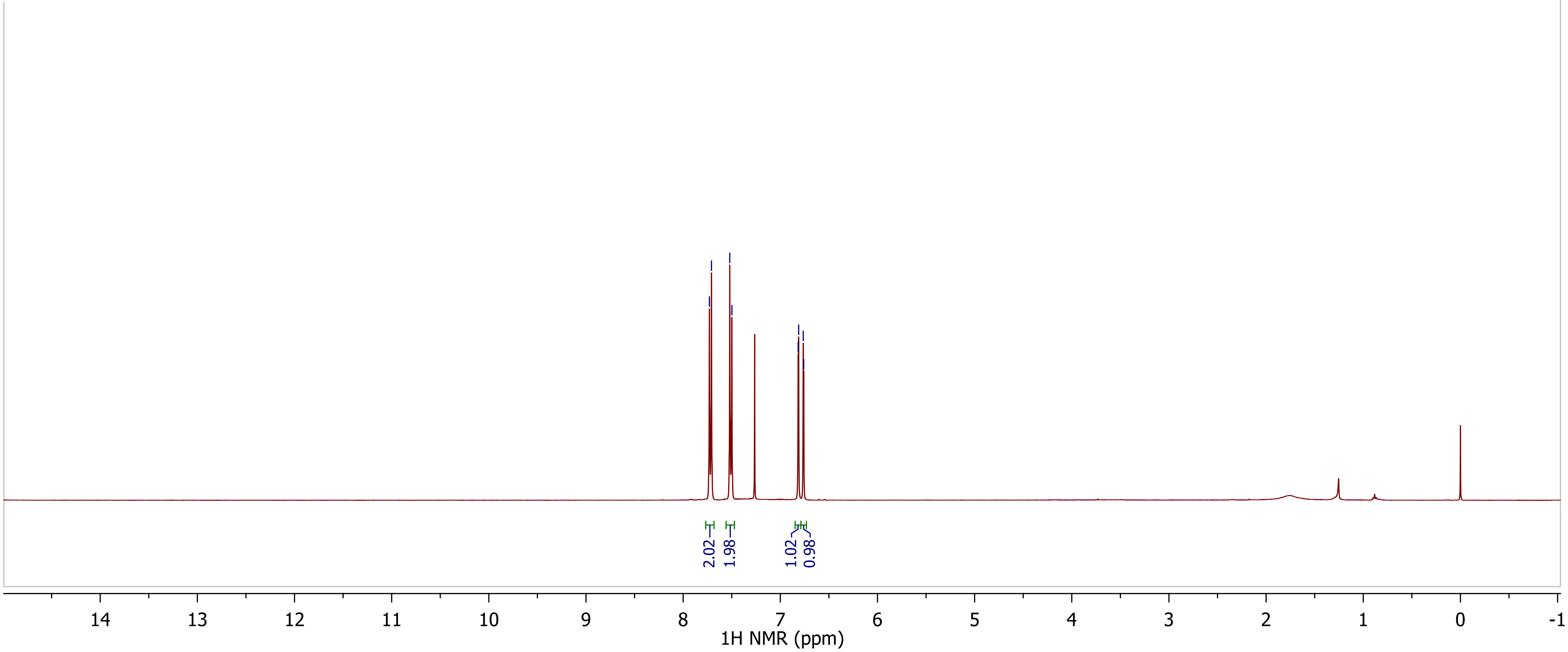

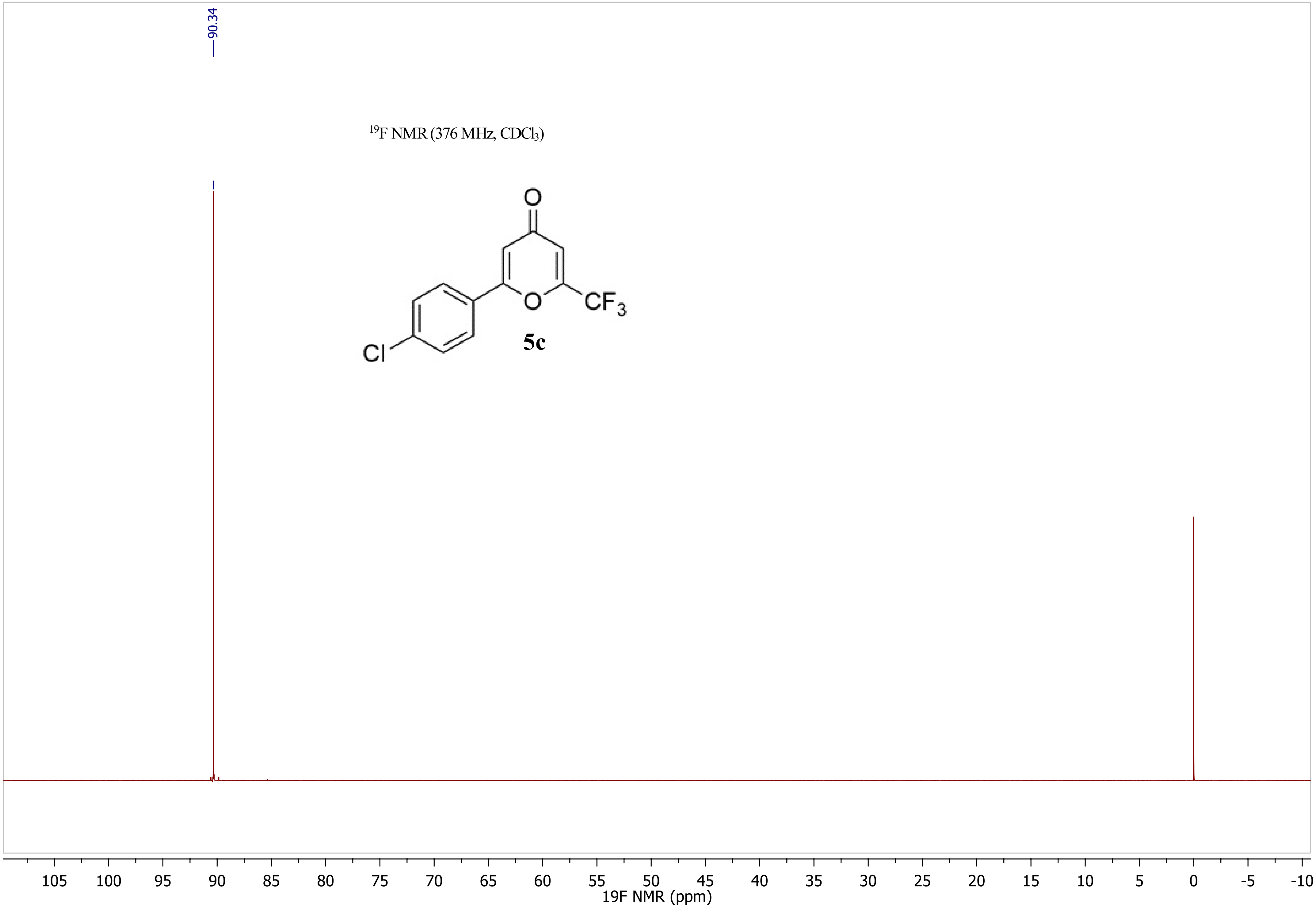

90.34
^{19}F NMR (376 MHz, $CDCl_3$)
O
O
CF3
Cl
5c
105 100 95 90 85 80 75 70 65 60 55 50 45 40 35 30 25 20 15 10 5 0 -5 -10
19F NMR (ppm)

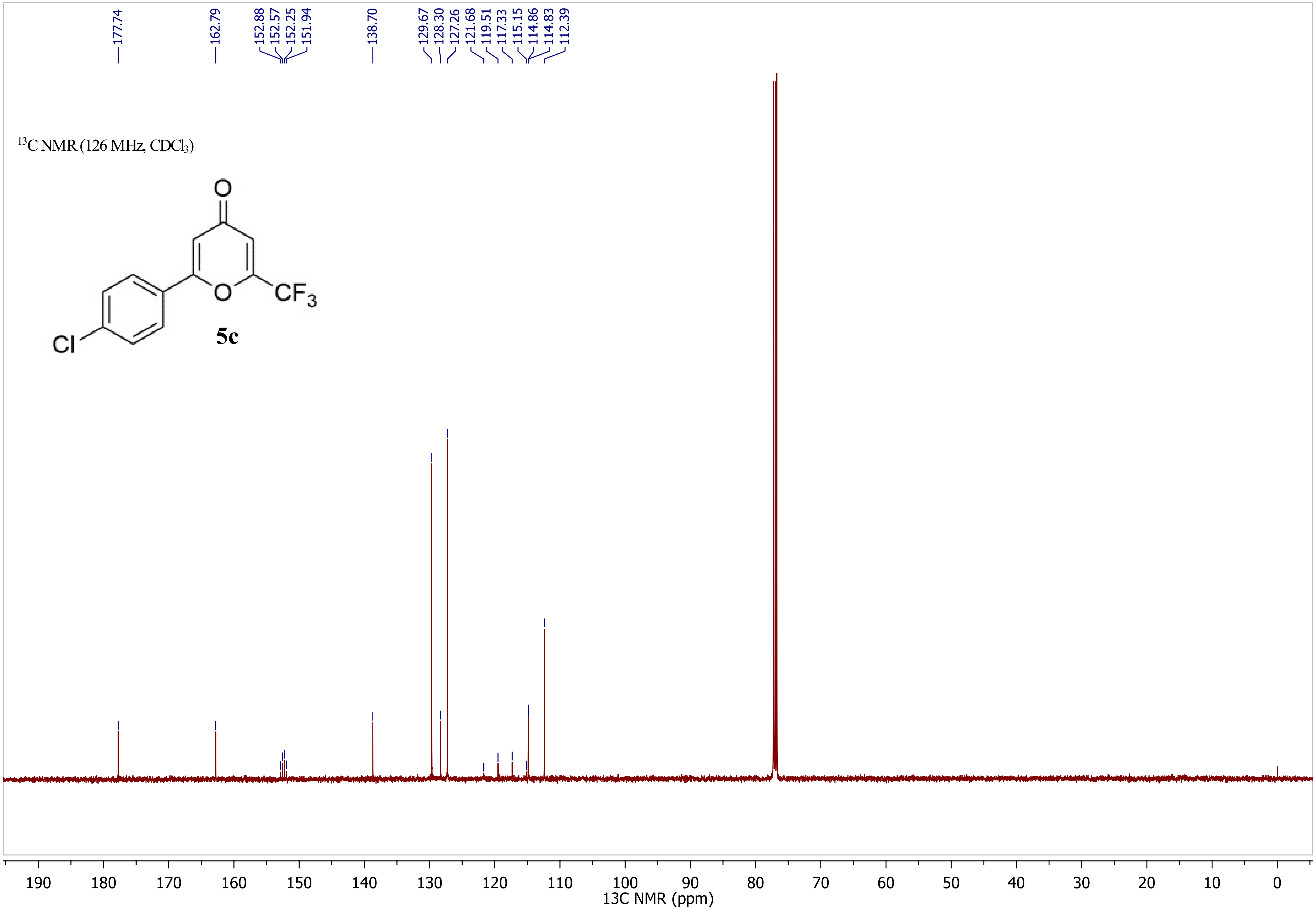

177.74
162.79
152.88
152.57
152.25
151.94
138.70
129.67
128.30
127.26
121.68
119.51
117.33
115.15
114.86
114.83
112.39
13C NMR (126 MHz, CDCl3)
O
O
CF3
Cl
5c
190
180
170
160
150
140
130
120
110
100
90
80
70
60
50
40
30
20
10
0
13C NMR (ppm)

^{1}H NMR (500 MHz, CDCl3)

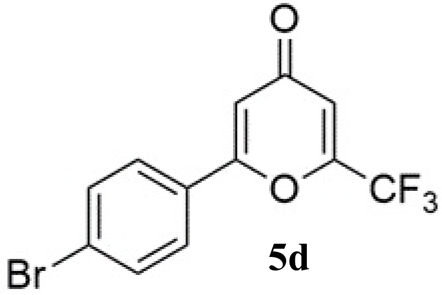

5d

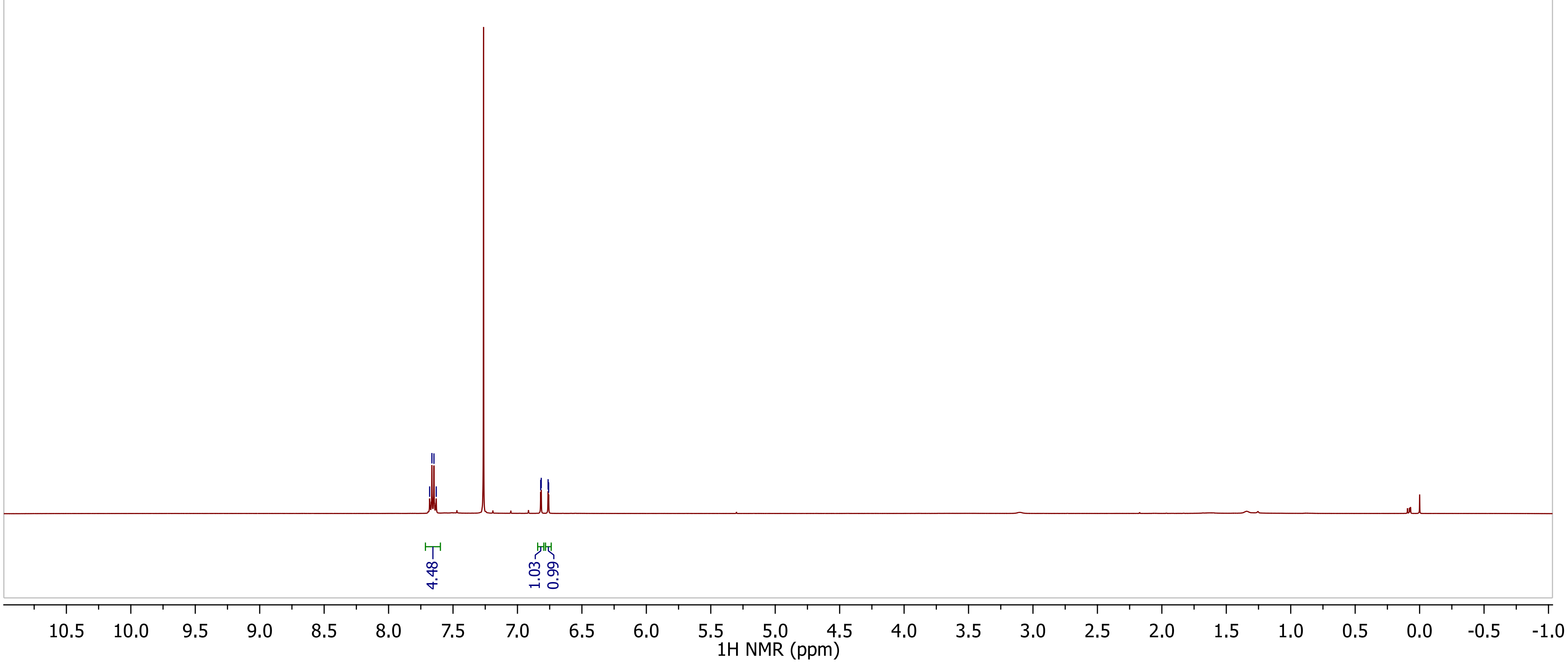

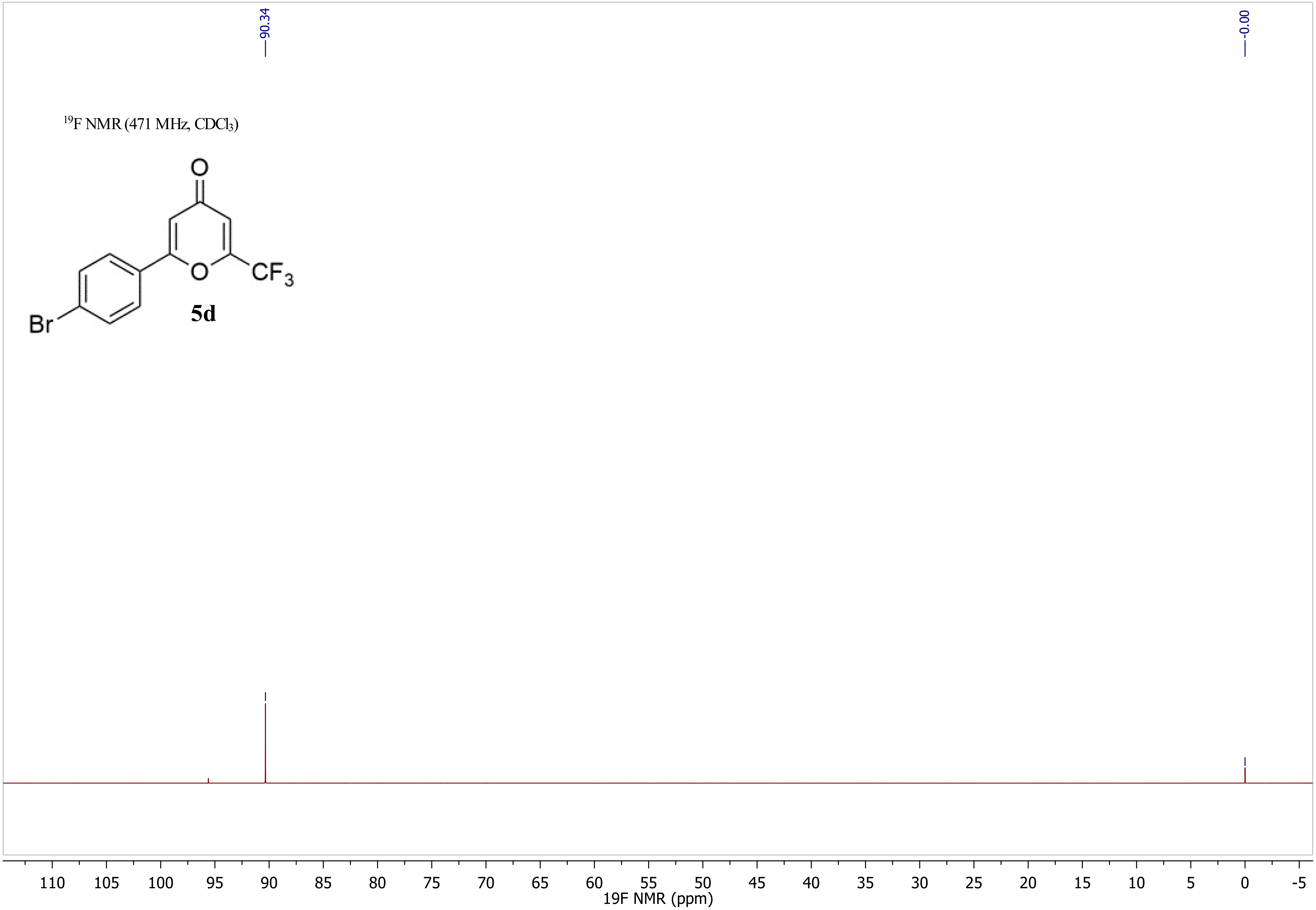

90.34
-0.00
19F NMR (471 MHz, CDCl3)
Br
O
CF3
5d
110 105 100 95 90 85 80 75 70 65 60 55 50 45 40 35 30 25 20 15 10 5 0 -5
19F NMR (ppm)

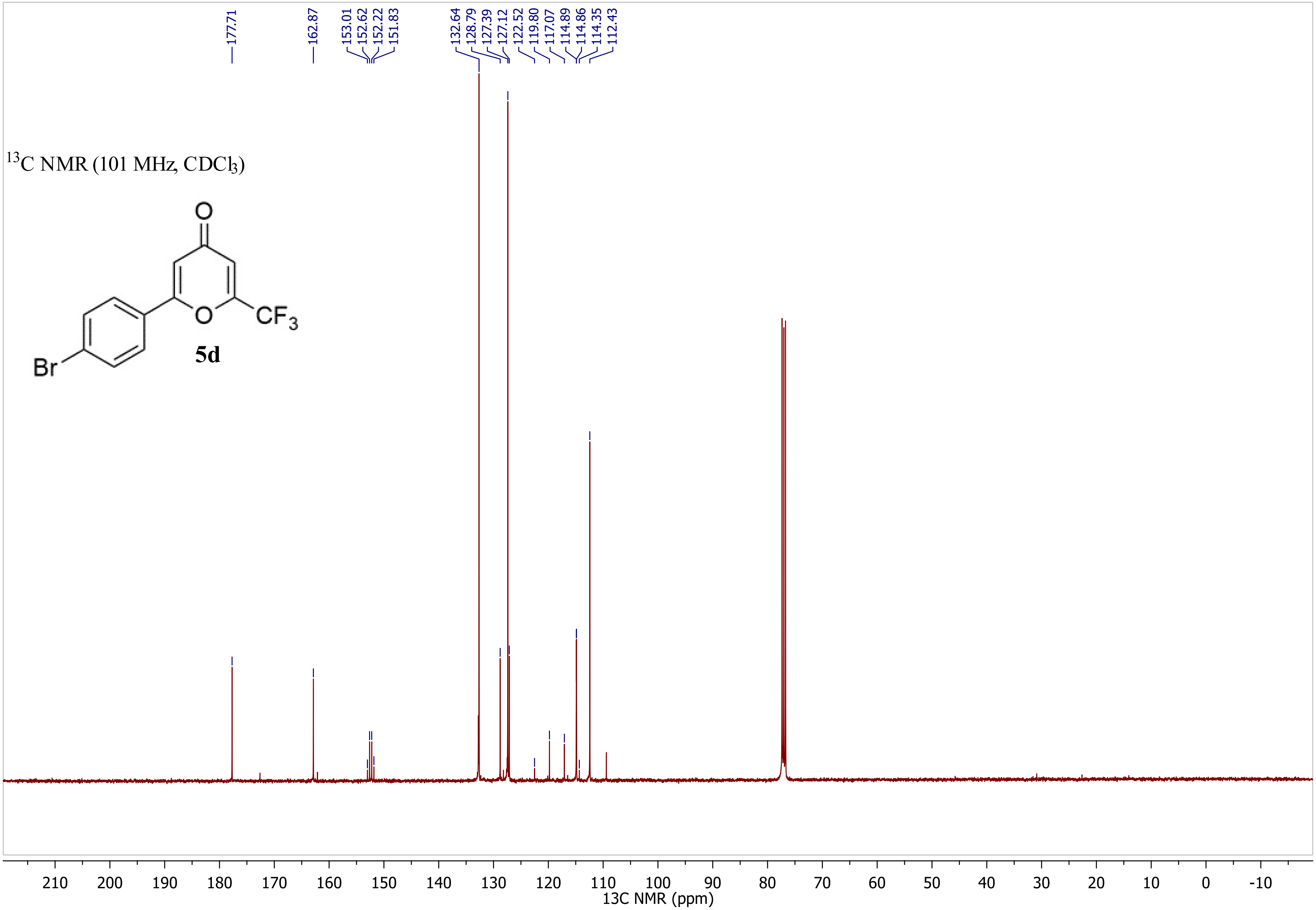
177.71
162.87
153.01
152.62
152.22
151.83
132.64
128.79
127.39
127.12
122.52
119.80
117.07
114.89
114.86
114.35
112.43
^{13}C NMR (101 MHz, $CDCl_3$)
O
O
CF3
Br
5d
210 200 190 180 170 160 150 140 130 120 110 100 90 80 70 60 50 40 30 20 10 0 -10
13C NMR (ppm)

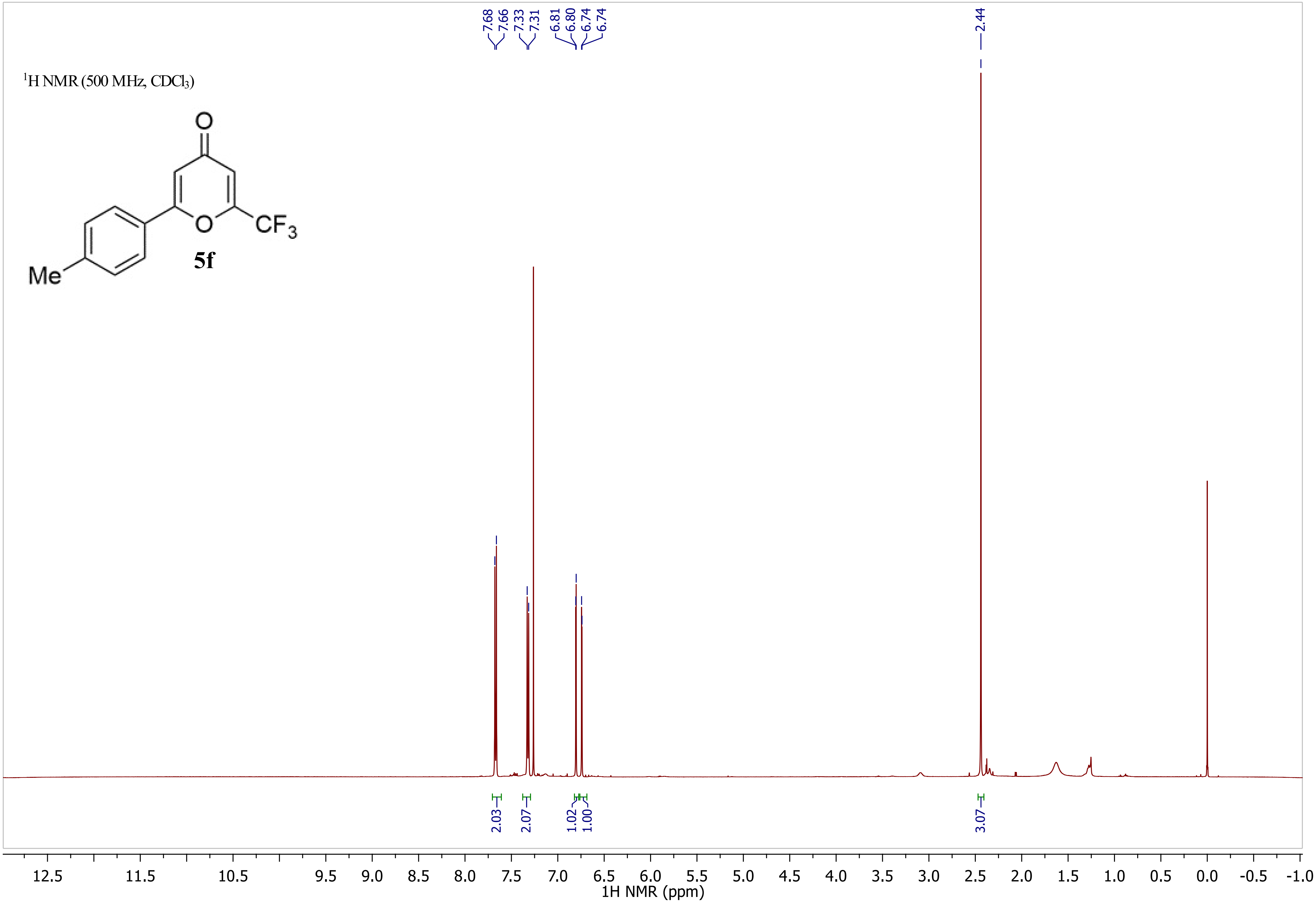

¹H NMR (500 MHz, CDCl₃)
O
O
CF₃
Me
5f
7.68
7.66
7.33
7.31
6.81
6.80
6.74
6.74
2.44
2.03
2.07
1.02
1.00
3.07
12.5
11.5
10.5
9.5
9.0
8.5
8.0
7.5
7.0
6.5
6.0
5.5
5.0
4.5
4.0
3.5
3.0
2.5
2.0
1.5
1.0
0.5
0.0
-0.5
-1.0
1H NMR (ppm)

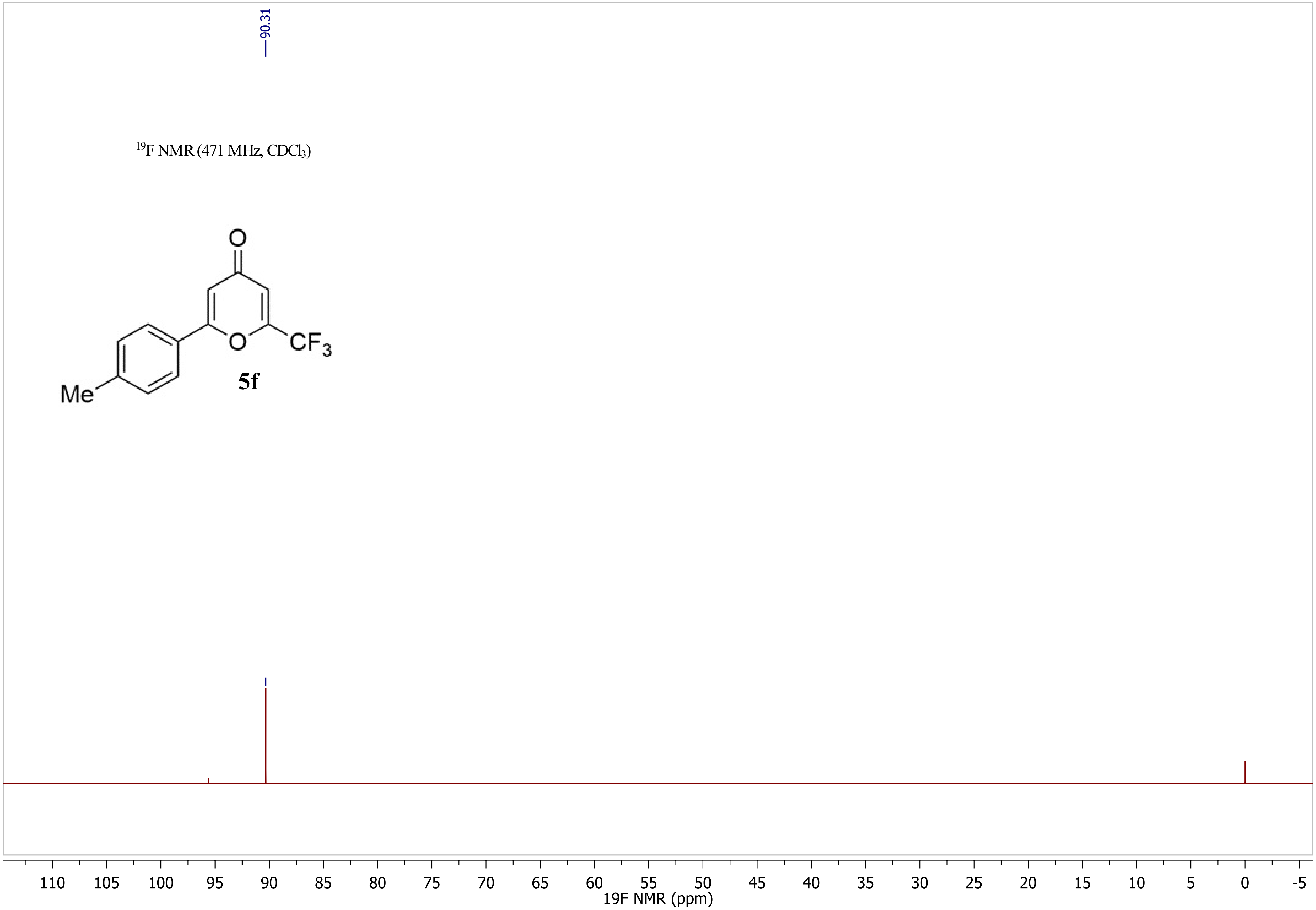

90.31
19F NMR (471 MHz, CDCl3)
O
O
CF3
Me
5f
110 105 100 95 90 85 80 75 70 65 60 55 50 45 40 35 30 25 20 15 10 5 0 -5
19F NMR (ppm)

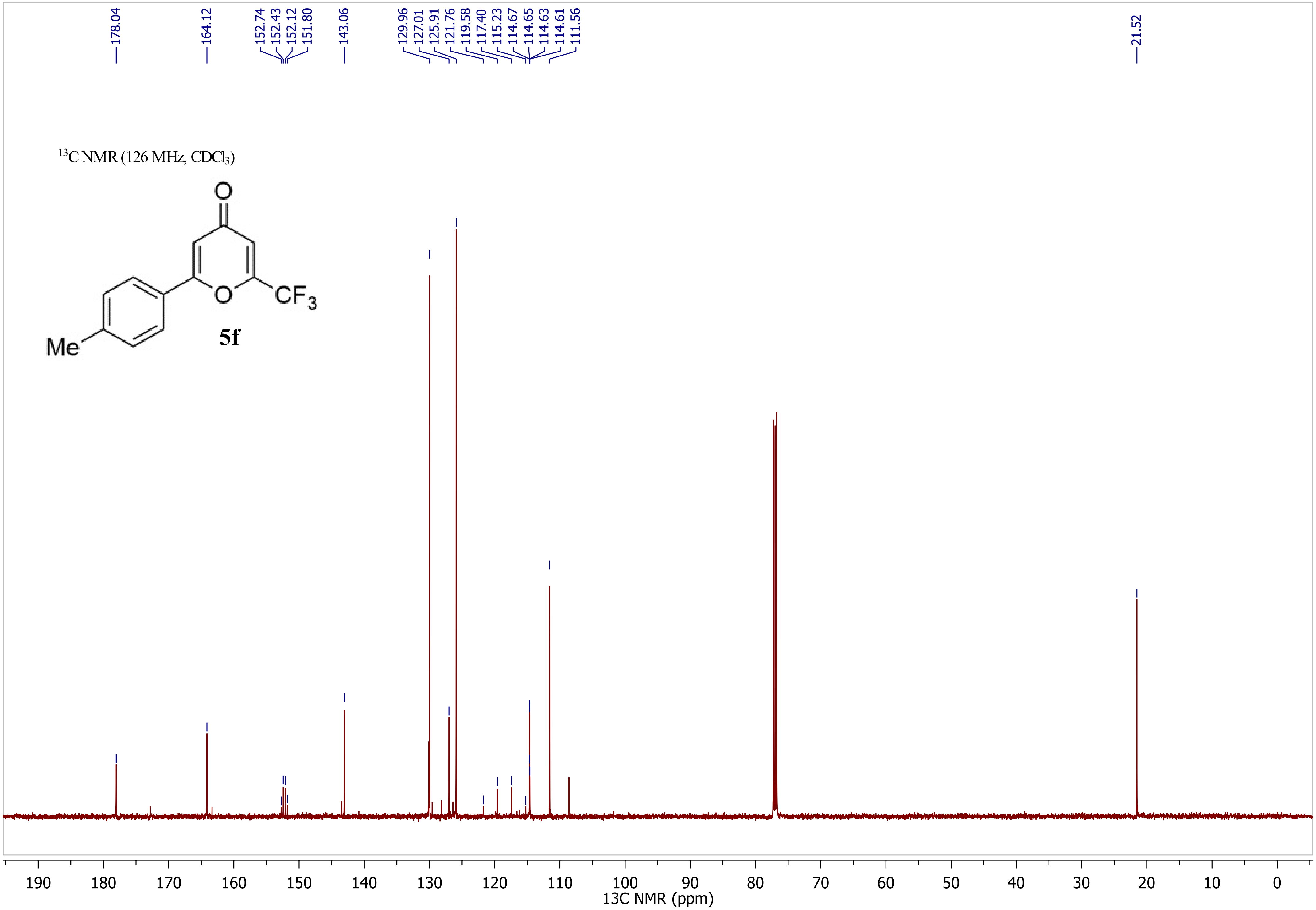
178.04
164.12
152.74
152.43
152.12
151.80
143.06
129.96
127.01
125.91
121.76
119.58
117.40
115.23
114.67
114.65
114.63
114.61
111.56
21.52
13C NMR (126 MHz, CDCl3)
O
O
CF3
Me
5f
190 180 170 160 150 140 130 120 110 100 90 80 70 60 50 40 30 20 10 0
13C NMR (ppm)

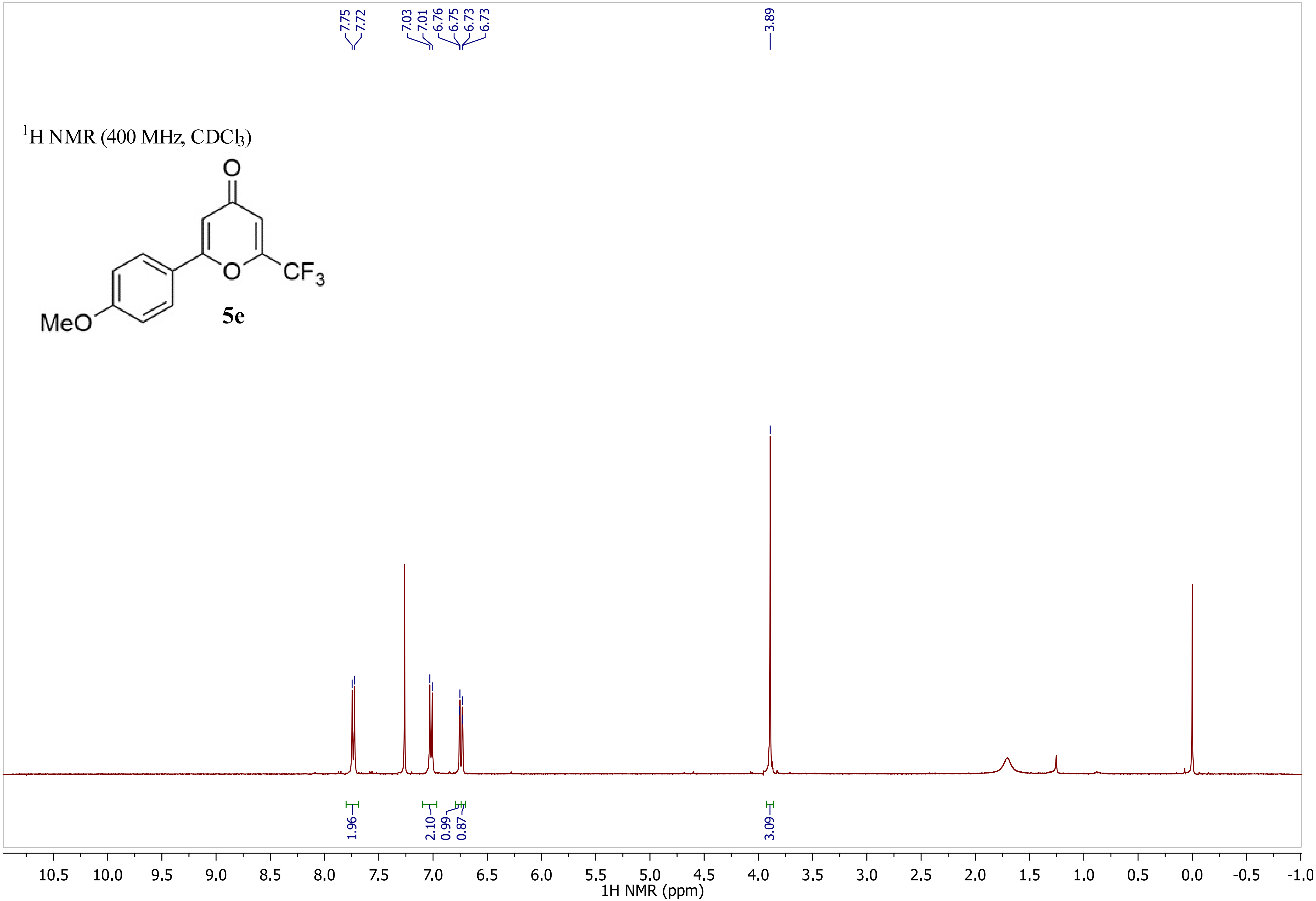
7.75
7.72
7.03
7.01
6.76
6.75
6.73
6.73
3.89
1H NMR (400 MHz, CDCl3)
O
O
CF3
MeO
5e
1.96
2.10
0.99
0.87
3.09
10.5
10.0
9.5
9.0
8.5
8.0
7.5
7.0
6.5
6.0
5.5
5.0
4.5
4.0
3.5
3.0
2.5
2.0
1.5
1.0
0.5
0.0
-0.5
-1.0
1H NMR (ppm)

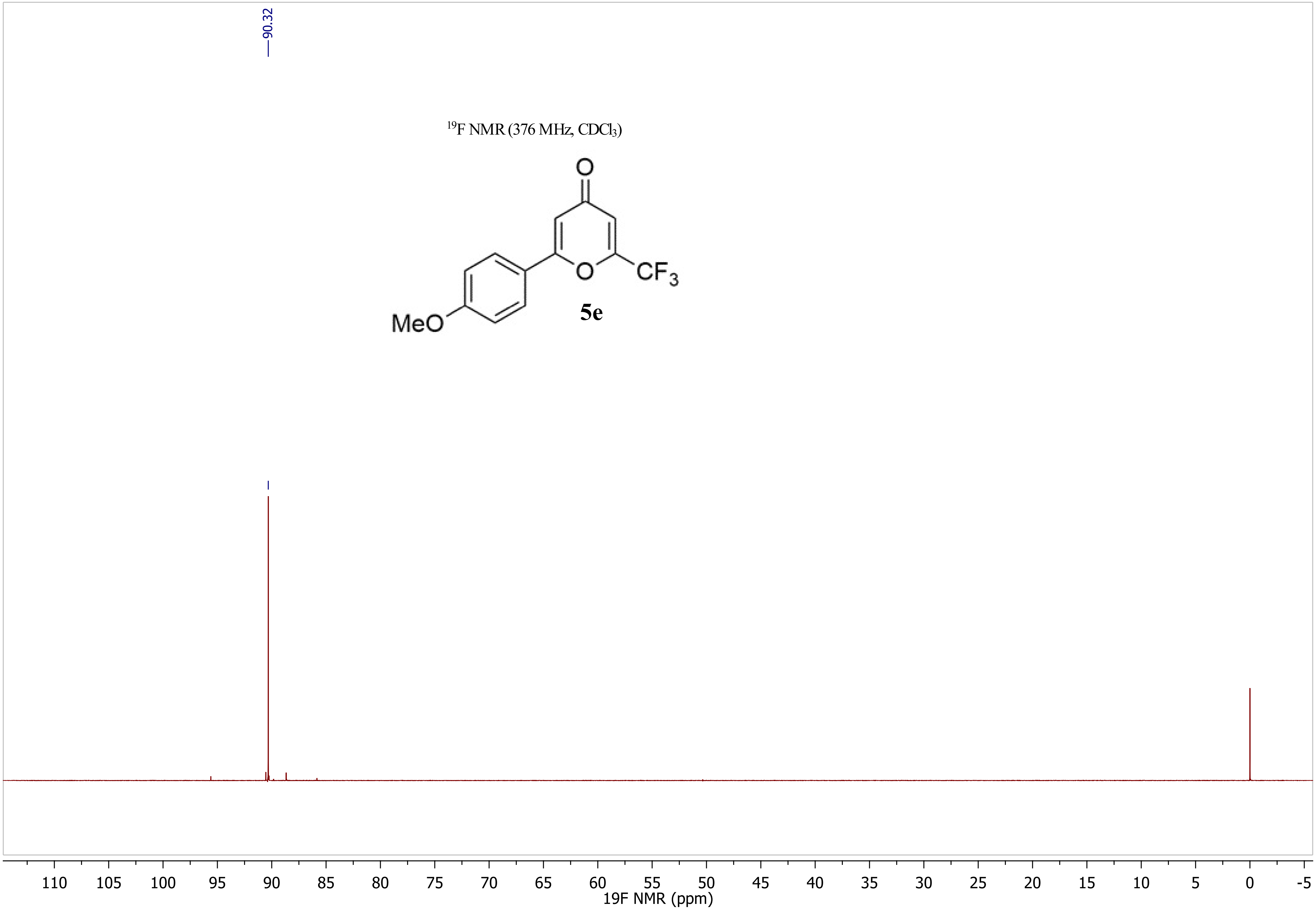

90.32
19F NMR (376 MHz, CDCl3)
O
O
CF3
MeO
5e
110
105
100
95
90
85
80
75
70
65
60
55
50
45
40
35
30
25
20
15
10
5
0
-5
19F NMR (ppm)

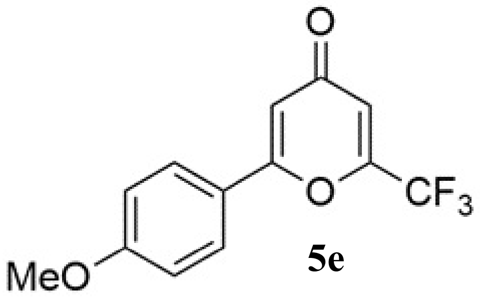
O
CF3
MeO

5e

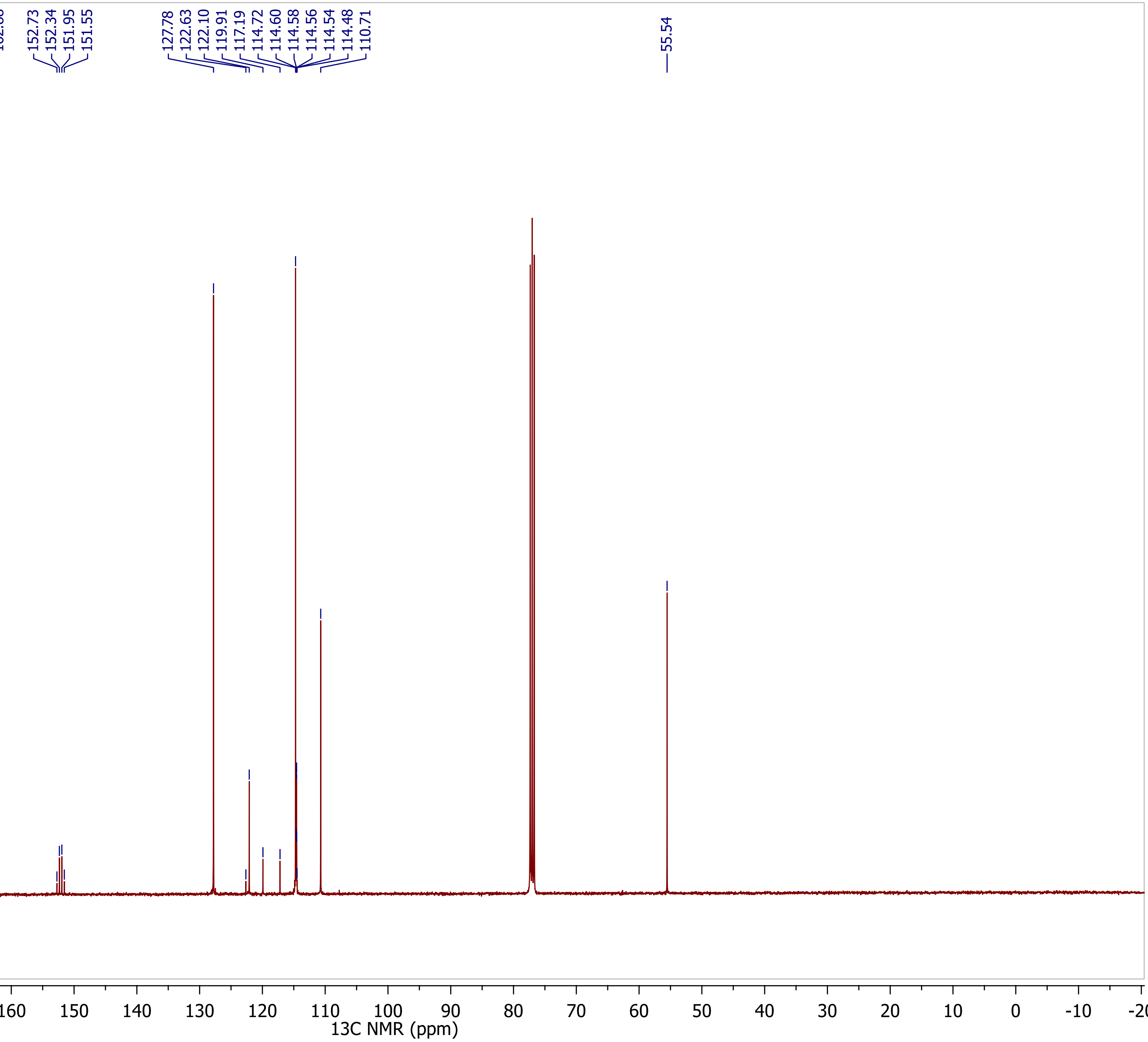
178.00
163.96
162.88
152.73
152.34
151.95
151.55
127.78
122.63
122.10
119.91
117.19
114.72
114.60
114.58
114.56
114.54
114.48
110.71
55.54
^{13}C NMR (101 MHz, $CDCl_3$)
210 200 190 180 170 160 150 140 130 120 110 100 90 80 70 60 50 40 30 20 10 0 -10 -20
13C NMR (ppm)

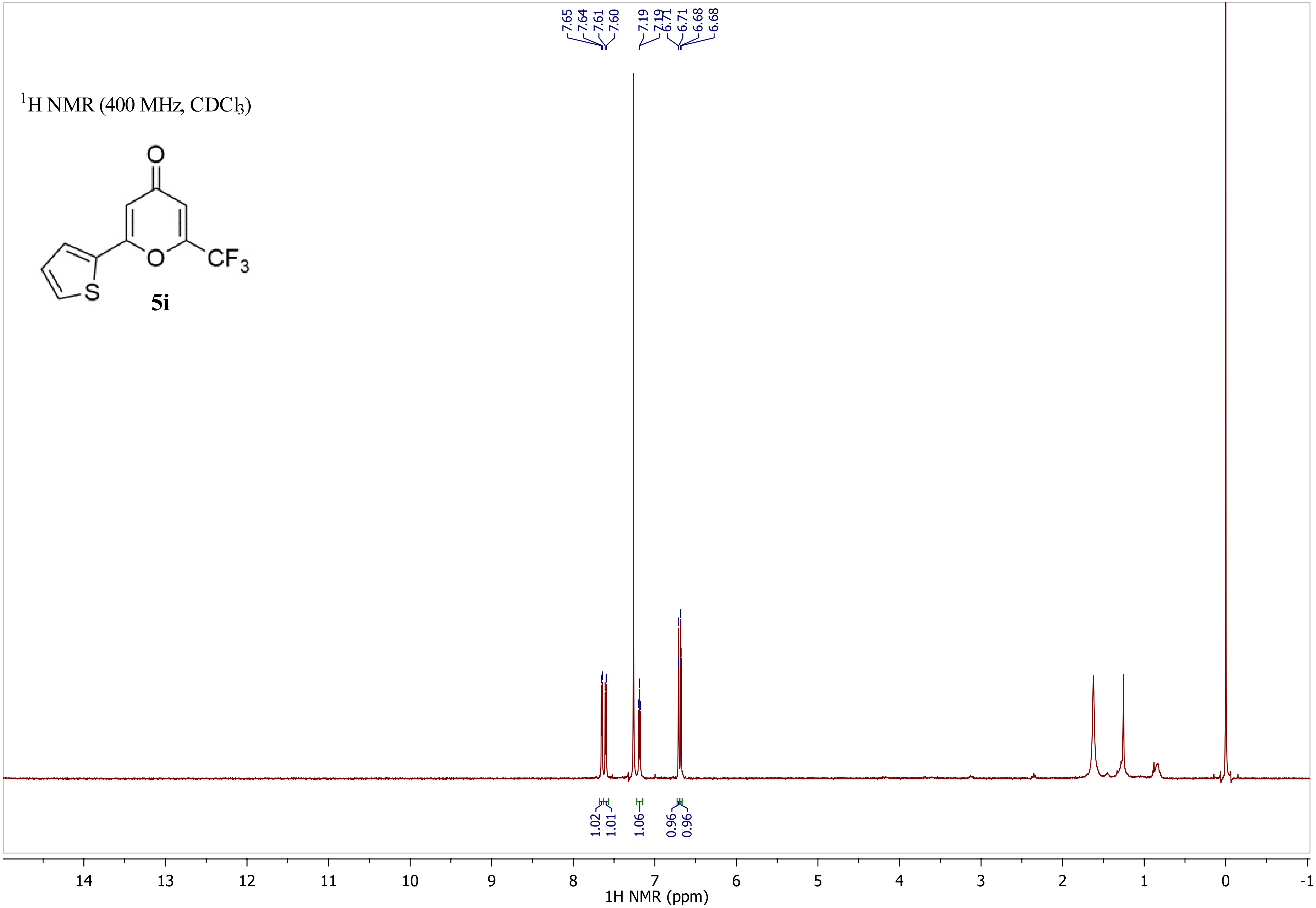

1H NMR (400 MHz, CDCl3)
O
O
CF3
S
5i
7.65
7.64
7.61
7.60
7.19
7.19
6.71
6.71
6.68
6.68
1.02
1.01
1.06
0.96
0.96
14
13
12
11
10
9
8
7
6
5
4
3
2
1
0
-1
1H NMR (ppm)

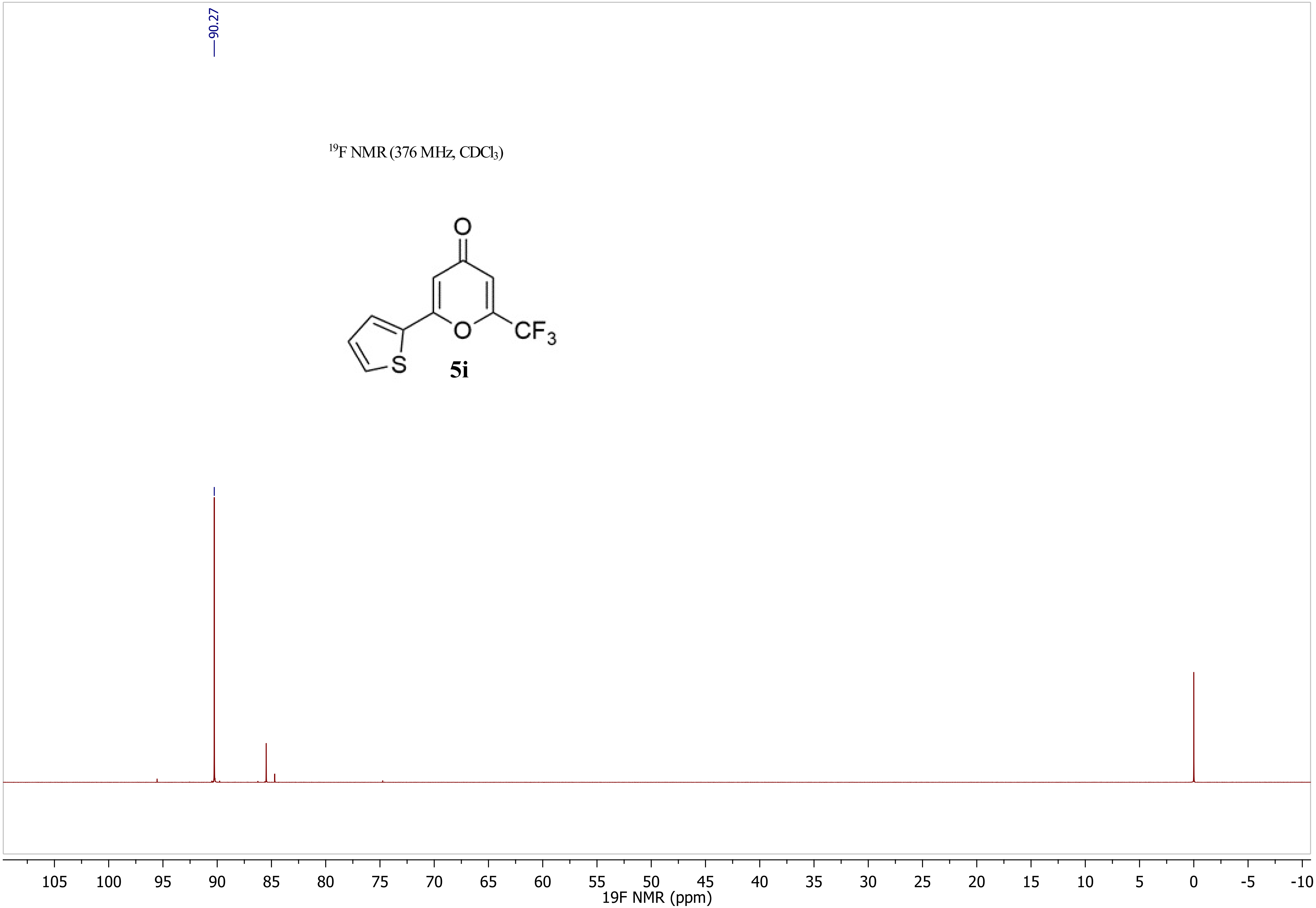

90.27
19F NMR (376 MHz, CDCl3)
O
O
S
CF3
5i
105 100 95 90 85 80 75 70 65 60 55 50 45 40 35 30 25 20 15 10 5 0 -5 -10
19F NMR (ppm)

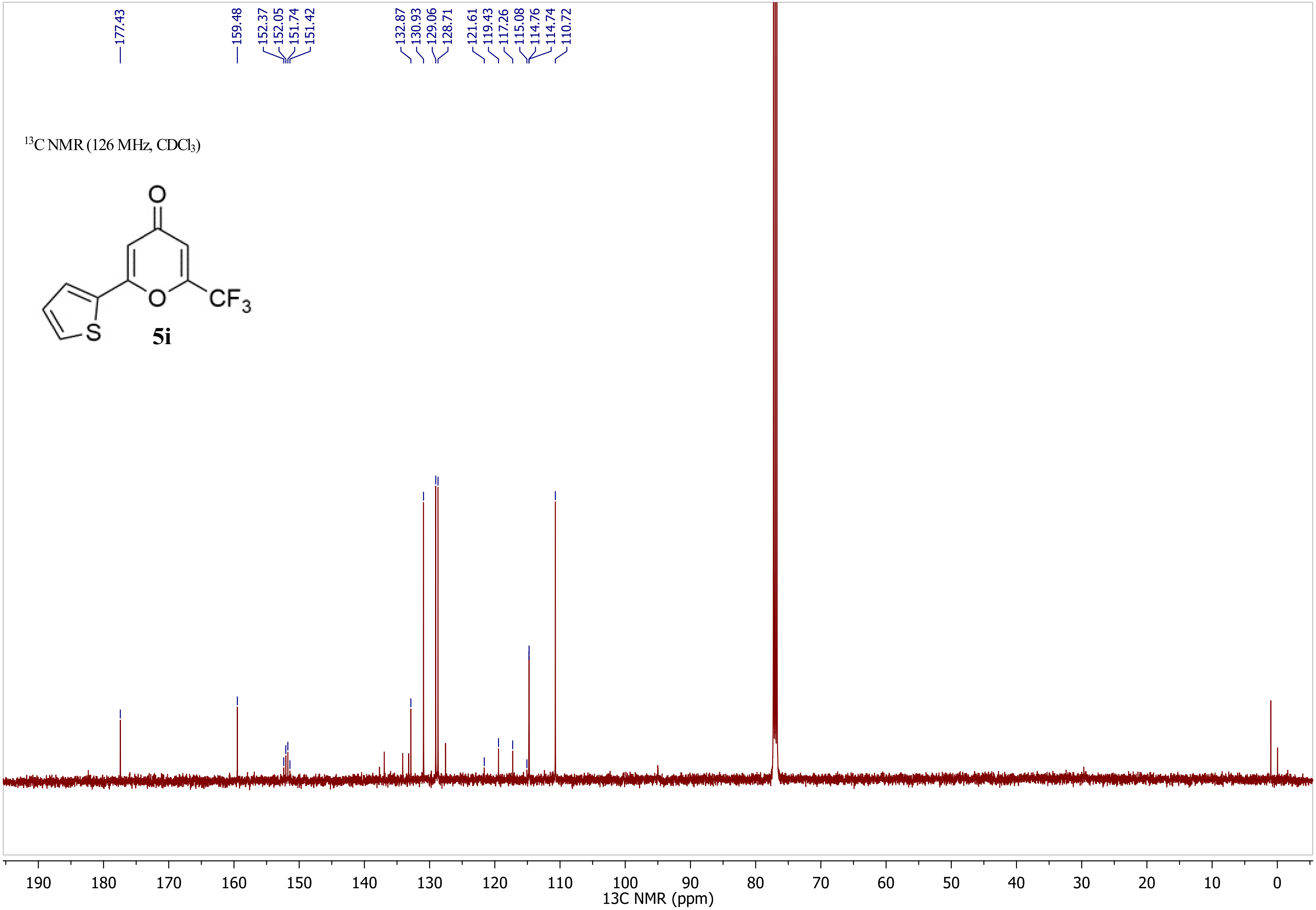

177.43
159.48
152.37
152.05
151.74
151.42
132.87
130.93
129.06
128.71
121.61
119.43
117.26
115.08
114.76
114.74
110.72
^{13}C NMR (126 MHz, $CDCl_3$)
O
O
S
CF_3
5i
190 180 170 160 150 140 130 120 110 100 90 80 70 60 50 40 30 20 10 0
13C NMR (ppm)

^{1}H NMR (500 MHz, $CDCl_3$)

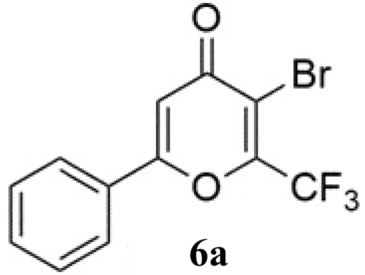

6a

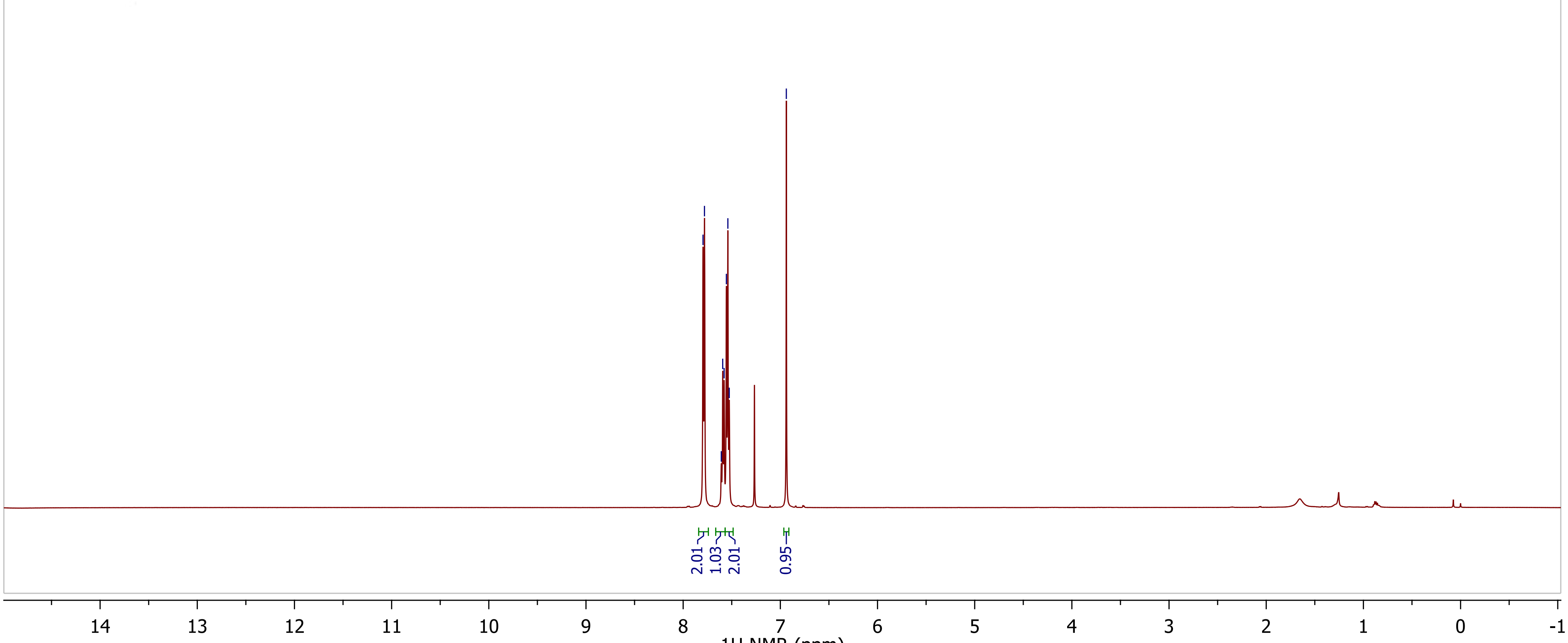

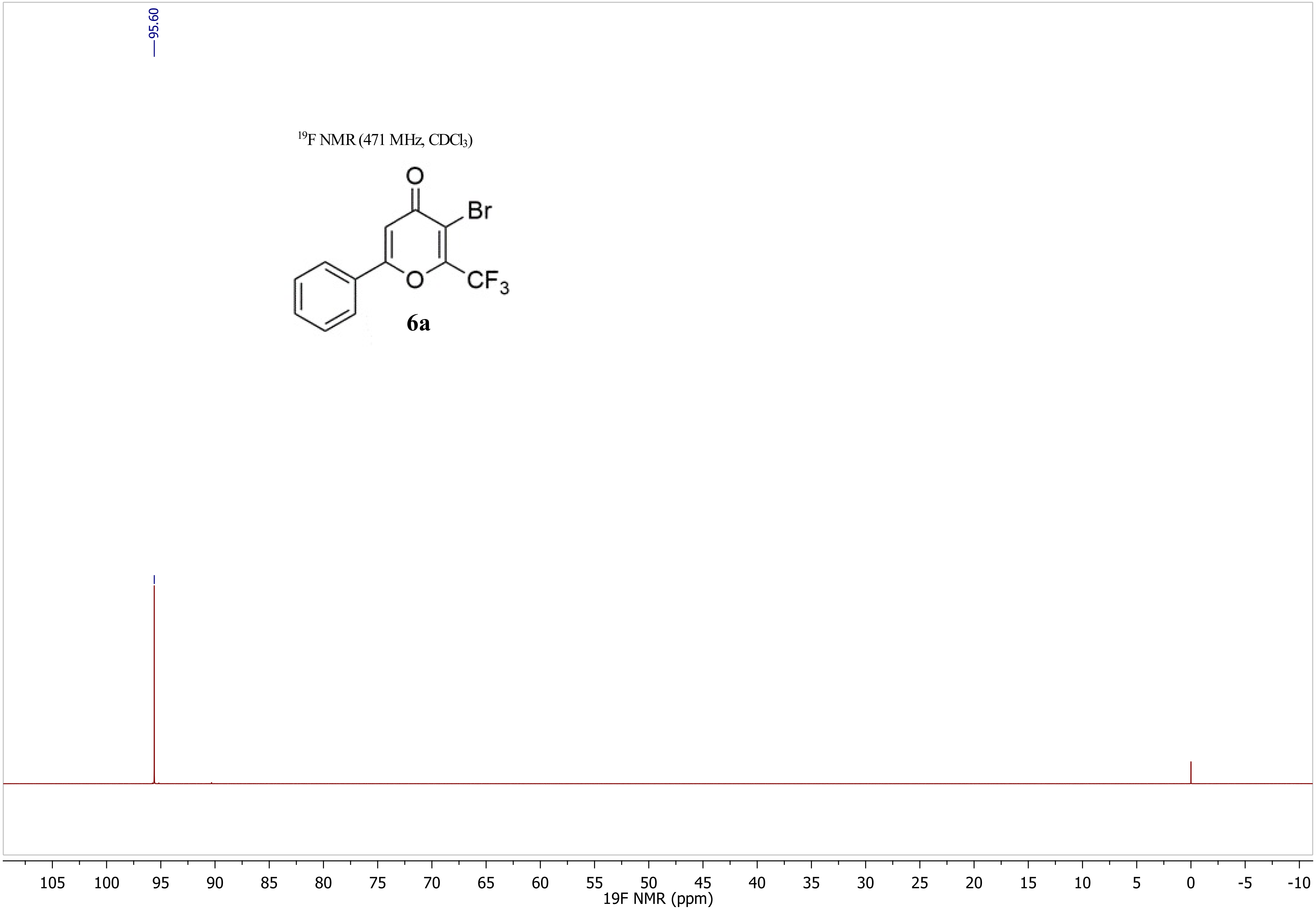

95.60
19F NMR (471 MHz, CDCl3)
O
Br
O
CF3
6a
105 100 95 90 85 80 75 70 65 60 55 50 45 40 35 30 25 20 15 10 5 0 -5 -10
19F NMR (ppm)

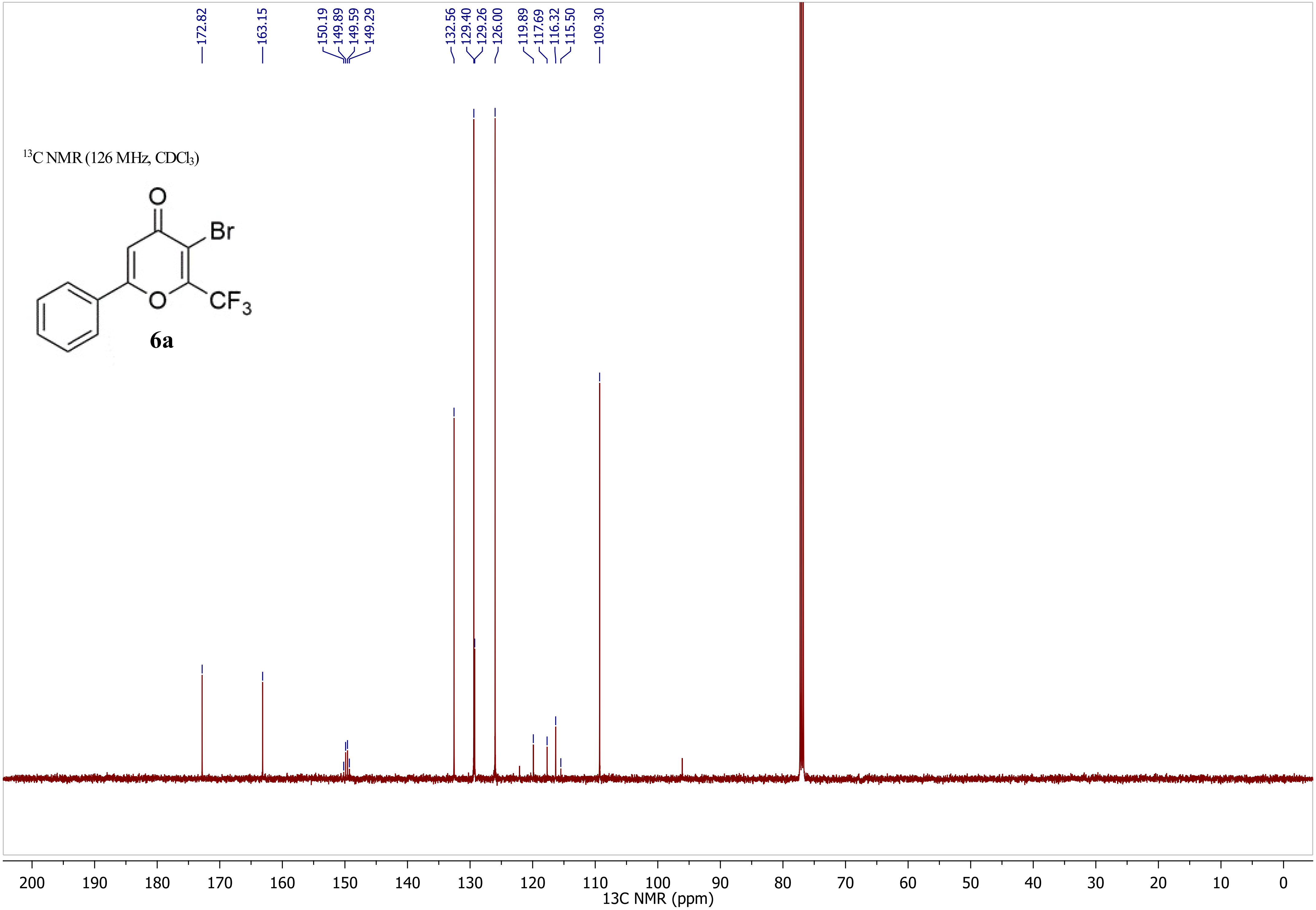
172.82
163.15
150.19
149.89
149.59
149.29
132.56
129.40
129.26
126.00
119.89
117.69
116.32
115.50
109.30
13C NMR (126 MHz, CDCl3)
O
Br
O
CF3
6a
200 190 180 170 160 150 140 130 120 110 100 90 80 70 60 50 40 30 20 10 0
13C NMR (ppm)

^{1}H NMR (400 MHz, $CDCl_3$)

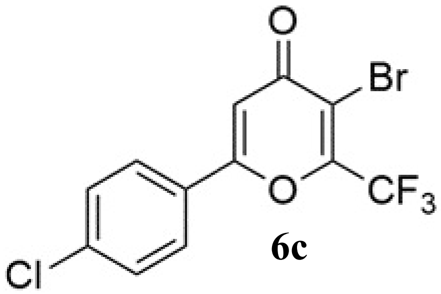

6c

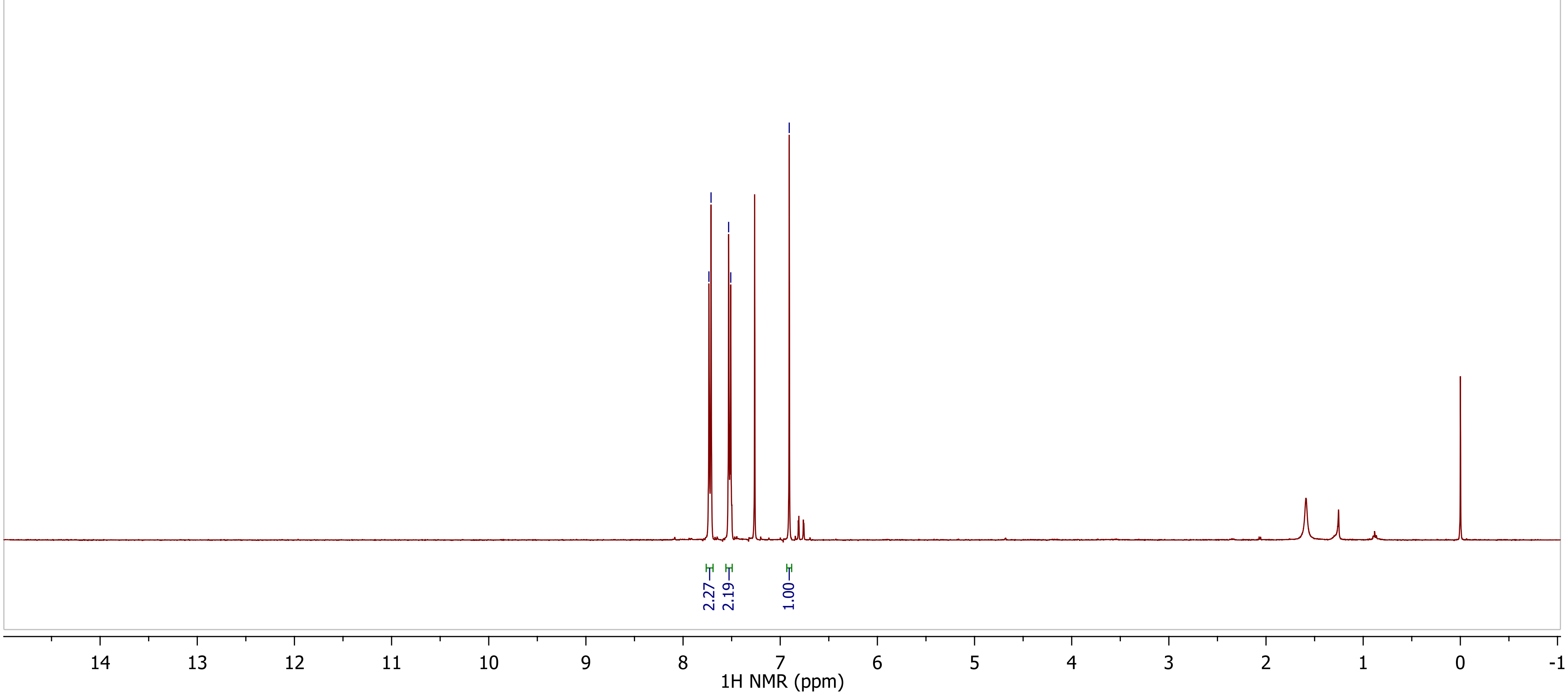

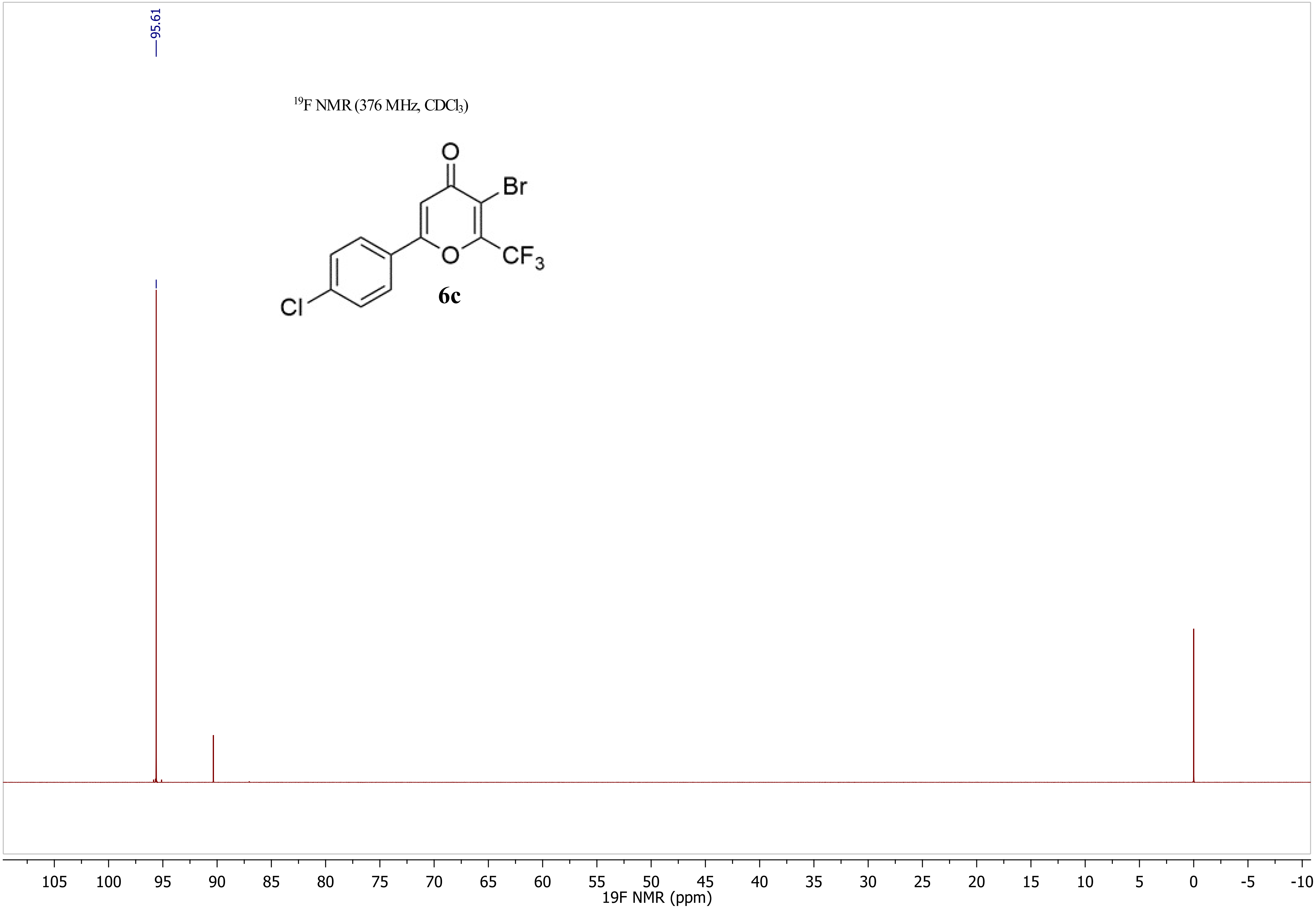
-95.61
19F NMR (376 MHz, CDCl3)
O
Br
O
CF3
Cl
6c
105 100 95 90 85 80 75 70 65 60 55 50 45 40 35 30 25 20 15 10 5 0 -5 -10
19F NMR (ppm)

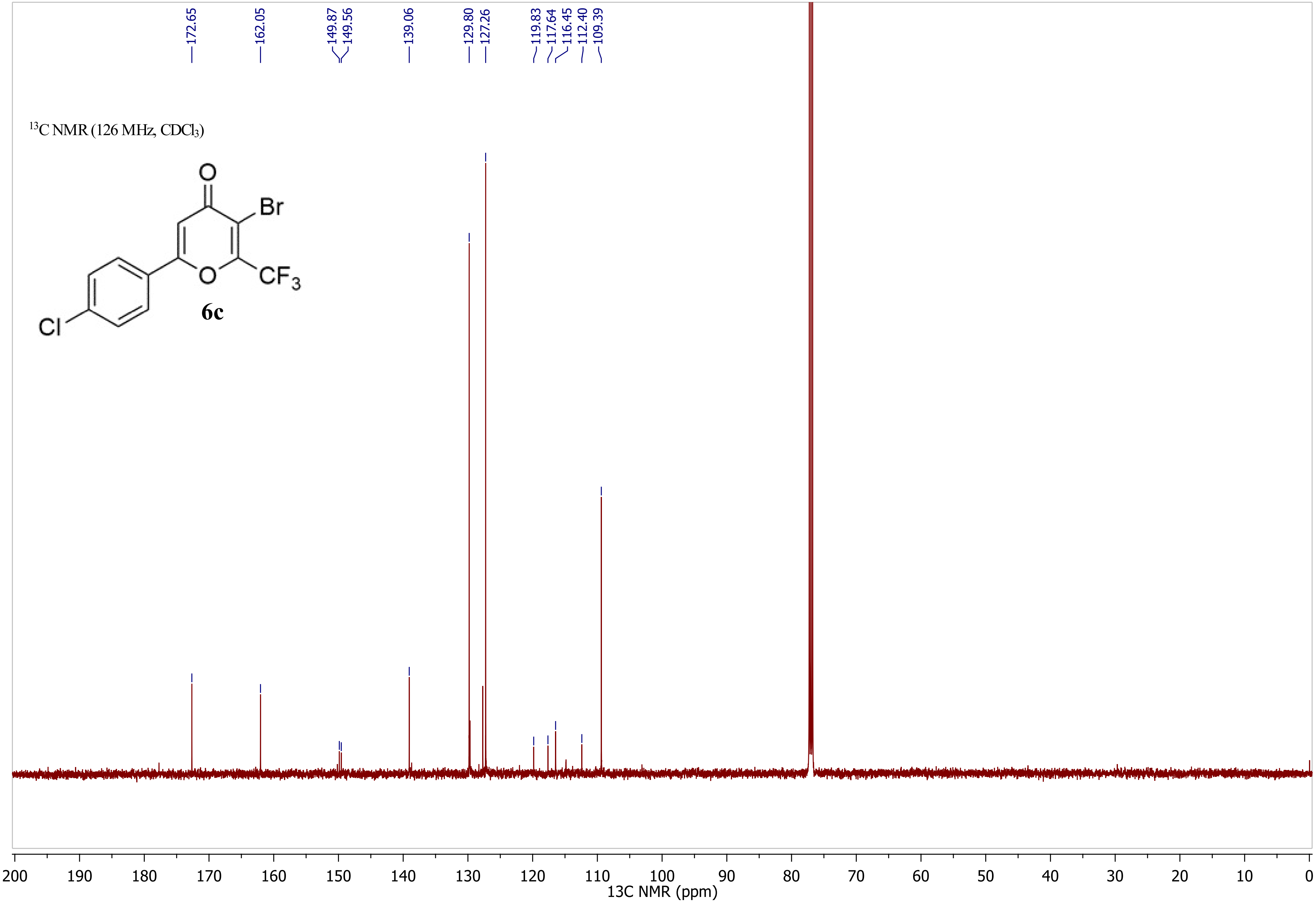
172.65
162.05
149.87
149.56
139.06
129.80
127.26
119.83
117.64
116.45
112.40
109.39
13C NMR (126 MHz, CDCl3)
O
Br
O
CF3
Cl
6c
200 190 180 170 160 150 140 130 120 110 100 90 80 70 60 50 40 30 20 10 0
13C NMR (ppm)

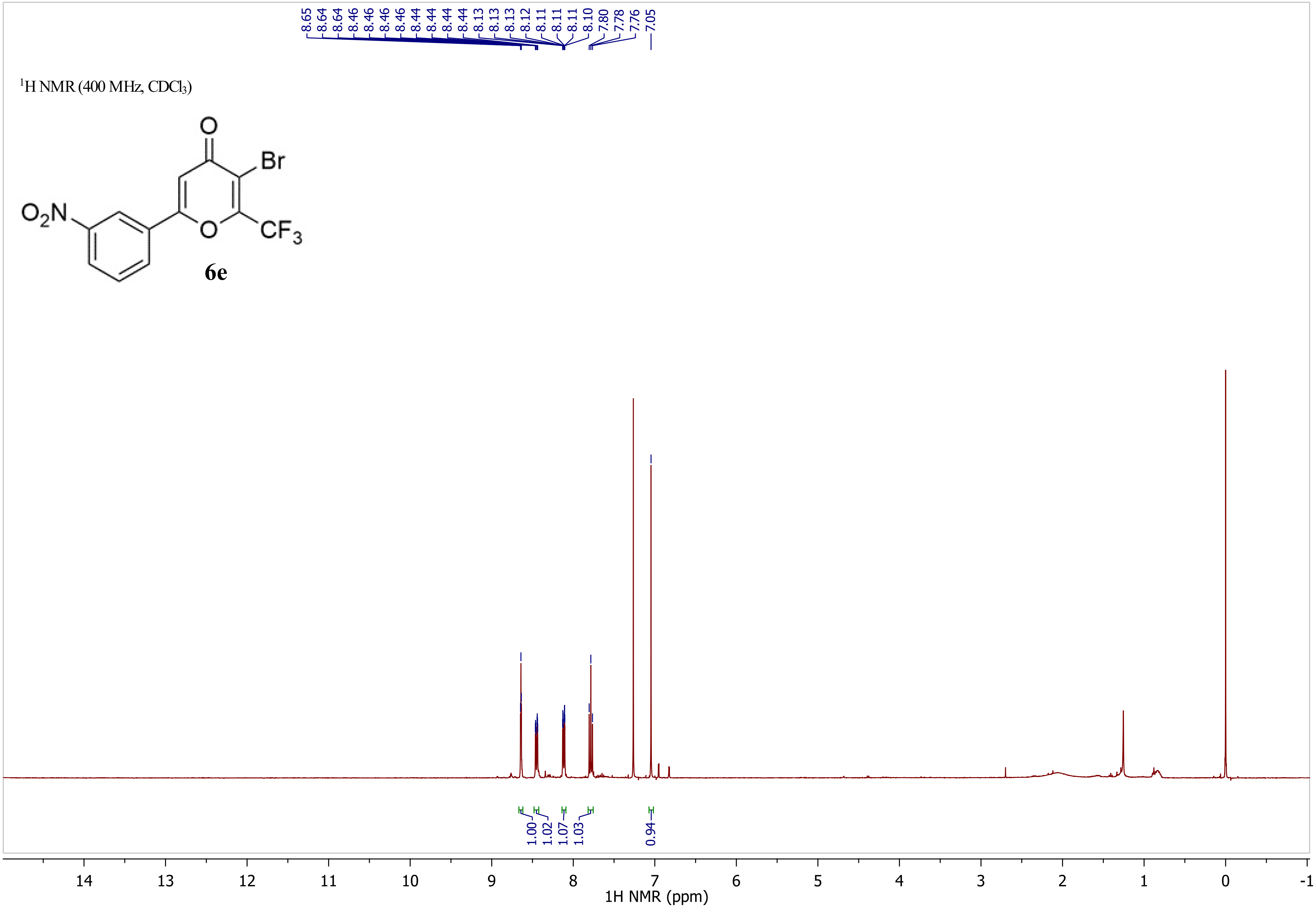

8.65
8.64
8.64
8.46
8.46
8.46
8.44
8.44
8.44
8.44
8.13
8.13
8.13
8.12
8.11
8.11
8.11
8.10
7.80
7.78
7.76
7.05
1H NMR (400 MHz, CDCl3)
O
Br
O2N
O
CF3
6e
1.00
1.02
1.07
1.03
0.94
14
13
12
11
10
9
8
7
6
5
4
3
2
1
0
-1
1H NMR (ppm)

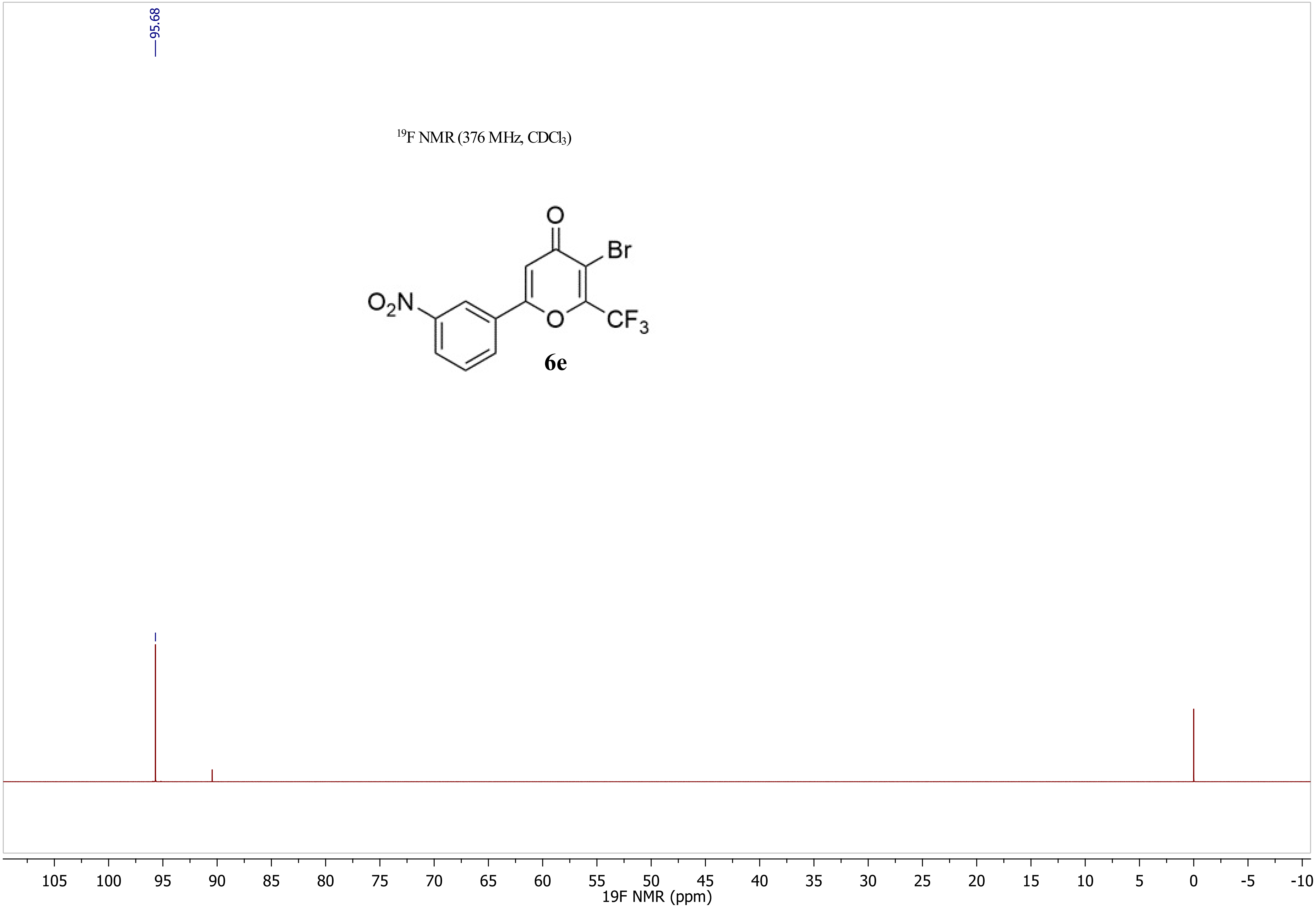

95.68
19F NMR (376 MHz, CDCl3)
O
Br
O2N
O
CF3
6e
105 100 95 90 85 80 75 70 65 60 55 50 45 40 35 30 25 20 15 10 5 0 -5 -10
19F NMR (ppm)

^1H NMR (500 MHz, $CDCl_3$)

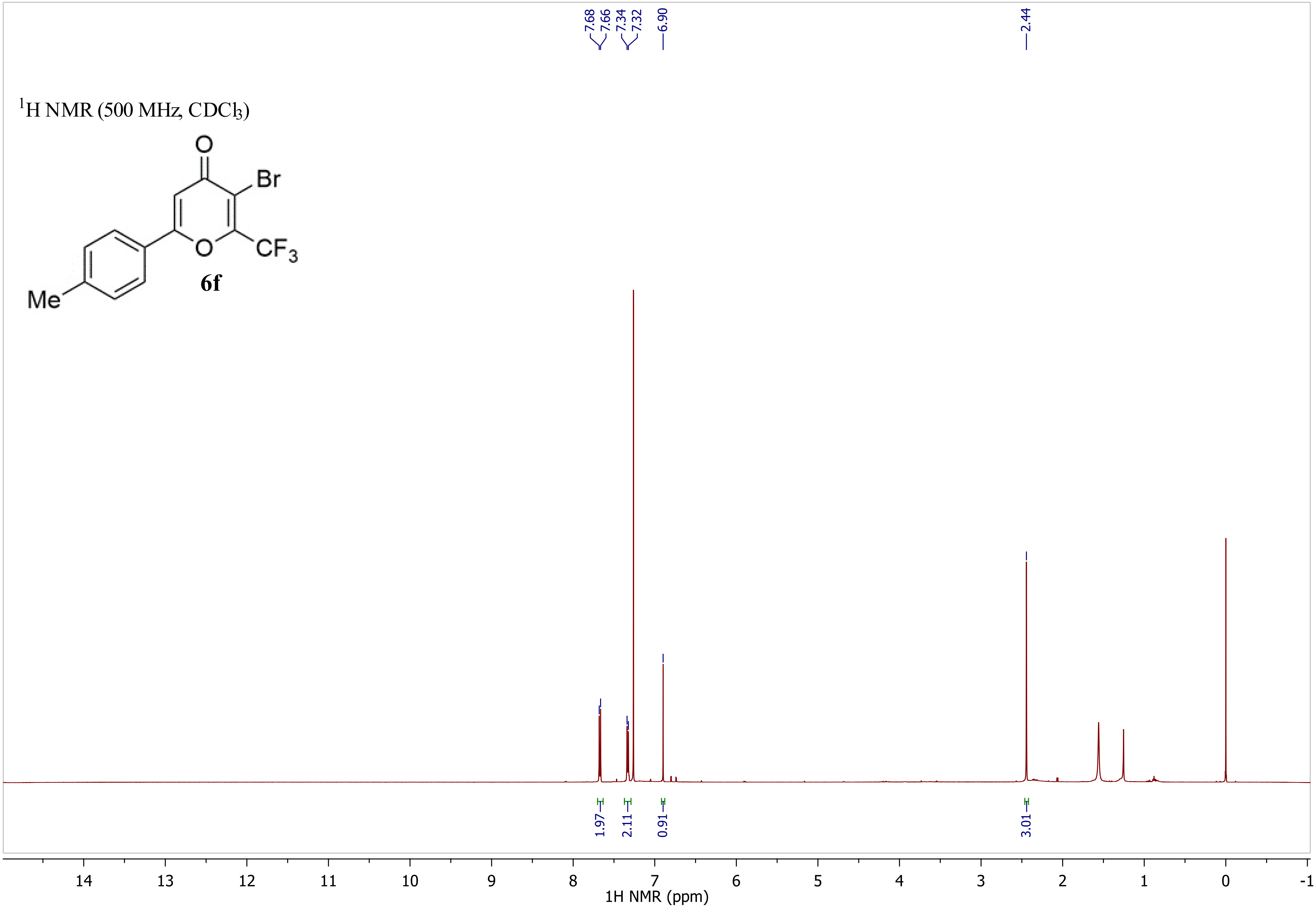

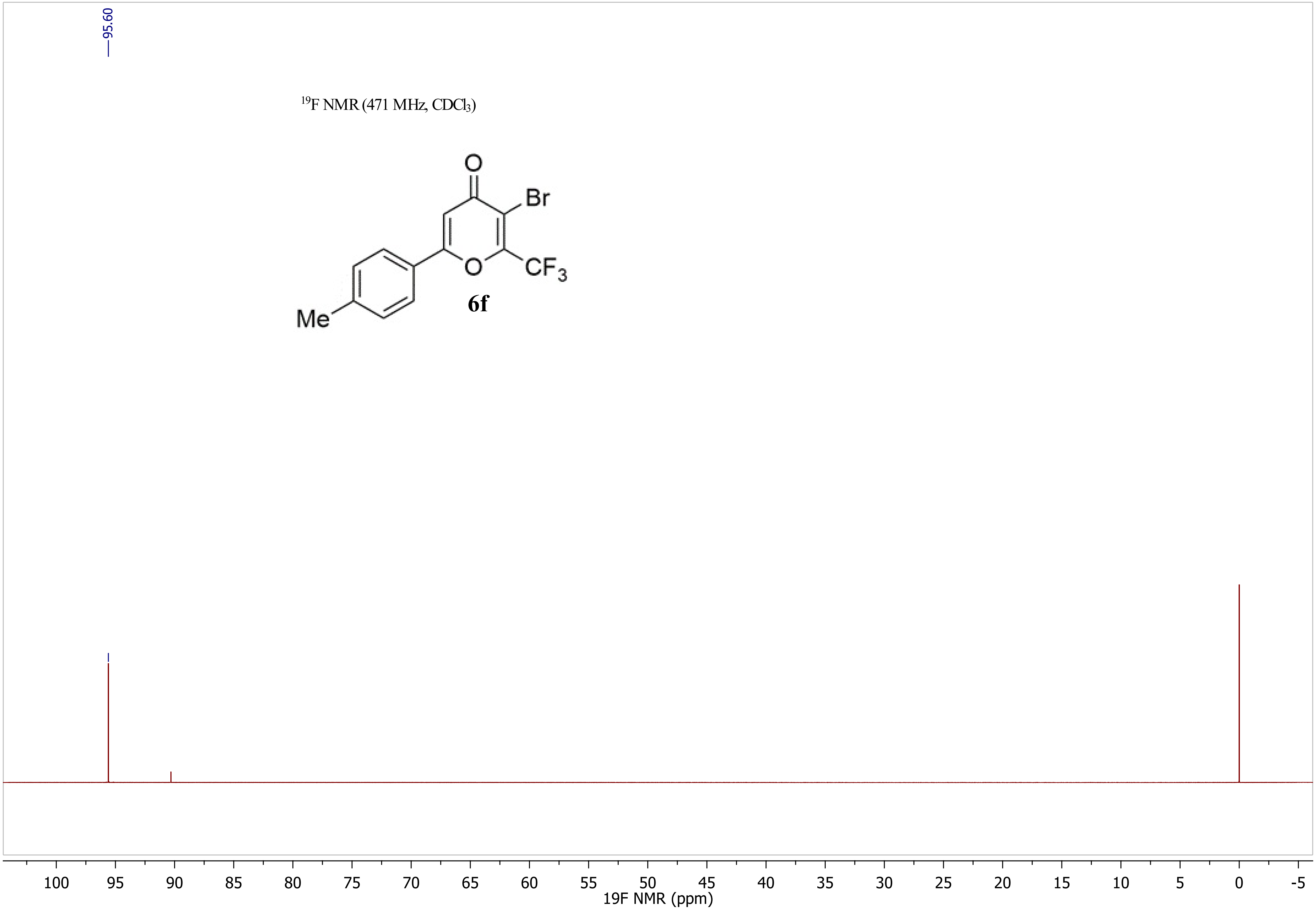

95.60
19F NMR (471 MHz, CDCl3)
O
Br
O
CF3
Me
6f
100 95 90 85 80 75 70 65 60 55 50 45 40 35 30 25 20 15 10 5 0 -5
19F NMR (ppm)

^{13}C NMR (101 MHz, $CDCl_3$)

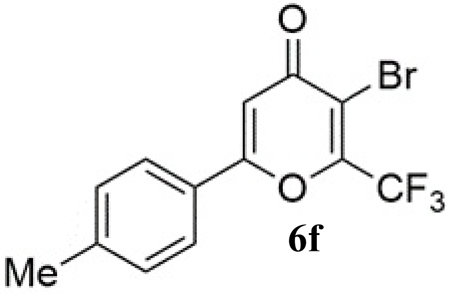

6f

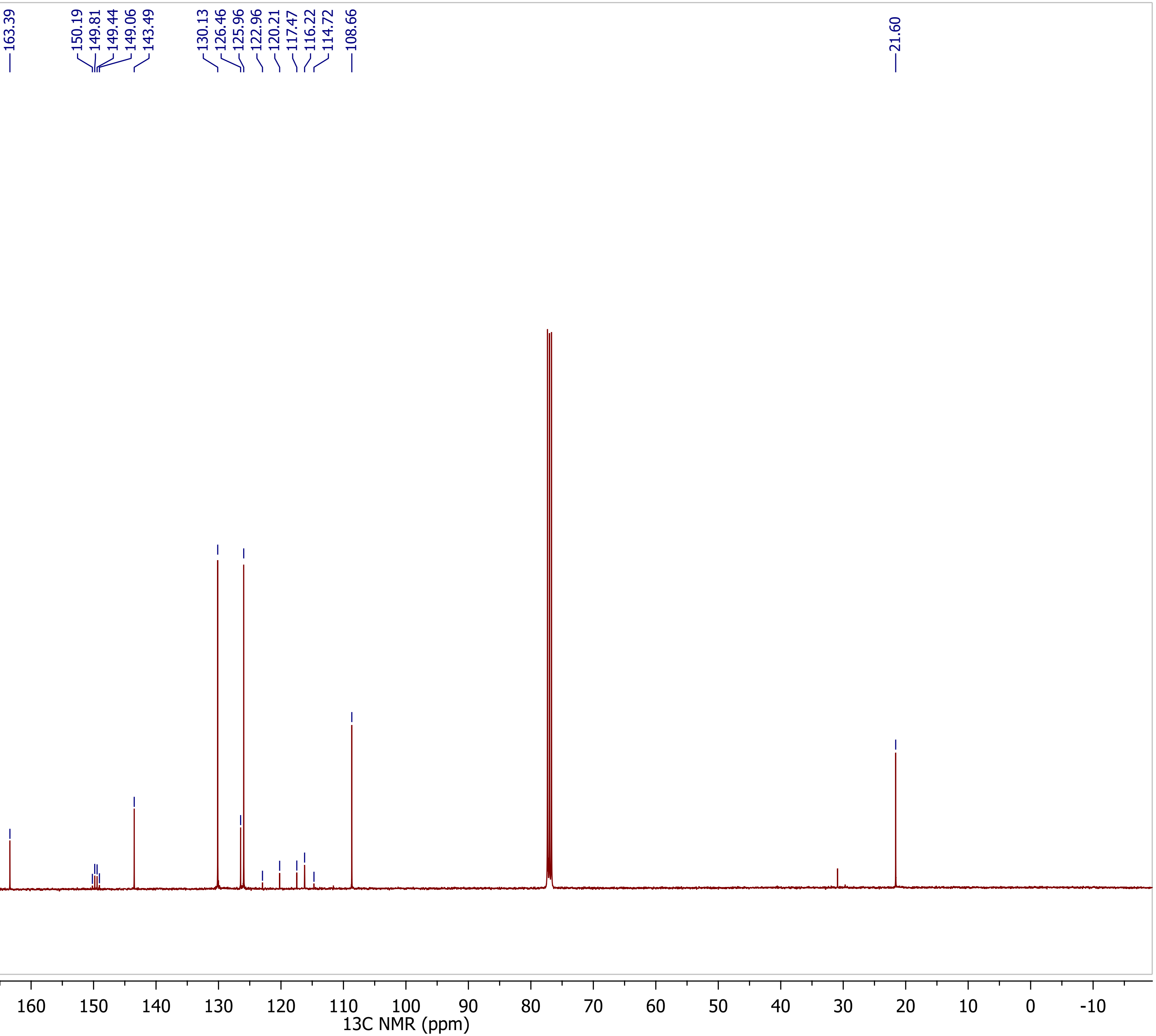

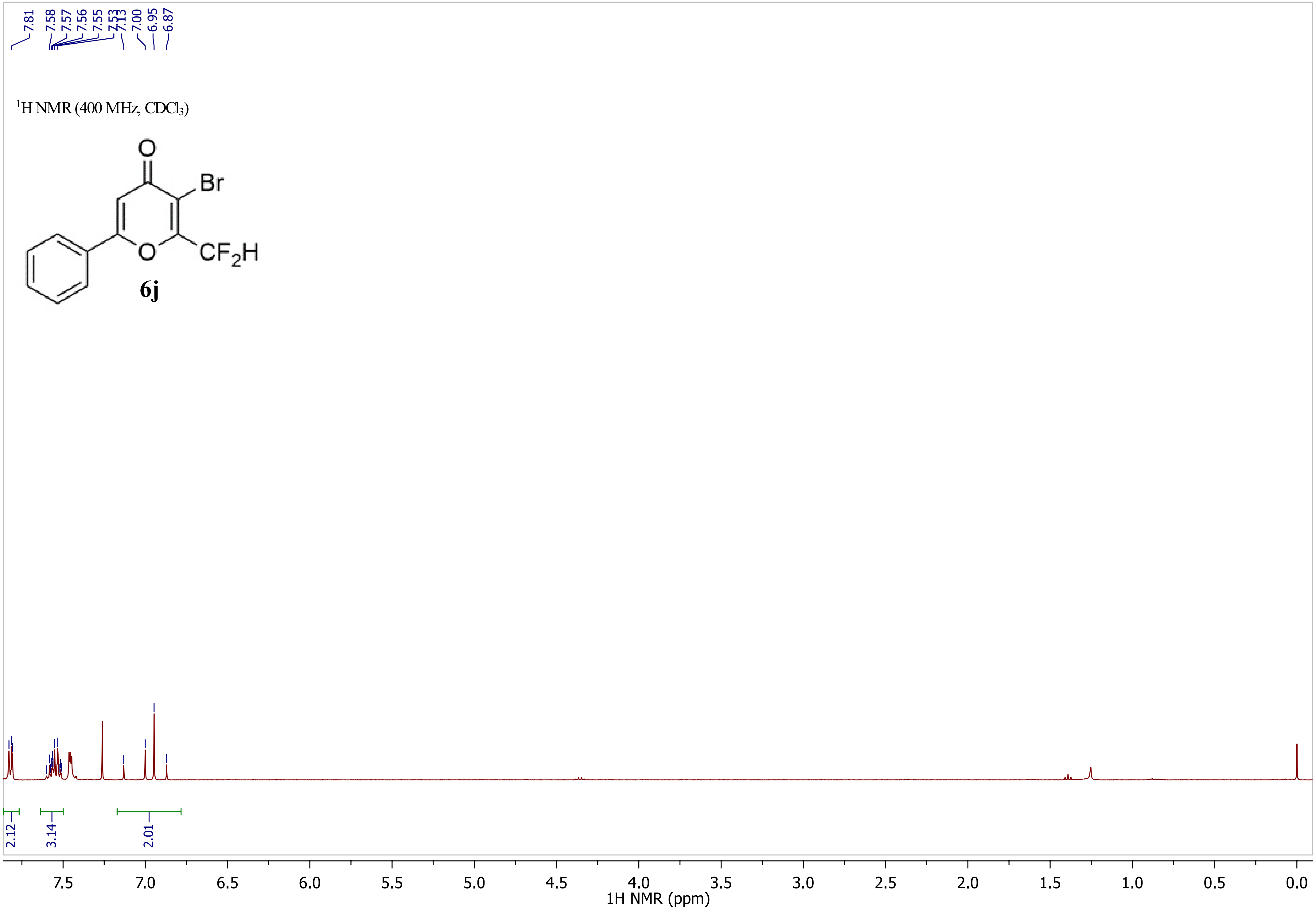

7.81
7.58
7.57
7.56
7.55
7.53
7.13
7.00
6.95
6.87
^{1}H NMR (400 MHz, CDCl$_3$)
O
Br
O
CF$_2$H
6j
2.12
3.14
2.01
7.5
7.0
6.5
6.0
5.5
5.0
4.5
4.0
3.5
3.0
2.5
2.0
1.5
1.0
0.5
0.0
1H NMR (ppm)

^{19}F NMR (376 MHz, CDCl$_3$)

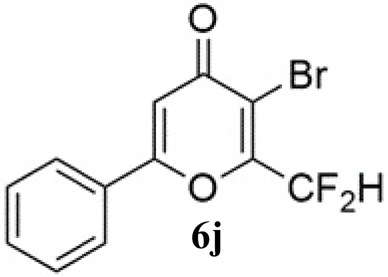

6j

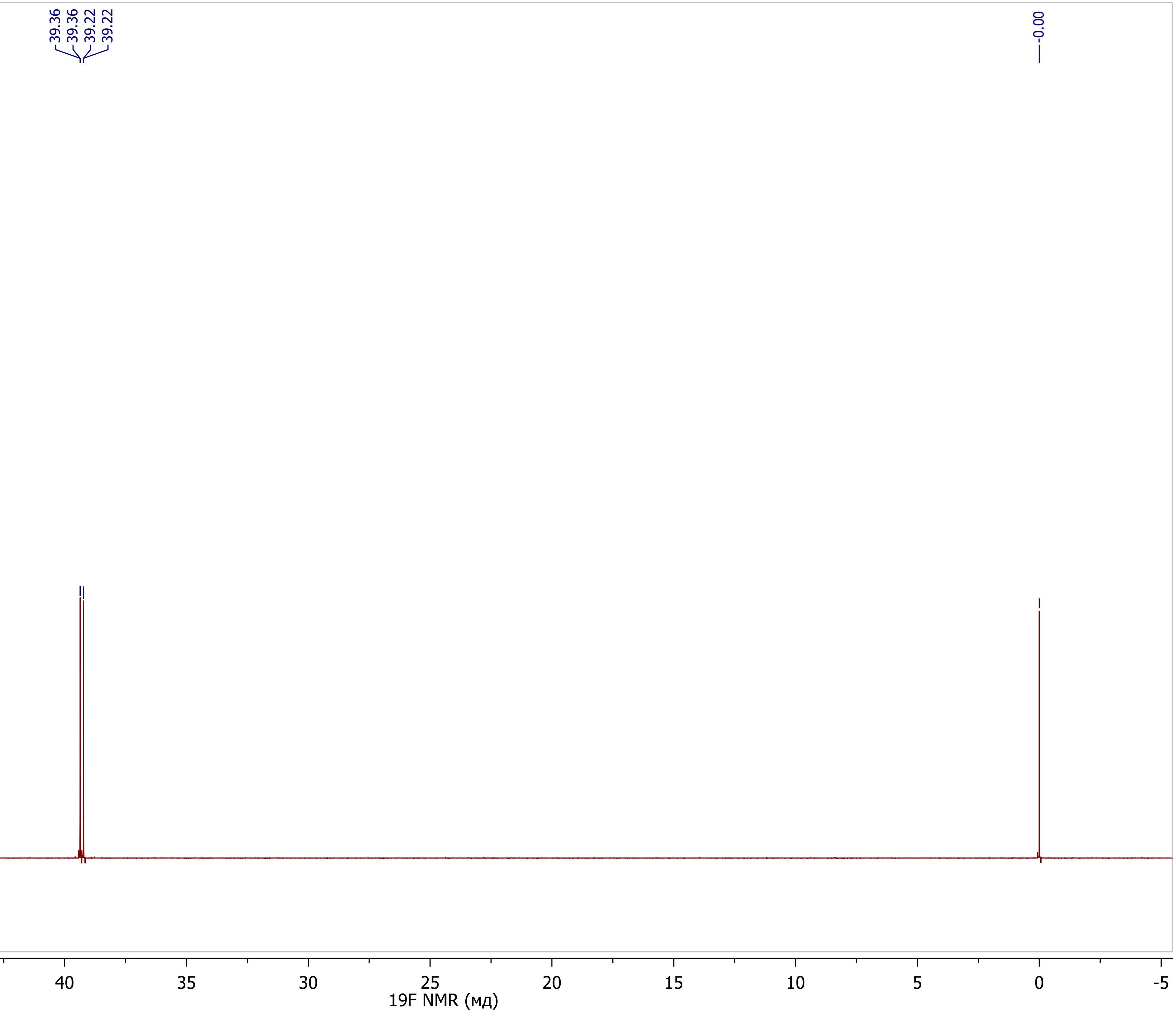

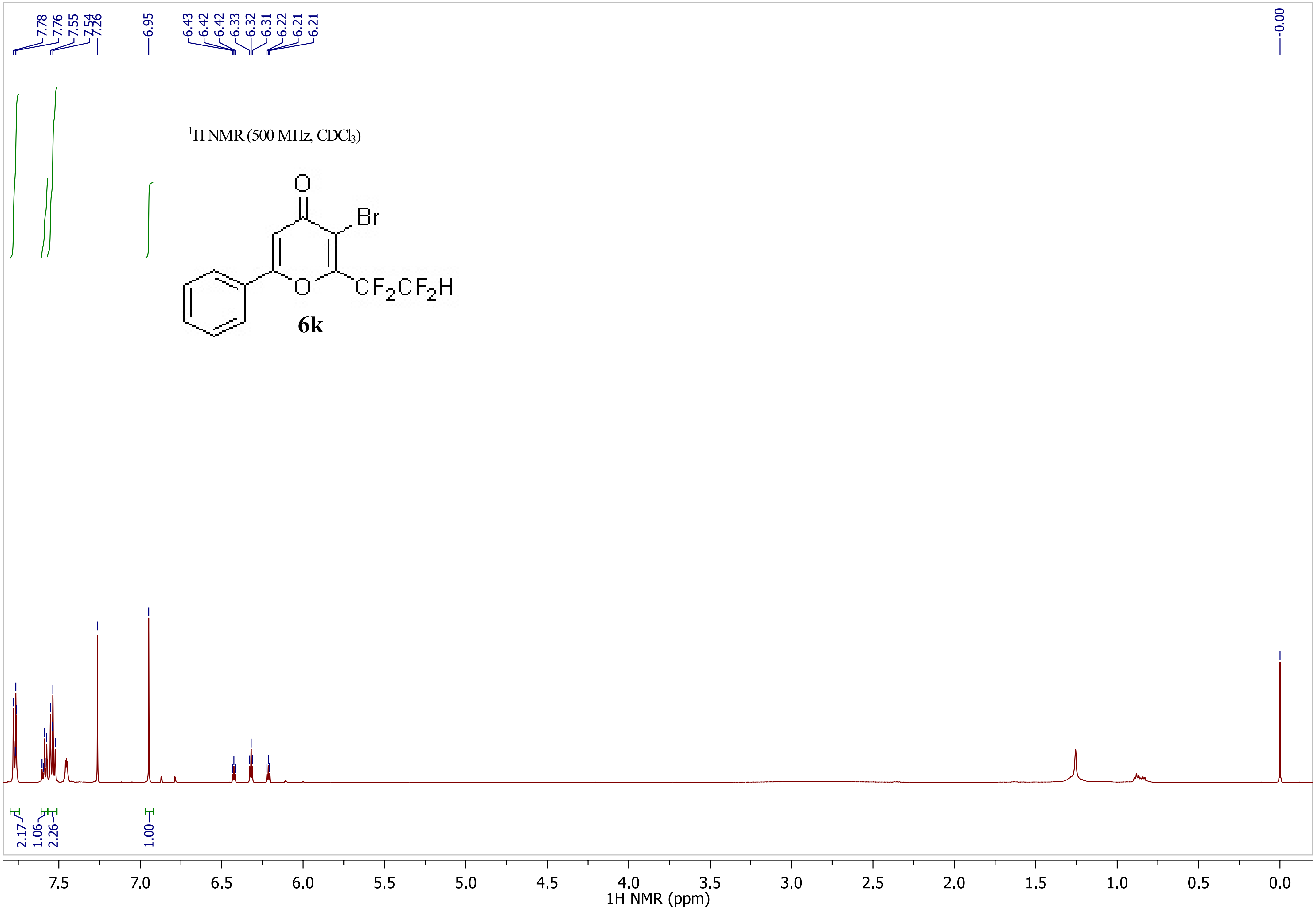

7.78
7.76
7.55
7.54
7.26
6.95
6.43
6.42
6.42
6.33
6.32
6.31
6.22
6.21
6.21
0.00
^{1}H NMR (500 MHz, $CDCl_3$)
O
Br
O
CF_2CF_2H
6k
2.17
1.06
2.26
1.00
7.5
7.0
6.5
6.0
5.5
5.0
4.5
4.0
3.5
3.0
2.5
2.0
1.5
1.0
0.5
0.0
1H NMR (ppm)

^{19}F NMR (471 MHz, $CDCl_3$)

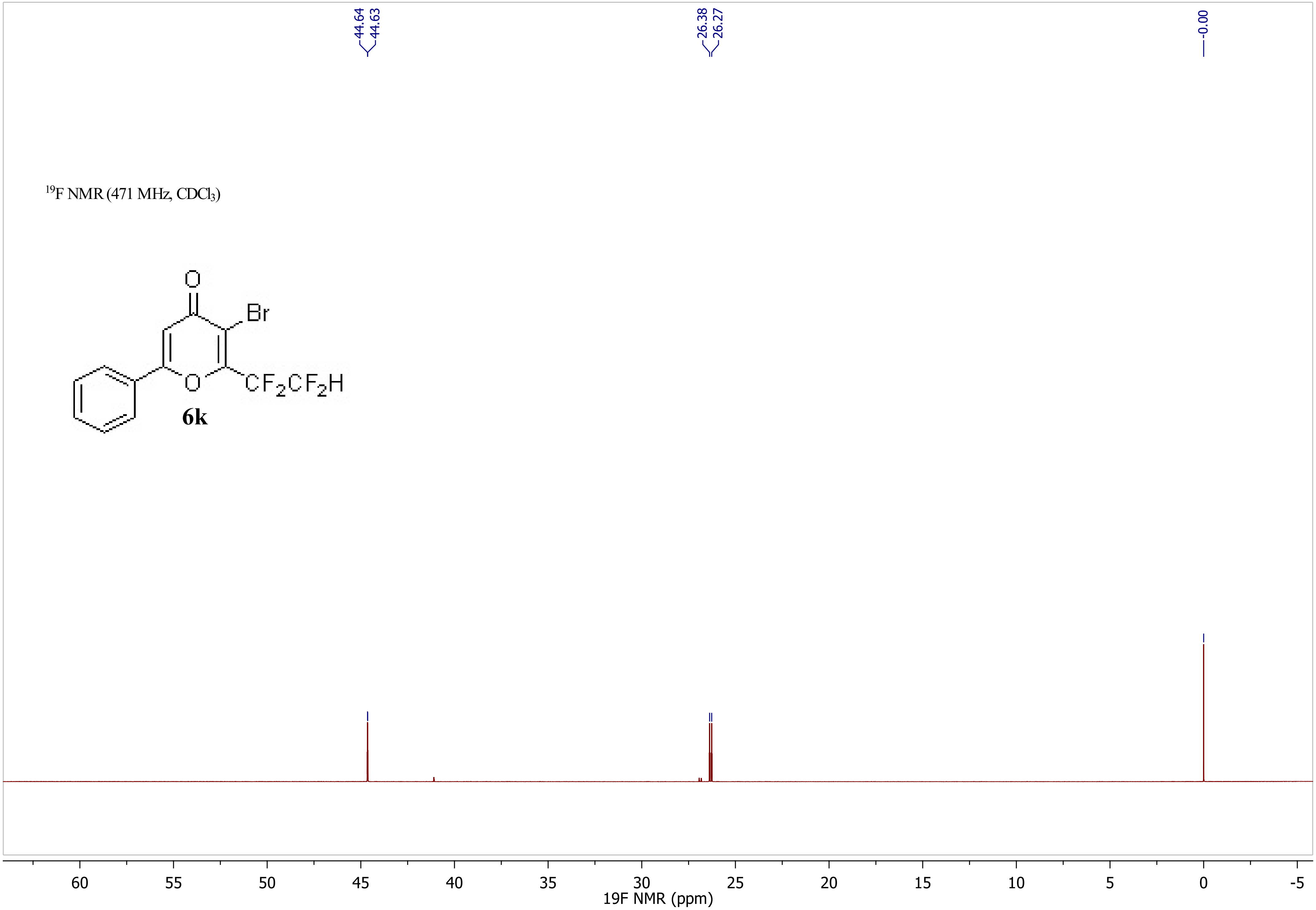

6k

^{1}H NMR (400 MHz, $CDCl_3$)

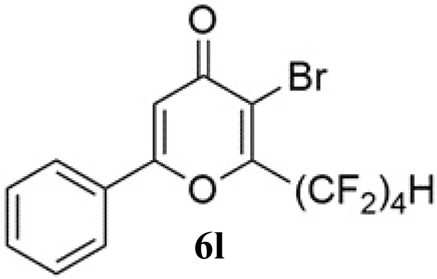

6l

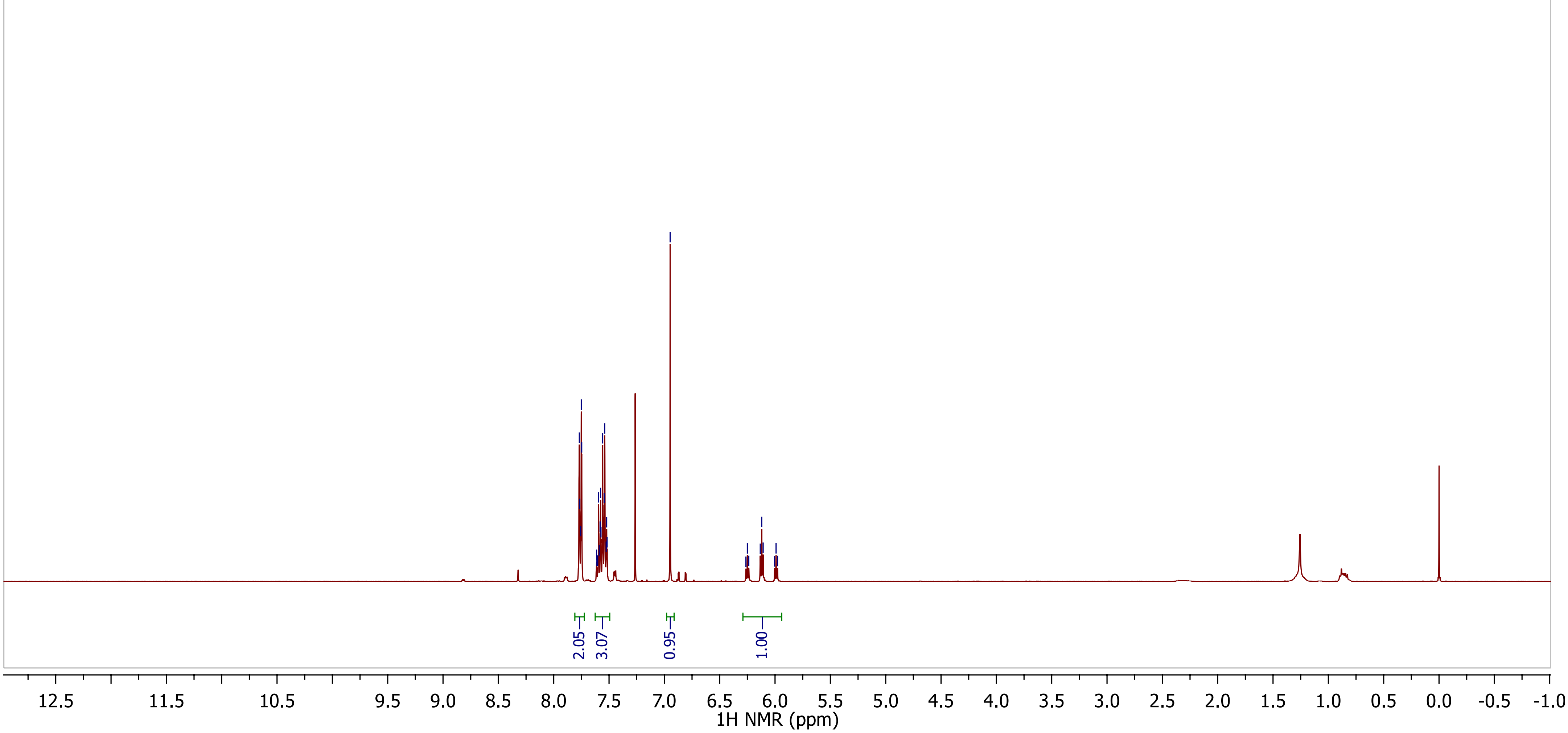

^{19}F NMR (376 MHz, $CDCl_3$)

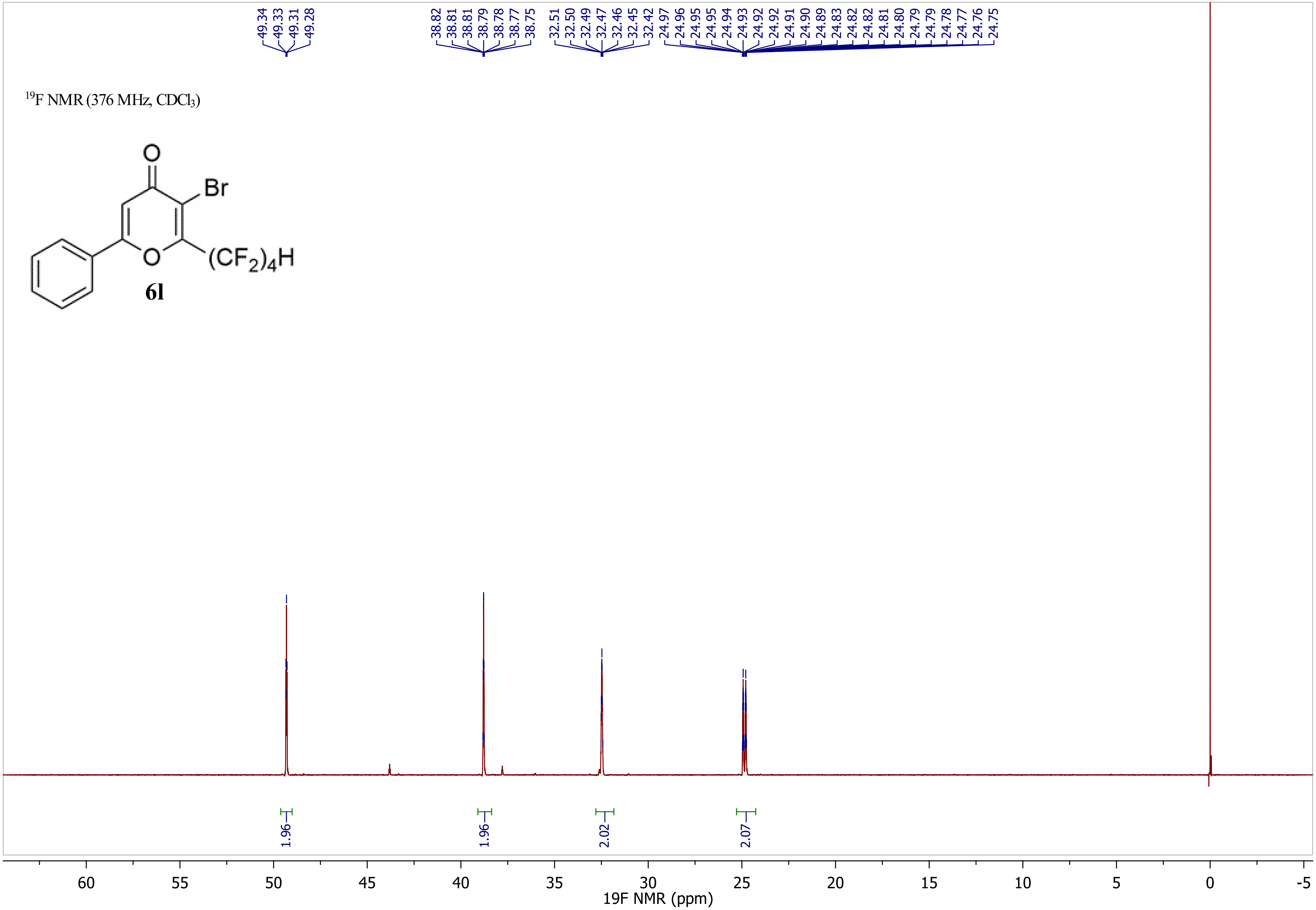

^{1}H NMR (500 MHz, $CDCl_3$)

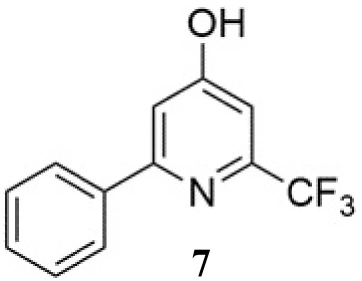

7

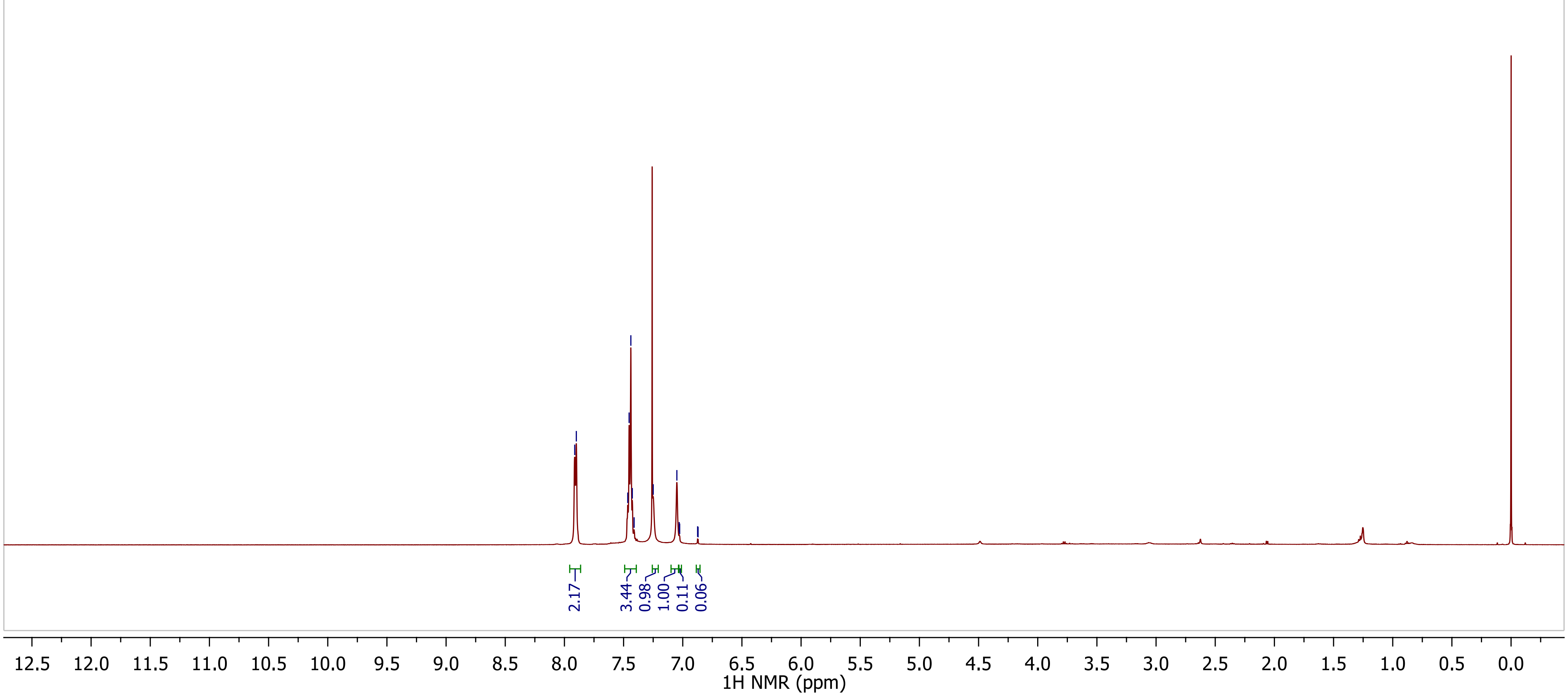

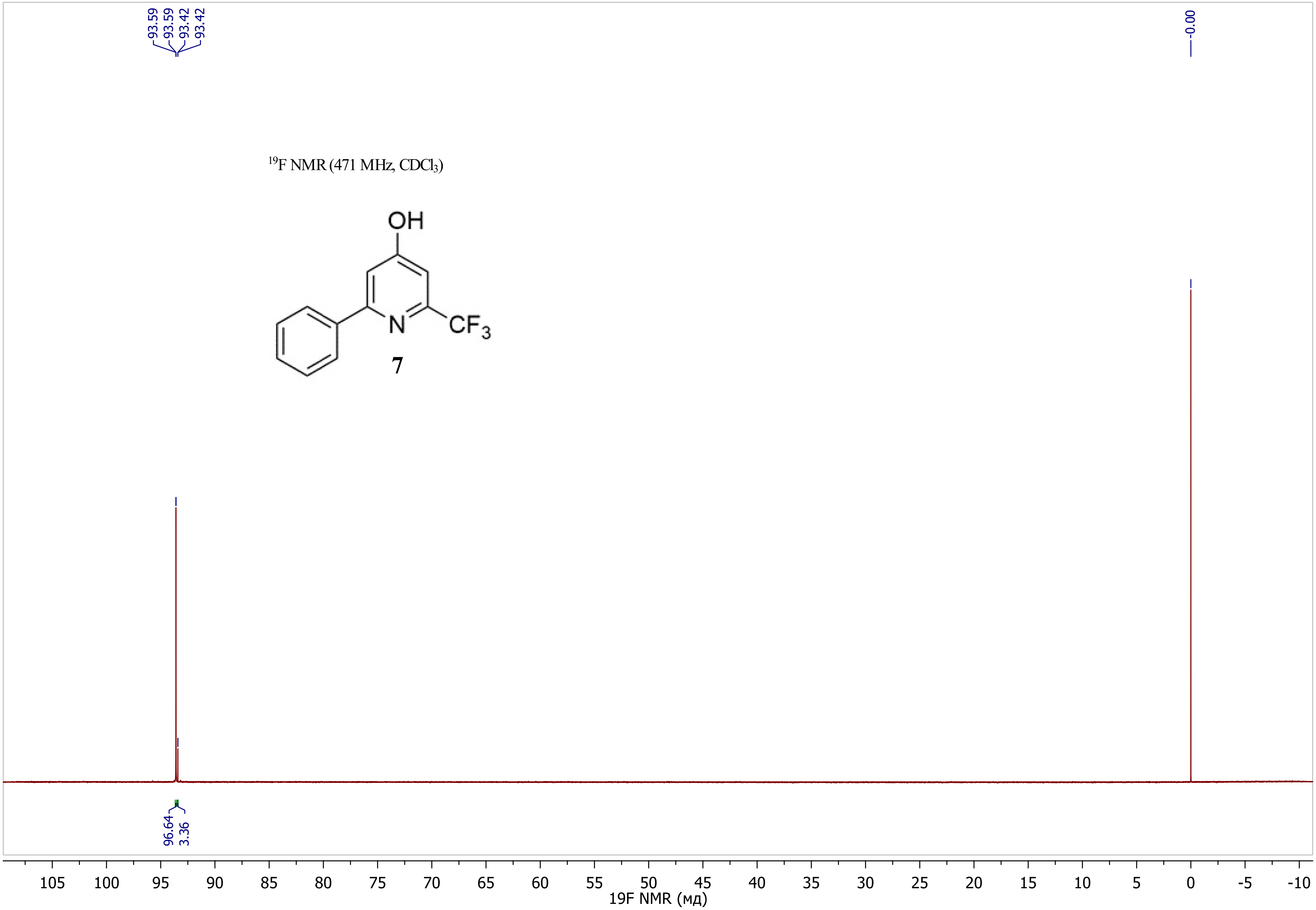
93.59
93.59
93.42
93.42
-0.00
^{19}F NMR (471 MHz, $CDCl_3$)
OH
N
CF_3
7
96.64
3.36
105 100 95 90 85 80 75 70 65 60 55 50 45 40 35 30 25 20 15 10 5 0 -5 -10
19F NMR (мд)

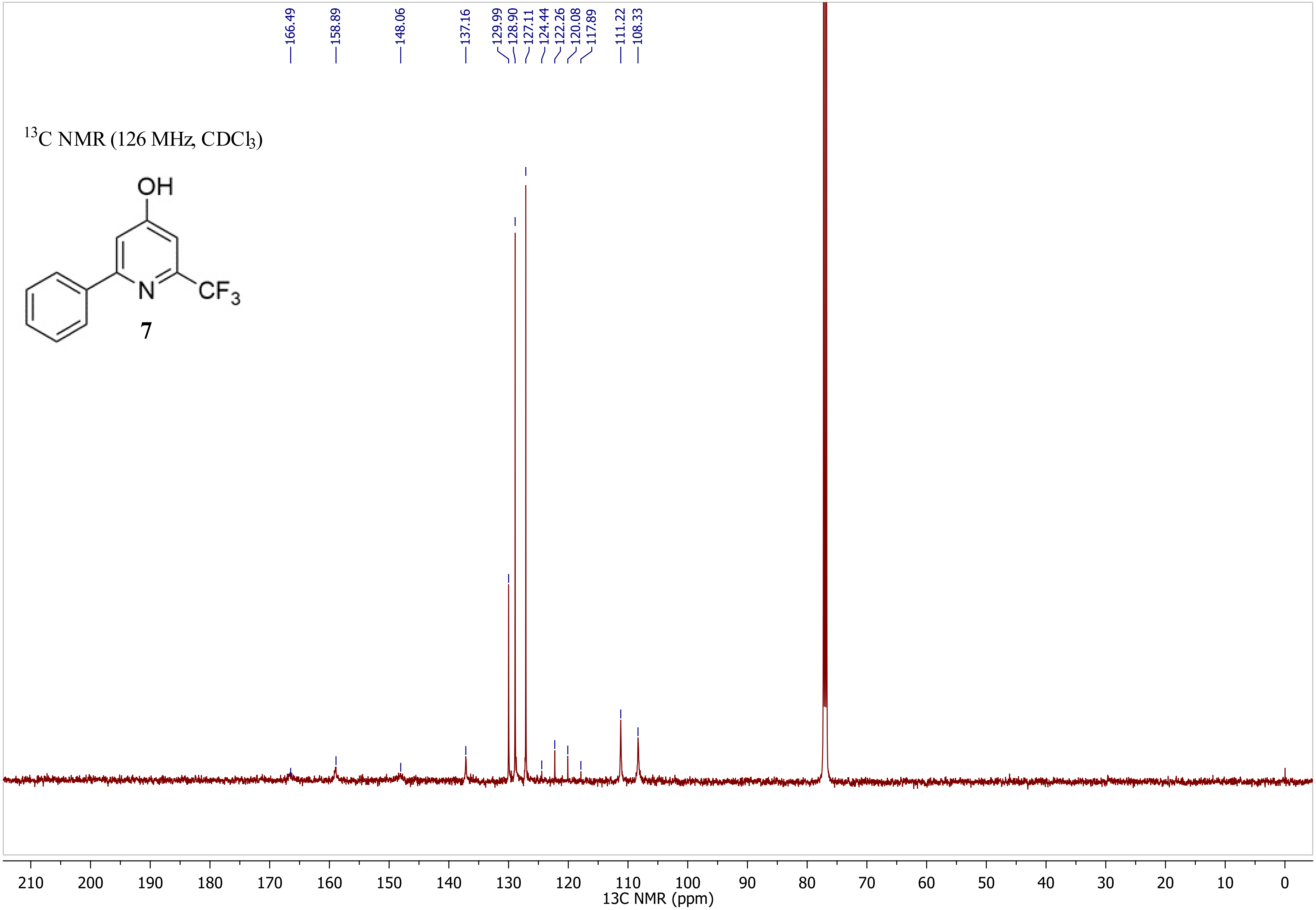
166.49
158.89
148.06
137.16
129.99
128.90
127.11
124.44
122.26
120.08
117.89
111.22
108.33
^{13}C NMR (126 MHz, $CDCl_3$)
OH
N
CF_3
7
210 200 190 180 170 160 150 140 130 120 110 100 90 80 70 60 50 40 30 20 10 0
13C NMR (ppm)

^{1}H NMR (400 MHz, DMSO)

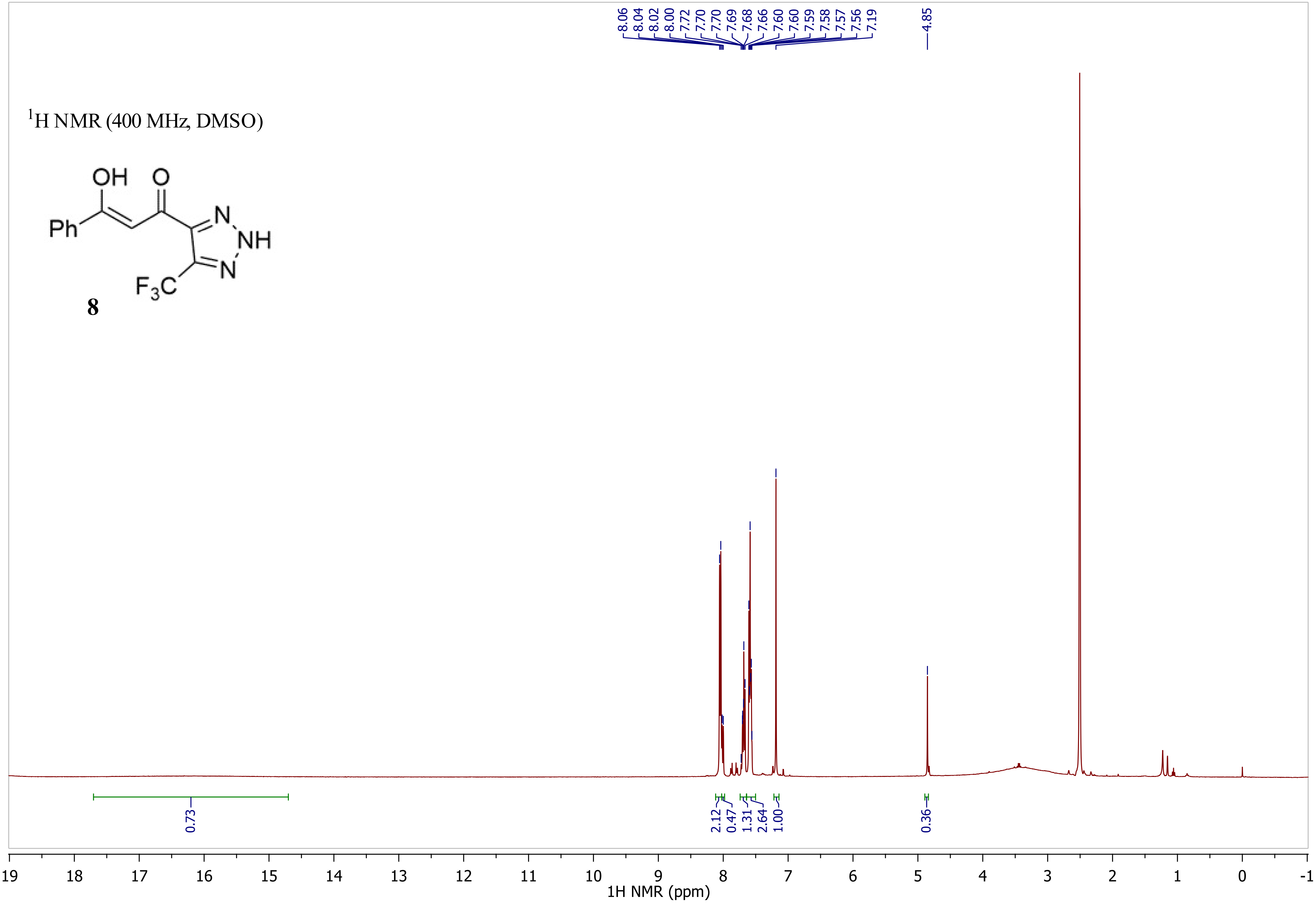

8

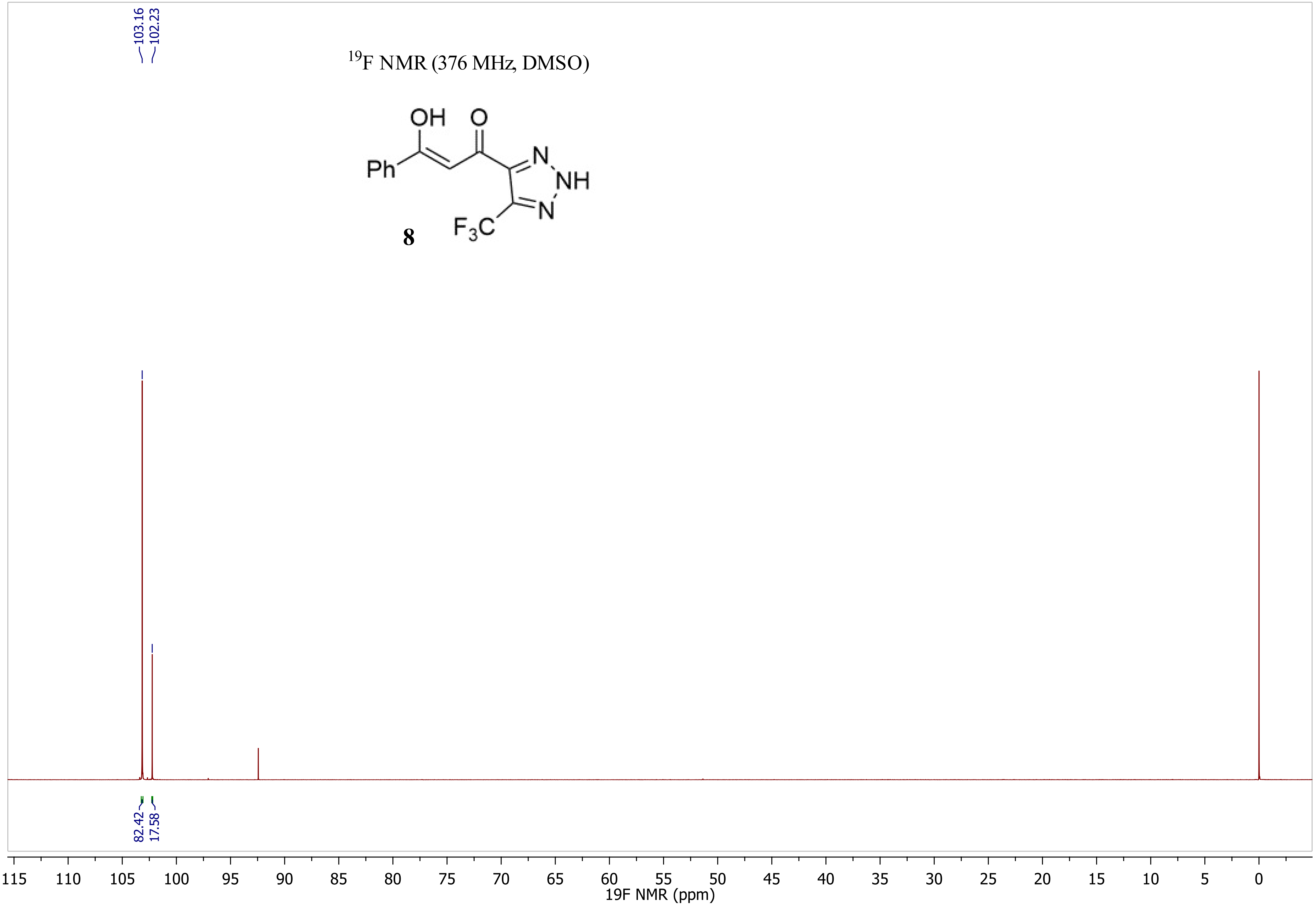

103.16
102.23
^{19}F NMR (376 MHz, DMSO)
OH
O
Ph
N
NH
N
F3C
8
82.42
17.58
115 110 105 100 95 90 85 80 75 70 65 60 55 50 45 40 35 30 25 20 15 10 5 0
19F NMR (ppm)

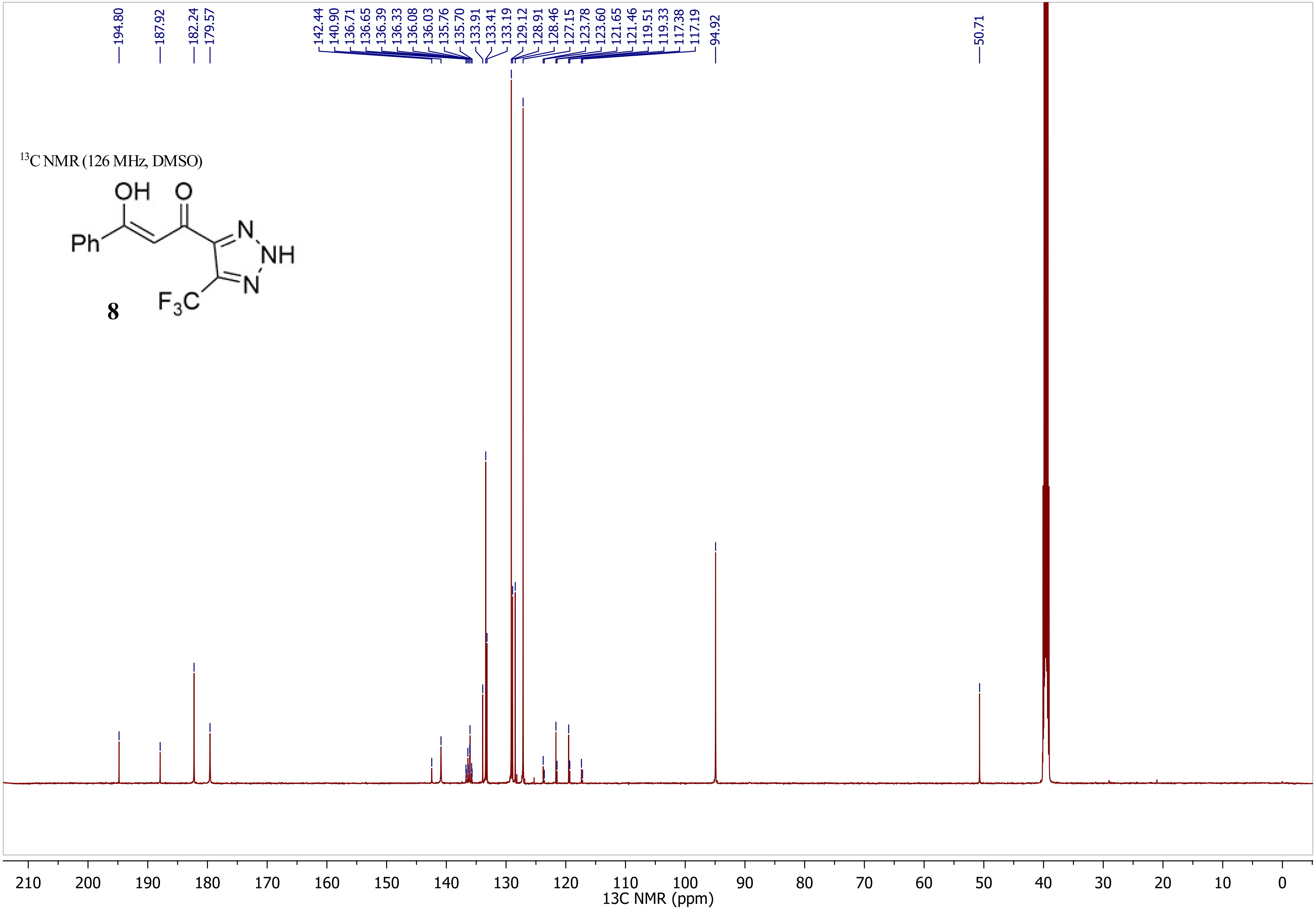
194.80
187.92
182.24
179.57
142.44
140.90
136.71
136.65
136.39
136.33
136.08
136.03
135.76
135.70
133.91
133.41
133.19
129.12
128.91
128.46
127.15
123.78
123.60
121.65
121.46
119.51
119.33
117.38
117.19
94.92
50.71
^{13}C NMR (126 MHz, DMSO)
OH
O
Ph
N
NH
N
F_3C
8
210 200 190 180 170 160 150 140 130 120 110 100 90 80 70 60 50 40 30 20 10 0
13C NMR (ppm)

^{1}H NMR (500 MHz, $CDCl_3$)

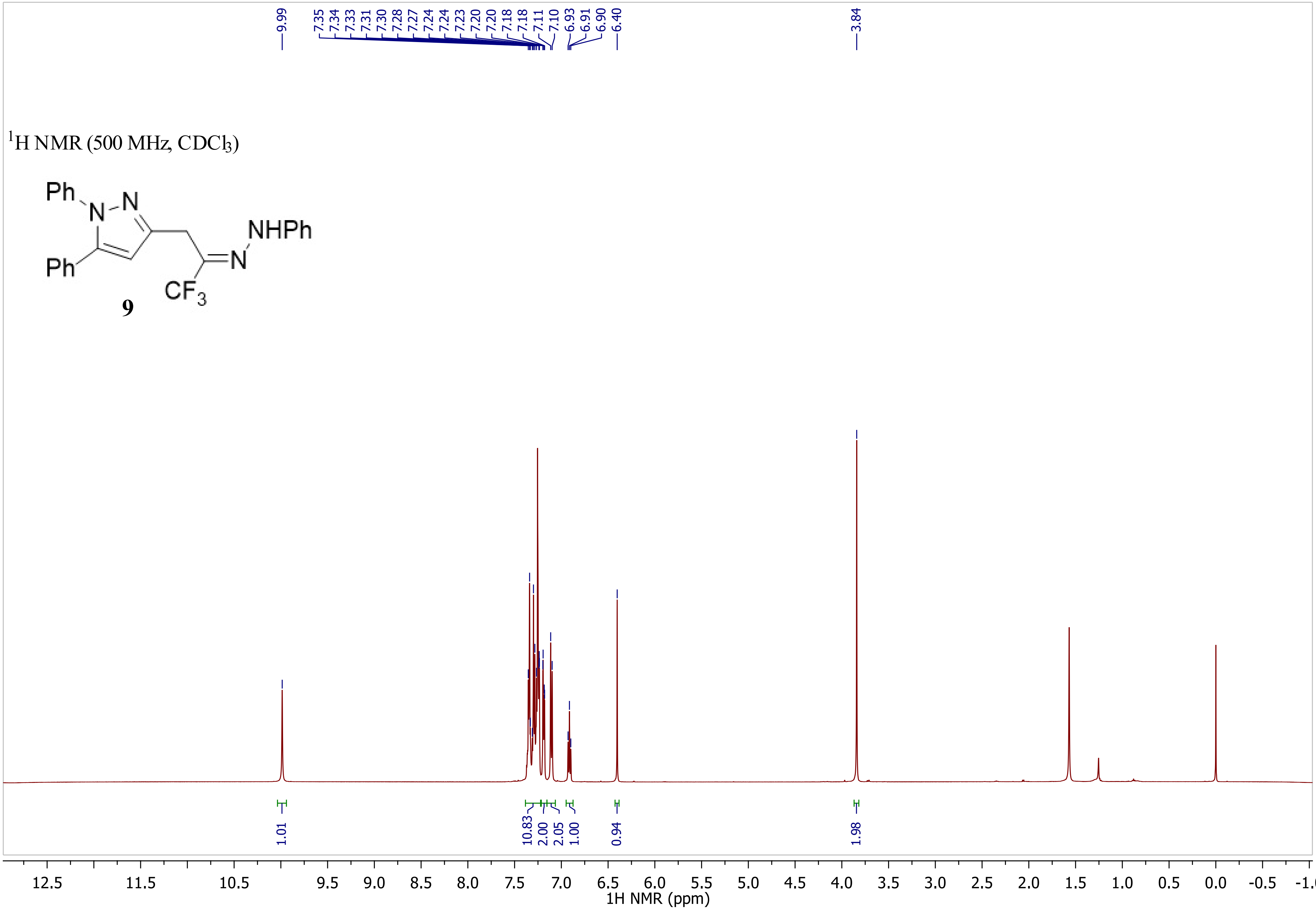

9

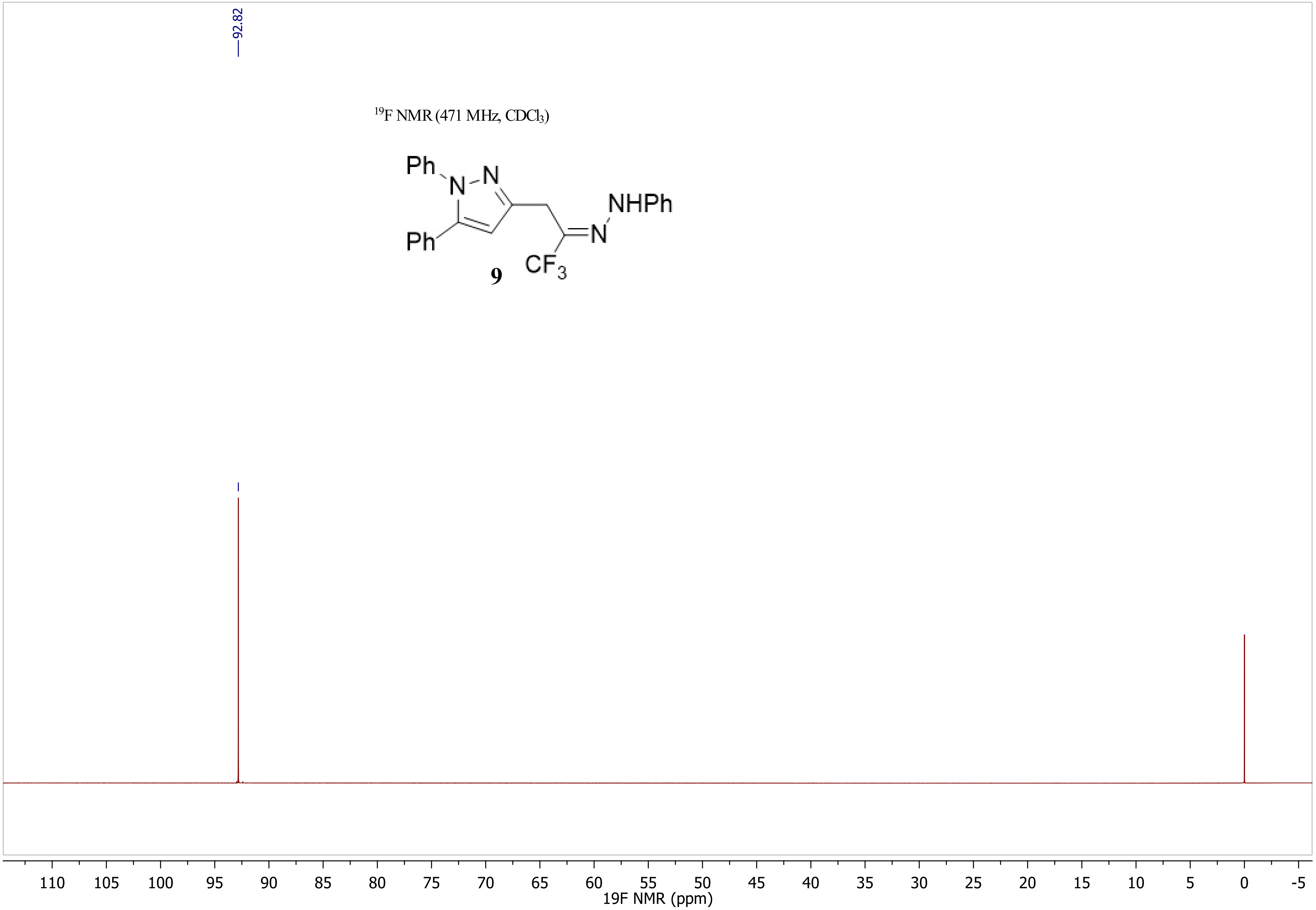

92.82
19F NMR (471 MHz, CDCl3)
Ph
N
N
NHPh
N
Ph
CF3
9
110 105 100 95 90 85 80 75 70 65 60 55 50 45 40 35 30 25 20 15 10 5 0 -5
19F NMR (ppm)

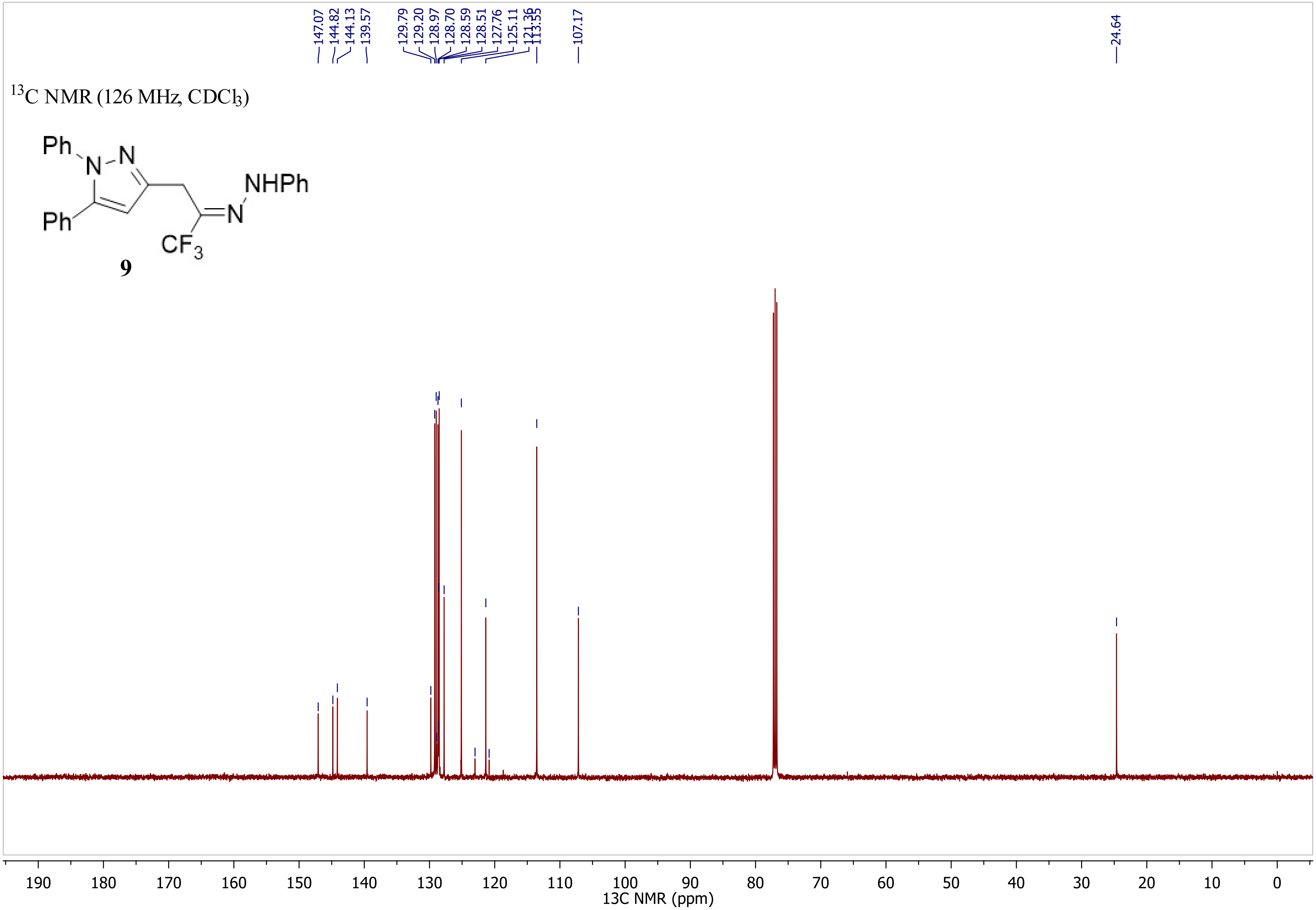

13C NMR (126 MHz, CDCl3)
Ph
N
N
NHPh
Ph
N
CF3
9
147.07
144.82
144.13
139.57
129.79
129.20
128.97
128.70
128.59
128.51
127.76
125.11
121.36
113.55
107.17
24.64
190 180 170 160 150 140 130 120 110 100 90 80 70 60 50 40 30 20 10 0
13C NMR (ppm)
